# Synthesis of Ionizable Calix[4]arenes for Chelation of Selected Divalent Cations

**DOI:** 10.3390/molecules27051478

**Published:** 2022-02-22

**Authors:** Markus Blumberg, Karrar Al-Ameed, Erik Eiselt, Sandra Luber, Constantin Mamat

**Affiliations:** 1 Helmholtz-Zentrum Dresden-Rossendorf, Institut für Radiopharmazeutische Krebsforschung, Bautzner Landstraße 400, D-01328 Dresden, Germany; blumberg.markus@yahoo.de (M.B.); erik.eiselt@gmx.de (E.E.); 2 Fakultät Chemie und Lebensmittelchemie, Technische Universität Dresden, D-01062 Dresden, Germany; 3 Department of Chemistry, University of Zurich, Winterthurerstrasse 190, CH-8057 Zürich, Switzerland; karrar.al-ameed@linacre.ox.ac.uk (K.A.-A.); sandra.luber@chem.uzh.ch (S.L.)

**Keywords:** calixarenes, barium, chelation

## Abstract

Two sets of functionalised calix[4]arenes, either with a 1,3-crown ether bridge or with an open-chain oligo ether moiety in 1,3-position were prepared and further equipped with additional deprotonisable sulfonamide groups to establish chelating systems for selected cations Sr^2+^, Ba^2+^, and Pb^2+^ ions. To improve the complexation behaviour towards these cations, calix[4]arenes with oligo ether groups and modified crowns of different sizes were synthesized. Association constants were determined by UV/Vis titration in acetonitrile using the respective perchlorate salts and logK values between 3.2 and 8.0 were obtained. These findings were supported by the calculation of the binding energies exemplarily for selected complexes with Ba^2+^.

## 1. Introduction

Molecular baskets based on the calixarene backbone were found to be a promising basis to search for suitable macrocyclic ligands especially for divalent metal ions. Calix[4]arenes are metacyclophanes having a hydrophobic cavity between the lower and upper rim, formed by four phenol units connected with methylene units [1,2]. Due to their numerous possibilities for functionalization on the upper as well as on the lower rim [3], this class of macromolecules is known for a wide range of applications [4,5]. Calix[4]arenes were found to act as biologically active compounds as they are used as antibacterial and even antimalarial agents or in cancer chemotherapy [6,7,8,9]. Additionally, they can interact with amino acids or show promising enzyme inhibitory effects [10]. Their ability to form inclusion compounds with neutral molecules [11] or ions [12,13,14,15,16] makes them useful as sensors [17,18], catalysts [19], ligands, or as effective separation agents for ions [20] when resin-bound [21,22,23,24]. Since calix[4]arene derivatives interact particularly strongly with heavy group 2 metals [25,26,27,28,29,30,31], one major field of application consists in their use as extraction agents for nuclear waste treatment [27,32,33,34,35].

In this regard, the calix[4]arene skeleton can be seen as an ideal platform to build an optimized chelator. Two out of the four hydroxy groups on the lower rim are possible to be functionalized with proton-ionizable groups, which leads to the formation of neutral complexes with divalent cations. The remaining two can be furnished with oligo ether groups, either open-chain or bridged, leading to the calixcrown scaffold, which is easily accessible. Using this concept, both the advantages of the electrostatic, macrocyclic, and cryptate effect are unified.

Three divalent cations Sr^2+^, Ba^2+^, and Pb^2+^ are in the focus of our interest, because they all possess radioisotopes with useful nuclear properties for various diagnostic or therapeutic applications in nuclear medicine [36], and are therefore suitable to prepare radiopharmaceuticals. The beta-emitter ^89^Sr is applied as “bone seeker” [37], and ^90^Sr is used for superficial brachytherapy of some cancers [38,39]. ^131^Ba (t_1/2_ = 11.5 d) is a γ-emitter for possible diagnostic uses and is discussed as a bone-scanning agent in scintigraphy [40,41,42]. Furthermore, Ba^2+^ functions as a non-radioactive surrogate [43,44,45] for both alpha-emitters ^223/224^Ra, because of their analogous chemical properties and their radii of similar range [46]. Radium-223 and radium-224, have suitable half-lives (^223^Ra: 11.4 d, ^224^Ra: 3.6 d) and nuclear decay properties (decay chain with four alpha and two beta particles) [47] that make them useful tools for alpha-particle therapy [48]. [^223^Ra]RaCl_2_ is known as Xofigo^®^ and is applied in clinics for the treatment of bone metastases. Furthermore, Pb^2+^ was part of our research. On the one hand, it is the stable end product (^207^Pb and ^208^Pb) of both radium decay chains. On the other hand, ^212^Pb is a promising β^–^-emitter, and a feasible candidate for radiopharmaceutical applications, since it can also be used as an in vivo generator for ^212^Bi, which is a strong alpha emitter [49,50]. No indications were found regarding radiopharmaceutical applications of the light alkaline earth metals beryllium, magnesium, and calcium for therapy or diagnosis.

To provide the ideal cavity for heavy group 2 metals for stable complexation, it is essential to choose a suitable oligo ether length or crown size, respectively, combined with an adequate number of donor sites. Recently, the impact of the crown-6-functionalization of two simple calix[4]crown-6 derivatives has been elaborated [29,30]. The group of R. A. Bartsch focused on extraction of alkaline earth metal cations using functionalized calixcrowns [51,52,53] and found the calix[4]arene-1,3-crown-6 derivatives to be very effective group 2 metal ion extraction agents. The additional impact of two trifluoromethylsulfonylamide groups as proton-ionizing residues was corroborated by them and our research group [26]. Furthermore, these compounds showed high selectivity for Ba^2+^ over the lighter alkaline earth metal or alkali metal ions. However, Ra^2+^ was not investigated. There are only a handful of reports dealing with Ra^2+^ and the efficiency of various ligands including calixarenes as ionophores in nuclear waste management [28,54,55]. Particularly in radiopharmacy, high stability of the complex is urgently important so that a M^2+^-release and the following accumulation in bone tissues is minimized [42,44].

The objective of this research was to evaluate and compare different open-chain and bridged *p*-tert-butylcalix[4]arene derivatives as possible leading compounds that could, upon further modification, yield viable chelators for the selected divalent metal ions Sr^2+^, Ba^2+^, and Pb^2+^ in radiopharmaceutical applications and provide information about comparable stability constants. The existing literature about group 2 metal ligands specifically with radium, is focused mainly on extraction studies. Therefore, the UV titration as reliable and constant method for the calculation of stability constants was used to determine association constants for the respective ions. Additionally, theoretical calculations involving Ba^2+^ as a surrogate for Ra^2+^ were accomplished to underline the results.

## 2. Results and Discussion

### 2.1. Preparation of the Functionalized Calix[4]arenes

**Scheme 1:**
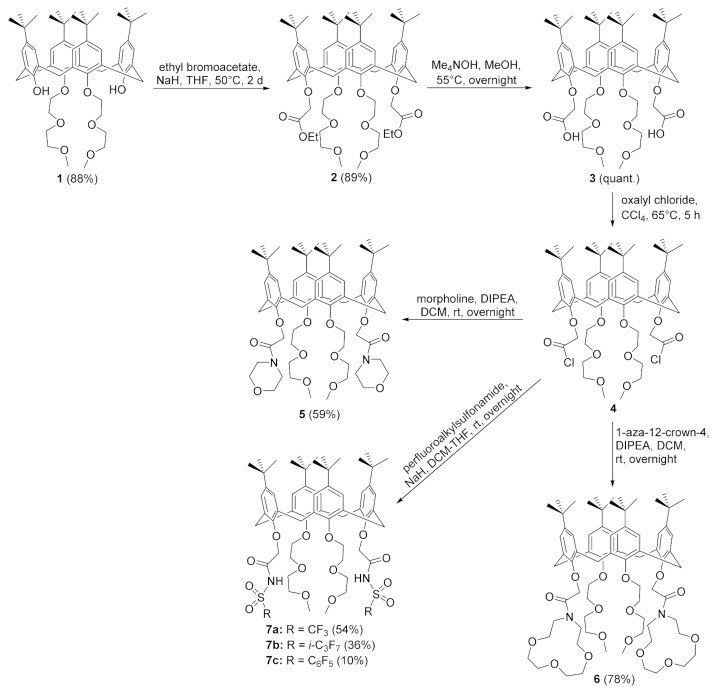
Synthesis of the amide functionalized open-chain calix[4] derivatives **5**, **6**, and **7a**–**c**.

For a better understanding of the complexation ability, two sets of calix[4]arene derivatives either with open-chain or bridged oligoethers were evaluated. The first set of chelators containing the open-chain oligo ether functions was prepared to start from the basic compound **1**, which contains the same number of oxygen donor atoms as calix[4]crown-6 **14a**. The complete synthesis path is described in Scheme 1. Calix **1** is proposed to form complexes with Na^+^ and K^+^ [56]. For the introduction of proton-ionizable groups to improve the complexation behavior, the two remaining free OH groups were modified by alkylation with ethyl bromoacetate to yield calix **2**. In the next step, calix **2** was saponified under basic conditions to yield diacid **3** in quantitative yield without further purification after precipitation with HCl. To introduce the amide functions, compound **3** was then treated with oxalyl chloride to form the dichloride **4**, which was instantly reacted with morpholine or 1,4,7-trioxa-10-azacyclododecane to yield amides **5** and **6**, respectively. Both amines were used for modification to raise the number of donors for complexation and the steric demand. The introduction of proton-ionizable sulfonamides to yield **7a**–**c** follows the same procedure by using trifluoromethyl sulfonamide, perfluoroisopropyl sulfonamide, and perfluorophenyl sulfonamide, respectively, which were deprotonated prior to the reaction with dichloride **4**.

**Scheme 2:**
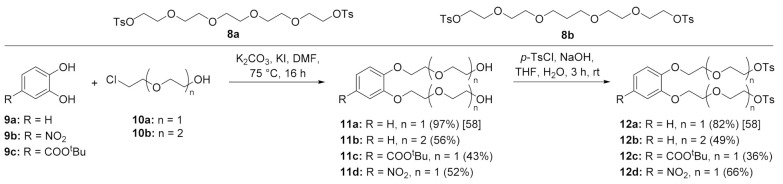
Structure of alkyl ditosylates **8a**,**b** and synthesis of the benzo ditosylates **12a**–**d**.

To check the influence of the resulting cavity, the flexibility of the functionalized crown ether bridge on the association constant and the possibility to introduce further functional groups at the crown, the second set of chelators is prepared based on the bridging moiety like a simple crown, benzocrown or aza crown. For this purpose, the respective bridging compounds 3,6,10,13-tetraoxapentadecane-1,15-diyl ditosylate (**8b**) [57] and the catechol derivatives **12a** and **12b** [58] were prepared according to literature procedures. Additionally, ditosylates **12c**,**d** resulting from functionalized catecholes **9b**,**c** were prepared to allow a later functionalisation of the calix-bridge using conventional ligation reactions. The preparation procedure is outlined in Scheme 2.

**Scheme 3:**
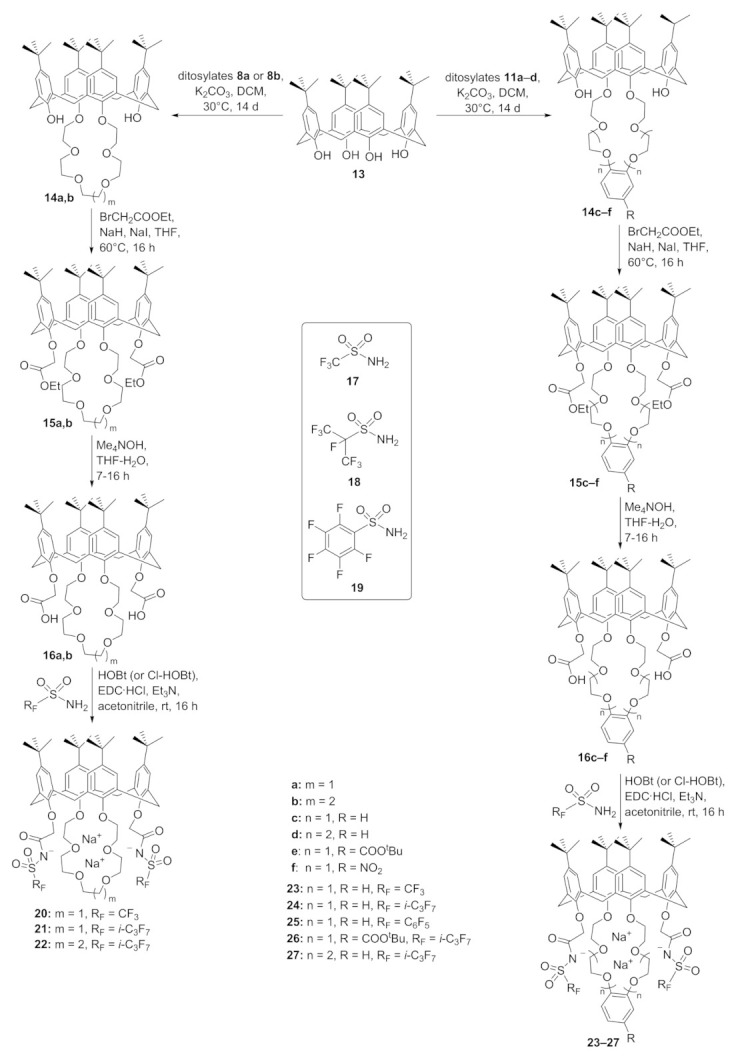
Preparation of the proton-ionizable calix[4]crown derivatives **20**–**27** with perfluorosulfonamide functions.

For the connection of the functionalized crown ethers with the calix[4]arene skeleton, the resulting ditosylates **8a**,**b** and **12a**–**d** of the oligo ethers were reacted with ^t^Bu-calix[4]arene (**13**) under basic conditions using K_2_CO_3_ in dichloromethane (DCM) to prepare the calix-crown-6 derivatives **14a**,**b** and the calix-benzocrown-6 derivatives **14c**–**f** in yields of 36–81%. The complete synthesis path is described in Scheme 3. The two remaining free OH groups of the calix-crowns **14a**–**f** were alkylated with ethyl bromoacetate to yield **15a**–**f**. In the next step, they were saponified under basic conditions to yield the respective diacid derivatives **16a**–**f** in mostly quantitative yields without further purification after precipitation with HCl. To introduce the proton-ionizable fluorinated sulfonamide functions, an amide coupling strategy using EDC and (Cl-)HOBt was applied. Thus, compounds **16a**–**f** were dissolved in anhydrous acetonitrile and reacted with the respective perfluorinated sulfonamides **17**–**19** at ambient temperature using the aforementioned coupling agents to form calix-crowns **20**–**22** and the modified calix-benzocrowns **23**–**27** in yields of 27–97%.

### 2.2. The Influence of the Crown Type

**Figure 1:**
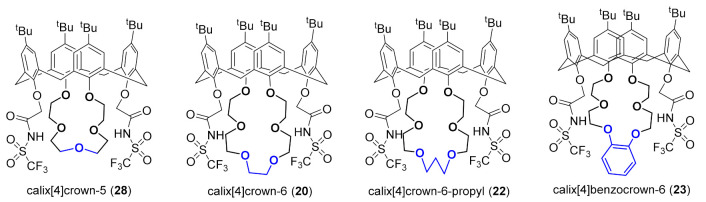
Calix[4]arene derivatives with modified crown ethers were used in the calculations.

The influence of lower-rim crown modifications on the metal-calixcrown-coordination was next checked. For this purpose, reaction binding energies of Ba^2+^ with four selected crown-bearing calixarenes were calculated (Figure 1). That involved calix[4]crown-6 **20** and additionally benzo-crown derivative **23** with the modification on adding an aromatic six-membered ring at the bottom of the crown, propylene derivative **22** with an extended crown by a CH_2_ group, and calix[4]crown-5 **28** lacking an ethoxy unit (CH_2_CH_2_O). As the result of the calculated binding energies (corrected for basis set superposition error) shown in Table 1, the energetically most favored environment to host the metal ion is found for calix[4]crown-6 **20**, while in the other three compounds, the metal-crown binding is weakened up to about 10%. Furthermore, the lowest binding energy is found for the barium ion and calix[4]crown-5. At first glance, the crown-5 might appear to feature an optimal size to host ions like Ba^2+^, as the size of the macrocycle cavity relative to the ionic radius is often used as a common parameter to rationalize and design new ligands in host-guest chemistry [59]. Considering the geometrical parameters given in Table S1, the crown-5 cavity seems to enclose the Ba^2+^ ion better than the crown-6; however, the calculated binding energies show a reversed trend. Our calculations are in accordance with previous studies in the field of host-guest chemistry of crown ethers [60]. Islam et al., for instance, showed that Na^+^ binds more tightly to a crown-6 body although the crown-5 hole’s size matches the sodium ion radius better compared to the one of crown-6 [61].

### 2.3. NMR Investigations

**Figure 2:**
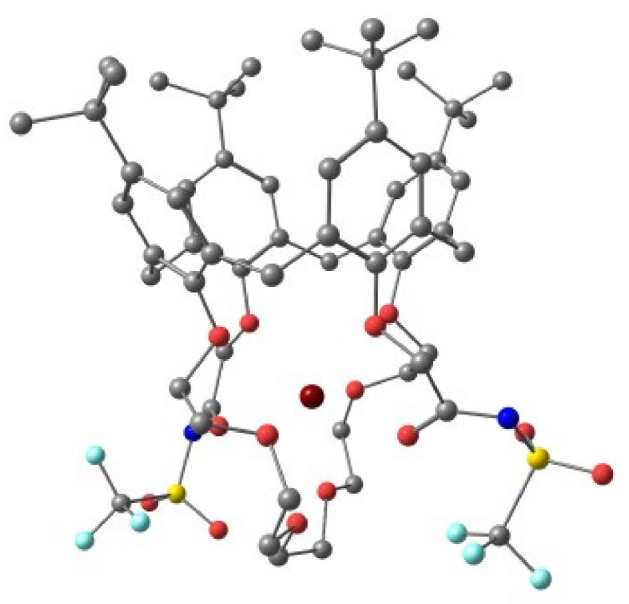
The optimized geometrical structure of the Ba-complex **Ba-20** (hydrogen atoms are hidden for clarity; the colors of the atoms are: carbon; grey, fluorine; green, sulfur; yellow, nitrogen; blue, oxygen; red, barium; brown).

**Figure 3:**
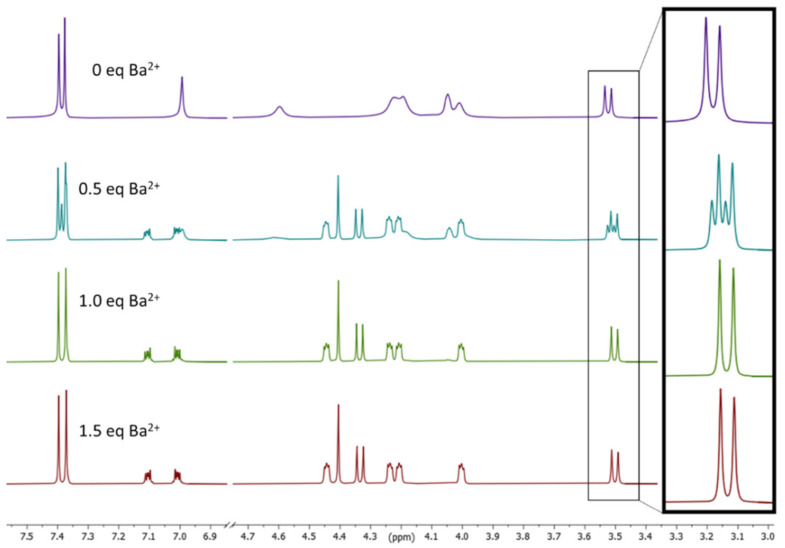
^1^H NMR spectra showing the change in chemical shifts by treatment of calix **24** with different equivalents of Ba(ClO_4_)_2_ in acetonitrile-d_3_.

To determine the stability constants for the complexation of Ba^2+^, Sr^2+^, and Pb^2+^, a reliable method was developed in the past by our group using ^1^H NMR spectroscopy. Due to the different ^1^H NMR spectra, which were recorded for the respective complexes in comparison with the ligands, a ^1^H NMR titration method was established [26,29,30]. For this purpose, the compounds were dissolved in acetonitrile-d_3_ and treated in portions with an acetonitrile-d_3_ solution containing Ba(ClO_4_)_2_. It was observed, that after addition of 0.5 equivalents of Ba salt, a new set of signals appeared, belonging to the respective Ba-complex exemplarily shown for calix **24** in Figure 2. This leads to two separate species: ligand **24** and complex **Ba-24** in the ^1^H NMR spectrum (see, for instance, the difference of the methylene protons of the calix skeleton in the box of Figure 3). After the addition of 1 eq. of Ba(ClO_4_)_2_, no ligand was detectable anymore, which leads to the assumption of a complex with 1:1 stoichiometry. The situation in Figure 2 is a consequence of a slow exchange on the NMR time scale and is therefore not suitable for a logK determination by NMR titration for our calix compounds [62]. Next, a more reliable titration method based on UV/vis spectroscopy was used instead to determine the association constants.

### 2.4. UV Titration Studies

**Figure 4:**
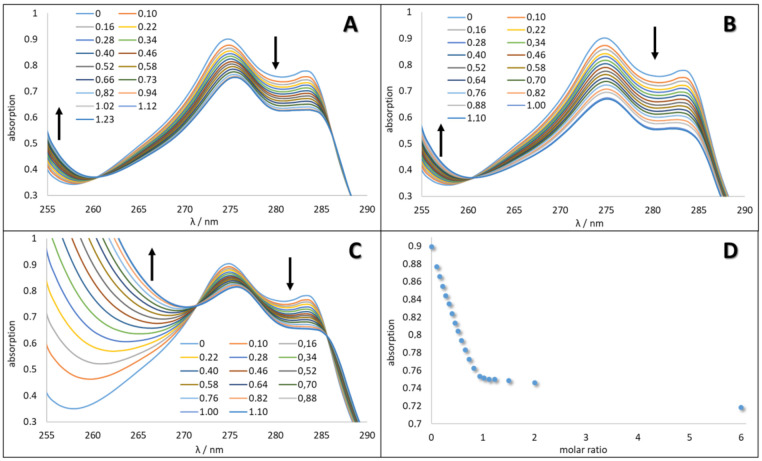
UV/Vis spectra of calix[4]arene **21** at different M(ClO_4_)_2_ concentrations (**A**) M = Ba, (**B**) M = Sr, (**C**) M = Pb for titration measured in acetonitrile. (**D**) Titration curve with change of the absorption using compound **21** at different Ba(ClO_4_)_2_ concentrations, measured in acetonitrile at λ = 275 nm.

To determine the association constants by UV titration, the open-chain calix[4]arenes **7a**,**b** as well as the calix[4]crowns **20**–**22** and benzocrown compounds **23**–**25** and **27** were dissolved in acetonitrile and aliquots of M(ClO_4_)_2_ with M = Ba, Sr, Pb in acetonitrile were added, which caused a clear change in the UV absorption. This behavior is shown in Figure 4 based on the example of compound **21** and its complexes **Ba-21**, **Sr-21** and **Pb-21**. Considering one wavelength with a high change in absorption, the stoichiometry of the complex was determined from the diagram titration curve (Figure 4, exemplarily for **Ba-21**). All titration experiments showed the presence of a complex with a 1:1 stoichiometry, formed by functionalized calix[4]crowns with M(ClO_4_)_2_ except for calix **6** (see Supplementary Materials), as the slope of the graph is changing at the 1:1 molar ratio. The results from the evaluation of the association constants are summarized in Table 2 (all UV titration experiments are expressed in the Supplementary Materials).

The association constants of the bridged derivatives **20**–**25** and **27** with Sr^2+^, Ba^2+^ and Pb^2+^ showing values up to 8.0 and were higher as for the basic complexes **Ba-14a** (logK 4.6), **Sr-14a** (logK: 4.3), and **Pb-14a** (logK 3.3) [26] without additional side functions due to the increasing number of donor atoms and the additional macrocyclic effect. Interestingly, higher association constants were obtained for the benzocrown derivatives **23**–**25** in comparison to the basic crown derivatives **20**–**22**. The calix derivatives with benzocrown bridge led to more stable complexes showing higher stability constants of approximately one magnitude (e.g., **Sr-21**: 5.0 vs. **Sr-24**: 6.4 (C_3_F_7_) or **Ba-20**: 5.7 vs. **Ba-23**: 6.6 (CF_3_)) for the group 2 metal ions. Moreover, the propylene bridge in calix **22** has only a marginal influence on the association constant regarding the respective Ba-complexes **Ba-21** and **Ba-22**, but a strong influence when comparing the Pb-complexes (logK_(**Pb-21**)_ = 5.5 vs. logK_(**Pb-22**)_ = 7.4). 

Additionally, a high value is even found for calix **5** with morpholine modification and its respective complexes with Ba^2+^ and Pb^2+^ possibly due to the higher number of donor atoms. The constants for the open-chain derivatives **Ba-7a**,**b** are lower or equal to the value found for **14a** in contrast to the values found for **Pb-7a**,**b**, which were higher. Differences between the M^2+^ complexes from the same ligand arose from the different ion radii as well as from the chemical behavior (HSAB concept), as Sr^2+^ and Ba^2+^ belong to the alkaline earth metals (group 2) and Pb^2+^ is a group 4 metal ion and therefore, show a softer ion character.

## 3. Materials and Methods

### 3.1. General

All chemicals were purchased from commercial suppliers and used without further purification unless otherwise specified. Anhydrous THF was purchased from Acros, anhydrous Ba(ClO_4_)_2_ was purchased from Alfa Aesar, and deuterated solvents were purchased from deutero GmbH. Compounds **8b** [57], **9c** [63], **11a**, **12a** [59], **11b**, **12b** [64], **14a**, **15a**, **16a**, and **20** [26] were prepared according to the literature. NMR spectra of all compounds were recorded on an Agilent DD2-400 MHz NMR or an Agilent DD2-600 MHz NMR spectrometer with ProbeOne. Chemical shifts of the ^1^H, ^19^F, and ^13^C spectra were reported in parts per million (ppm) using TMS as an internal standard for ^1^H/^13^C and CFCl_3_ for ^19^F spectra. Mass spectrometric (MS) data were obtained on a Xevo TQ-S mass spectrometer (Waters) by electron spray ionization (ESI). The melting points were determined on a Galen III melting point apparatus (Cambridge Instruments & Leica) and are uncorrected. TLC detections were performed using Silica Gel 60 F_254_ sheets (Merck, Darmstadt, Germany). TLCs were developed by visualization under UV light (λ = 254 nm). Chromatographic separations were accomplished by using an automated silica gel column chromatography system Biotage Isolera Four and appropriate Biotage KP-SIL SNAP columns. UV/Vis- measurements were realized at a Specord 50 by Analytik Jena. The calculation of the stability constants was accomplished using HypSpec 1.1.18.

### 3.2. The Computational Methodology

The calculations of calixcrowns were performed using Kohn-Sham DFT. The general gradient approximation functional BP86 [65,66] was used for geometry optimizations and the B3LYP [67,68] to calculate single point energies. With the latter settings, we have used a continuum solvation COSMO model for acetonitrile (dielectric constant ζ = 37.5) in order to approximately include the solvent environment used in the experiment. Ahlrichs’ triple zeta valence polarized (def2-TZVP) [69] basis set was employed as well as the resolution of identity (RI) approach and corresponding auxiliary basis sets [70], along with Grimme’s D3 dispersion correction [71]. Since the def2-TZVP was not available for radium, we have used the split valence polarized (def-SVP) basis set [72] for both Ra^2+^ and Ba^2+^ in the calculations that involved a comparison between their binding energies. Effective core potentials have been used for the heavy metals in order to account for scalar relativistic effects. The Turbomole 7.3 package [73] was used for all calculations in this study. To prevent the over-stabilization of final energies, basis set superposition error (BSSE) was considered. Binding energies were calculated as differences of electronic energies E via E(complex)-[E(calixcrown)+E(M^2+^)].

### 3.3. Chemical Syntheses

**5**,**11**,**17**,**23-Tetrakis(*tert*-butyl)-25**,**27-dihydroxy-26**,**28-bis[2-(2-methoxyethoxy)ethoxy]calix[4]arene 1.** Under Ar, *tert*-butylcalix[4]arene (2 g, 3.08 mmol) was dissolved in anhydrous acetonitrile (40 mL) and 2-(2-methoxyethoxy)ethyl tosylate (1.86 g, 6.78 mmol) and K_2_CO_3_ (1.06 g, 7.7 mmol) added. The reaction mixture was stirred at 72 °C for 4 d. After cooling, the solvent was changed to chloroform (40 mL), the insoluble ingredients were removed by filtration and the organic phase was washed with 10% aqueous HCl (2 × 30 mL) and water (2 × 30 mL). After separation, the organic phase was dried over Na_2_SO_4_ and the crude product was purified using column chromatography (petroleum ether:acetone = 10:1 → 5:1) to obtain **1** as colorless oil, which tends to crystallize after standing (2.31 g, 88%); mp 100 °C; R*_f_* 0.46 (petroleum ether:acetone = 2:1); ^1^H NMR (600 MHz, CDCl_3_): δ 0.95 (s, 18 H, ^t^Bu), 1.30 (s, 18 H, ^t^Bu), 3.30 (d, ^2^*J* = 13.1 Hz, 4 H, CH_2_Ar), 3.39 (s, 6 H, OCH_3_), 3.61–3.64 (m, 4 H, OCH_2_), 3.83–3.86 (m, 4 H, OCH_2_), 3.96–4.00 (m, 4 H, OCH_2_), 4.16–4.19 (m, 4 H), 4.36 (d, ^2^*J* = 13.1 Hz, CH_2_Ar), 6.77 (s, 4 H, ArH), 7.06 (s, 4 H, ArH), 7.15 (s, 2 H, OH); ^13^C NMR (151 MHz, CDCl_3_): δ 31.2 (^t^Bu), 31.9 (^t^Bu), 33.9 (C_q_), 34.0 (C_q_), 59.2 (OCH_3_), 70.1 (OCH_2_), 71.1 (OCH_2_), 72.3 (OCH_2_), 75.4 (OCH_2_), 125.1, 125.6 (2 × CH_Ar_), 128.0 (C Ar), 132.7, 141.4, 146.9, 150.0, 150.8 (6 × C_Ar_) ppm; MS (ESI+) *m*/*z* = 871 (M^+^ + NH_4_), 876 (M^+^ + Na), 892 (M^+^ + K).

**5**,**11**,**17**,**23-Tetrakis(*tert*-butyl)-25**,**27-bis(2-ethoxy-2-oxoethoxy)-26**,**28-bis[2-(2-methoxyethoxy)ethoxy]calix[4]arene 2.** Under Ar, compound **1** (1 g, 1.17 mmol) was dissolved in anhydrous THF (40 mL) and NaH (234 mg, 5.86 mmol, 60% in mineral oil) added and stirred at rt for 30 min. Afterwards, a solution of ethyl bromoacetate (978 mg, 5.86 mmol) in 5 mL of THF was added and the resulting mixture stirred at 50 °C for 2 d. After cooling to rt, the solvent was changed to chloroform (40 mL), the insoluble ingredients were removed by filtration and the organic phase was washed with 10% aqueous HCl (3 × 30 mL) and water (2 × 30 mL). After separation, the organic phase was dried over Na_2_SO_4_ and the crude product was purified using column chromatography (petroleum ether:acetone = 4:1) to obtain **2** as light-yellow oil (1.07 g, 89%); R*_f_* 0.52 (petroleum ether:acetone = 2:1); ^1^H NMR (400 MHz, CDCl_3_): δ 1.03 (s, 18 H, ^t^Bu), 1.12 (s, 18 H, ^t^Bu), 1.29 (t, ^3^*J* = 7.1 Hz, 6 H, CH_3_), 3.15 (d, ^2^*J* = 12.9 Hz, 4 H, CH_2_Ar), 3.37 (s, 6 H, OCH_3_), 3.53–3.57 (m, 4 H, OCH_2_), 3.66–3.69 (m, 4 H, OCH_2_), 3.94 (t, ^3^*J* = 5.6 Hz, 4 H, OCH_2_), 4.12 (t, ^3^*J* = 5.6 Hz, 4 H, OCH_2_), 4.22 (q, ^3^*J* = 7.2 Hz, 4 H, OCH_2_), 4.67 (d, ^2^*J* = 12.9 Hz, 4 H, CH_2_Ar), 4.77 (s, 4 H, CH_2_C=O), 6.70 (s, 4 H, ArH), 6.83 (s, 4 H, ArH); ^13^C NMR (101 MHz, CDCl_3_): δ 14.4, 31.5, 31.6 (3 × CH_3_), 31.7 (CH_2_Ar), 33.9 (C_q_), 34.0 (C_q_), 59.2 (OCH_3_), 60.5 (OCH_2_), 70.4 (OCH_2_), 70.7 (OCH_2_), 71.3 (CH_2_C=O), 72.2 (OCH_2_), 73.2 (OCH_2_), 125.1 (CH_Ar_), 125.5 (CH_Ar_), 133.5, 134.0, 144.8, 145.2, 153.2, 153.4 (6 × C_Ar_), 170.8 (C=O) ppm; MS (ESI+) *m*/*z* = 1042 (M^+^ + NH_4_), 1047 (M^+^ + Na).

**5**,**11**,**17**,**23-Tetrakis(*tert*-butyl)-25**,**27-bis(carboxymethoxy)-26**,**28-bis[2-(2-methoxyethoxy)ethoxy]calix[4]arene 3.** Compound **2** (213 mg, 0.21 mmol) was dissolved in THF (20 mL), a solution of Me_4_NOH ∙ 5 H_2_O (264 mg, 1.46 mmol) in methanol (1 mL) was added and the reaction mixture was stirred at 55 °C overnight. The major part of the solvent was removed and the product was precipitated with ice-cold aqueous HCl (6 M). The solid residue was filtered and washed with water (20 mL) and dissolved in chloroform (20 mL). The organic phase was washed with brine (3 × 10 mL) and 10% aqueous HCl (2 × 10 mL). After separation, the organic phase was dried over Na_2_SO_4_, the solvent was removed and the product was obtained as pale-orange solid (200 mg, >99%); mp 104–106 °C; ^1^H NMR (400 MHz, CDCl_3_): δ 0.83 (s, 18 H, ^t^Bu), 1.34 (s, 18 H, ^t^Bu) 3.24 (d, ^2^*J* = 13 Hz, 4 H, CH_2_Ar), 3.38 (s, 6H, OCH_3_), 3.23–3.26 (m, 4 H, OCH_2_), 3.73–3.75 (m, 4H, OCH_2_), 3.83–3.85 (m, 4 H, OCH_2_), 3.99–4.01 (m, 4 H, OCH_2_), 4.45 (d, ^2^*J* = 13.0 Hz, 4 H, CH_2_Ar), 4.75 (s, 4 H, CH_2_C=O), 6.56 (s, 4 H, ArH), 7.17 (s, 4 H, ArH); ^13^C NMR (101 MHz, CDCl_3_): δ 31.09 (CH_2_Ar), 31.14 (CH_3_), 31.8 (CH_3_), 33.9 (C_q_), 34.4 (C_q_), 59.2 (OCH_3_), 69.8 (OCH_2_), 70.5 (OCH_2_), 72.2 (OCH_2_), 72.3 (CH_2_C=O), 76.2 (OCH_2_), 125.5 (CH_Ar_), 126.2 (CH_Ar_), 132.3, 135.0, 146.1, 147.3, 150.2, 153.3 (6 × C_Ar_), 170.6 (C=O); MS (ESI−) *m*/*z* = 968 (M^–^ − H).

**5**,**11**,**17**,**23-Tetrakis(*tert*-butyl)-26**,**28-bis[2-(2-methoxyethoxy)ethoxy]-25**,**27-bis(2-morpholino-2-oxoethyl)calix[4]arene 5.** Under Ar, compound **3** (100 mg, 0.103 mmol) was dissolved in CCl_4_ (5 mL), oxalyl chloride (0.75 g, 5.88 mmol) dropwise added and the reaction mixture stirred at 65 °C for 5 h. After cooling to rt, the remaining oxalyl chloride and the solvent were removed under high vacuum and anhydrous DCM (5 mL) added. After cooling the solution to 0 °C, morpholine (22 mg, 0.26 mmol) and DIPEA (40 mg, 0.31 mmol) were added to resulting mixture was allowed to come to rt and was stirred at rt overnight. Afterwards, DCM (20 mL) was added and the organic phase washed with saturated hydrogen carbonate solution (20 mL), aqueous HCl (10%, 20 mL), and brine (15 mL). After separation, the organic phase was dried over Na_2_SO_4_ and the crude product was purified using automated column chromatography (chloroform: methanol = 0 → 15%) to obtain **5** as colorless oil (67 mg, 59%); R*_f_* 0.16 (chloroform:methanol = 9:1); ^1^H NMR (400 MHz, CDCl_3_): δ 1.04 (s, 18 H, ^t^Bu), 1.09 (s, 18 H, ^t^Bu), 3.14 (d, ^2^*J* = 12.8 Hz, 4 H, CH_2_Ar), 3.34 (s, 6 H, OCH_3_), 3.37–3.44 (m, 4 H, NCH_2_), 3.49–3.55 (m, 8 H, OCH_2_), 3.59–3.71 (m, 12 H, NCH_2_, OCH_2_), 3.97 (t, ^3^*J* = 5.7 Hz, 4 H, OCH_2_), 4.18 (t, ^3^*J* = 5.7 Hz, 4 H, OCH_2_), 4.60 (d, ^2^*J* = 12.8 Hz, 4 H, CH_2_Ar), 4.78 (s, 4 H, CH_2_C=O), 6.74 (s, 4 H, ArH), 6.78 (s, 4 H, ArH); ^13^C NMR (101 MHz, CDCl_3_): δ 31.5 (CH_3_), 31.56 (CH_3_), 31.62 (CH_2_Ar), 33.9 (C_q_), 34.0 (C_q_), 42.0 (NCH_2_), 45.9 (NCH_2_), 59.1 (OCH_3_), 66.8 (OCH_2_), 67.0 (OCH_2_), 70.1 (OCH_2_), 70.5 (OCH_2_), 71.6 (CH_2_C=O), 72.2 (OCH_2_), 72.8 (OCH_2_), 125.2 (CH_Ar_), 125.4 (CH_Ar_), 133.4, 133.9, 144.8, 145.1, 153.1, 153.6 (6 × C_Ar_), 168.3 (C=O) ppm; MS (ESI+): *m*/*z* = 1130 (M^+^ + Na).

**5**,**11**,**17**,**23-Tetrakis(tert-butyl)-26**,**28-bis[2-(2-methoxyethoxy)ethoxy]-25**,**27-[2-(1**,**4**,**7-trioxa-10-azacyclododecan-10-yl)-2-oxoethyl]calix[4]arene 6.** Under Ar, compound 3 (100 mg, 0.10 mmol) was dissolved in CCl_4_ (5 mL), oxalyl chloride (0.75 g, 5.88 mmol) dropwise added and the reaction mixture stirred at 65 °C for 5 h. After cooling to rt, the remaining oxalyl chloride and the solvent were removed under high vacuum and anhydrous DCM (5 mL) added. After cooling the solution to 0 °C, 1-aza-12-crown-4 (45 mg, 0.26 mmol) and DIPEA (40 mg, 0.31 mmol) were added to resulting mixture was allowed to come to rt and was stirred at rt overnight. Afterwards, DCM (20 mL) was added and the organic phase washed with saturated hydrogen carbonate solution (15 mL), aqueous HCl (10%, 15 mL), and brine (15 mL). After separation, the organic phase was dried over Na_2_SO_4_ and the crude product was purified using automated column chromatography (chloroform:methanol = 0 → 15%) to obtain **6** as colorless solid (103 mg, 78%); R*_f_* 0.29 (chloroform:methanol = 85:15); ^1^H NMR (400 MHz, CDCl_3_): δ = 1.08 (s, 18 H, ^t^Bu), 1.13 (s, 18 H, ^t^Bu), 3.26–3.32 (m, 10 H, ArCH_2_, OCH_3_), 3.34–3.42 (m, 4 H, CH_2_), 3.44–3.48 (m, 4 H, CH_2_), 3.50–3.55 (m, 4 H, CH_2_), 3.58–3.67 (m, 16 H, CH_2_), 3.69–3.76 (m, 8 H, CH_2_), 3.86–3.94 (m, 8 H, CH_2_), 4.12–4.18 (m, 4 H, CH_2_), 4.34 (d, 4 H, ^2^*J* = 12.5 Hz, CH_2_Ar), 4.78 (s, 4 H, CH_2_C=O), 7.05 (s, 4 H, ArH), 7.07 (s, 4 H, ArH) ppm; ^13^C NMR (101 MHz, CDCl_3_): δ = 30.0 (CH_2_Ar), 31.3 (CH_3_), 31.4 (CH_3_), 34.2 (C_q_), 34.3 (C_q_), 49.3 (CH_2_), 50.0 (CH_2_), 59.0 (OCH_3_), 68.6, 68.8, 69.1, 69.5, 69.9, 70.0, 70.1, 70.9, 72.0 (9 × CH_2_), 73.9 (CH_2_C=O), 75.2 (CH_2_), 125.7 (CH_Ar_), 125.9 (CH_Ar_), 134.6, 134.8, 147.7, 148.3, 148.5, 150.0 (6 × C_Ar_), 170.1 (C=O) ppm.

**5**,**11**,**17**,**23-Tetrakis(*tert*-butyl)-26**,**28-bis[2-(2-methoxyethoxy)ethoxy]-25**,**27-[2-(trifluoromethylsulfonamido)-2-oxoethyl]calix[4]arene 7a.** Under Ar, compound 3 (100 mg, 0.10 mmol) was dissolved in CCl_4_ (5 mL), oxalyl chloride (0.75 g, 5.88 mmol) dropwise added and the reaction mixture stirred at 65 °C for 5 h. After cooling to rt, the remaining oxalyl chloride and the solvent were removed under high vacuum, anhydrous DCM (5 mL) added and the solution cooled to 0 °C. In a second flask, trifluoromethanesulfonamide (39 mg, 0.26 mmol) was dissolved in anhydrous THF (2 mL) and NaH (41 mg, 1.03 mmol, 10 eq., 60% in mineral oil) was added. After stirring for 15 min, this solution was added to the DCM solution at 0 °C and the combined mixture was stirred for 1.5 h at rt. The solvent was changed to chloroform (20 mL) and the organic phase washed with saturated hydrogen carbonate solution (15 mL), aqueous HCl (10%, 15 mL), and brine (15 mL). After separation, the organic phase was dried over Na_2_SO_4_ and the crude product was purified using automated column chromatography (petroleum ether:ethyl acetate = 0 → 30%) to obtain **7a** as colorless solid (68 mg, 54%); mp 113–115 °C; R*_f_* 0.76 (petroleum ether:ethyl acetate = 1:1); ^1^H NMR (400 MHz, CDCl_3_): δ 1.04 (s, 18 H, ^t^Bu), 1.20 (s, 18 H, ^t^Bu), 3.34 (d, ^2^*J* = 12.4 Hz, 4 H, CH_2_Ar), 3.34 (s, 6 H, OCH_3_), 3.59 (t, ^3^*J* = 4.3 Hz, 4 H, OCH_2_), 3.69–3.78 (m, 8 H, OCH_2_), 4.08–4.12 (m, 4 H, OCH_2_), 4.28 (d, ^2^*J* = 12.4 Hz, 4 H, CH_2_Ar), 4.46 (s, 4 H, CH_2_C=O), 6.99 (s, 4 H, ArH), 7.15 (s, 4 H, ArH); ^13^C NMR (101 MHz, CDCl_3_): δ 30.0 (CH_2_Ar), 31.2 (CH_3_), 31.5 (CH_3_), 34.2 (C_q_), 34.3 (C_q_), 59.2 (OCH_3_), 68.7 (OCH_2_), 70.0 (OCH_2_), 70.6 (OCH_2_), 76.4 (OCH_2_), 76.9 (CH_2_C=O), 120.5 (q, ^1^*J*_C,F_ = 323 Hz, CF_3_), 125.97 (CH_Ar_), 126.02 (CH_Ar_), 134.3, 134.7, 147.6, 148.4, 148.5, 149.6 (6 × C_Ar_), 178.3 (C=O); ^19^F NMR (376 MHz, CDCl_3_): δ − 79.0 (CF_3_) ppm; MS (ESI+) *m*/*z* = 1232 (M^+^ + H), 1254 (M^+^ + Na).

**5**,**11**,**17**,**23-Tetrakis(tert-butyl)-26**,**28-bis[2-(2-methoxy ethoxy)ethoxy]-25**,**27-[2-(heptafluoroisopropylsulfonamido)-2-oxoethyl]calix[4]arene 7b.** Under Ar, compound 3 (100 mg, 0.10 mmol) was dissolved in CCl_4_ (5 mL), oxalyl chloride (0.75 g, 5.88 mmol) dropwise added and the reaction mixture stirred at 65 °C for 5 h. After cooling to rt, the remaining oxalyl chloride and the solvent were removed under high vacuum, anhydrous THF (3 mL) added and the solution cooled to 0 °C. In a second flask, heptafluoroisopropylsulfonamide (64 mg, 0.26 mmol) was dissolved in anhydrous THF (2 mL) and NaH (41 mg, 1.03 mmol, 60% in mineral oil) was added. After stirring for 15 min, this solution was added to the other THF solution at 0 °C and the combined mixture was stirred for 1.5 h at rt. The solvent was changed to chloroform (20 mL) and the organic phase washed with saturated hydrogen carbonate solution (15 mL), aqueous HCl (10%, 15 mL), and brine (15 mL). After separation, the organic phase was dried over Na_2_SO_4_ and the crude product was purified using automated column chromatography (petroleum ether:ethyl acetate = 0 → 20%) to obtain **7b** as colorless solid (53 mg, 36%); mp 113–115 °C; R*_f_* 0.76 (petroleum ether:ethyl acetate = 1:1); ^1^H NMR (400 MHz, CDCl_3_): δ 1.04 (s, 18 H, ^t^Bu), 1.21 (s, 18 H, ^t^Bu), 3.35 (d, ^2^*J* = 12.5 Hz, 4 H, CH_2_Ar), 3.41 (s, 6 H, OCH_3_), 3.58 (t, ^3^*J* = 4.4 Hz, 4 H, OCH_2_), 3.70–3.77 (m, 8 H, OCH_2_), 4.06–4.11 (m, 4 H, OCH_2_), 4.27 (d, ^2^*J* = 12.5 Hz, 4 H, CH_2_Ar), 4.44 (s, CH_2_C=O), 6.99 (s, 4 H, ArH), 7.16 (s, 4 H, ArH); ^13^C NMR (101 MHz, CDCl_3_): δ 29.9 (CH_2_Ar), 31.2 (CH_3_), 31.5 (CH_3_), 34.2 (C_q_), 34.4 (C_q_), 59.2 (OCH_3_), 68.8 (OCH_2_), 70.0 (OCH_2_), 70.6 (OCH_2_), 76.3 (OCH_2_), 76.9 (CH_2_C=O), 96.5 (dsep, ^1^*J*_C,F_ = 240 Hz, ^2^*J*_C,F_ = 32 Hz, CF), 119.6 (dq, ^1^*J*_C,F_ = 288 Hz, ^2^*J*_C,F_ = 26 Hz, CF_3_), 126.0 (CH_Ar_), 126.04 (CH_Ar_), 134.3, 134.7, 147.7, 148.5, 148.52, 149.5 (6 × C_Ar_), 178.0 (C=O); ^19^F NMR (565 MHz, CDCl_3_): δ –170.5 (sep, ^3^*J* = 7.0 Hz, 1 F, CF), −71.9 (d, ^3^*J* = 7.0 Hz, 6 F, CF_3_) ppm.

**5**,**11**,**17**,**23-Tetrakis(*tert*-butyl)-26**,**28-bis[2-(2-methoxyethoxy)ethoxy]-25**,**27-[2-(pentafluorophenylsulfonamido)-2-oxoethyl]calix[4]arene 7c.** Under Ar, compound 3 (100 mg, 0.10 mmol) was dissolved in CCl_4_ (5 mL), oxalyl chloride (0.75 g, 5.88 mmol) dropwise added and the reaction mixture stirred at 65 °C for 5 h. After cooling to rt, the remaining oxalyl chloride and the solvent were removed under high vacuum, anhydrous THF (3 mL) added and the solution cooled to 0 °C. In a second flask, pentafluorophenylsulfonamide (64 mg, 0.26 mmol) was dissolved in anhydrous THF (2 mL) and NaH (41 mg, 1.03 mmol, 60% in mineral oil) was added. After stirring for 15 min, this solution was added to the other THF-solution at 0 °C and the combined mixture was stirred for 1.5 h at rt. The solvent was changed to chloroform (20 mL) and the organic phase washed with saturated hydrogen carbonate solution (15 mL), aqueous HCl (10%, 15 mL), and brine (15 mL). After separation, the organic phase was dried over Na_2_SO_4_ and the crude product was purified using automated column chromatography (petroleum ether:ethyl acetate = 0 → 20%) to obtain **7c** as colorless solid (15 mg, 10%); mp 98–100 °C; R*_f_* 0.76 (petroleum ether:ethyl acetate = 1:1); ^1^H NMR (600 MHz, CDCl_3_): δ 1.06 (s, 18 H, ^t^Bu), 1.18 (s, 18 H, ^t^Bu), 3.31 (d, ^2^*J* = 12.4 Hz, 4 H, ArCH_2_), 3.39 (s, 6 H, OCH_3_), 3.51 (t, ^3^*J* = 4.6 Hz, 4 H, OCH_2_), 3.58 (t, ^3^*J* = 3.8 Hz, 4 H, OCH_2_), 3.67 (t, ^3^*J* = 4.6 Hz, 4 H, OCH_2_), 4.02 (t, ^3^*J* = 3.8 Hz, 4 H, OCH_2_), 4.25 (d, ^2^*J* = 12.4 Hz, 4 H, CH_2_Ar), 4.41 (s, 4 H, CH_2_C=O), 7.00 (s, 4 H, ArH), 7.12 (s, 4 H, ArH); ^13^C NMR (151 MHz, CDCl_3_): δ 30.0 (CH_2_Ar), 31.3 (CH_3_), 31.5 (CH_3_), 34.2 (C_q_), 34.3 (C_q_), 59.3 (OCH_3_), 68.3 (OCH_2_), 69.7 (OCH_2_), 70.4 (OCH_2_), 75.9 (OCH_2_), 76.9 (CH_2_C=O), 125.9 (CH_Ar_), 125.94 (CH_Ar_), 134.5, 134.6, 147.7, 148.3, 148.9, 149.5 (6 × C_q_), 177.3 (C=O); ^19^F NMR (564 MHz, CDCl_3_): δ –160.7 (t, ^3^*J* = 20.0 Hz, 4 F, m-ArF), –150.3 (t, ^3^*J* = 20.5 Hz, 2 F, p-ArF), −130.1 (d, ^3^*J* = 20.0 Hz, 4 F, o-ArF) ppm; MS (ESI+) *m*/*z* = 1428 (M^+^ + H), 1445 (M^+^ + NH_4_).

***tert*-Butyl 3**,**4-bis[2-(2-hydroxyethoxy)ethoxy]benzoate 11c.** Under Ar, compound **9c** (176 mg, 0.84 mmol), K_2_CO_3_ (578 mg, 4.19 mmol) and KI (194 mg, 4.2 mmol) were suspended in anhydrous DMF (5 mL) and 2-(2-chloroethoxy)ethanol (354 μL, 417 mg, 3.35 mmol) was added. The mixture was stirred at 75 °C overnight. After cooling to rt, the solvent was changed to DCM (15 mL) and filtered. The organic phase was washed with hydrochloric acid (10 mL, 10%), brine (10 mL) and water (10 mL). After removal of the solvent, the crude product was purified via column chromatography (ethyl acetate:methanol 99:1) to obtain **11c** as colorless oil (140 mg, 43%); R*_f_* = 0.4 (DCM:methanol = 9:1); ^1^H NMR (400 MHz, CDCl_3_): δ 1.58 (s, 9H, ^t^Bu). 2.79 (s, 2H, OH), 3.72–3.65 (m, 4H, OCH_2_), 3.79–3.71 (m, 4H, OCH_2_), 3.95–3.88 (m, 4H, OCH_2_), 4.24–4.17 (m, 4H, OCH_2_), 6.86 (d, ^3^*J* = 8.4 Hz, 1H, ArH), 7.53 (d, ^4^*J* = 1.9 Hz, 1H, ArH), 7.60 (dd, ^3^*J* = 8.4 Hz, ^4^*J* = 1.9 Hz, 1H, ArH); ^13^C NMR (101 MHz, CDCl_3_): δ 28.2 (^t^Bu), 61.6 (OCH_2_), 68.3 (OCH_2_), 68.4 (OCH_2_), 69.0 (OCH_2_), 69.2 (OCH_2_), 72.7 (OCH_2_), 72.8 (OCH_2_), 80.8 (C_q_), 111.7 (CH_Ar_), 113.9 (CH_Ar_), 123.6 (CH_Ar_), 125.0 (C_Ar_), 147.7 (C_Ar_), 151.9 (C_Ar_), 165.5 (C=O) ppm; MS (ESI+): *m*/*z* = 409 (M^+^ + Na).

**1**,**2-Bis[2-(2-hydroxyethoxy)ethoxy]-4-nitrobenzene 11d.** Under Ar, 4-nitrocatechol (1.0 g, 6.45 mmol), K_2_CO_3_ (8.0 g, 64 mmol) and KI (1.66 g, 9.03 mmol) were suspended in anhydrous DMF (20 mL) and 2-(2-chloroethoxy)ethanol (2.72 g, 29mmol) was added. The mixture was stirred at 75 °C overnight. After cooling to rt, the solvent was changed to DCM (35 mL) and filtered. The organic phase was washed with hydrochloric acid (25 mL, 10%), brine (25 mL) and water (25 mL). After removal of the solvent, the crude product was purified via column chromatography (DCM:methanol 97:3) to obtain **11d** as colorless oil (1.08 g, 52%); R*_f_* 0.4 (DCM:methanol 9:1); ^1^H NMR (600 MHz, CDCl_3_): δ 3.41 (s, 2H, OH), 3.63–3.59 (m, 4H, OCH_2_), 3.69–3.66 (m, 4H, OCH_2_), 3.90–3.84 (m, 4H, OCH_2_), 4.20–4.12 (m, 4H, OCH_2_), 6.85 (d, ^3^*J* = 8.9 Hz, 1H, ArH), 7.67 (d, ^4^*J* = 2.6 Hz, 1H, ArH), 7.80 (dd, ^3^*J* = 8.9 Hz, ^4^*J* = 2.6 Hz, 1H, ArH); ^13^C NMR (151 MHz, CDCl_3_): δ 61.4 (OCH_2_), 61.5 (OCH_2_), 68.6 (OCH_2_), 68.7 (OCH_2_), 68.8 (OCH_2_), 68.9 (OCH_2_), 72.7 (OCH_2_), 72.7 (OCH_2_), 108.0 (CH_Ar_), 111.3 (CH_Ar_), 118.0 (CH_Ar_), 141.4 (C_Ar_), 148.0 (C_Ar_), 153.9 (C_Ar_) ppm; MS (ESI+): *m*/*z* = 354 (M^+^ + Na).

***tert*-Butyl 3**,**4-bis{2-[2-(tosyloxy)ethoxy]ethoxy}benzoate 12c.** Under Ar, compound **11c** (459 mg, 1.2 mmol) and Et_3_N (536 μL, 392 mg, 3.87 mmol) were dissolved in anhydrous DCM (15 mL), cooled to 0 °C, *p*-TsCl (1.40 g, 9.9 mmol) added and the reaction mixture stirred at rt overnight. Afterwards, the organic phase was washed with water (2 × 15 mL), dried over Na_2_SO_4_. After removal of the solvent, the crude product was purified using automated column chromatography (DCM → DCM:methanol 99:1) to obtain **12c** as colorless oil (298 mg, 36%); R*_f_* 0.6 (DCM:methanol = 99:1); ^1^H NMR (400 MHz, CDCl_3_): δ 1.59 (s, 9H, ^t^Bu), 2.42–2.39 (m, 6H, CH_3_), 3.85–3.73 (m, 8H, OCH_2_), 4.15–4.05 (m, 4H, OCH_2_), 4.21–4.14 (m, 4H, OCH_2_), 6.84 (d, ^3^*J* = 8.4 Hz, 1H, ArH), 7.32–7.25 (m, 4H, Ar_Ts_), 7.48 (d, ^4^*J* = 2.0 Hz, 1H, ArH), 7.60 (dd, ^3^*J* = 8.4 Hz, ^4^*J* = 2.0 Hz, 1H, ArH), 7.78 (d, ^3^*J* = 8.0 Hz, 4H, Ar_Ts_); ^13^C NMR (151 MHz, CDCl_3_): δ 21.6 (CH_3_), 21.6 (CH_3_), 28.2 (^t^Bu), 68.6 (OCH_2_), 68.8 (OCH_2_), 68.9 (OCH_2_), 69.0 (OCH_2_), 69.3 (OCH_2_), 69.4 (OCH_2_), 69.6 (OCH_2_), 69.8 (OCH_2_), 80.8 (C_q_), 112.6 (CH_Ar_), 114.8 (CH_Ar_), 123.8 (CH_Ar_), 125.1 (C_Ar_), 128.0 (CH_Ts_), 129.8 (CH_Ts_), 133.0, 144.8, 148.1, 152.4 (4 × C_Ar_), 165.5 (C=O) ppm; MS (ESI+): *m*/*z* 718 (M^+^ + Na).

**1**,**2-Bis{2-[2-(tosyloxy)hydroxyethoxy]ethoxy}-4-nitrobenzene 12d.** Compound **11d** (1.37 g, 4,13 mmol) was dissolved in THF and cooled to 0 °C. NaOH (500 mg, 12.4 mmol) dissolved in water (2.5 mL) and *p*-tosyl chloride (1.97 g, 10.3 mmol) were added and the mixture was stirred 3 h at rt. Afterwards, saturated hydrogen carbonate solution (10 mL) was added and extracted with DCM (2 × 50 mL). The organic phase was washed with water (2 × 50 mL), dried over Na_2_SO_4_ and the crude product was purified using automated column chromatography (DCM → DCM:methanol 50:1) to obtain **12d** as yellowish oil (1.74 g, 66%); R*_f_* 0.6 (DCM:methanol = 99:1); ^1^H NMR (600 MHz, CDCl_3_): δ 2.42–2.40 (m, 6H, CH_3_), 3.79–3.76 (m, 4H, OCH_2_), 3.87–3.81 (m, 4H, OCH_2_), 4.15–4.11 (m, 2H, OCH_2_), 4.20–4.15 (m, 6H, OCH_2_), 6.91 (d, ^3^*J* = 8.9 Hz, 1H, ArH), 7.32–7.28 (m, 4H, Ts), 7.73 (d, ^4^*J* = 2.6 Hz, 1H, ArH), 7.80–7.75 (m, 4H, Ts), 7.88 (dd, ^3^*J* = 8.9 Hz, ^4^*J* = 2.6 Hz, 1H, ArH); ^13^C NMR (151 MHz, CDCl_3_): δ 21.7 (CH_3_), 21.8 (CH_3_), 69.1 (OCH_2_), 69.2 (OCH_2_), 69.2 (OCH_2_), 69.3 (OCH_2_), 69.4 (OCH_2_), 69.5 (OCH_2_), 69.6 (CH_2_), 69.7 (OCH_2_), 109.0 (CH_Ar_), 112.1 (CH_Ar_), 118.3 (CH_Ar_), 128.1 (CH_Ts_), 128.2 (CH_Ts_), 129.9 (CH_Ts_), 130.0 (CH_Ts_), 133.1, 141.7, 145.0, 145.1, 148.5, 154.5 (6 × C_Ar_) ppm; MS (ESI+): *m*/*z* = 662 (M^+^ + Na).

**5**,**11**,**17**,**23-Tetrakis(*tert*-butyl)-26**,**28-dihydroxycalix[4]arene-propylenecrown-6 14b.** Under Ar, *tert*-butylcalix[4]arene (**13**, 158 mg, 0.24 mmol) and K_2_CO_3_ (39 mg, 0.28 mmol) were suspended in DCM (10 mL) and compound **8b** (273 mg, 0.49 mmol) dissolved in 5 mL of DCM was added dropwise. The resulting mixture was stirred at 30 °C for 11 d. Afterwards, the DCM phase was washed with water (2 × 15 mL) and aqueous HCl (10%, 2 × 15 mL), dried over Na_2_SO_4_ and the solvent was removed. The crude product was purified via column chromatography (petroleum ether:ethyl acetate = 2:1 → 1:1) yielding compound **14b** as a colourless solid (85 mg, 40%); R*_f_* 0.2 (petroleum ether:ethyl acetate = 1:1); ^1^H NMR (600 MHz, CDCl_3_): δ 0.94 (s, 18H, ^t^Bu), 1.30 (s, 18H, ^t^Bu), 1.76 (p, ^3^*J* = 5.8 Hz, 2H, OCH_2_), 3.30 (d, ^2^*J* = 13.0 Hz, 4H, CH_2_Ar), 3.67 (t, ^3^*J* = 5.8 Hz, 4H, OCH_2_), 3.75–3.78 (m, 4H, OCH_2_), 3.90–3.93 (m, 4H, OCH_2_), 4.00–4.03 (m, 4H, OCH_2_), 4.13–4.16 (m, 4H, OCH_2_), 4.37 (d, ^2^*J* = 13.0 Hz, 4 H, CH_2_Ar), 6.78 (s, 4H ArH), 7.06 (s, 4H, ArH), 7.18 (s, 2H, OH); ^13^C NMR (101 MHz, CDCl_3_): δ 30.4 (OCH_2_), 31.1 (CH_3_), 31.6 (CH_2_Ar), 31.9 (CH_3_), 34.0 (C_q_), 34.1 (C_q_), 67.5 (OCH_2_), 71.0 (OCH_2_), 70.1 (OCH_2_), 71.3 (OCH_2_), 76.1 (OCH_2_), 125.1 (CH_Ar_), 125.6 (CH_Ar_), 128.0 (C_Ar_), 132.7 (C_Ar_), 141.4 (C_Ar_), 146.9 (C_Ar_), 150.0 (C_Ar_), 150.8 (C_Ar_) ppm; MS (ESI+): *m*/*z* = 883 (M^+^ + NH_4_), 888 (M^+^ + Na).

**5**,**11**,**17**,**23-Tetrakis(*tert*-butyl)-26**,**28-dihydroxycalix[4]arene-benzocrown-6 14c.** Under Ar, *tert*-butylcalix[4]arene (**13**, 610 mg, 0.94 mmol) and K_2_CO_3_ (150 mg, 1.08 mmol) were suspended in DCM (20 mL) and compound **12a** (1.12 g, 1.88 mmol) dissolved in 10 mL of DCM was added dropwise. The resulting mixture was stirred at 30 °C for 11 d. Afterwards, the DCM phase was washed with water (2 × 30 mL) and aqueous HCl (10%, 2 × 30 mL), dried over Na_2_SO_4_ and the solvent was removed. The crude product was purified via column chromatography (DCM:ethyl acetate = 20:1 → 10:1) yielding compound **14c** as colourless solid (346 mg, 41%); mp 154 °C; R*_f_* 0.2 (DCM:methanol = 96:4); ^1^H NMR (600 MHz, CDCl_3_): δ 0.93 (s, 18H, ^t^Bu), 1.29 (s, 18H, ^t^Bu), 3.29 (d, ^2^*J* = 13.1 Hz, 4H, CH_2_Ar), 4.11 (t, ^3^*J* = 4.7 Hz, 4H, OCH_2_), 4.15 (t, ^3^*J* = 4.7 Hz, 4H, OCH_2_), 4.23 (t, ^3^*J* = 4.7 Hz, 4H, OCH_2_), 4.27–4.33 (m, 8H, OCH_2_, CH_2_Ar), 6.77 (s, 4H, ArH), 6.91–6.94 (m, 2H, ArH), 6.97 (m, 2H, ArH), 7.05 (s, 4H, ArH), 7.18 (s, 2H, OH); ^13^C NMR (151 MHz, CDCl_3_): δ 31.1 (^t^Bu), 31.6 (CH_2_Ar), 31.9 (^t^Bu), 34.0 (C_q_), 34.1 (C_q_), 70.3 (OCH_2_), 70.5 (OCH_2_), 71.1 (OCH_2_), 76.5 (OCH_2_), 116.5 (CH_Ar_), 122.2 (CH_Ar_), 125.1 (CH_Ar_), 125.7 (CH_Ar_), 127.9, 132.6, 141.5, 147.1, 149.5, 149.9, 150.8 (7 × C_Ar_) ppm; MS (ESI+): *m*/*z* = 917 (M^+^ + NH_4_), 922 (M^+^ + Na).

**5**,**11**,**17**,**23-Tetrakis(*tert*-butyl)-26**,**28-dihydroxycalix[4]arene-benzocrown-8 14d.** Under Ar, *tert*-butylcalix[4]arene (**13**, 265 mg, 0.41 mmol) and K_2_CO_3_ (65 mg, 0.47 mmol) were suspended in DCM (20 mL) and compound **12b** (557 mg, 0.82 mmol) dissolved in 5 mL of DCM was added dropwise. The resulting mixture was stirred at 30 °C for 11 d. Afterwards, the DCM phase was washed with water (2 × 20 mL) and aqueous HCl (10%, 2 × 20 mL), dried over Na_2_SO_4_ and the solvent was removed. The crude product was purified via column chromatography (petroleum ether:acetone = 3:1) yielding compound **14d** as colourless solid (325 mg, 81%); R*_f_* 0.2 (petroleum ether:acetone = 3:1); ^1^H NMR (400 MHz, CDCl_3_): δ 0.95 (s, 18H, ^t^Bu), 1.29 (s, 18H, ^t^Bu), 3.28 (d, ^2^*J* = 13.0 Hz, 4H, CH_2_Ar), 3.91–3.96 (m, 8H, OCH_2_), 3.97–4.05 (m, 8H, OCH_2_), 4.10–4.16 (m, 8H, OCH_2_), 4.35 (d, ^2^*J* = 13.0 Hz, 4H, CH_2_Ar), 6.78 (s, 4H, ArH), 6.85–6.91 (m, 4H, ArH), 7.05 (s, 4H, ArH), 7.30 (s, 2H, OH) ppm; ^13^C NMR (101 MHz, CDCl_3_): δ 31.2 (CH_3_), 31.6 (CH_2_Ar), 31.9 (CH_3_), 34.0 (C_q_), 34.1 (C_q_), 69.6 (OCH_2_), 70.1 (OCH_2_), 70.4 (OCH_2_), 71.4 (OCH_2_), 71.5 (OCH_2_), 76.1 (OCH_2_), 114.2 (CH_Ar_), 121.5 (CH_Ar_), 125.1 (CH_Ar_), 125.6 (CH_Ar_), 127.9, 132.8, 141.4, 147.0, 149.2, 149.9, 150.9 (7 × C_Ar_) ppm; MS (ESI+): *m*/*z* = 1009 (M^+^ + Na).

**5**,**11**,**17**,**23-Tetrakis(*tert*-butyl)-26**,**28-dihydroxycalix[4]arene-4-*tert*-butoxycarbonyl-benzocrown-6 14e**. Under Ar, *tert*-butylcalix[4]arene (**13**, 140 mg, 0.22 mmol) and K_2_CO_3_ (34 mg, 0.25 mmol) were suspended in DCM (10 mL). Compound **12e** (298 mg, 0.43 mmol) dissolved in 5 mL of DCM was added dropwise. The resulting mixture was stirred at 30 °C for 11 d. Afterwards, the DCM phase was washed with water (2 × 20 mL) and aqueous HCl (10%, 2 × 20 mL), dried over Na_2_SO_4_ and the solvent was removed. The crude product was purified via column chromatography (petroleum ether:acetone = 4:1 → 1:1) yielding compound **14e** as colourless solid (78 mg, 36%); R*_f_* 0.5 (petroleum ether:acetone = 2:1); ^1^H NMR (400 MHz, CDCl_3_): δ 0.93 (s, 18H, ^t^Bu), 1.30 (s, 18H, ^t^Bu), 1.58 (s, 9H, O ^t^Bu), 3.30 (d, ^2^*J* = 12.7 Hz, 4H, CH_2_Ar), 4.10–4.33 (m, 20H, OCH_2_ + CH_2_Ar), 6.76 (s, 4H, ArH), 6.90 (d, ^3^*J* = 8.1 Hz, 1H, ArH), 7.05 (s, 4H, ArH), 7.14 (s, 2H, OH), 7.59–7.64 (m, 2H, ArH); ^13^C NMR (101 MHz, CDCl_3_): δ 28.4 (O^t^Bu), 31.6 (CH_2_Ar), 31.1 (CH_3_), 31.9 (CH_3_), 34.0 (C_q_), 34.0 (C_q_), 69.8 (OCH_2_), 70.3 (OCH_2_), 70.5 (OCH_2_), 70.6 (OCH_2_), 70.8 (OCH_2_), 76.4 (OCH_2_), 76.5 (OCH_2_), 80.8 (OC_q_), 114.0 (CH_Ar_), 116.9 (CH_Ar_), 124.4 (CH_Ar_), 125.1 (CH_Ar_), 125.2 (CH_Ar_), 125.7 (CH_Ar_), 126.1, 127.8, 127.9, 132.6, 141.8, 141.9, 147.0, 147.1, 148.5, 150.8, 153.8 (11 × C_Ar_), 165.7 C=O) ppm; MS (ESI+): *m*/*z* = 1022 (M^+^ + Na).

**5**,**11**,**17**,**23-Tetrakis(*tert*-butyl)-26**,**28-dihydroxycalix[4]arene-4-nitrobenzocrown-6 14f.** Under Ar, *tert*-butylcalix[4]arene (**13**, 882 mg, 1.36 mmol) and K_2_CO_3_ (220 mg, 1.56 mmol) were suspended in DCM (20 mL), and compound **12f** (1.74 g, 2.72 mmol) dissolved in 10 mL of DCM was added dropwise. The resulting mixture was stirred at 30 °C for 11 d. Afterwards, the DCM phase was washed with water (2 × 30 mL) and aqueous HCl (10%, 2 × 30 mL), dried over Na_2_SO_4_ and the solvent was removed. The crude product was purified via column chromatography (DCM:ethyl acetate = 97:3 → 9:1) yielding compound **14f** as colourless solid (870 mg, 68%); R*_f_* 0.3 (DCM:ethyl acetate = 9:1); ^1^H NMR (600 MHz, CDCl_3_): δ 0.91 (s, 18H, CH_3_), 1.29 (s, 18H, CH_3_), 3.25–3.30 (m, 4H, ArCH_2_), 4.08–4.15 (m, 8H, OCH_2_, ArCH_2_), 4.18–4.30 (m, 8H, OCH_2_), 4.34–4.40 (m, 4H, OCH_2_), 6.71–6.76 (m, 4H, ArH), 6.88–6.92 (m, 1H, ArH), 6.99 (s, 2H, OH), 7.02–7.04 (m, 4H, ArH), 7.77–7.83 (m, 2H, ArH); ^13^C NMR (151 MHz, CDCl_3_): δ 31.1 (^t^Bu), 31.5 (ArCH_2_), 31.9 (^t^Bu), 34.0 (C_q_), 34.1 (C_q_), 69.9 (OCH_2_), 70.3 (OCH_2_), 70.4 (OCH_2_), 70.5 (OCH_2_), 70.6 (OCH_2_), 70.8 (OCH_2_), 76.2 (OCH_2_), 76.3 (OCH_2_), 110.5 (CH_Ar_), 113.3 (CH_Ar_), 118.5 (CH_Ar_), 125.1 (CH_Ar_), 125.2 (CH_Ar_), 125.7 (CH_Ar_), 127.8, 127.9, 132.4, 132.5, 141.6, 141.9, 147.1, 149.8, 149.9, 150.7, 154.9 ppm (11 × C_Ar_); MS (ESI+): *m*/*z* = 967 (M^+^ + Na), 983 (M^+^ + K).

**5**,**11**,**17**,**23-Tetrakis(*tert*-butyl)-25**,**27-bis(2-ethoxy-2-oxoethoxy)calix[4]arene-propylenecrown-6 15b.** Under Ar, compound **14b** (85 mg, 0.12 mmol) was dissolved in anhydrous THF (10 mL) and NaH (23 mg, 0.58 mmol, 60% in mineral oil) was added and the mixture stirred for 30 min at rt. Next, ethyl bromoacetate (65 μL, 0.58 mmol) was added and the mixture stirred at 60 °C overnight. Afterwards, the solvent was changed to DCM (15 mL), the organic phase washed with water (2 × 15 mL) and dried over Na_2_SO_4_. After removal of the solvent, the crude product was purified via column chromatography (petroleum ether:ethyl acetate = 2:1 → 1:1) yielding compound **15b** as colourless solid (45 mg, 37%); R*_f_* 0.2 (petroleum ether:ethyl acetate = 2:1); ^1^H NMR (600 MHz, CDCl_3_): δ 0.88 (s, 18H, ^t^Bu), 1.26 (s, 18H, ^t^Bu), 1.30 (t, ^3^*J* = 7.1 Hz, 6H, CH_3_), 1.83 (p, ^3^*J* = 5.7 Hz, 2H, CH_2_), 3.16 (d, ^2^*J* = 12.7 Hz, 4H, CH_2_Ar), 3.67 (t, ^3^*J* = 5.7 Hz, 4H, OCH_2_), 3.70 (m, 4H, OCH_2_), 3.75–3.78 (m, 4H, OCH_2_), 4.11–4.15 (m, 4H, OCH_2_), 4.21–4.28 (m, 8H, CH_2_C=O, OCH_2_), 4.51 (d, ^2^*J* = 12.7 Hz, 4H, CH_2_Ar), 6.55 (s, 4H, ArH), 4.56 (s, 4H, OCH_2_), 7.02 (s, 4H, ArH); ^13^C NMR (151 MHz, CDCl_3_): δ 14.4 (CH_3_), 30.5 (CH_2_), 31.3 (CH_3_), 31.4 (CH_2_Ar), 31.8 (CH_3_), 33.8 (C_q_), 34.2 (C_q_), 60.8 (CH 3 CH 2 OCO), 67.1 (OCH_2_), 70.0 (OCH_2_), 70.1 (OCH_2_), 70.4 (OCH_2_), 72.1 (OCH_2_C=O), 72.7 (OCH_2_), 125.0 (CH_Ar_), 125.6 (CH_Ar_), 132.4, 135.1, 145.0, 145.2, 152.3, 154.3 (6 × C_Ar_), 169.9 (C=O) ppm; MS (ESI+): *m*/*z* = 1060 (M^+^ + Na).

**5**,**11**,**17**,**23-Tetrakis(*tert*-butyl)-25**,**27-bis(2-ethoxy-2-oxoethoxy)calix[4]arene-benzocrown-6 15c.** Under Ar, compound **14c** (346 mg, 0.39 mmol) was dissolved in anhydrous THF (20 mL) and NaH (77 mg, 1.93 mmol, 60% in mineral oil) was added and the mixture stirred for 30 min at rt. Next, ethyl bromoacetate (214 μL, 1.93 mmol) and NaI in catalytic amounts were added and the mixture stirred at 60 °C overnight. Afterwards, the solvent was changed to DCM (20 mL), the organic phase was washed with water (2 × 20 mL) and dried over Na_2_SO_4_. After removal of the solvent, the product was recrystallized from hot methanol. After filtration the product was washed with cold methanol yielding compound **15c** as colorless solid (238 mg, 58%); R*_f_* 0.2 (petroleum ether: ethyl acetate = 2:1); ^1^H NMR (600 MHz, CDCl_3_): δ 0.92 (s, 18H, CH_3_), 1.14 (t, ^3^*J* = 7.1 Hz, 6H, CH_3_), 1.23 (s, 18H, CH_3_), 3.17 (d, ^2^*J* = 12.7 Hz, 4H, ArCH_2_), 4.00–4.07 (m, 8H, OCH_2_, CH_2_), 4.19–4.28 (m, 12H, OCH_2_), 4.54 (d, ^2^*J* = 12.7 Hz, 4H, ArCH_2_), 4.61 (s, 4H, CH_2_C=O), 6.58 (s, 4H, ArH), 6.91 (s, 4H, ArH), 6.97 (s, 4H, ArH); ^13^C NMR (151 MHz, CDCl_3_): δ 28.1 (CH_3_), 31.2 (ArCH_2_), 31.3 (^t^Bu), 31.7 (^t^Bu), 33.8 (C_q_), 34.1 (C_q_), 69.4 (OCH_2_), 70.2 (OCH_2_), 70.9 (OCH_2_), 72.0, (CH_2_C=O), 77.4 (OCH_2_), 114.6 (CH_Ar_), 121.6 (CH_Ar_), 123.6 (CH_Ar_), 125.1 (CH_Ar_), 125.6 (CH_Ar_), 128.2, 132.6, 134.7, 145.1, 149.6, 149.6 (6 × C_Ar_), 170.0 (C=O) ppm; MS (ESI+): *m*/*z* = 1094 (M^+^ + Na).

**5**,**11**,**17**,**23-Tetrakis(*tert*-butyl)-25**,**27-bis(2-ethoxy-2-oxoethoxy)calix[4]arene-benzocrown-8 15d.** Under Ar, compound **14d** (325 mg, 0.33 mmol) was dissolved in anhydrous THF (20 mL) and NaH (184 mg, 1.7 mmol, 60% in mineral oil) was added and the mixture stirred for 30 min at rt. Next, ethyl bromoacetate (183 μL, 1.7 mmol) and NaI in catalytic amounts were added and the mixture stirred at 60 °C overnight. Afterwards, the solvent was changed to DCM (15 mL), the organic phase was washed with brine (2 × 15 mL) and dried over Na_2_SO_4_. After removal of the solvent, the crude product was purified via column chromatography (DCM:methanol = 95:5) yielding compound **15d** as colorless solid (170 mg, 45%); R*_f_* 0.2 (petroleum ether:ethyl acetate = 2:1); ^1^H NMR (600 MHz, CDCl_3_): δ 0.87 (s, 18H, CH_3_), 1.23–1.28 (m, 24H, CH_3_ + OCH_2_CH_3_), 3.15 (d, ^2^*J* = 12.7 Hz, 4H, ArCH_2_), 3.79–3.82 (m, 4H, OCH_2_), 3.87–3.90 (m, 4H, OCH_2_), 3.94 (t, ^3^*J* = 4.5 Hz, 4H, OCH_2_CH_3_), 4.10–4.33 (m, 16H, OCH_2_), 4.48 (d, ^2^*J* = 12.7 Hz, 4H, ArCH_2_), 4.52 (s, 4H, CH_2_C=O), 6.53 (s, 4H, ArH), 6.88–6.92 (m, 4H, ArH), 7.02 (m, 4H, ArH); ^13^C NMR (151 MHz, CDCl_3_): δ 14.4 (CH_3_), 31.3 (^t^Bu), 31.6 (ArCH_2_), 31.8 (^t^Bu), 60.9 (CH_2_), 69.6 (OCH_2_), 69.9 (OCH_2_), 70.0 (OCH_2_), 70.8 (OCH_2_), 71.2 (OCH_2_), 72.2 (CH_2_C=O), 72.5 (OCH_2_), 77.4 (OCH_2_), 114.3 (CH_Ar_), 121.6 (CH_Ar_), 125.1 (CH_Ar_), 125.6 (CH_Ar_), 132.3, 135.1, 145.0, 145.2, 149.2, 152.3, 154.4 (7 × C_Ar_), 169.9 (C=O) ppm; MS (ESI+): *m*/*z* = 1182 (M^+^ + Na).

**5**,**11**,**17**,**23-Tetrakis(*tert*-butyl)-25**,**27-bis(2-ethoxy-2-oxoethoxy)calix[4]arene-4-*tert*-butoxycarbonyl-benzocrown-6 15e.** Under Ar, compound **14e** (78 mg, 0.078 mmol) was dissolved in anhydrous THF (10 mL) and NaH (16 mg, 0.39 mmol, 60% in mineral oil) was added and the mixture stirred for 30 min at rt. Next, ethyl bromoacetate (44 μL, 0.39 mmol) and NaI in catalytic amounts were added and the mixture stirred at 60 °C overnight. Afterwards, the solvent was changed to DCM (15 mL), the organic phase was washed with brine (2 × 15 mL) and dried over Na_2_SO_4_. After removal of the solvent, the crude product was purified via column chromatography (petroleum ether:ethyl acetate = 4:1) yielding compound **15e** as colorless solid (31 mg, 34%); R*_f_* 0.2 (petroleum ether:ethyl acetate = 2:1); ^1^H NMR (400 MHz, CDCl_3_): δ 0.93 (s, 18H, CH_3_), 1.12 (t, ^3^*J* = 7.2 Hz, 6H, CH_2_CH_3_), 1.21 (s, 18H, CH_3_), 1.58 (s, 9H, O^t^Bu), 3.17 (d, ^2^*J* = 12.8 Hz, 4H, ArCH_2_), 3.97–4.07 (m, 8H, OCH_2_), 4.20–4.29 (m, 12H, OCH_2_), 4.50–4.58 (m, 4H, ArCH_2_), 4.62 (s, 4H, CH_2_C=O), 6.59 (s, 4H, ArH), 6.86 (d, ^3^*J* = 8.5 Hz, 1H, ArH), 6.95 (s, 4H, ArH), 7.52 (s, 1H, ArH), 7.60 (d, ^3^*J* = 8.5 Hz, 1H, ArH); ^13^C NMR (101 MHz, CDCl_3_): δ 14.2 (CH_2_CH_3_), 28.4 (O^t^Bu), 31.4 (^t^Bu), 31.5 (ArCH_2_), 31.7 (^t^Bu), 33.9 (C_q_), 34.1 (C_q_), 60.7 (CH_2_CH_3_), 69.2 (OCH_2_), 69.6 (OCH_2_), 70.0 (OCH_2_), 70.1 (OCH_2_), 71.1 (OCH_2_), 71.2 (OCH_2_), 71.9 (CH_2_C=O), 73.5 (OCH_2_), 80.8 (COOC(CH_3_)_3_), 112.4 (CH_Ar_), 115.0 (CH_Ar_), 123.8 (CH_Ar_), 124.9 (C_Ar_), 125.1 (CH_Ar_), 125.1 (CH_Ar_), 125.6 (CH_Ar_), 132.7, 134.6, 134.6, 145.1, 148.6, 152.2, 153.1, 154.5, 154.6 (9 × C_Ar_), 165.7 (C=O), 170.0 (C=O) ppm; MS (ESI+): *m*/*z* = 1195 (M^+^ + Na).

**5**,**11**,**17**,**23-Tetrakis(*tert*-butyl)-25**,**27-bis(2-ethoxy-2-oxoethoxy)calix[4]arene-4-nitrobenzocrown-6 15f.** Under Ar, compound **14f** (870 mg, 0.92 mmol) was dissolved in anhydrous THF (40 mL) and NaH (184 mg, 4.61 mmol, 60% in mineral oil) was added and the mixture stirred for 30 min at rt. Next, ethyl bromoacetate (513 μL, 1.93 mmol) and NaI in catalytic amounts were added and the mixture stirred at 60 °C overnight. Afterwards, the solvent was changed to DCM (30 mL), the organic phase was washed with brine (2 × 30 mL) and dried over Na_2_SO_4_. After removal of the solvent, the crude product was purified via column chromatography (DCM → DCM:methanol = 98:2) yielding compound **15f** as colorless solid (960 mg, 93%); R*_f_* 0.5 (DCM:methanol = 95:5); ^1^H NMR (600 MHz, CDCl_3_): δ 0.95 (s, 18H, CH_3_), 1.11 (t, ^3^*J* = 7.1 Hz, 6H, CH_2_CH_3_), 1.19 (br. s, 18H, CH_3_), 3.17 (d, ^2^*J* = 12.8 Hz, 4H, ArCH_2_), 3.98–4.03 (m, 4H, CH_2_CH_3_), 4.04–4.09 (m, 4H, OCH_2_), 4.20–4.30 (m, 12H, OCH_2_), 4.50–4.58 (m, 4H, ArCH_2_), 4.65 (s, 4H, OCH_2_), 6.62 (s, 4H, ArH), 6.91–6.94 (m, 1H, ArH), 6.93 (d, ^3^*J* = 8.9 Hz, 4H, ArH), 7.75 (d, ^4^*J* = 2.7 Hz, 1H, ArH), 7.90 (dd, ^3^*J* = 8.9 Hz, ^4^*J* = 2.7 Hz, 1H, ArH); ^13^C NMR (151 MHz, CDCl_3_): δ 14.2 (CH_2_CH_3_). 31.4 (^t^Bu), 31.7 (ArCH_2_), 31.8 (^t^Bu), 33.9 (C_q_), 34.2 (C_q_), 60.7 (CH_2_CH_3_), 69.8 (OCH_2_), 69.9 (OCH_2_), 70.0 (OCH_2_), 71.3 (OCH_2_), 71.4 (OCH_2_), 72.0 (OCH_2_CO), 73.4 (OCH_2_), 73.5 (OCH_2_), 109.2 (CH_Ar_), 112.1 (CH_Ar_), 118.1 (CH_Ar_), 125.1 (CH_Ar_), 125.2 (CH_Ar_), 125.7 (CH_Ar_), 132.8, 134.5, 134.6, 141.9, 145.2, 145.3, 152.4, 154.6, 154.7, 155.1 (10 × C_Ar_), 169.9 (C=O) ppm; MS (ESI+): *m*/*z* = 1139 (M^+^ + Na).

**5**,**11**,**17**,**23-Tetrakis(*tert*-butyl)-25**,**27-bis(carboxymethoxy)calix[4]arene-propylenecrown-6 16b.** Compound **15b** (45 mg, 43 μmol) was dissolved in THF (5 mL), a solution of Me_4_NOH ∙ 5 H_2_O (55 mg, 0.30 mmol) in methanol (2 mL) was added and the reaction mixture was stirred at 55 °C overnight. The major part of the solvent was removed and the product was precipitated with ice-cold aqueous HCl (6 M). The solid residue was filtered and washed with water (20 mL) and dissolved in DCM (10 mL). The organic phase was washed with cold water (5 mL). After separation, the organic phase was dried over Na_2_SO_4_, the solvent was removed and **16b** was obtained as grey solid (41 mg, 95%); ^1^H NMR (600 MHz, CDCl_3_): δ 0.83 (s, 18H, ^t^Bu), 1.33 (s, 18H, ^t^Bu), 1.67 (p, ^3^*J* = 6.7 Hz, 2H, CH_2_), 3.21 (d, ^2^*J* = 13.0 Hz, 4H, CH_2_Ar), 3.58 (t, ^3^*J* = 6.7 Hz, 4H, OCH_2_), 3.65–3.68 (m, 4H, OCH_2_), 3.72–3.75 (m, 4H, OCH_2_), 3.83–3.86 (m, 4H, OCH_2_), 3.96–4.01 (m, 4H, OCH_2_), 4.50 (d, ^2^*J* = 13.0 Hz, 4H, CH_2_), 4.86 (s, 4H, CH_2_C=O), 6.59 (s, 4H, ArH), 7.14 (s, 4H, ArH); ^13^C NMR (151 MHz, CDCl_3_): δ = 29.8 (CH_2_); 31.0 (CH_2_Ar), 31.1 (CH_3_), 31.8 (CH_3_), 33.9 (C_q_), 34.3 (C_q_), 67.3 (OCH_2_), 70.3 (OCH_2_), 70.6 (OCH_2_), 70.7 (OCH_2_), 72.9 (CH_2_C=O), 77.1 (OCH_2_), 125.5 (CH_Ar_), 126.1 (CH_Ar_), 132.6, 135.2, 146.0, 146.9, 150.6, 153.4 (6 × C_Ar_), 171.0 (C=O) ppm; MS (ESI+): *m*/*z* = 1048 (M^+^ − 2 H + 3 Na).

**5**,**11**,**17**,**23-Tetrakis(*tert*-butyl)-25**,**27-bis(carboxymethoxy)calix[4]arene-benzocrown-6 16c.** Compound **15c** (446 mg, 0.435 mmol) was dissolved in THF (20 mL), a solution of Me_4_NOH ∙ 5 H_2_O (2.45 g, 6.85 mmol) in methanol (5 mL) was added and the reaction mixture was stirred at 55 °C overnight. After filtration, the major part of the solvent was removed and the product was precipitated with ice-cold aqueous HCl (6 M). The solid residue was filtered and washed with water (20 mL) and dissolved in DCM (30 mL). The organic phase was washed with cold water (15 mL). After separation, the organic phase was dried over Na_2_SO_4_, the solvent was removed and **16c** was obtained as brownish solid (440 mg, >99%); ^1^H NMR (400 MHz, CDCl_3_): δ 0.83 (s, 18H, CH_3_), 1.31 (s, 18H, CH_3_), 3.19 (d, ^2^*J* = 12.8 Hz, 4H, ArCH_2_), 3.80–4.28 (m, 16H, OCH_2_), 4.45 (d, ^2^*J* = 12.8 Hz, 4H, ArCH_2_), 4.86 (s, 4H, OCH_2_CO), 6.60 (s, 4H, ArH), 6.75–6.88 (m, 4H, ArH), 7.12 (s, 4H, ArH); ^13^C NMR (101 MHz, CDCl_3_): δ 30.9 (ArCH_2_), 31.1 (^t^Bu), 31.8 (^t^Bu), 33.9 (C_q_), 34.3 (C_q_), 67.9 (OCH_2_), 70.7 (OCH_2_), 71.0 (OCH_2_), 72.9 (OCH_2_C=O), 77.4 (OCH_2_), 111.5 (CH_Ar_), 120.6 (CH_Ar_), 125.5 (CH_Ar_), 126.0 (CH_Ar_), 132.6, 135.2, 145.9, 146.9, 148.4, 150.5 (6 × C_Ar_), 170.1 (C=O) ppm; MS (ESI+): *m*/*z* = 1060 (M^+^ − H + 2 Na), 1082 (M^+^ − 2 H + 3 Na).

**5**,**11**,**17**,**23-Tetrakis(*tert*-butyl)-25**,**27-bis(carboxymethoxy)calix[4]arene-benzocrown-8 16d.** Compound **15d** (170 mg, 0.15 mmol) was dissolved in THF (10 mL), a solution of Me_4_NOH ∙ 5 H_2_O (186 mg, 1.03 mmol) in methanol (2 mL) was added and the reaction mixture was stirred at 55 °C overnight. After filtration, the major part of the solvent was removed and the product was precipitated with ice-cold aqueous HCl (6 M). The solid residue was filtered and washed with water (10 mL) and dissolved in DCM (30 mL). The organic phase was washed with cold water (15 mL). After separation, the organic phase was dried over Na_2_SO_4_, the solvent was removed and **16d** was obtained as brownish solid (134 mg, 83%); ^1^H NMR (400 MHz, CDCl_3_): δ 0.83 (s, 18H, CH_3_), 1.33 (s, 18H, CH_3_), 3.17 (d, ^2^*J* = 13.0 Hz, 4H, ArCH_2_), 3.72–3.75 (m, 4H, OCH_2_), 3.78–3.84 (m, 8H, OCH_2_), 3.86–3.90 (m, 4H, OCH_2_), 3.95–4.04 (m, 8H, OCH_2_), 4.46 (d, ^2^*J* = 13.0 Hz, 4H, ArCH_2_), 4.74 (s, 4H, OCH_2_CO), 6.56 (s, 4H, ArH), 6.71–6.76 (m, 2H, ArH), 6.81–6.85 (m, 2H, ArH), 7.12 (s, 4H, ArH); ^13^C NMR (101 MHz, CDCl_3_): δ 30.9 (ArCH_2_), 31.1 (^t^Bu), 31.8 (^t^Bu), 33.9 (C_q_), 34.3 (C_q_), 69.1 (OCH_2_), 70.1 (OCH_2_), 70.5 (OCH_2_), 71.1 (OCH_2_), 72.4 (OCH_2_CO), 77.4 (OCH_2_), 115.2 (CH_Ar_), 121.6 (CH_Ar_), 125.5 (CH_Ar_), 126.1 (CH_Ar_), 132.5, 135.2, 147.1, 149.1, 150.4, 153.2 (6 × C_Ar_), 170.6 (C=O) ppm; MS (ESI+): *m*/*z* = 1170 (M^+^ − 2 H + 3 Na).

**5**,**11**,**17**,**23-Tetrakis(*tert*-butyl)-25**,**27-bis(carboxymethoxy)calix[4]arene-4-*tert*-butylcarboxy-benzocrown-6 16e.** Compound **15e** (31 mg, 27 μmol) was dissolved in THF (4 mL), a solution of Me_4_NOH ∙ 5 H_2_O (34 mg, 0.19 mmol) in methanol (2 mL) was added and the reaction mixture was stirred at 55 °C overnight. The solvent was removed and the crude product was dissolved in DCM (30 mL). The organic phase was washed with saturated NH_4_Cl solution (15 mL) and water (15 mL). After separation, the organic phase was dried over Na_2_SO_4_, the solvent was removed and **16e** was obtained as yellowish oil (26 mg, 87%); ^1^H NMR (400 MHz, CDCl_3_): δ 0.83 (s, 18H, CH_3_), 1.31 (s, 18H, CH_3_), 1.56 (s, 9H, OC(CH_3_)_3_), 3.19 (d, ^2^*J* = 12.8 Hz, 4H, ArCH_2_), 3.83–4.08 (m, 12H, OCH_2_), 4.26–4.31 (m, 4H, OCH_2_), 4.44 (d, ^3^*J* = 12.8 Hz, 4H, ArCH_2_), 4.84 (s, 4H, OCH_2_CO), 6.60 (s, 4H, ArH), 6.76 (d, ^3^*J* = 8.5 Hz, 1H, ArH), 6.98 (s, 4H, ArH), 7.40 (d, ^4^*J* = 1.9 Hz, 1H, ArH), 7.55 (dd, ^3^*J* = 8.5 Hz, ^4^*J* = 1.9 Hz, 1H, ArH); ^13^C NMR (101 MHz, CDCl_3_): δ 28.4 (O^t^Bu), 31.1 (^t^Bu), 31.2 (ArCH_2_), 31.8 (^t^Bu), 33.9 (C_q_), 34.3 (C_q_), 68.2 (OCH_2_), 68.5 (OCH_2_), 70.4 (OCH_2_), 70.5 (OCH_2_), 70.9 (OCH_2_), 71.0 (OCH_2_), 72.0 (OCH_2_CO), 77.4 (OCH_2_), 80.6 (OC_q_), 110.6 (CH_Ar_), 112.2 (CH_Ar_), 123.2 (CH_Ar_), 125.3 (C_Ar_), 125.5 (CH_Ar_), 125.7 (CH_Ar_), 135.2, 136.0, 143.3, 145.8, 145.9, 147.0, 147.9, 150.4, 150.5, 151.7, 152.1 (11 × C_Ar_), 165.9 (COO^t^Bu), 172.8 (C=O) ppm; MS (ESI+): *m*/*z* = 1160 (M^+^ − H + 2 Na).

**Disodium 5**,**11**,**17**,**23-tetrakis(*tert*-butyl)-25**,**27-bis(*N*-heptafluoropropane-2-sulfonylcarbamoyl-methoxy)calix[4]arene-crown-6 21.** Calixarene **16a** (300 mg, 0.31 mmol) was suspended in CCl_4_ (6 mL), oxalyl chloride (1.5 mL) was added and the mixture was stirred for 5 h at 65 °C. After cooling to rt, the solvent and remaining oxalyl chloride was removed. Under argon, the residue was dissolved in anhydrous DCM (5 mL) and a mixture of perfluoroisopropanesulfonamide (193 mg, 0.77 mmol) and NaH (60% in mineral oil, 124 mg, 3.1 mmol) dissolved in anhydrous DCM was added. After stirring overnight at rt, the mixture was filtered, the organic phase was washed with 10% HCl, the organic phase was separated and dried over Na_2_SO_4_. Next, the solvent was removed and the crude product was purified by column chromatography (DCM → DCM/methanol 5:1) to yield calixarene **21** as colorless solid (254 mg, 57%). R*_f_* 0.6 (petroleum ether:ethyl acetate = 2:1); ^1^H NMR (600 MHz, CDCl_3_): δ 1.11 (s, 18H, ^t^Bu), 1.15 (s, 18H, ^t^Bu), 3.38 (d, ^2^*J* = 12.3 Hz, 4H, ArCH_2_), 3.71–3.74 (m, 4H, OCH_2_), 3.82–3.86 (m, 4H, OCH_2_), 3.86–3.92 (s, 8H, OCH_2_), 4.10–4.18 (m, 8H, OCH_2_+ArCH_2_), 4.63 (s, 4H, CH_2_C=O), 7.08 (s, 4H, *m*-H_Ar_), 7.11 (s, 4H, *m*-H_Ar_); ^13^C NMR (151 MHz, CDCl_3_): δ 30.1 (ArCH_2_), 31.3, 31.4 (2 x CH_3_), 34.1, 34.3 (C_q_), 69.3, 69,4, 72.1, 72.2, (4 × OCH_2_), 77.0 (CH_2_C=O), 78.8 (OCH_2_), 125.8, 126.3 (2 × *m*-C_Ar_), 134.1, 134.4 (2 × *o*-C_Ar_), 147.8, 148.4 (2 × *p*-C_Ar_), 148.3, 151.5 (2 × *i*-C_Ar_), 176.2 (C=O); ^19^F NMR (565 MHz, CDCl_3_): δ −169.3 (2F), −71.7 (12F) ppm; MS (MALDI-TOF): *m*/*z* = 1429 (M^+^).

**Disodium 5**,**11**,**17**,**23-tetrakis(*tert*-butyl)-25**,**27-bis(*N*-heptafluoropropane-2-sulfonylcarbamoyl-methoxy)calix[4]arene-propylenecrown-6 22.** Under Ar, compound **16b** (41 mg, 42 μmol), 6-chloro-1-hydroxybenzotriazole (16 mg, 92 μmol), EDC · HCl (18 mg, 92 μmol), and Et_3_N (13 μL, 9.3 mg, 92 μmol) was dissolved in anhydrous acetonitrile (8 mL) and stirred at 0 °C for 30 min. Afterwards, heptafluoroisopropane-2-sulfonamide (42 mg, 167 μmol) and Et_3_N (12 μL, 8.4 mg, 84 μmol) dissolved in anhydrous acetonitrile (5 mL) were added and the resulting mixture stirred at rt overnight. Next, the solvent was changed to DCM (15 mL) and the organic phase was washed with brine (15 mL), saturated hydrogen carbonate solution (15 mL) and brine (15 mL), dried over Na_2_SO_4_ and the solvent was removed. The crude product was purified using column chromatography (petroleum ether:ethyl acetate = 4:1 → 2:1) to obtain **22** as colourless solid (24 mg, 40%); mp >350 °C; R*_f_* = 0.6 (petroleum ether:ethyl acetate = 2:1); ^1^H NMR (400 MHz, CDCl_3_): δ 1.07 (s, 18H, ^t^Bu), 1.18 (s, 18H, ^t^Bu), 2.09–2.01 (m, 2H, CH_2_), 3.36 (d, ^2^*J* = 12.3 Hz, 4H, CH_2_Ar), 3.74–3.69 (m, 4H, OCH_2_), 3.82–3.74 (m, 8H, 2 × OCH_2_), 3.89–3.82 (m, 4H, OCH_2_), 4.07–4.00 (m, 4H, OCH_2_), 4.16 (d, ^2^*J* = 12.3 Hz, 4H, CH_2_Ar), 4.58 (s, 4H, CH_2_C=O), 7.04 (s, 4H, ArH), 7.13 (s, 4H, ArH); ^13^C NMR (101 MHz, CDCl_3_): δ 30.0 (CH_2_Ar), 30.3 (CH_2_), 31.3 (CH_3_), 31.5 (CH_3_), 34.2 (C_q_), 34.3 (C_q_), 70.4 (OCH_2_), 71.1 (OCH_2_), 71.4 (OCH_2_), 72.5 (OCH_2_), 77.4 (CH_2_C=O), 78.0 (OCH_2_), 125.9 (CH_Ar_), 126.5 (CH_Ar_), 134.1, 134.5, 147.7, 148.4, 148.8, 151.0 (6 × C_Ar_), 175.9 (C=O); ^19^F NMR (376 MHz, CDCl_3_): δ −169.5 (hept, ^3^*J* = 7.5 Hz, 2F), −71.7 (d, ^3^*J* = 7.5 Hz, 12F) ppm; MS (ESI+): *m*/*z* = 1466 (M^+^ + Na).

**Disodium 5**,**11**,**17**,**23-tetrakis(*tert*-butyl)-25**,**27-bis(*N*-trifluormethanesulfonylcarbamoylmethoxy)-calix[4]arene-benzocrown-6 23.** Under Ar, compound **16c** (100 mg, 99 μmol), 6-chloro-1-hydroxybenzotriazole (37 mg, 0.22 mmol), EDC · HCl (42 mg, 0.22 mmol), and Et_3_N (30 μL, 22 mg, 0.22 mmol) were dissolved in anhydrous acetonitrile (10 mL) and stirred at 0 °C for 30 min. Afterwards, trifluoromethanesulfonamide (59 mg, 0.39 mmol) and Et_3_N (27 μL, 20 mg, 0.20 mmol) dissolved in anhydrous acetonitrile (5 mL) were added and the resulting mixture stirred at rt overnight. Next, the solvent was changed to DCM (15 mL) and the organic phase was washed with water (15 mL), saturated hydrogen carbonate solution (15 mL) and water (15 mL), dried over Na_2_SO_4_ and the solvent was removed. The crude product was purified using column chromatography (petroleum ether:ethyl acetate = 2:1 → 3:2) to obtain **23** as yellowish solid (122 mg, 97%); mp 320 °C (decomp.); R*_f_* 0.6 (petroleum ether:ethyl acetate = 1:1); ^1^H NMR (400 MHz, CDCl_3_): δ 1.13 (s, 18H, ^t^Bu), 1.15 (s, 18H, ^t^Bu), 1.10–1.16 (m, 36H, CH_3_), 3.40 (d, ^2^ *J* = 12.3 Hz, 4H, CH_2_Ar), 3.96–4.02 (m, 4H, OCH_2_), 4.04–4.21 (m, 12H, OCH_2_, CH_2_Ar), 4.27–4.33 (m, 4H, OCH_2_), 4.64 (s, 4H, CH_2_C=O), 6.88–6.98 (m, 4H, ArH), 7.07–7.14 (m, 8H, ArH) ppm; ^13^C NMR (101 MHz, CDCl_3_): δ 30.3 (CH_2_Ar), 31.3 (CH_3_), 31.4 (CH_3_), 34.2 (C_q_), 34.3 (C_q_), 67.0 (OCH_2_), 70.6 (OCH_2_), 72.6 (OCH_2_), 77.4 (CH_2_C=O), 78.3 (OCH_2_), 111.7 (CH_Ar_), 121.6 (CH_Ar_), 125.8 (CH_Ar_), 126.3 (CH_Ar_), 134.2, 134.3, 147.0, 148.0, 148.1, 148.4, 151.6 (7 × C_Ar_), 176.3 (C=O); ^19^F NMR (376 MHz, CDCl_3_): δ − 76.6 (CF_3_) ppm; MS (ESI+): *m*/*z* = 1278 (M^+^ + H), 1295 (M^+^ + NH_4_), 1295 (M^+^ + Na).

**Disodium 5**,**11**,**17**,**23-tetrakis(*tert*-butyl)-25**,**27-bis(*N*-heptafluoropropane-2-sulfonylcarbamoyl-methoxy)calix[4]arene-benzocrown-6 24.** Under Ar, compound **16c** (67 mg, 66 μmol), 6-chloro-1-hydroxybenzotriazole (20 mg, 0.15 mmol), EDC · HCl (29 mg, 0.15 mmol), and Et_3_N (21 μL, 15 mg, 0.15 mmol) were dissolved in anhydrous acetonitrile (10 mL) and stirred at 0 °C for 30 min. Afterwards, heptafluoroisopropane-2-sulfonamide (66 mg, 0.26 mmol) and Et_3_N (18 μL, 13 mg, 0.13 mmol) dissolved in anhydrous acetonitrile (5 mL) were added and the resulting mixture stirred at rt overnight. Next, the solvent was changed to DCM (15 mL) and the organic phase was washed with water (15 mL), saturated hydrogen carbonate solution (15 mL) and water (15 mL), dried over Na_2_SO_4_ and the solvent was removed. The crude product was purified using column chromatography (DCM → DCM:methanol = 74:1) to obtain **24** as colorless solid (50 mg, 50%); mp 340 °C (decomp.); R*_f_* 0.7 (DCM:methanol = 98:2); ^1^H NMR (400 MHz, CDCl_3_): δ 1.10 (s, 18H, ^t^Bu), 1.17 (s, 18H, ^t^Bu), 3.41 (d, ^2^*J* = 12.4 Hz, 4H, CH_2_Ar), 3.98–4.06 (m, 8H, OCH_2_), 4.17 (d, ^2^*J* = 12.4 Hz, 4H, CH_2_Ar), 4.20–4.25 (m, 8H, OCH_2_), 4.65 (s, 4H, CH_2_C=O), 6.84–6.88 (m, 2H, ArH), 6.93–6.97 (m, 2H, ArH), 7.06 (s, 4H, ArH_Calix_), 7.14 (s, 4H, ArH_Calix_); ^13^C NMR (101 MHz, CDCl_3_): δ 30.3 (CH_2_Ar), 31.4 (CH_3_), 31.4 (CH_3_), 34.2 (C_q_), 34.3 (C_q_), 66.5 (OCH_2_), 70.2 (OCH_2_), 72.8 (CH_2_C=O), 76.8 (OCH_2_), 78.1 (OCH_2_), 110.9 (CH_Ar_), 119.0 (dd, ^1^*J*_C-F_ = 288 Hz, ^3^*J*_C-F_ = 26 Hz, CF), 119.5 (qd, ^1^*J*_C-F_ = 288 Hz, ^3^*J*_C-F_ = 26 Hz, CF_3_), 121.4 (CH_Ar_), 125.8 (CH_Ar_), 126.3 (CH_Ar_), 134.1, 134.2, 146.8, 147.9, 148.0, 148.5, 151.9 (7 × C_Ar_), 176.1 (C=O); ^19^F NMR (564 MHz, CDCl_3_): δ − 72.0 (d, ^3^*J* = 7.0 Hz), −169.9 (hept, ^3^*J* = 7.0 Hz) ppm; MS (MALDI-TOF): *m*/*z* = 1499 (M^+^ + Na).

**Disodium 5**,**11**,**17**,**23-tetrakis(*tert*-butyl)-25**,**27-bis(*N*-pentafluorobenzenesulfonylcarbamoylmethoxy)-calix[4]arene-benzocrown-6 25**. Under Ar, compound **16c** (100 mg, 99 μmol), 6-chloro-1-hydroxybenzotriazole (37 mg, 0.22 mmol), EDC · HCl (42 mg, 0.22 mmol), and Et_3_N (30 μL, 22 mg, 0.22 mmol) were dissolved in anhydrous acetonitrile (10 mL) and stirred at 0 °C for 30 min. Afterwards, pentafluorobenzenesulfonamide (97 mg, 0.39 mmol) and Et_3_N (27 μL, 20 mg, 0.20 mmol) dissolved in anhydrous acetonitrile (5 mL) were added and the resulting mixture stirred at rt overnight. Next, the solvent was changed to DCM (15 mL) and the organic phase was washed with water (15 mL), saturated hydrogen carbonate solution (15 mL) and water (15 mL), dried over Na_2_SO_4_ and the solvent was removed. The crude product was purified using column chromatography (petroleum ether:ethyl acetate = 2:1 → 3:2), dissolved again in DCM and washed with 10% HCl to obtain **25** as yellowish solid (122 mg, 97%); mp 320 °C (decomp.); R*_f_* 0.6 (petroleum ether:ethyl acetate = 1:1); ^1^H NMR (400 MHz, CDCl_3_): δ 0.83 (s, 18H, CH_3_), 1.24 (s, 18H, CH_3_), 3.14 (d, ^2^*J* = 12.8 Hz, 4H, ArCH_2_), 4.00–4.10 (m, 8H, OCH_2_), 4.17–4.23 (m, 4H, OCH_2_), 4.32 (d, ^2^*J* = 12.8 Hz, 4H, ArCH_2_), 4.41–4.47 (m, 4H, OCH_2_), 5.17 (s, 4H, OCH_2_CO), 6.47 (s, 4H, ArH), 6.93 (s, 4H, ArH), 6.96–7.02 (m, 4H, ArH), 11.12 (s, 2H, C=O); ^13^C NMR (101 MHz, CDCl_3_): δ 31.1 (CH_3_), 31.6 (CH_3_), 32.1 (ArCH_2_), 33.8 (C_q_), 34.2 (C_q_), 68.1 (OCH_2_), 70.0 (OCH_2_), 70.3 (OCH_2_C=O), 71.7 (OCH_2_), 73.4 (OCH_2_), 114.0 (CH_Ar_), 122.1 (CH_Ar_), 125.0 (CH_Ar_), 125.4 (CH_Ar_), 131.9, 135.1, 145.4, 146.1, 148.0, 151.4, 152.5 (7 × C_Ar_), 168.8 (C=O); ^19^F NMR (376 MHz, CDCl_3_): δ −138.6 (d, ^3^*J* = 20.4 Hz), −148.6 (d, ^3^*J* = 20.4 Hz), −163.5 (t, ^3^*J* = 20.4 Hz) ppm; MS (ESI +): *m*/*z* = 1474 (M^+^ + H), 1491 (M^+^ + NH_4_), 1518 (M^+^ − H + 2 Na), 1534 (M^+^ − H + Na + K).

**Disodium 5**,**11**,**17**,**23-tetrakis(*tert*-butyl)-25**,**27-bis(*N*-heptafluoro-2-propanesulfonylcarbamoyl-methoxy)calix[4]arene-4-*tert*-butylcarboxybenzocrown-6 26**. Under Ar, compound **16e** (26 mg, 42 μmol), 6-chloro-1-hydroxybenzotriazole (9 mg, 51 μmol), EDC · HCl (10 mg, 51 μmol), and Et_3_N (7,1 μL, 5 mg, 51 μmol) were dissolved in anhydrous acetonitrile (8 mL) and stirred at 0 °C for 30 min. Afterwards, heptafluoropropane-2-sulfonamide (23 mg, 0.093 mmol) and Et_3_N (6.5 μL, 5 mg, 0.084 mmol) dissolved in anhydrous acetonitrile (5 mL) were added and the resulting mixture stirred at rt overnight. Next, the solvent was changed to DCM (15 mL) and the organic phase was washed with water (15 mL), saturated hydrogen carbonate solution (15 mL) and water (15 mL), dried over Na_2_SO_4_ and the solvent removed. The crude product was purified using column chromatography (petroleum ether:ethyl acetate 3:1 → 2:1) to obtain **26** as colourless solid (13 mg, 35%); mp 340 °C (decomp.); R*_f_* 0.3 (petroleum ether:ethyl acetate 2:1); ^1^H NMR (400 MHz, CDCl_3_): δ 1.10 (s, 18H, CH_3_), 1.17 (s, 18H, CH_3_), 1.61 (s, 9H, OC(CH_3_)_3_), 3.41 (d, ^2^*J* = 12.3 Hz, 4H, ArCH_2_), 3.98–4.10 (m, 8H, OCH_2_), 4.16 (d, ^2^*J* = 12.3 Hz, 4H, ArCH_2_), 4.21–4.31 (m, 8H, OCH_2_), 4.64 (s, 4H, OCH_2_CO), 6.86 (d, ^3^*J* = 8.5 Hz, 1H, ArH), 7.06 (s, 4H, ArH), 7.14 (s, 4H, ArH), 7.50 (d, ^4^*J* = 1.9 Hz, 1H, ArH), 7.67 ppm (dd, ^3^*J* = 8.5 Hz, ^4^*J* = 1.9 Hz, 1H, ArH); ^13^C NMR (101 MHz, CDCl_3_): δ 28.4 (O^t^Bu), 30.3 (ArCH_2_), 30.4 (ArCH_2_), 31.3 (^t^Bu), 31.4 (^t^Bu), 34.2 (C_q_), 34.3 (C_q_), 66.9 (OCH_2_), 67.2 (OCH_2_), 69.9 (OCH_2_), 70.0 (OCH_2_), 72.8 (OCH_2_), 72.9 (OCH_2_), 76.7 (OCH_2_CO), 78.0 (OCH_2_), 78.1 (OCH_2_), 80.9 (OC_q_), 110.2 (CH_Ar_), 111.8 (CH_Ar_), 124.0 (CH_Ar_), 125.4 (C_Ar_), 125.8 (CH_Ar_), 126.3 (CH_Ar_), 134.1, 134.2, 146.4, 147.9, 148.0, 148.1, 148.5, 150.3, 151.8, 151.9 (10 × C_Ar_), 165.6 (C=OOtBu), 176.1 (C=ONH); ^19^F NMR (376 MHz, CDCl_3_): δ −72.0 (m, 6F), −169.9 (hept, ^3^*J* = 7.3 Hz, 1F) ppm; MS (ESI +): *m*/*z* = 1595 (M^+^ + NH_4_), 1600 (M^+^ + Na), 1616 (M^+^ + K), 1622 (M^+^ − H + 2 Na).

**5**,**11**,**17**,**23-Tetrakis(*tert*-butyl)-25**,**27-bis(*N*-heptafluoro-2-propanesulfonylcarbamoyl-methoxy)calix[4]arene-benzocrown-8 27.** Under Ar, compound **16d** (135 mg, 0.12 mmol), 1-hydroxybenzotriazole (36 mg, 0.27 mmol), EDC · HCl (52 mg, 0.27 mmol), and Et_3_N (37 μL, 27 mg, 0.27 mmol) were dissolved in anhydrous acetonitrile (20 mL) and stirred at 0 °C for 30 min. Afterwards, heptafluoropropane-2-sulfonamide (122 mg, 0.49 mmol) and Et_3_N (34 μL, 25 mg, 0.24 mmol) dissolved in anhydrous acetonitrile (5 mL) were added and the resulting mixture stirred at rt overnight. Next, the solvent was changed to DCM (20 mL) and the organic phase was washed with water (20 mL), saturated hydrogen carbonate solution (20 mL) and water (20 mL), dried over Na_2_SO_4_ and the solvent removed. The crude product was purified using column chromatography (DCM:petroleum ether:ethyl acetate 3:8:2 → DCM:ethyl acetate 1:1) and washed with 10% aqueous HCl to obtain **27** as colourless solid (51 mg, 27%); mp 162 °C; R*_f_* 0.25 (DCM:petroleum ether:ethyl acetate 3:8:2); ^1^H NMR (400 MHz, CDCl_3_): δ 0.82 (s, 18H, CH_3_), 1.31 (s, 18H, CH_3_), 3.22 (d, ^2^*J* = 13.0 Hz, 4H, ArCH_2_), 3.77–3.79 (m, 4H, OCH_2_), 3.79–3.82 (m, 8H, OCH_2_), 3.98–4.00 (m, 4H, OCH_2_), 4.07–4.10 (m, 4H, OCH_2_), 4.20–4.23 (m, 4H, OCH_2_), 4.55 (d, ^2^*J* = 13.0 Hz, 4H, ArCH_2_), 5.28 (s, 4H, OCH_2_CO), 6.50 (s, 4H, ArH), 6.84–6.89 (m, 2H, ArH), 6.90–6.95 (m, 2H, ArH), 7.08 (s, 4H, ArH); ^13^C NMR (151 MHz, CDCl_3_): δ 33.7 (^t^Bu), 34.2 (^t^Bu), 34.7 (ArCH_2_), 36.2 (C_q_), 36.7 (C_q_), 69.4 (OCH_2_), 71.5 (OCH_2_), 71.9 (OCH_2_), 72.9 (OCH_2_), 73.3 (OCH_2_CO), 73.4 (OCH_2_), 114.5 (CH_Ar_), 123.7 (CH_Ar_), 127.5 (CH_Ar_), 128.4 (CH_Ar_), 134.7, 137.8, 147.7, 148.1, 150.4, 154.0 (6 × C_Ar_); ^19^F NMR (564 MHz, CDCl_3_): δ −71.7 (d, ^3^*J* = 8.0 Hz, 6F), −168.5 (m, 1F) ppm; MS (ESI +): *m*/*z* = 1394 (M^+^ − H − NHSO_2_CF(CF_3_)_2_ + K + Na), 1625 (M^+^ − H + K + Na).

## 4. Conclusions

Two sets of new functionalized calixarene ligands were prepared and analysed via UV/vis titration regarding their potential to chelate divalent cations like Ba, Sr and Pb. These ions are of high importance, e.g., as diagnostic or therapeutic radionuclides in radiopharmacy, or they are found in environmental chemistry. Thus, there exists a keen necessity for a strong complexation of these ions. In summary, the additional proton-ionizable side functions connected together with a benzocrown ether structure to the calixarene skeleton led to a considerable improvement of the complex stability resulting in high association constants. There is still room to improve the stability of these complexes for future application as chelators for radiometal ions in radiopharmacy.

## Figures and Tables

**Table 1:** Computed binding energies of different types of calixcrowns (B3LYP-D3/def2-TZVP).

Calixarene	Crown Type	ΔE (kcal/mol)
**20**	Crown-6	−100.9
**22**	Propylene-crown-6	−93.1
**23**	Benzocrown-6	−90.2
**28**	Crown-5	−89.9

**Table 2:** Determined logK values using the UV titration method.

Calixarene	logK(Ba)	logK(Sr)	logK(Pb)
**5**	6.1 ± 0.3	3.8	5.7 ± 0.2
**6**	--- ^(a)^	--- ^(a)^	--- ^(a)^
**7a**	4.4 ± 0.2	4.9 ± 0.2	5.0 ± 0.6
**7b**	4.6 ± 0.3	3.2 ± 0.2	5.4 ± 0.1
**14a**	4.6 ± 0.1 ^(b)^	4.3 ± 0.1 ^(b)^	3.3 ± 1 ^(b)^
**20**	5.7 ± 0.1	n.d.	n.d.
**21**	5.0 ± 0.8	5.0 ± 0.8	5.5 ± 0.8
**22**	4.9 ± 0.7	5.6 ± 0.8	7.4 ± 1.5 ^(c)^
**23**	6.6 ± 1.0 ^(c)^	7.0 ± 1.4 ^(c)^	4.0 ± 0.2
**24**	5.8 ± 0.9	6.4 ± 1.0 ^(c)^	6.3 ± 0.9 ^(c)^
**25**	7.5 ± 1.5 ^(c)^	7.6 ± 1.5 ^(c)^	8.0 ± 1.6 ^(c)^
**27**	5.2 ± 0.8	7.0 ± 1.4 ^(c)^	3.9 ± 0.1

^(a)^ No value available from UV titration, ^(b)^ see ref. [29], ^(c)^ Value over the upper method detection limit, n.d.—not determined.

## Data Availability

All data are included in this paper.

## Supplementary Materials

The following supporting information can be downloaded online. Table S1: Calculated bond lengths for Ba-O bonds of calix[4]crown-6 (20) and calix[4]crown-5 (BP86-D3/def2-TZVP), Figure S1: Partly labelled schemes of calix[4]crown-6 (20) and calix[4]crown-5, Table S2: the optimized coordinates (in Angstrom) of Ba-calix[4]crown complex calculated using BP86/TZVP, Table S3: The optimized coordinates (in Angstrom) of Ra-calix[4]crown complex calculated using BP86/TZVP, Figures S2–S34: ^1^H and ^13^C NMR spectra of compounds, Figures S35–S64: UV spectra and UV titration plot of compounds with Ba^2+^, Sr^2+^ and Pb^2+^.

## Sample Availability

Samples of the compounds are not available from the authors.
