# Supplementary material for: Synthesis of Ionizable Calix[4]arenes for Chelation of Selected Divalent Cations
Source: Molecules. 2022 Feb 22;27(5):1478. doi: 10.3390/molecules27051478 (PMC8911665; doi:10.3390/molecules27051478)
Supplement: Supplementary file 1 [file molecules-27-01478-s001.zip › molecules-1595506-supplementary.pdf]

Electronic Supplementary Information

Synthesis of Ionizable Calix[4]arenes for Chelation of Selected Divalent Cations

Markus Blumberg [1,2], Karrar Al-Ameed [3,†], Erik Eiselt [1], Sandra Luber [3] and Constantin Mamat [1,2,*]

[1] Helmholtz-Zentrum Dresden-Rossendorf, Institut für Radiopharmazeutische Krebsforschung, Bautzner Landstraße 400, D-01328 Dresden, Germany; blumberg.markus@yahoo.de (M.B.); erik.eiselt@gmx.de (E.E.)
[2] Fakultät Chemie und Lebensmittelchemie, Technische Universität Dresden, D-01062 Dresden, Germany
[3] Department of Chemistry, University of Zurich, Winterthurerstrasse 190, CH-8057 Zürich, Switzerland; karrar.al-ameed@linacre.ox.ac.uk (K.A.-A.); sandra.luber@chem.uzh.ch (S.L.)
* Correspondence: c.mamat@hzdr.de
† Current address: Department of Chemistry, University of Kufa, Najaf 54001, Iraq.

Content

Geometrical parameters of calix[4]-crown-5 compared to calix[4]-crown-6 ………………….. 2

^{1}H and ^{13}C NMR spectra of compounds ……………………………………………………….. 11

UV spectra and titration plots of final compounds ……………………………………………… 46

Geometrical parameters of calix[4]-crown-5 compared to calix[4]-crown-6

Table S1. Calculated bond lengths for Ba-O bonds of calix[4]crown-6 (**20**) and calix[4]crown-5 (BP86-D3/ def2-TZVP).

Bond (Å)	Calix[4]crown-6 (20)	Calix[4]crown-5
Ba–O13	2.87	2.84
Ba–O14	3.04	2.76
Ba–O8	2.86	2.91
Ba–O18	2.99	2.75
Ba–O9	2.96	2.73
Ba–O12	2.93	2.76
Ba–O15	2.60	2.61
Ba–O19	2.68	2.67
Ba–O10	2.97	2.80

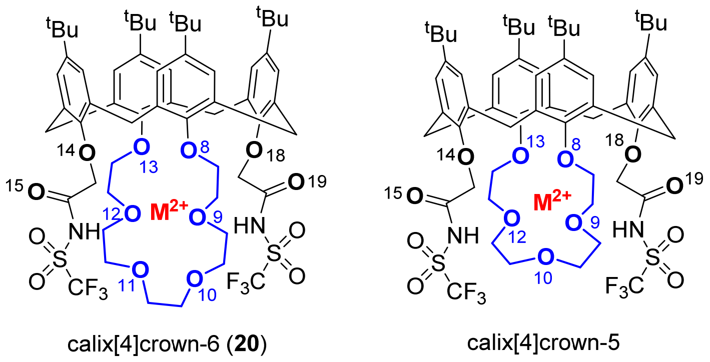

Figure S1. Partly labelled schemes of calix[4]crown-6 (**20**) and calix[4]crown-5.

Table S2. The optimized coordinates (in Angstrom) of Ba-calix[4]crown complex calculated using BP86/TZVP.

Ba	3.006274600	8.473328500	5.561369300
S	1.111080000	11.355733500	1.453487800
S	6.695885000	9.947123100	9.257986000
F	2.468807900	12.555828200	3.497650100
F	0.286616300	12.584820200	3.745035800
F	5.316350500	8.189961000	10.814113600
F	4.142096200	9.960005300	10.281943900
O	1.929030700	6.405186800	7.224960700
O	0.452150100	8.695604600	7.063708300
O	2.141174900	10.960414000	6.948841900
O	4.677125800	10.941319300	5.783472600
O	5.544756100	8.923092100	4.131219000
O	3.649633000	7.293648600	3.037189200
O	0.665762100	7.302770900	4.036404600
O	1.462430100	9.895428500	4.008223800
O	2.482062500	10.945478600	1.160462100
O	0.332762700	12.026146200	0.432880800
O	4.950572400	6.393068800	6.182397200
O	4.529666700	8.506524000	7.765142000
O	6.167850700	11.116427900	8.562225800
O	7.928519300	10.068100300	10.009313000
N	0.212124500	10.129489500	2.046659900
N	6.802175400	8.620103700	8.335760200
C	1.519967200	5.075566200	7.366229800
C	2.405126600	4.171905200	7.979563500
C	1.937916800	2.887943800	8.265272100
H	2.618098100	2.192846900	8.760498400
C	0.645813600	2.464017100	7.915800900
C	-0.164283600	3.373347200	7.231364300
H	-1.164325800	3.077725400	6.918521000
C	0.259075700	4.675106600	6.911279100
C	0.201332400	1.029618100	8.234580500
C	1.198120500	0.038180600	7.590642900
H	2.210205500	0.153111100	8.001782600
H	1.256502600	0.191757600	6.503415200
H	0.880647800	-0.998573300	7.776790200
C	0.188003500	0.826217400	9.765580000
H	-0.521922400	1.516157300	10.244032400
H	1.179493400	1.004980000	10.204794600
H	-0.110595800	-0.203185000	10.014341000
C	-1.201030700	0.717499100	7.685603200
H	-1.968668300	1.372408600	8.123304000
H	-1.471087300	-0.318880800	7.931909100
H	-1.240822200	0.817902100	6.590257200
C	-0.557502900	5.537205000	5.965073800
H	-0.317113600	6.598391000	6.089241600
H	-1.629954700	5.401352200	6.173630700
C	0.355092000	6.009571100	3.618224800
C	-0.269685400	5.130821700	4.522153200
C	-0.569315400	3.838716600	4.084978900
H	-1.035985600	3.153057900	4.791030300

C	-0.267281700	3.384810400	2.795529300
C	0.358384200	4.286674900	1.933524800
H	0.639284100	3.971000600	0.930546500
C	0.691837200	5.591915000	2.319486000
C	-0.560252300	1.926874600	2.414206500
C	0.291674700	1.007724800	3.320080000
H	0.062661900	1.162561700	4.384364400
H	1.362085700	1.211284200	3.176925500
H	0.105472600	-0.050615000	3.082008600
C	-2.057911300	1.617207500	2.627335500
H	-2.685672500	2.273053400	2.007177600
H	-2.355837100	1.757240600	3.675718300
H	-2.275237700	0.573427300	2.355430900
C	-0.202568200	1.624425900	0.948679600
H	-0.777633100	2.250873800	0.251254500
H	-0.432078400	0.573696900	0.721463000
H	0.869025500	1.778725900	0.750863200
C	1.509190900	6.449295600	1.357031700
H	1.037148600	6.440660100	0.364631300
H	1.545329500	7.490507200	1.691544900
C	3.875416100	6.181775400	2.222229400
C	2.900974500	5.847197000	1.271804300
C	3.187659200	4.824855700	0.357925600
H	2.433650300	4.588054400	-0.391520400
C	4.388922500	4.107914800	0.396564100
C	5.294155400	4.424213000	1.419619300
H	6.228338600	3.865650600	1.499558400
C	5.052509400	5.431132400	2.362222700
H	2.644301300	2.540288500	-1.216245400
H	5.579219300	1.685130500	0.996387300
C	4.704869800	2.960772200	-0.574533100
C	3.621546000	2.792852700	-1.653645000
H	3.505727800	3.703320900	-2.259680500
H	3.899545100	1.973524100	-2.331207900
C	6.053588300	3.227432700	-1.278128800
H	6.015073600	4.165080200	-1.850647600
H	6.289618300	2.407897300	-1.973060100
H	6.881831500	3.304968100	-0.560263300
C	4.791260700	1.643538800	0.230526200
H	5.014031100	0.797532400	-0.436461000
H	3.838960500	1.438349300	0.741332100
C	5.951713400	5.592812300	3.574401200
H	5.891722300	6.611753600	3.971311400
H	6.996486800	5.400618800	3.285552500
C	5.030487600	5.026611200	5.898462400
C	5.543294700	4.602502200	4.661080200
C	5.631259300	3.227654000	4.423348600
H	6.000982000	2.899908400	3.453276900
C	5.247800900	2.268533800	5.367644300
C	4.729534200	2.736958900	6.577707600
H	4.384829700	2.025911100	7.326735900
C	4.593075100	4.100919100	6.860451800

C	6.591686600	0.421622100	4.261037500
H	6.653960000	0.935754600	3.292458100
H	6.626078400	-0.658607100	4.056970900
H	7.481808100	0.689383400	4.848189400
C	5.226190300	-0.113287100	6.280250400
H	4.266625500	-0.005854900	6.805267200
H	6.036309000	0.122131900	6.985099100
H	5.319125000	-1.170536500	5.993661500
C	4.075415000	0.461590300	4.128446900
H	3.141709500	0.682891800	4.665171300
H	4.065027900	-0.599893800	3.837747200
H	4.093002100	1.070378100	3.213531400
C	5.297143100	0.772976100	5.023265500
C	3.874421000	4.526428600	8.138039600
H	4.298930700	3.989437600	8.998388500
H	4.004217700	5.601417800	8.307532800
C	1.687635500	7.183862700	8.434833000
H	2.550053400	7.856076900	8.553790800
H	1.651297700	6.512017900	9.305771600
C	0.396307200	7.965535400	8.301149500
H	-0.480106400	7.295566200	8.284011700
H	0.299339800	8.641348200	9.167591200
C	-0.035819600	10.045731800	7.114474300
H	-1.006119100	10.093471600	7.637946000
H	-0.172113300	10.341947600	6.065111800
C	0.972986800	10.973171800	7.761574400
H	0.551573600	11.995331000	7.820777700
H	1.213960300	10.647202000	8.792285200
C	3.233974400	11.708201900	7.520544200
H	3.768928800	11.085834300	8.251046300
H	2.851499100	12.617227500	8.017504100
C	4.168692300	12.122550600	6.414905700
H	3.647251000	12.748892500	5.670470200
H	5.000150100	12.688892200	6.868081000
C	5.795101100	11.201312600	4.937500500
H	6.510493800	11.875388500	5.443133800
H	5.461192300	11.680781500	3.997734600
C	6.473236600	9.871464400	4.683748400
H	7.349986000	9.992103800	4.025426400
H	6.808005800	9.453021500	5.643246400
C	5.411544300	8.956717300	2.698928500
H	6.154332200	8.274100400	2.250067200
H	5.574510800	9.973066500	2.302309600
C	3.994138300	8.546600000	2.367336900
H	3.283733100	9.308784000	2.714935900
H	3.851511900	8.450153000	1.282478700
C	-0.257693100	8.311003200	3.539836700
H	-0.981883600	8.549416500	4.338362300
H	-0.796209000	7.934076700	2.661550500
C	0.560444700	9.551164900	3.191557300
C	1.293105300	12.676129500	2.845911900
C	6.095513100	6.870920000	6.917463200

H	6.920962200	7.099237200	6.223893100
H	6.445721700	6.092957000	7.614025700
C	5.726729900	8.118905400	7.727932200
C	5.383981200	9.526177200	10.607949300
F	1.252032500	13.903855500	2.291826700
F	5.733408400	10.118545700	11.764643400

Table S3. The optimized coordinates (in Angstrom) of Ra-calix[4]crown complex calculated using BP86/TZVP.

Ra	2.972567300	8.486182400	5.594293500
S	1.173815300	11.361228100	1.400476700
S	6.822563200	10.025521500	9.088524700
F	2.305543800	12.671621100	3.517938300
F	0.110495100	12.685733500	3.531667900
F	5.659849800	8.355523400	10.886046100
F	4.345503900	9.999460100	10.280023600
O	1.899591200	6.389901200	7.276398000
O	0.398516700	8.686305300	7.195437800
O	2.095368200	10.982883600	7.038037400
O	4.612042700	10.980407000	5.775627500
O	5.541025700	8.952073500	4.135541400
O	3.663236100	7.288449700	3.037469200
O	0.630481100	7.337448200	4.001917500
O	1.350487900	9.979097500	4.029085800
O	2.577133800	10.988390900	1.234294500
O	0.462395800	11.968704000	0.294842500
O	4.959372500	6.362359000	6.225558000
O	4.580883900	8.457209500	7.853989400
O	6.217138900	11.145283800	8.373724800
O	8.106768900	10.211336000	9.732111000
N	0.265667600	10.127773200	1.961768800
N	6.888647700	8.655890600	8.228764700
C	1.492863700	5.057392000	7.381416200
C	2.375620000	4.142202100	7.981388300
C	1.902935800	2.855079900	8.242295900
H	2.577683700	2.149381600	8.729981500
C	0.610814400	2.442448300	7.880342100
C	-0.191118200	3.362274600	7.199892400
H	-1.189819000	3.072607400	6.876576300
C	0.238251200	4.667274900	6.900320500
C	0.161605900	1.004703100	8.177484000
C	1.143427500	0.021765900	7.498063500
H	2.163610600	0.129363000	7.890595400
H	1.180919600	0.193095900	6.412645900
H	0.827635100	-1.017438600	7.673235300
C	0.170421900	0.769104100	9.704072400
H	-0.526211000	1.454203500	10.208234000
H	1.169797100	0.929839100	10.132389800
H	-0.132656900	-0.263027700	9.935569700
C	-1.250973300	0.712240500	7.644698800
H	-2.005727600	1.367710900	8.103247500
H	-1.526360700	-0.325503400	7.879585200
H	-1.306551300	0.828506900	6.551626700
C	-0.560376700	5.547692900	5.952266300
H	-0.310868300	6.605678700	6.089691400
H	-1.636147100	5.423316900	6.151357400
C	0.345585400	6.033512600	3.596214800
C	-0.268508700	5.149856700	4.505410800
C	-0.552731700	3.852891300	4.070879200
H	-1.016526900	3.165145800	4.776161200

C	-0.240073000	3.394761200	2.785437900
C	0.386196800	4.296289500	1.924878100
H	0.681397800	3.975976200	0.927843300
C	0.702717400	5.607374900	2.305242200
C	-0.527396300	1.935635000	2.404683300
C	0.269600200	1.012353400	3.354774800
H	-0.009826000	1.171628900	4.405893000
H	1.347700300	1.203899000	3.263837600
H	0.083765700	-0.044812500	3.110836900
C	-2.036937800	1.645609900	2.550710300
H	-2.627007900	2.299045900	1.892212400
H	-2.381313300	1.806215000	3.582015800
H	-2.253971000	0.600323400	2.284047800
C	-0.103916200	1.617460100	0.960075300
H	-0.645641300	2.236735800	0.230457200
H	-0.324606100	0.564771600	0.733325800
H	0.976152200	1.769134700	0.810284500
C	1.530816300	6.458194000	1.344631900
H	1.064841300	6.449646700	0.349356000
H	1.569859300	7.500412400	1.675399700
C	3.889796600	6.176354900	2.225416500
C	2.919870900	5.846156400	1.269046500
C	3.205906300	4.816572500	0.363214800
H	2.457192400	4.581283500	-0.391827800
C	4.400471000	4.088970900	0.417839500
C	5.300435800	4.403378400	1.446590100
H	6.229106600	3.837657400	1.535758900
C	5.059023800	5.416789700	2.382581100
H	2.651097400	2.514575500	-1.180613200
H	5.580852800	1.666444100	1.041262900
C	4.713252600	2.931259000	-0.541719500
C	3.630092400	2.757052700	-1.620140400
H	3.519360700	3.661002900	-2.236450500
H	3.903977000	1.928593400	-2.288384800
C	6.064481200	3.185708200	-1.244934900
H	6.030490200	4.116690800	-1.828331900
H	6.300311700	2.357284500	-1.929584500
H	6.890607000	3.269266200	-0.525324300
C	4.793620200	1.621080500	0.275173000
H	5.014217300	0.768377700	-0.384124600
H	3.840117500	1.423524500	0.786836900
C	5.947481400	5.579346100	3.603556300
H	5.877612400	6.597992900	4.000852500
H	6.996130800	5.396044500	3.322866200
C	5.027430300	4.999504200	5.927102500
C	5.538677100	4.582768400	4.686043300
C	5.624252100	3.208682700	4.441032800
H	5.997110100	2.885016900	3.471304500
C	5.235302500	2.244913700	5.378157200
C	4.707227200	2.706462600	6.586778500
H	4.354314000	1.990893600	7.327378800
C	4.574642000	4.068821200	6.878175200

C	6.565190900	0.406159100	4.239512800
H	6.603841800	0.918371400	3.268801800
H	6.601685000	-0.674140800	4.036550000
H	7.465799800	0.680040100	4.807429800
C	5.244724800	-0.138794000	6.285036600
H	4.294722900	-0.040196800	6.828680900
H	6.066407700	0.102168200	6.974389600
H	5.340180500	-1.194824900	5.994807900
C	4.047023600	0.436113100	4.157908000
H	3.123782000	0.648172600	4.715919100
H	4.036916000	-0.623734600	3.860831500
H	4.042575400	1.050903000	3.246837500
C	5.285142000	0.750531800	5.028516400
C	3.845961200	4.491023000	8.151670600
H	4.261410200	3.949293400	9.013621800
H	3.979619800	5.565140800	8.324636900
C	1.665360400	7.139297400	8.505409500
H	2.524763300	7.815222000	8.630073200
H	1.645457500	6.447434000	9.361222100
C	0.364852900	7.912531800	8.406009500
H	-0.502144000	7.230858300	8.370365100
H	0.264093500	8.555847400	9.296513700
C	-0.065433000	10.041690100	7.300743700
H	-1.010283600	10.090263300	7.869082400
H	-0.246693400	10.363501400	6.265460300
C	0.981061300	10.946047800	7.923335500
H	0.559349700	11.961287000	8.057098900
H	1.289751500	10.574360800	8.919993000
C	3.207776500	11.740539600	7.556374500
H	3.779158700	11.127418100	8.268629800
H	2.839411800	12.650240100	8.062917000
C	4.093338300	12.156551900	6.409689500
H	3.531593700	12.758673300	5.675334200
H	4.924926600	12.749803600	6.826225700
C	5.734980900	11.246064200	4.935259200
H	6.436994300	11.931345700	5.443979900
H	5.400629400	11.715041800	3.990629700
C	6.439100700	9.926424800	4.694709300
H	7.321496300	10.064329700	4.047461300
H	6.770997900	9.518291600	5.659668400
C	5.403885200	8.979936600	2.703788100
H	6.155592300	8.306755900	2.255890600
H	5.549872300	9.997164700	2.303214600
C	3.992358600	8.546129700	2.370426700
H	3.269401300	9.299400200	2.712609700
H	3.855118400	8.449337600	1.284946100
C	-0.284267900	8.334933500	3.460861800
H	-1.046111000	8.563632100	4.226316000
H	-0.775519700	7.947376500	2.560410300
C	0.531018900	9.590466600	3.150120200
C	1.205032700	12.744955200	2.740559600
C	6.120999800	6.832605800	6.939766300

H	6.942412900	7.035826700	6.234513200
H	6.463046200	6.060453200	7.647365400
C	5.780868600	8.101537700	7.731929400
C	5.629321000	9.666864800	10.555636300
F	1.216470100	13.943473600	2.123499000
F	6.015522500	10.386588400	11.625772300

^{1}H and ^{13}C NMR spectra of compounds

Compound 1

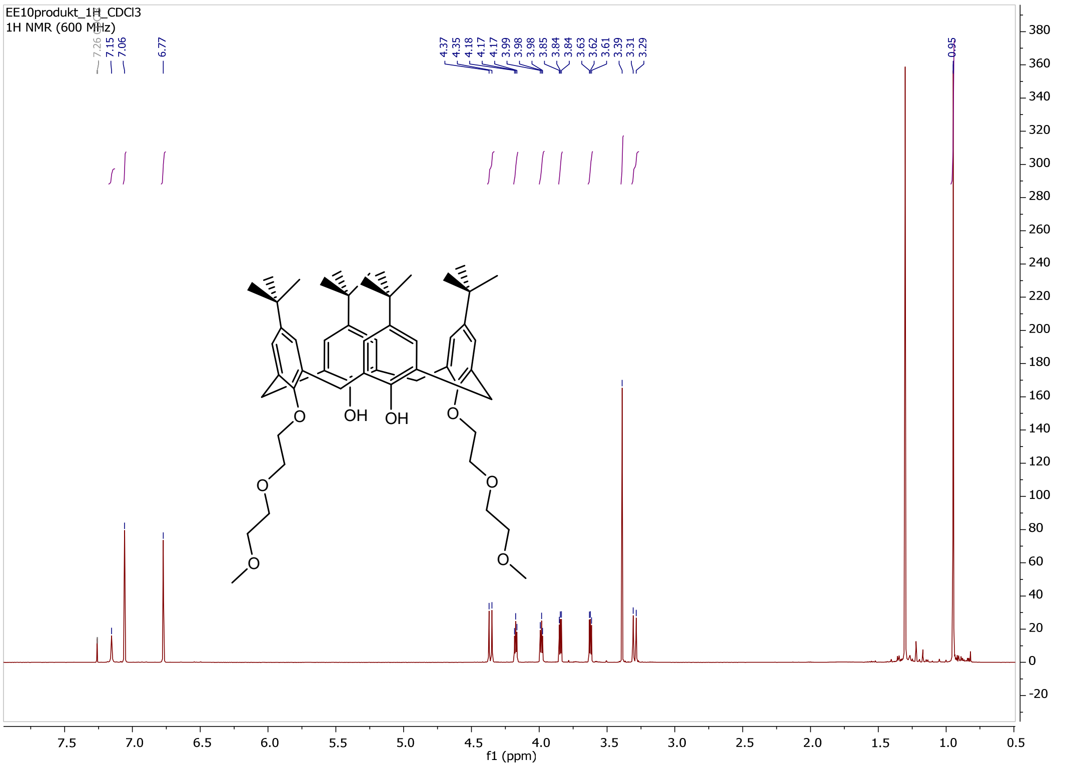

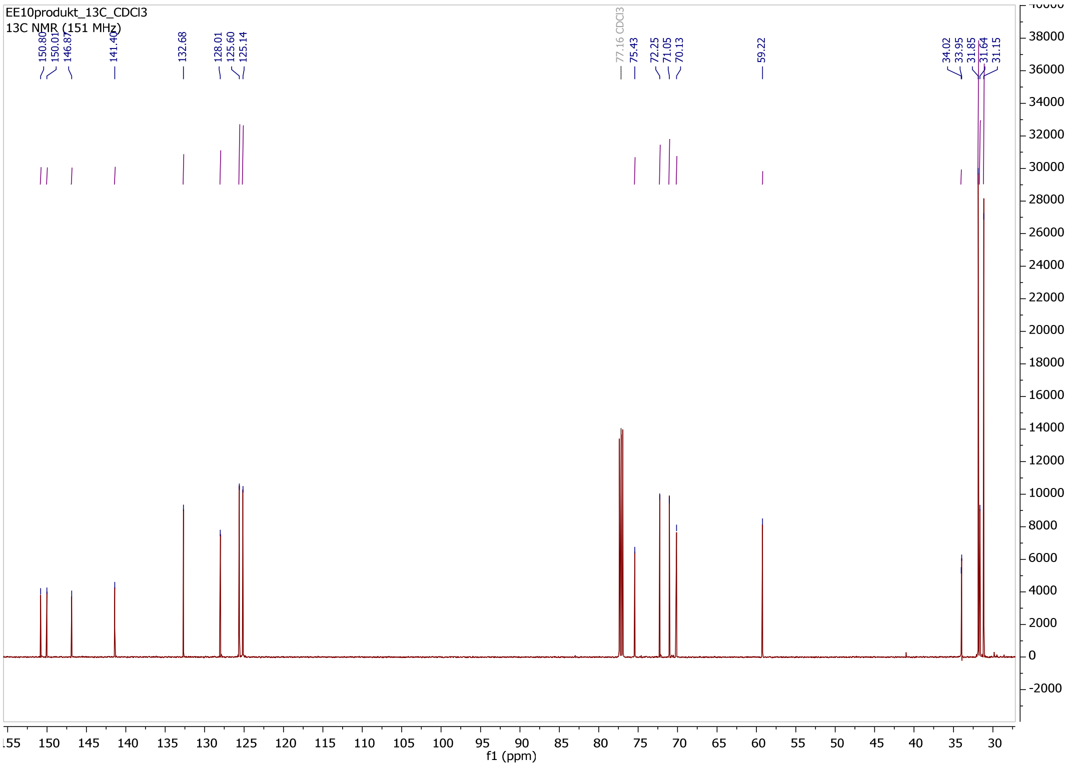

Figure S2. ^{1}H and ^{13}C NMR spectra of compound **1**.

Compound **2**

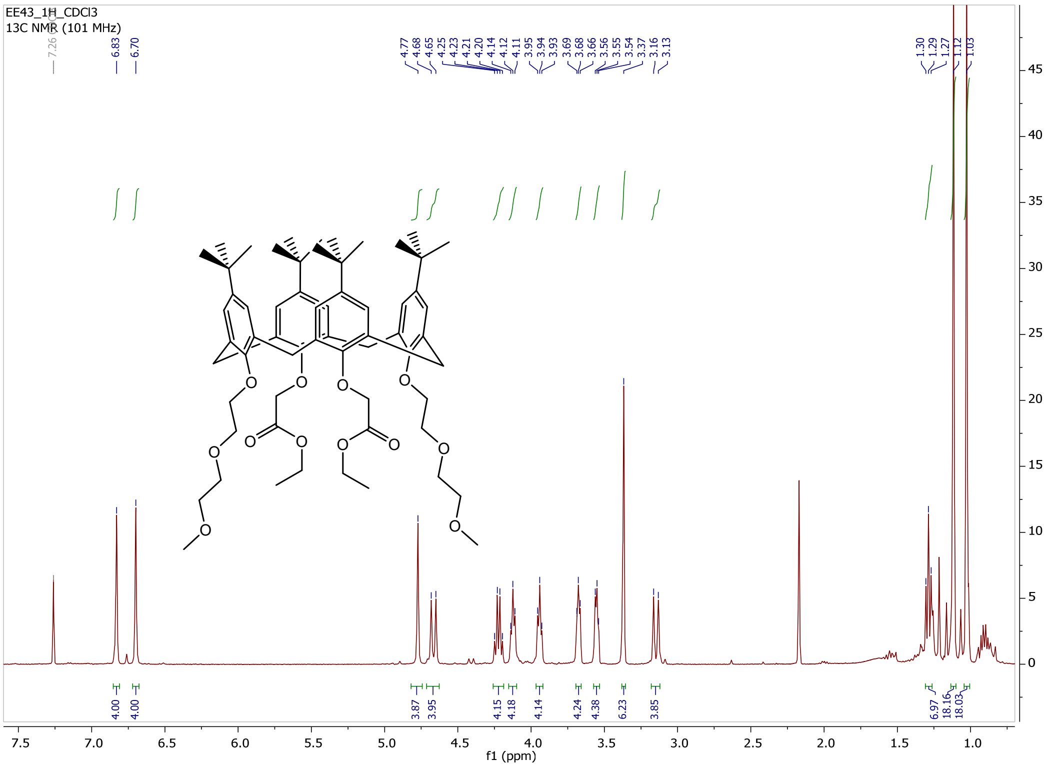

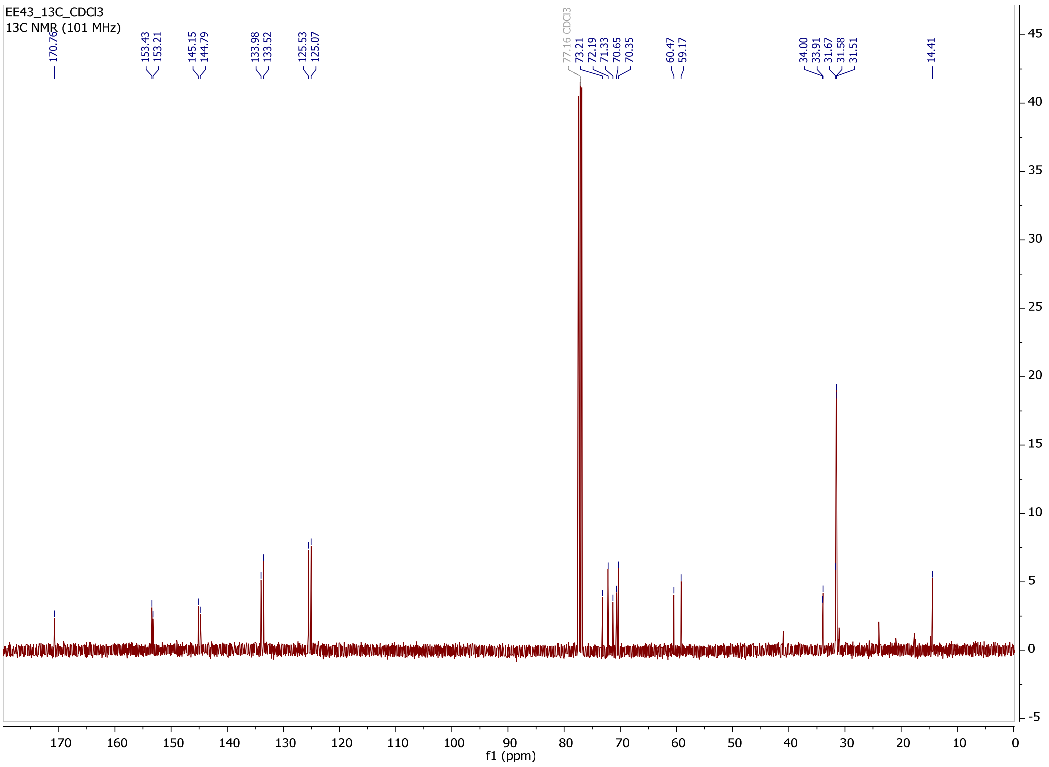

Figure S3. ^{1}H and ^{13}C NMR spectra of compound **2**.

Compound **3**

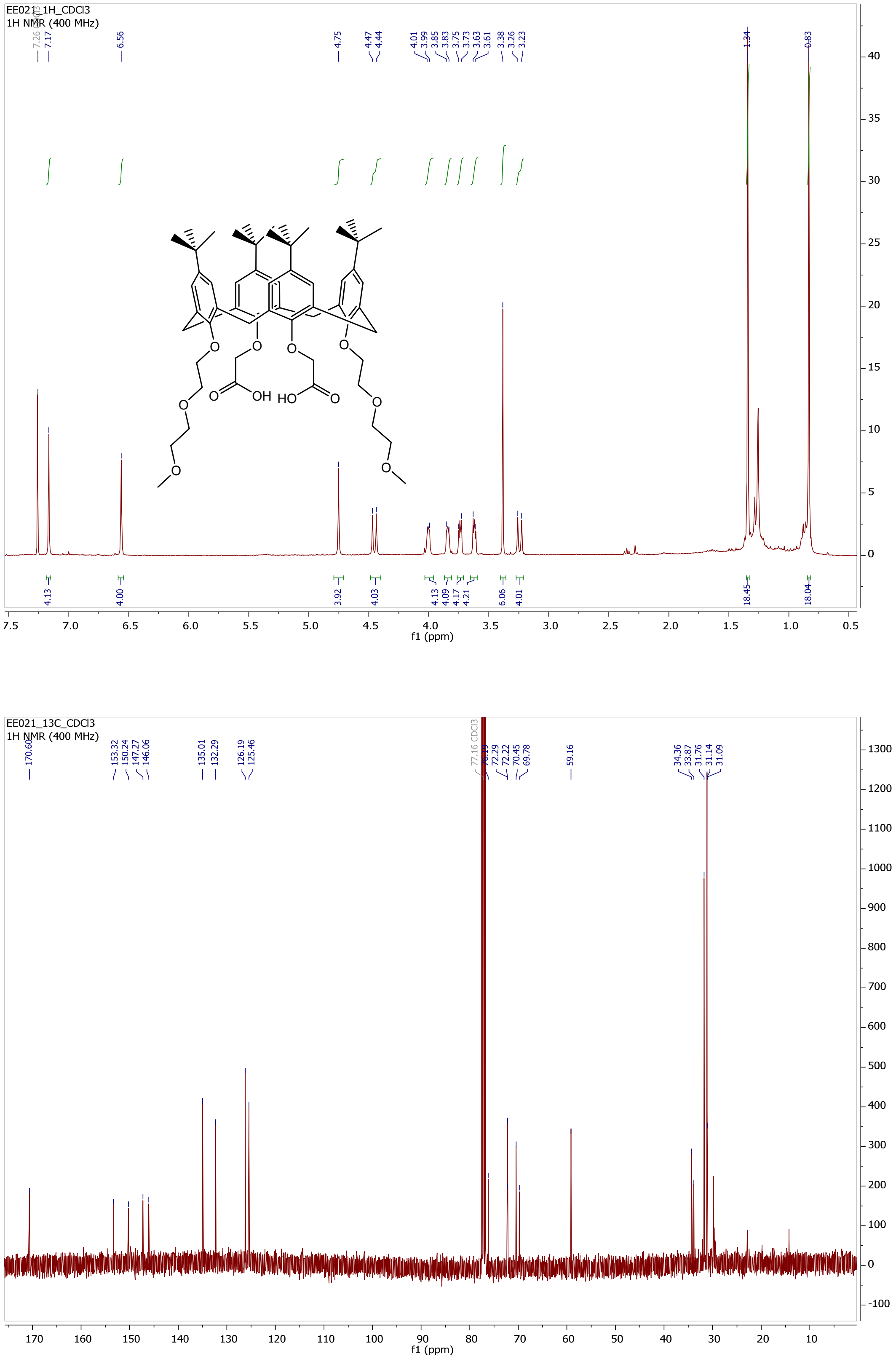

Figure S4. ^{1}H and ^{13}C NMR spectra of compound **3**.

Compound **5**

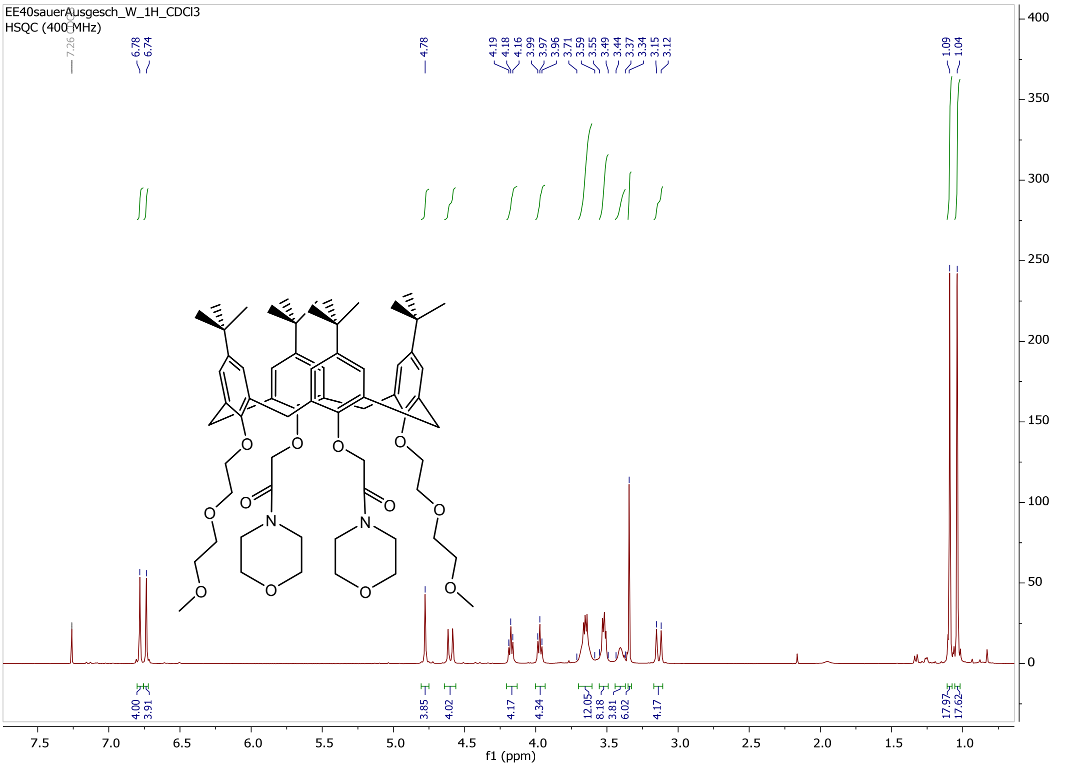

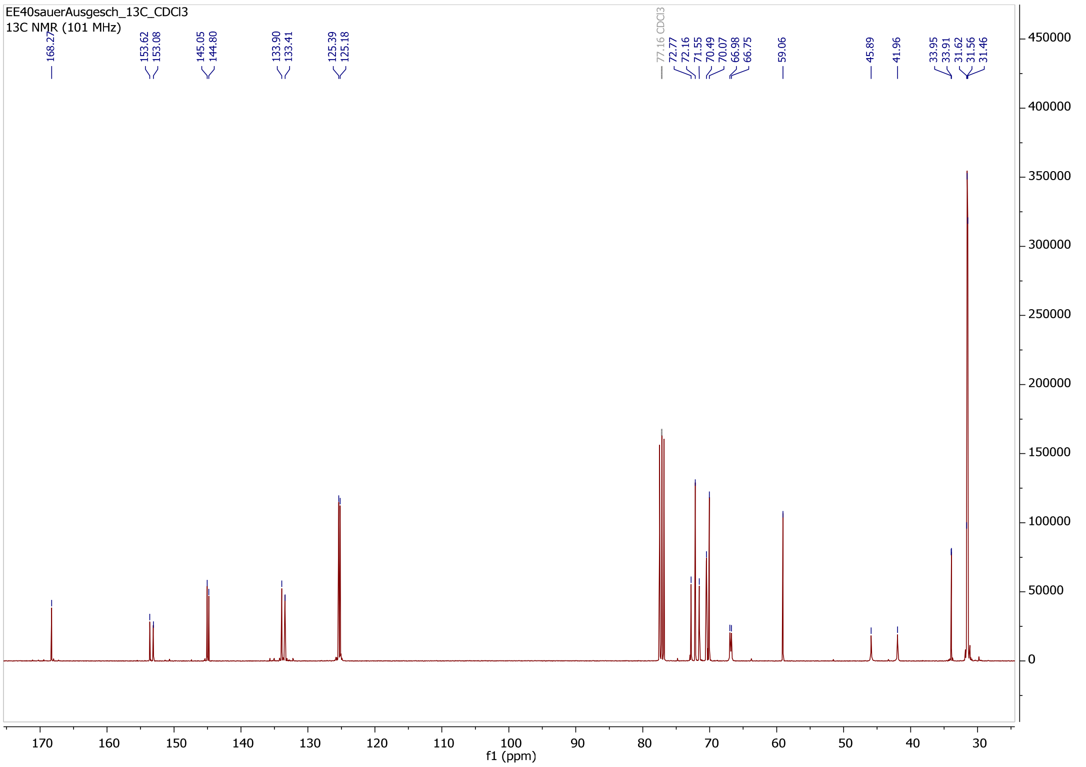

Figure S5. ^{1}H and ^{13}C NMR spectra of compound **5**.

Compound **6**

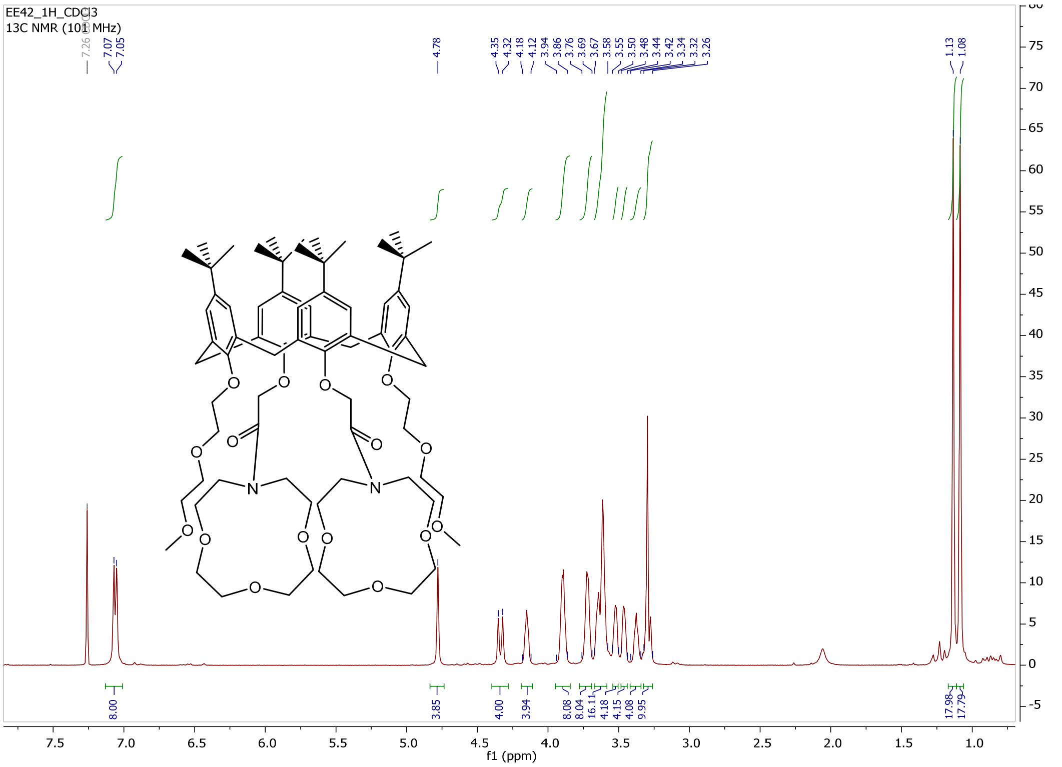

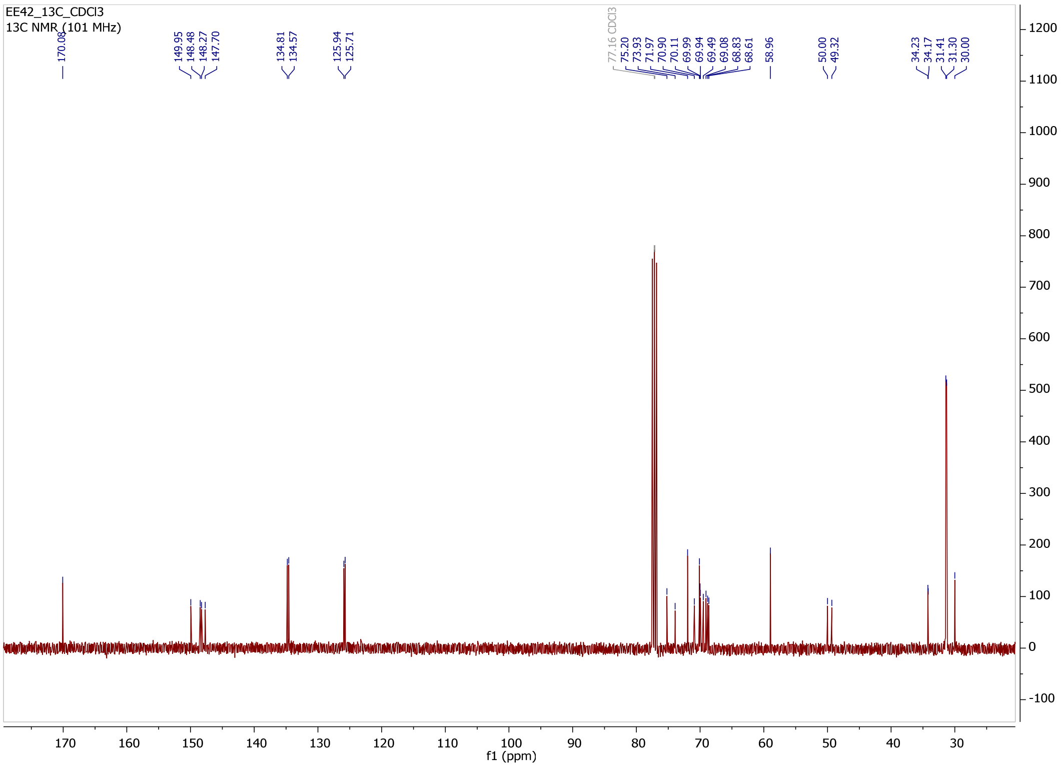

Figure S6. ^{1}H and ^{13}C NMR spectra of compound **6**.

Compound **7a**

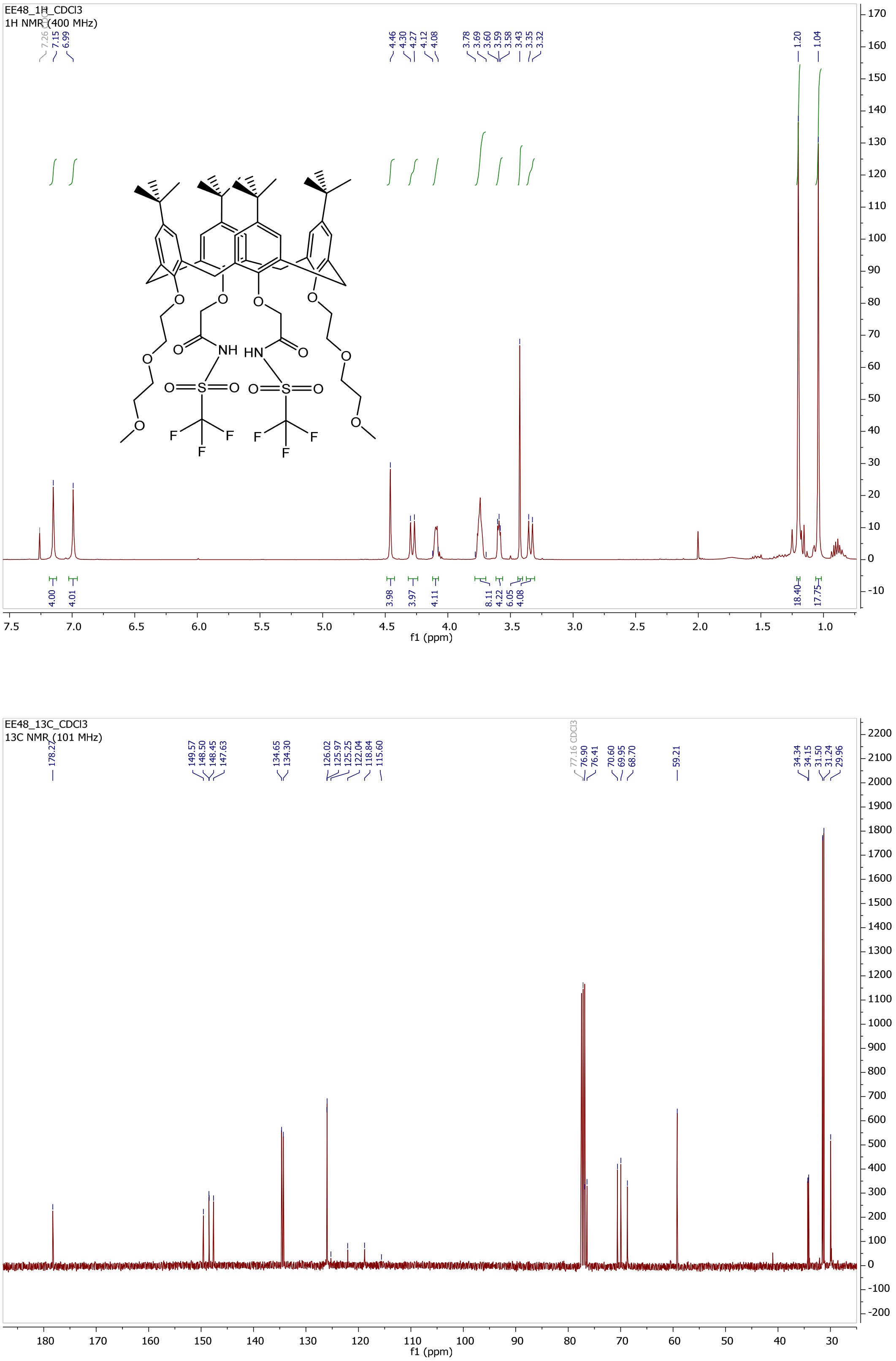

Figure S7. ^{1}H and ^{13}C NMR spectra of compound **7a**.

Compound **7b**

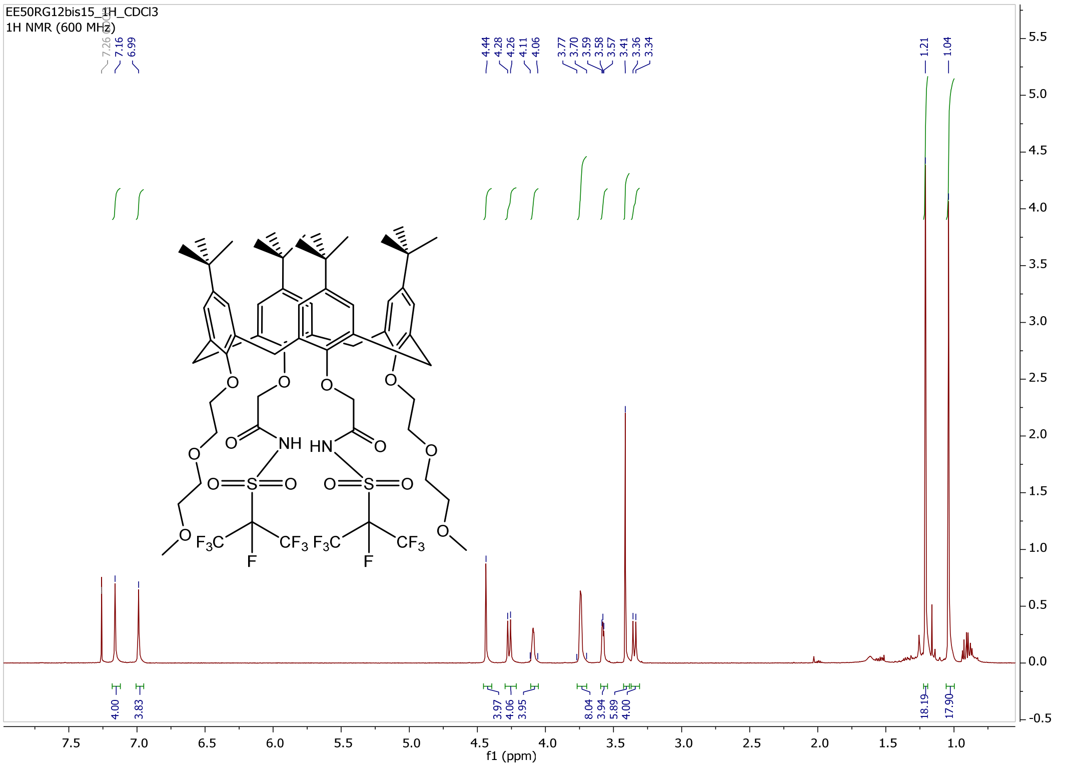

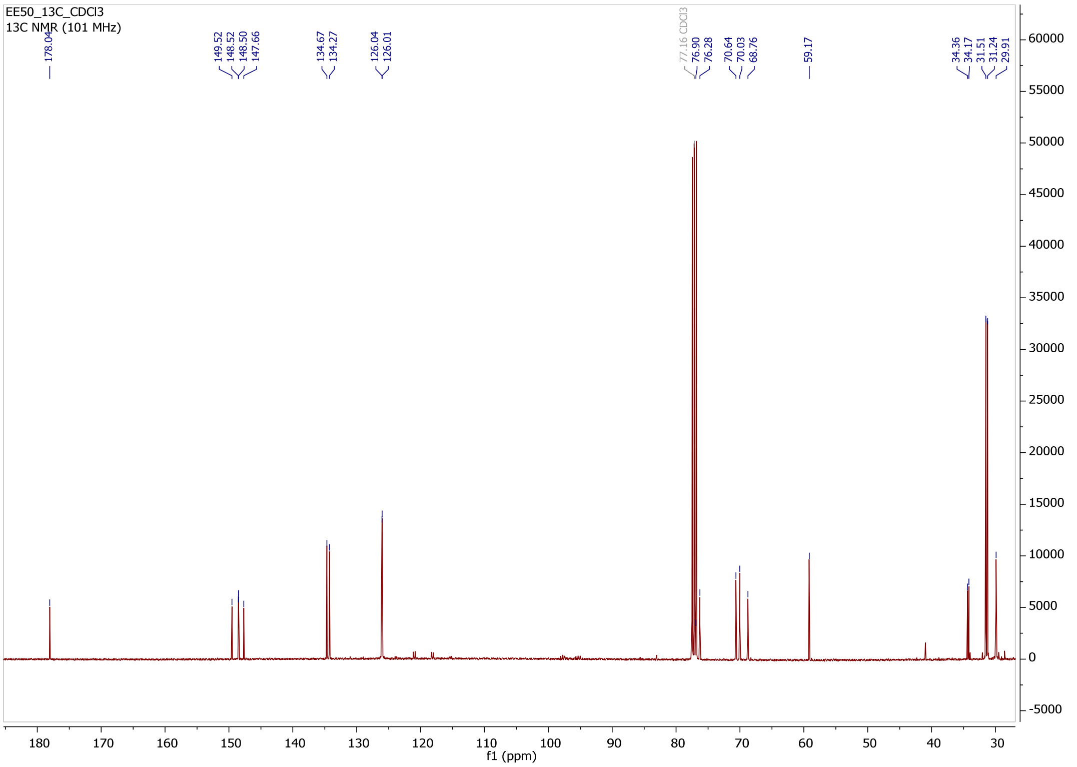

Figure S8. ^{1}H and ^{13}C NMR spectra of compound **7b**.

Compound **7c**

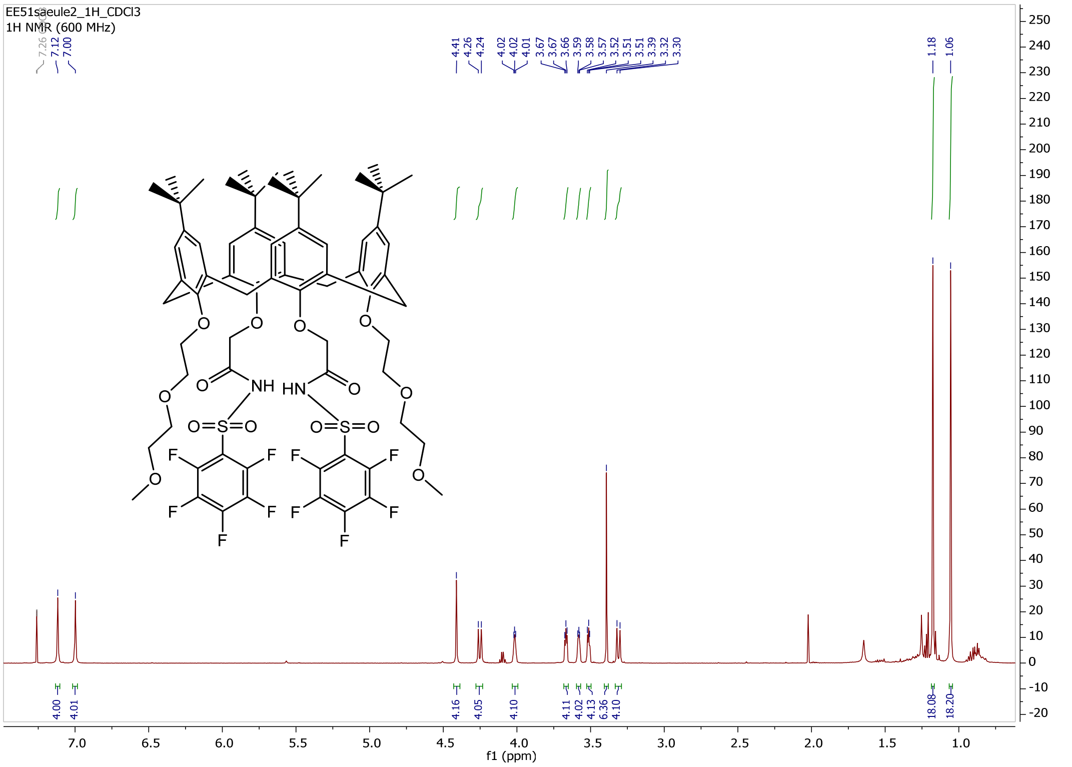

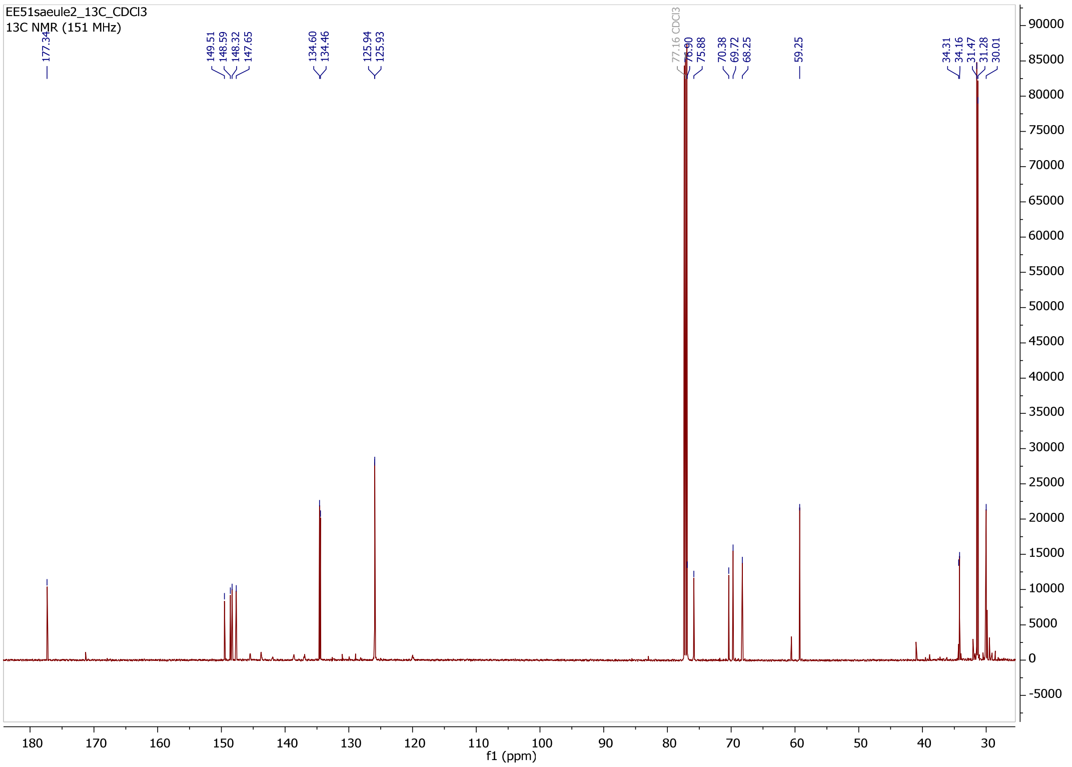

Figure S9. ^{1}H and ^{13}C NMR spectra of compound **7c**.

Compound **8b**

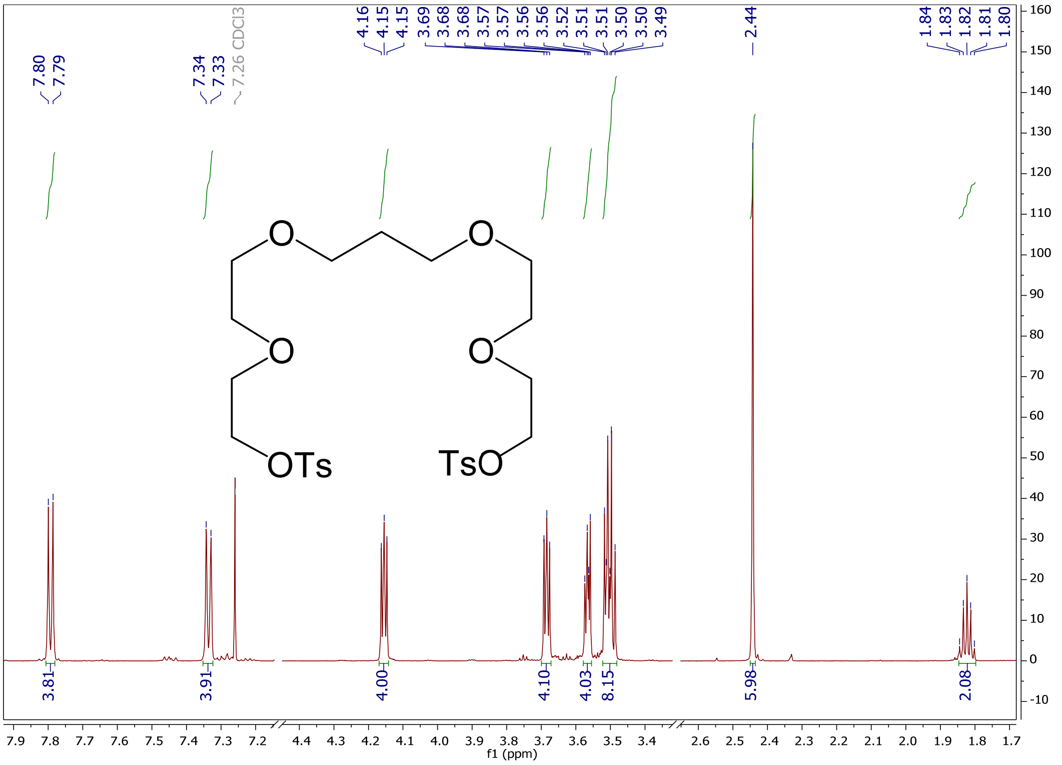

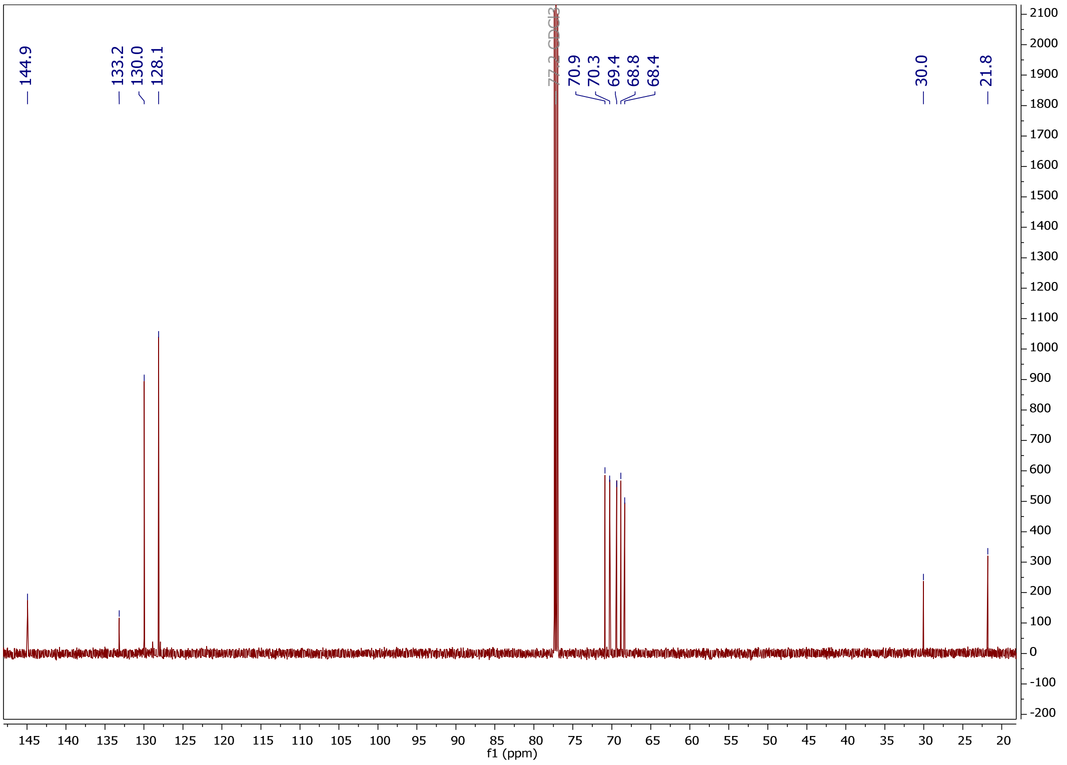

Figure S10. ^{1}H and ^{13}C NMR spectra of compound **8b**.

Compound **11c**

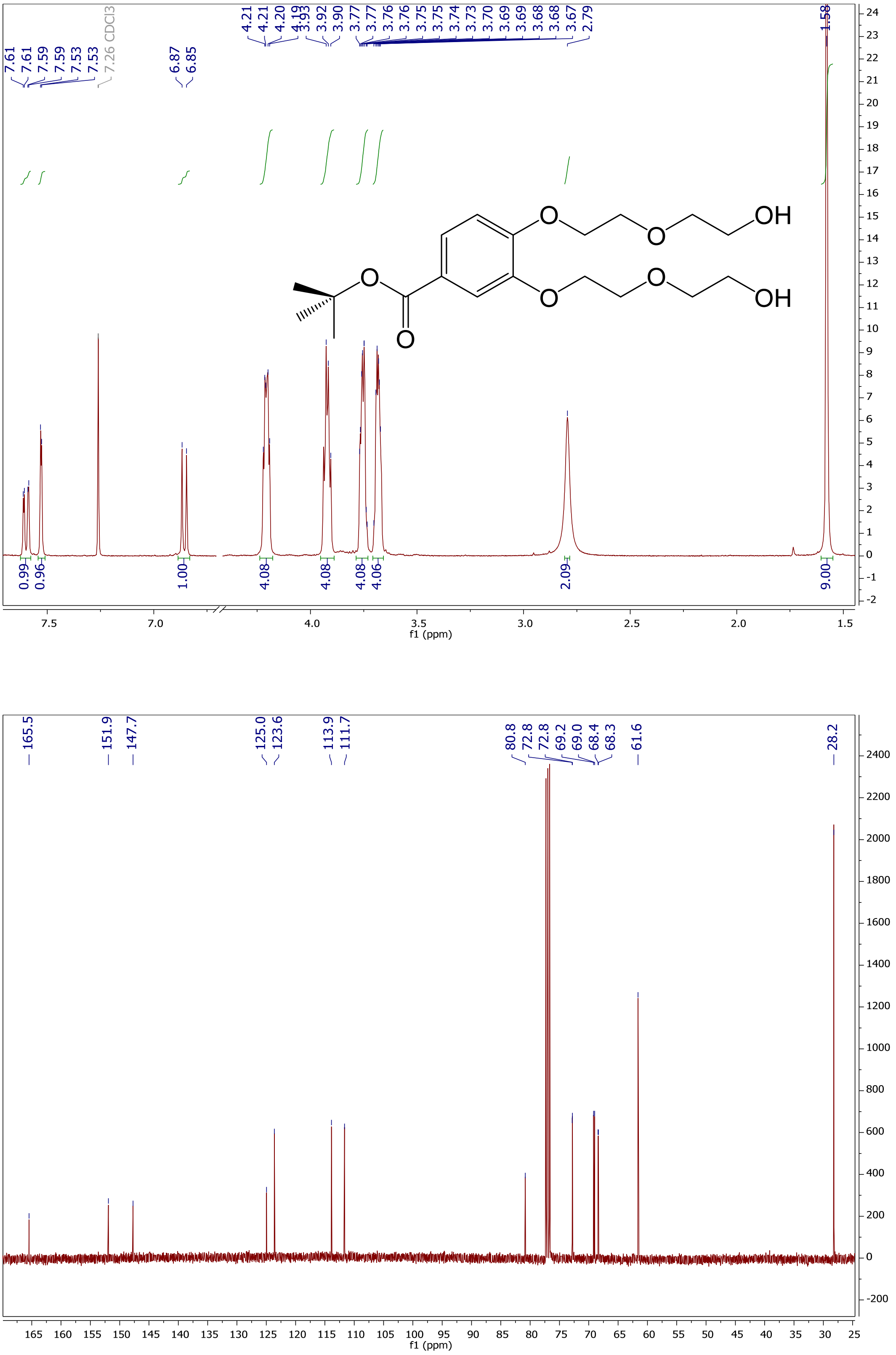

Figure S11. ^{1}H and ^{13}C NMR spectra of compound **11c**.

Compound **11d**

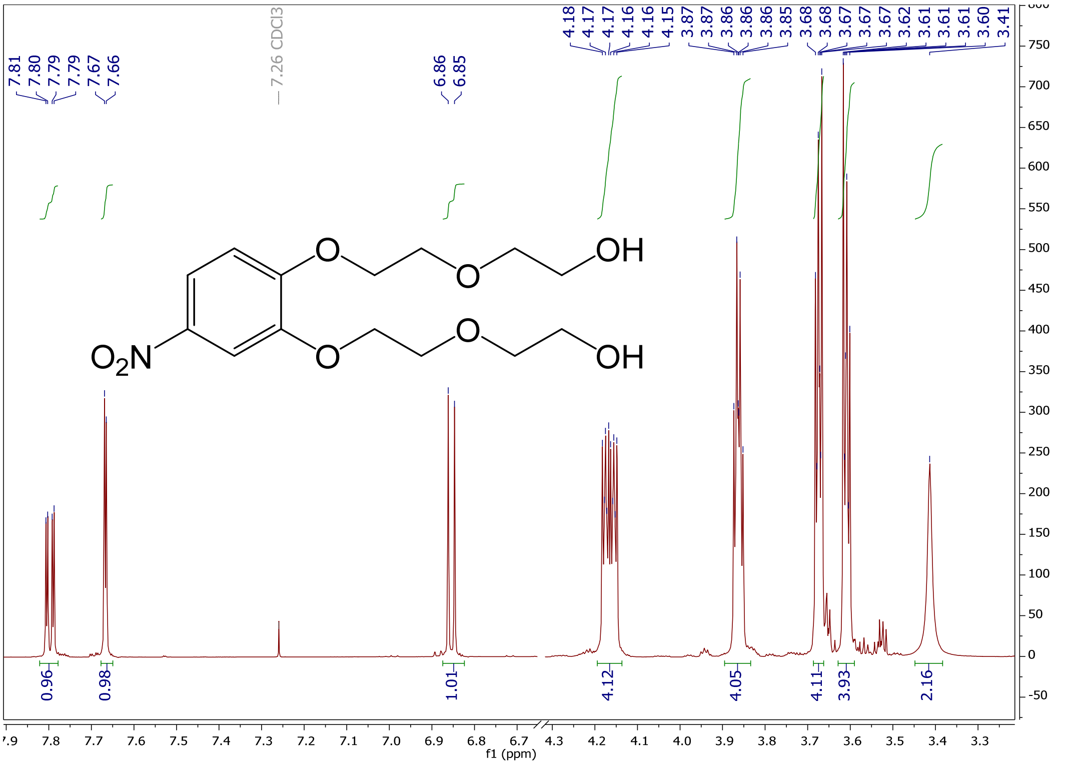

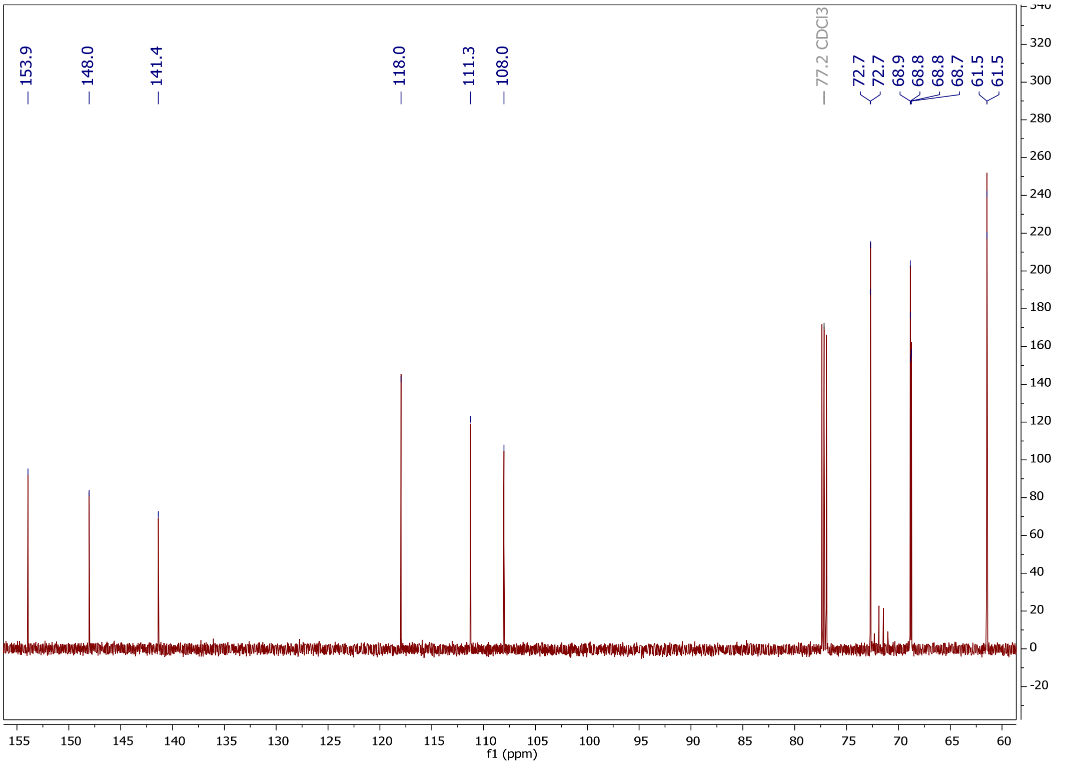

Figure S12. ^{1}H and ^{13}C NMR spectra of compound **11d**.

Compound **12a**

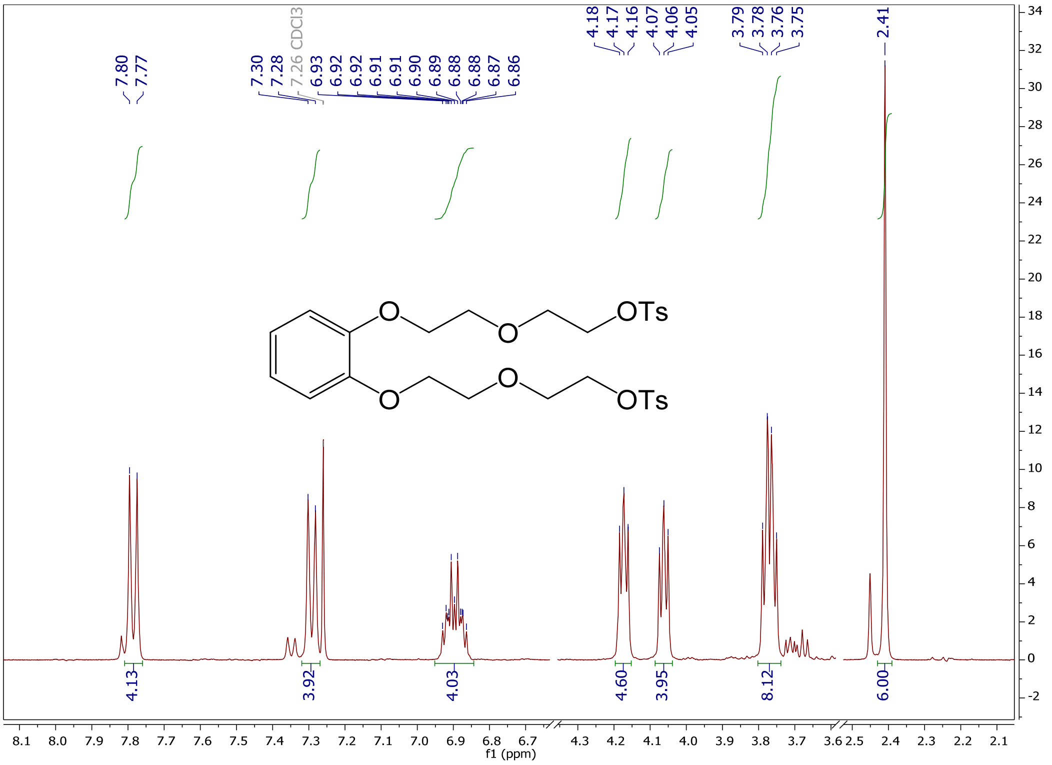

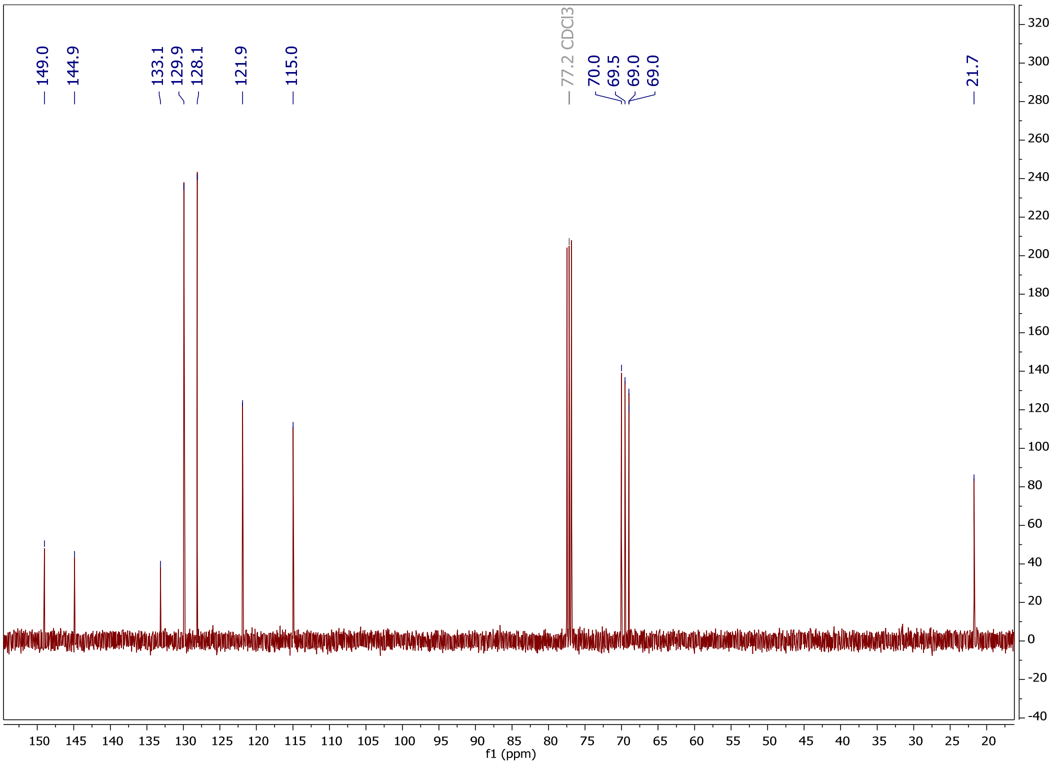

Figure S13. ^{1}H and ^{13}C NMR spectra of compound **12a**.

Compound **12b**

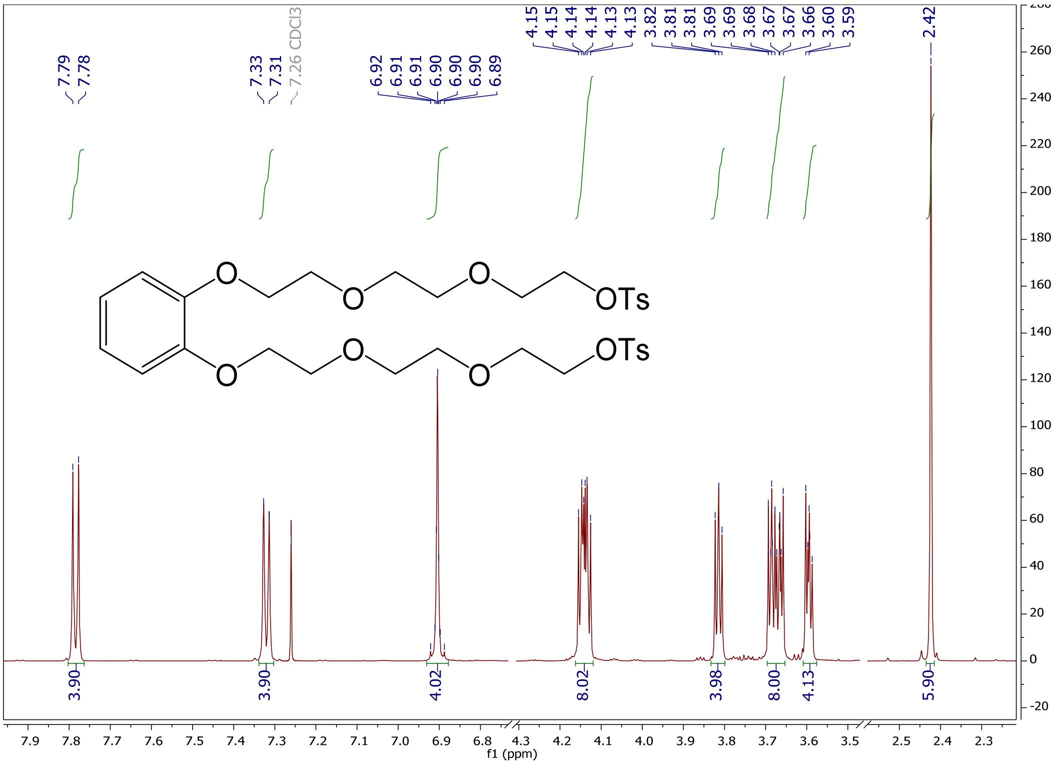

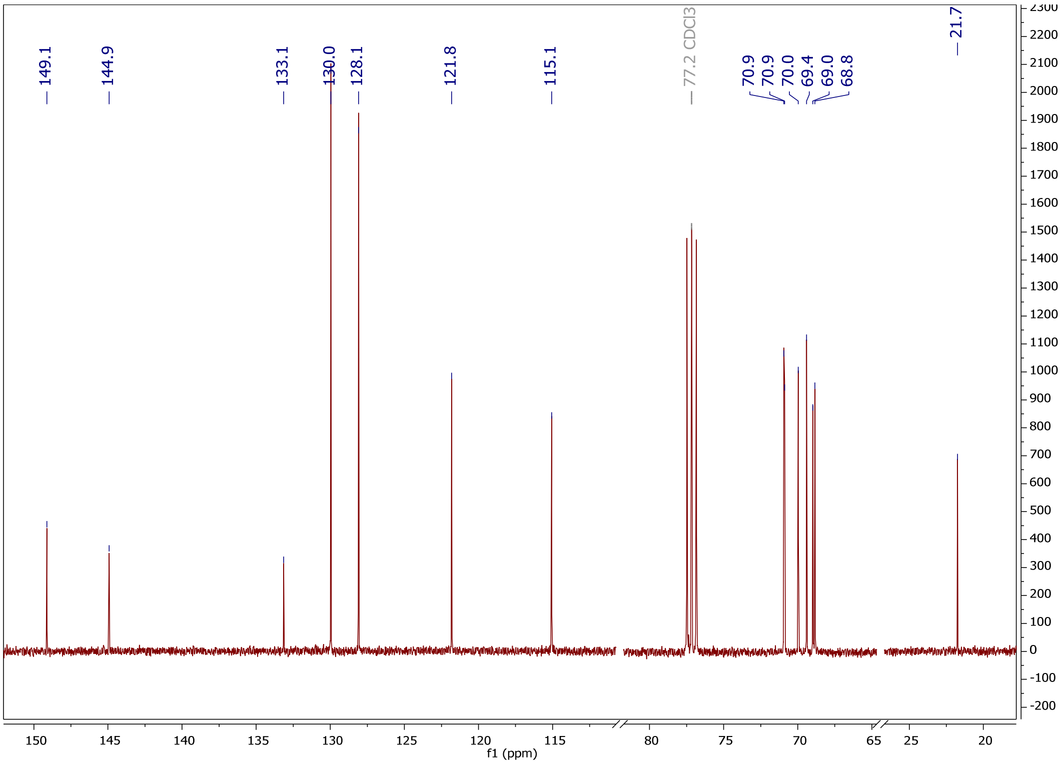

Figure S14. ^{1}H and ^{13}C NMR spectra of compound **12b**.

Compound **12c**

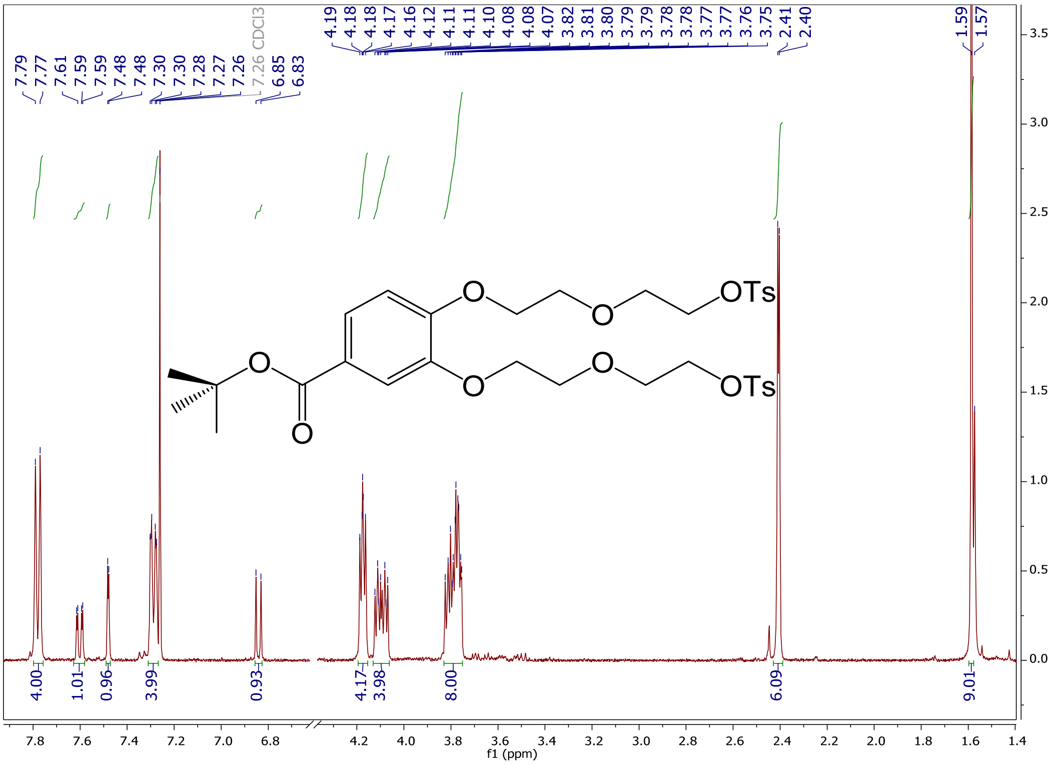

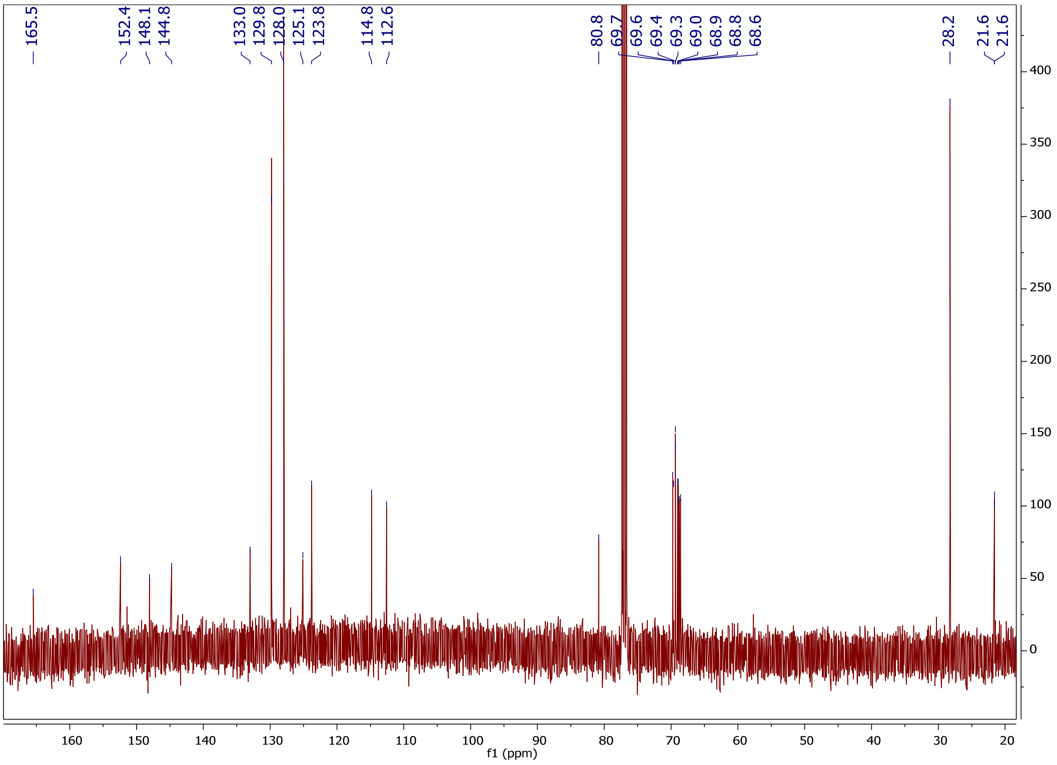

Figure S15. ^{1}H and ^{13}C NMR spectra of compound **12c**.

Compound **12d**

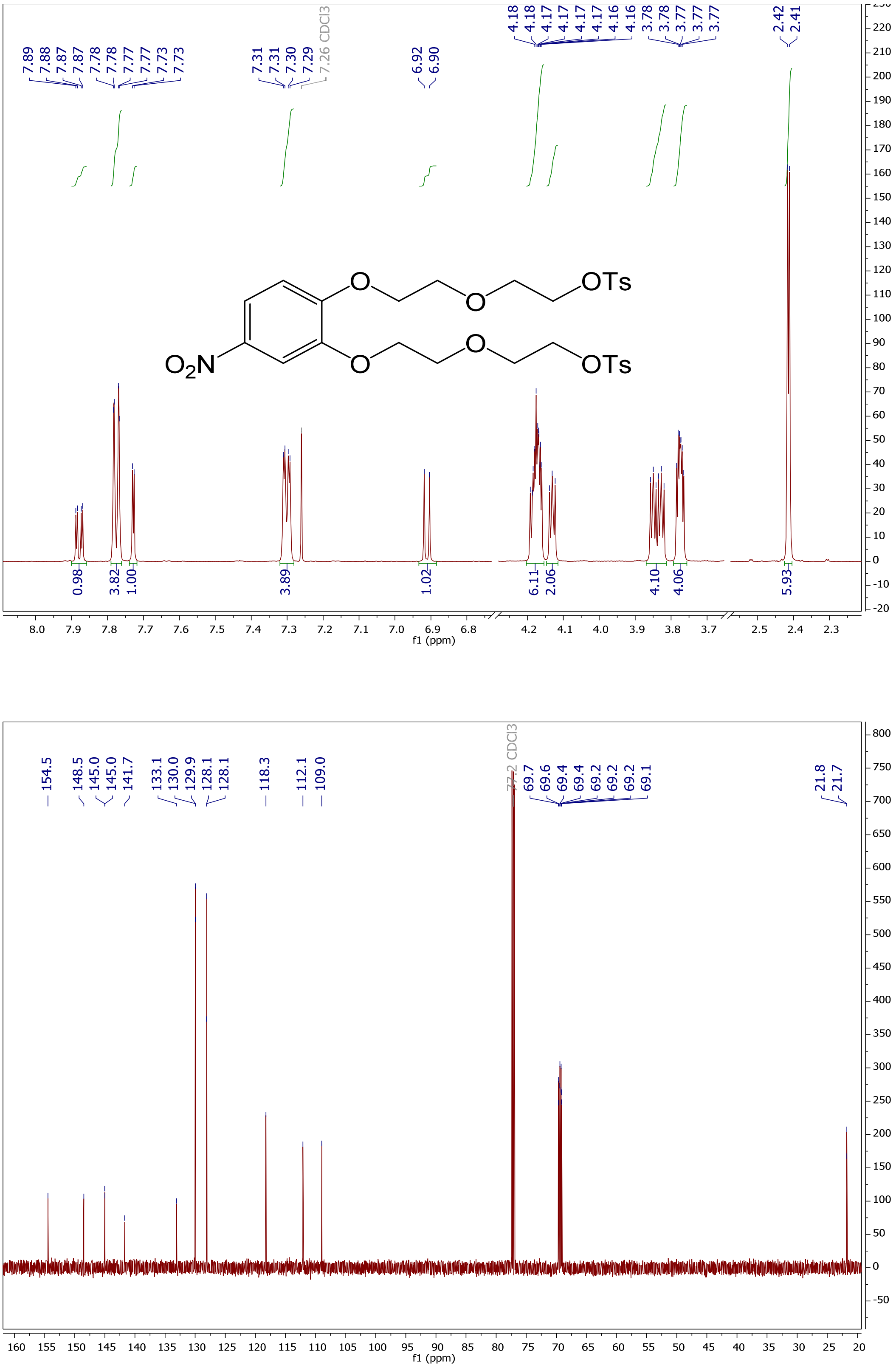

Figure S16. ^{1}H and ^{13}C NMR spectra of compound **12d**.

Compound **14b**

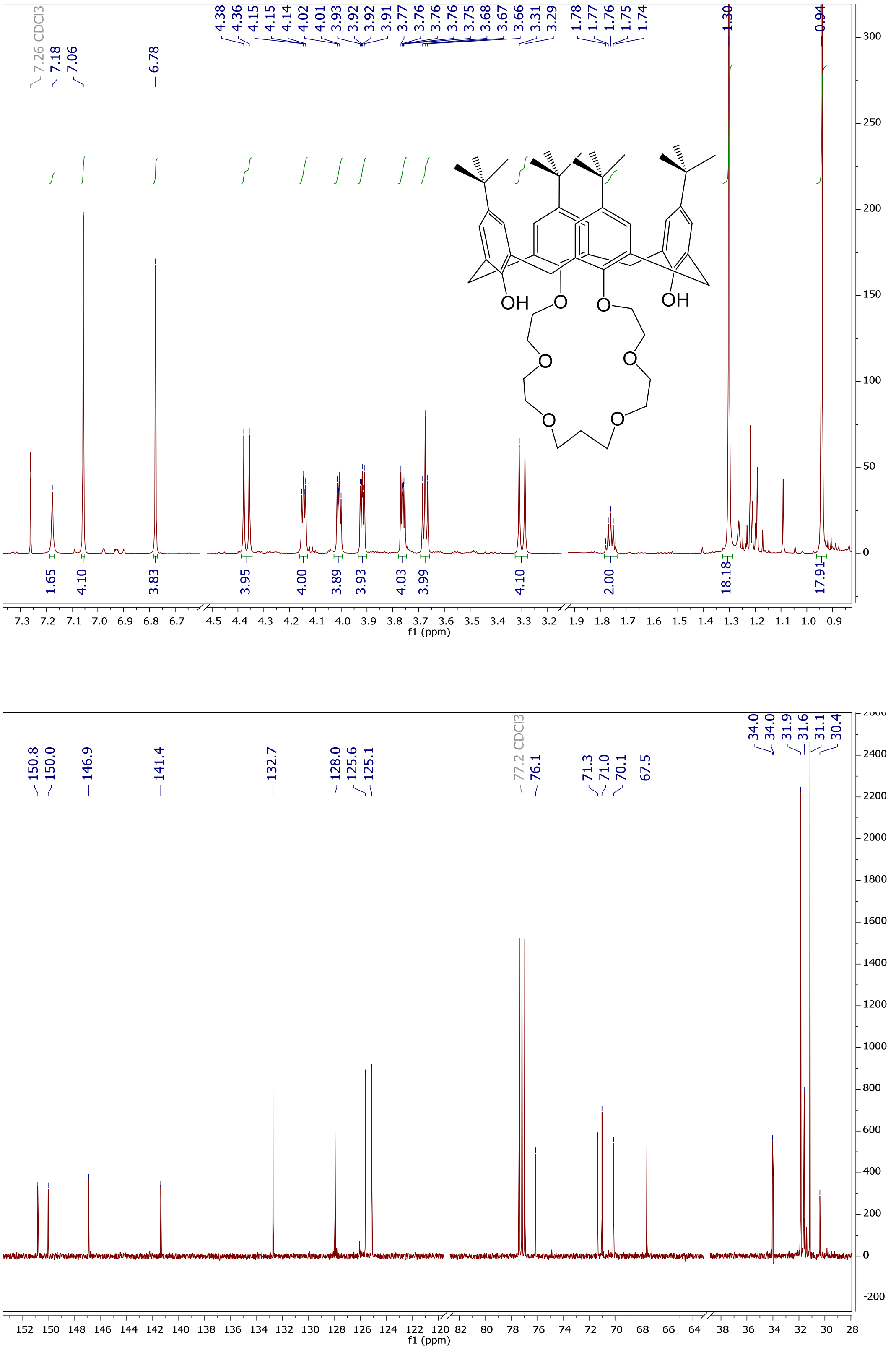

Figure S17. ^{1}H and ^{13}C NMR spectra of compound **14b**.

Compound **14c**

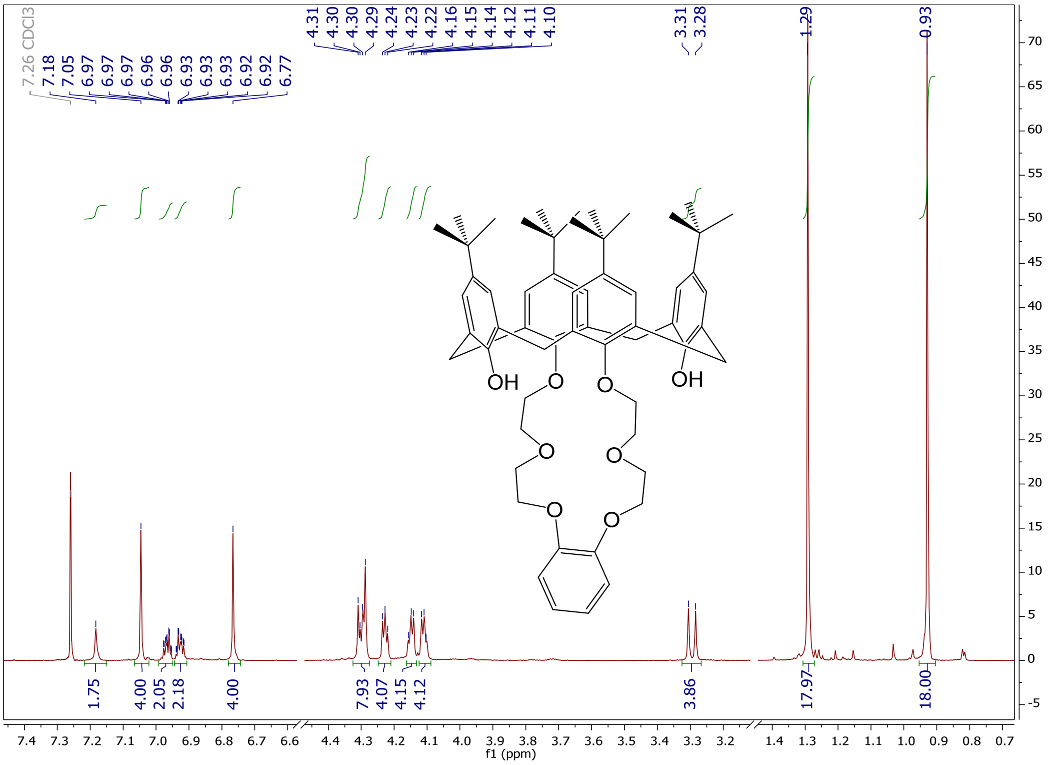

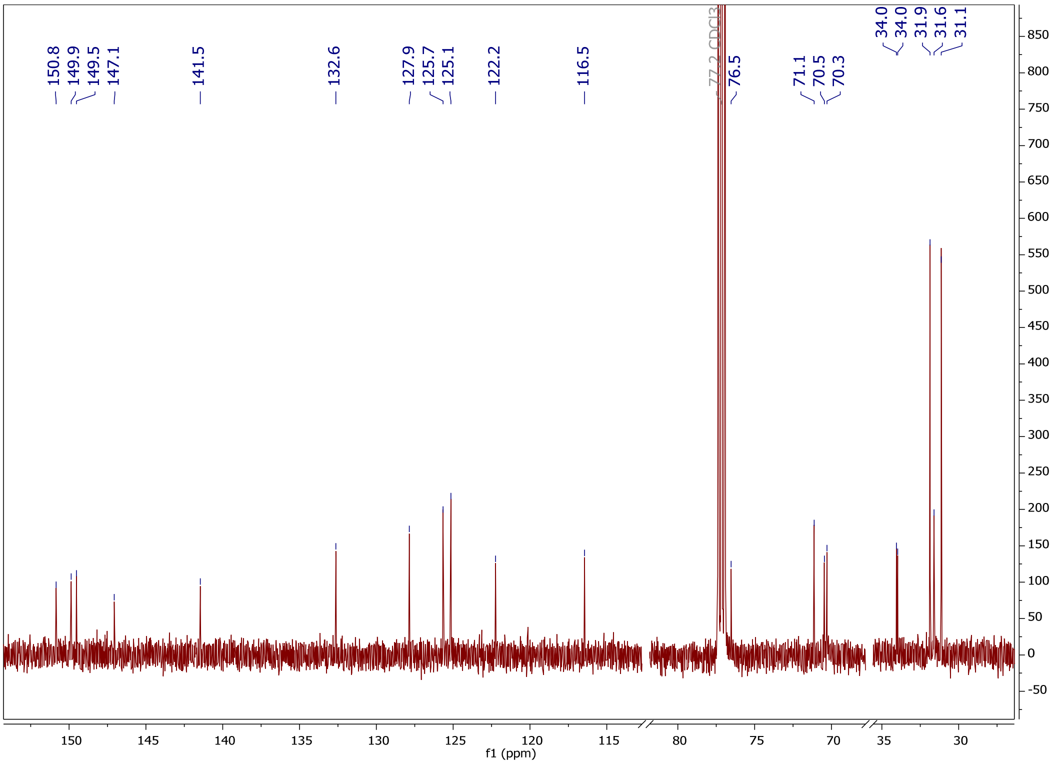

Figure S18. ^{1}H and ^{13}C NMR spectra of compound **14c**.

Compound **14d**

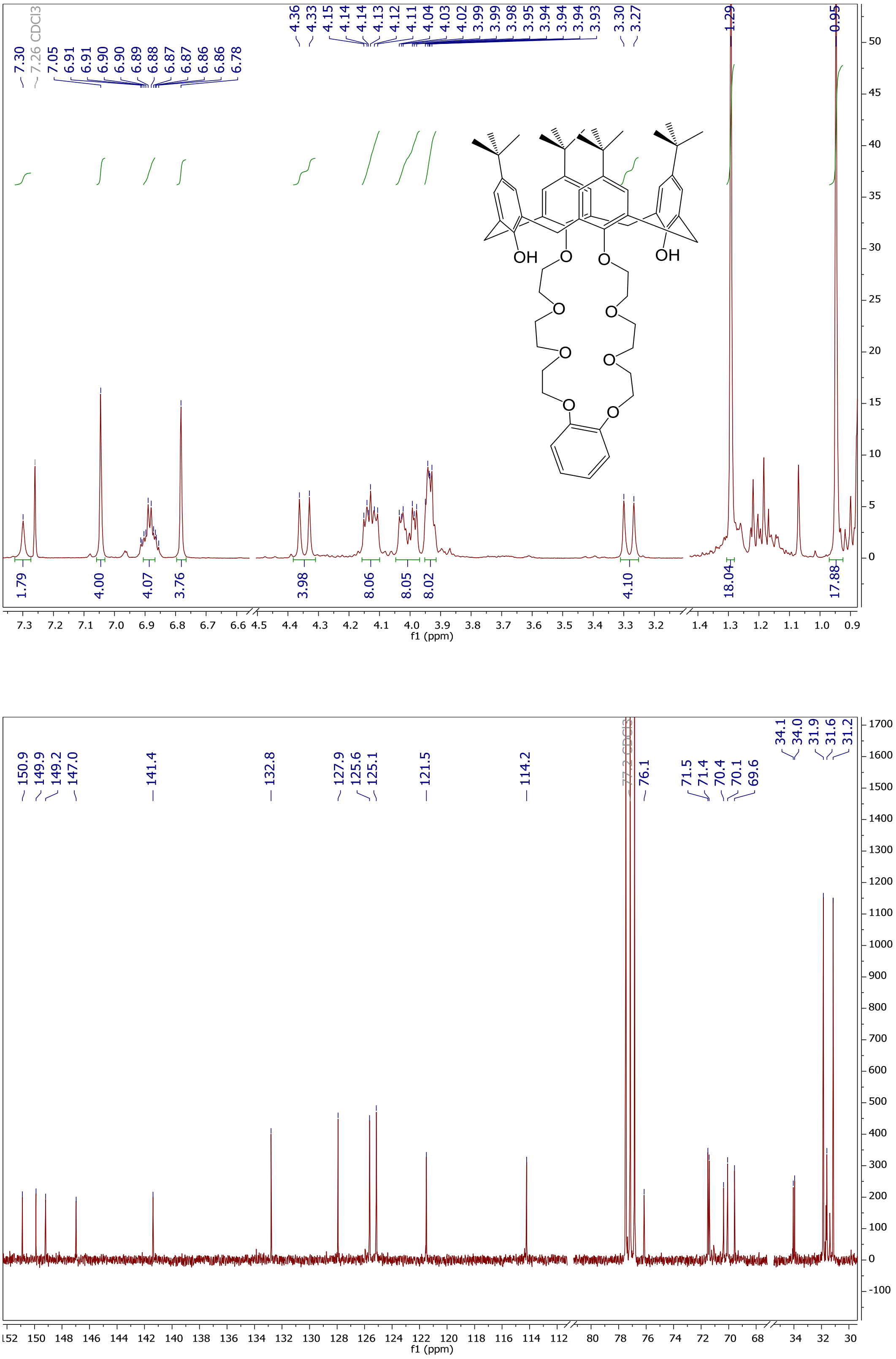

Figure S19. ^{1}H and ^{13}C NMR spectra of compound **14d**.

Compound **14e**

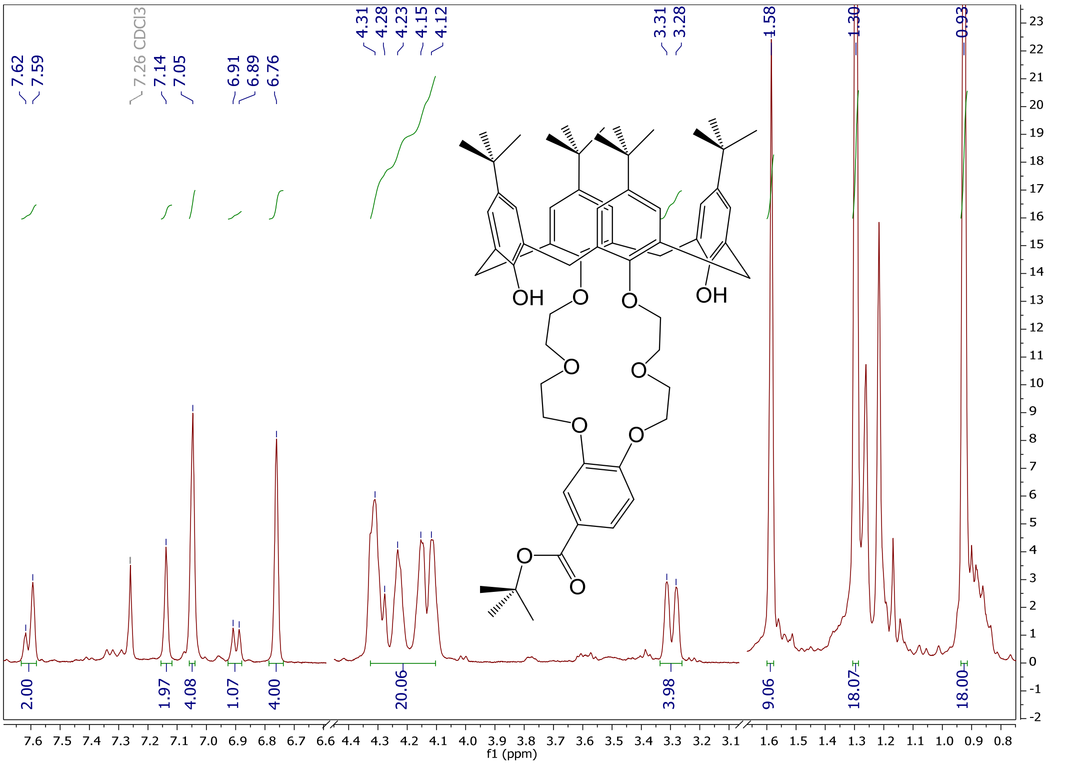

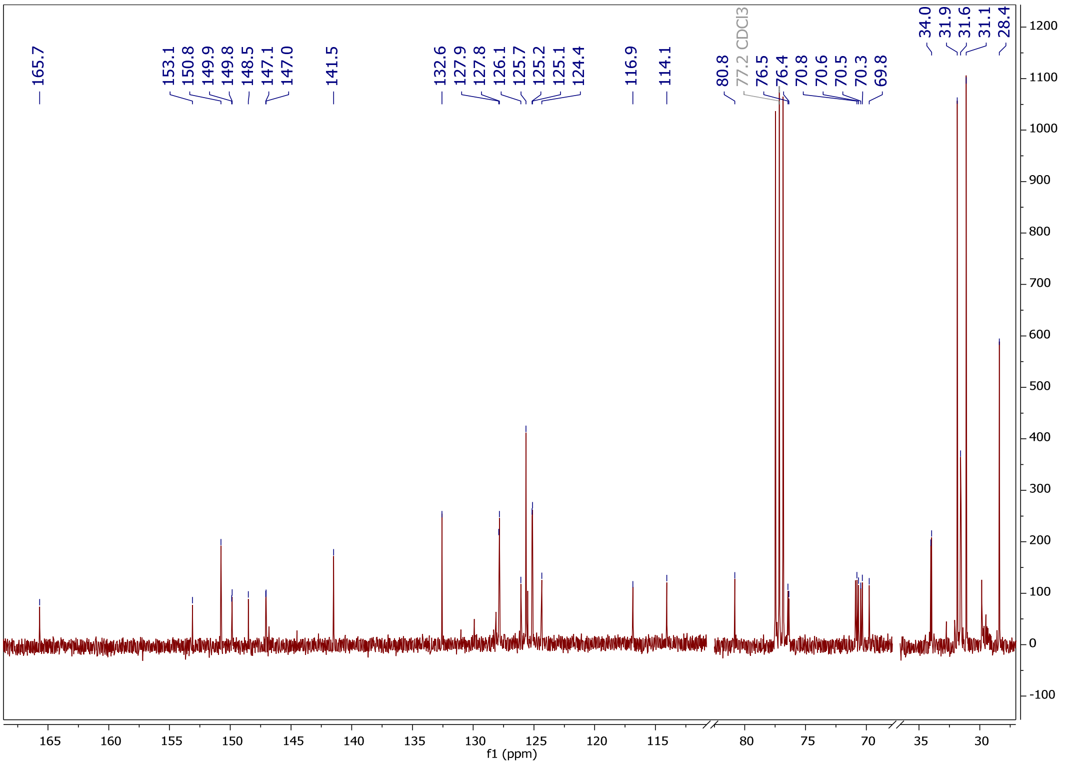

Figure S20. ^{1}H and ^{13}C NMR spectra of compound **14e**.

Compound **14f**

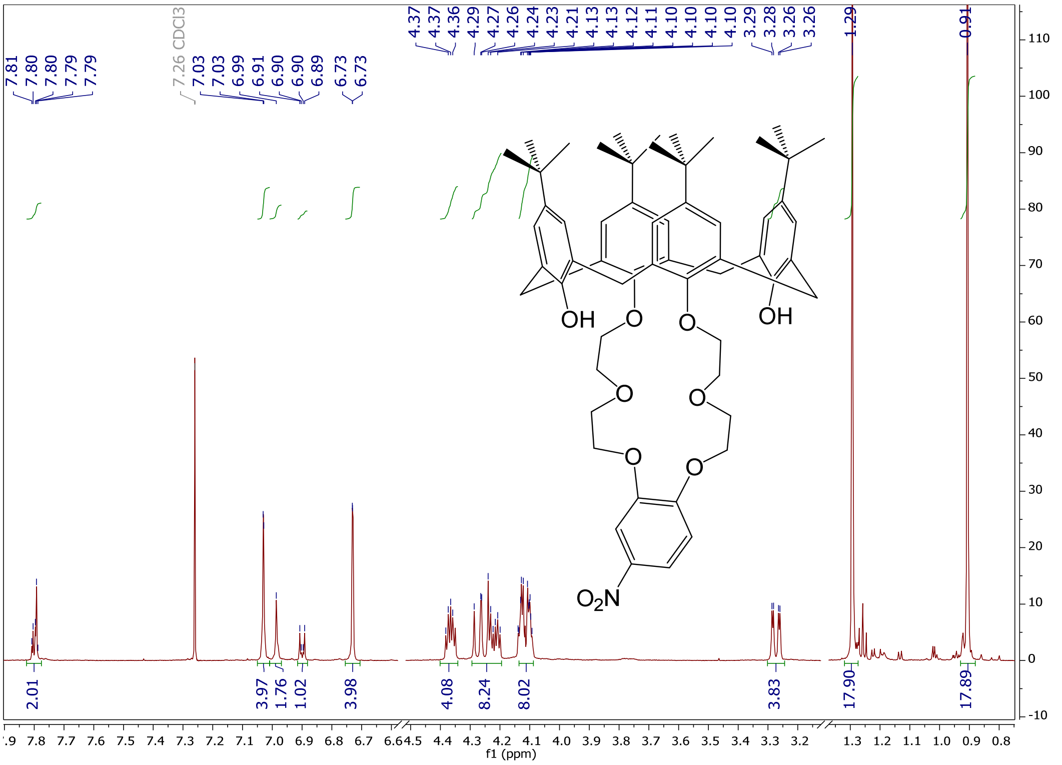

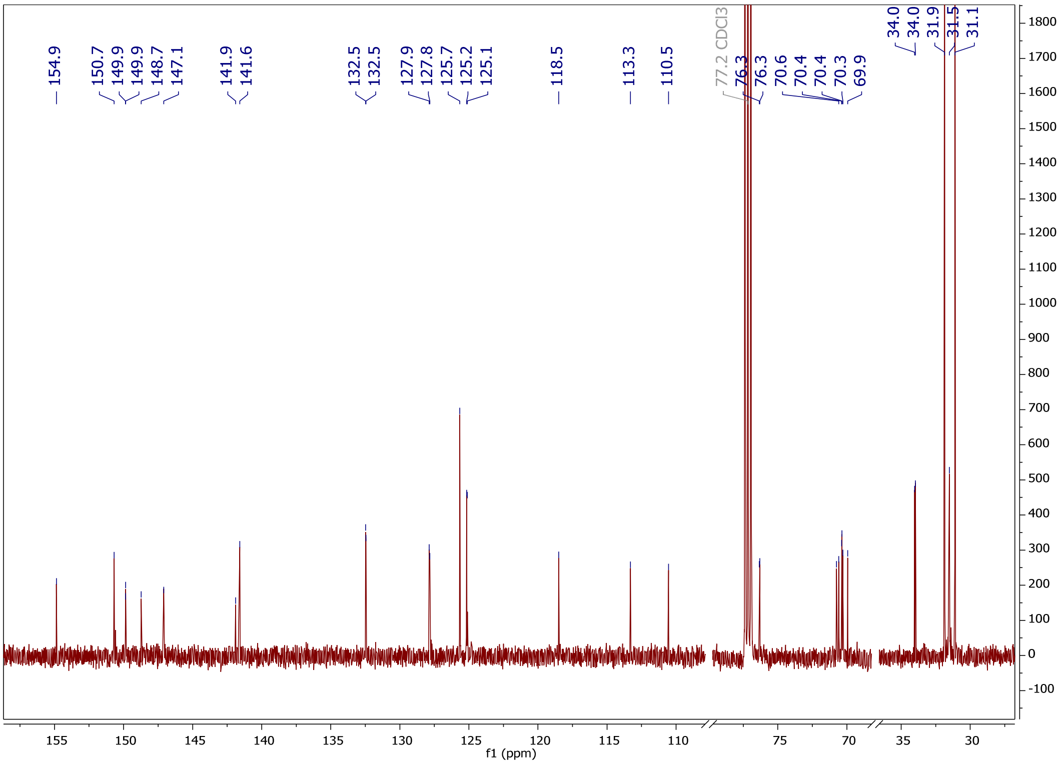

Figure S21. ^{1}H and ^{13}C NMR spectra of compound **14f**.

Compound **15b**

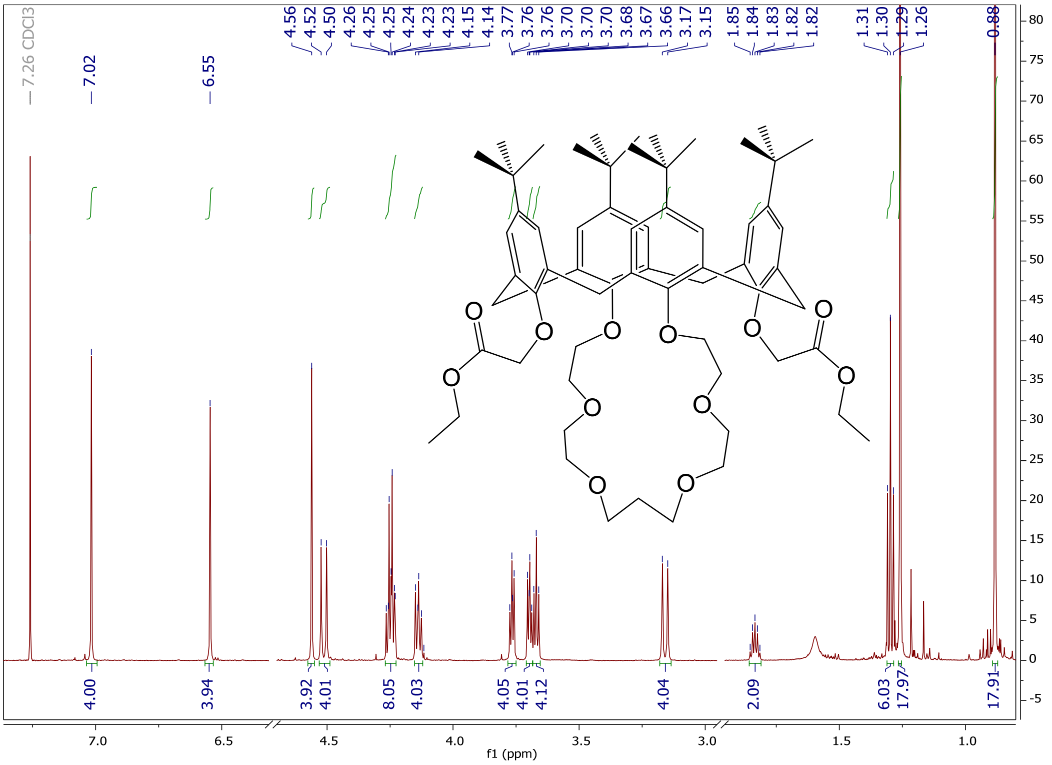

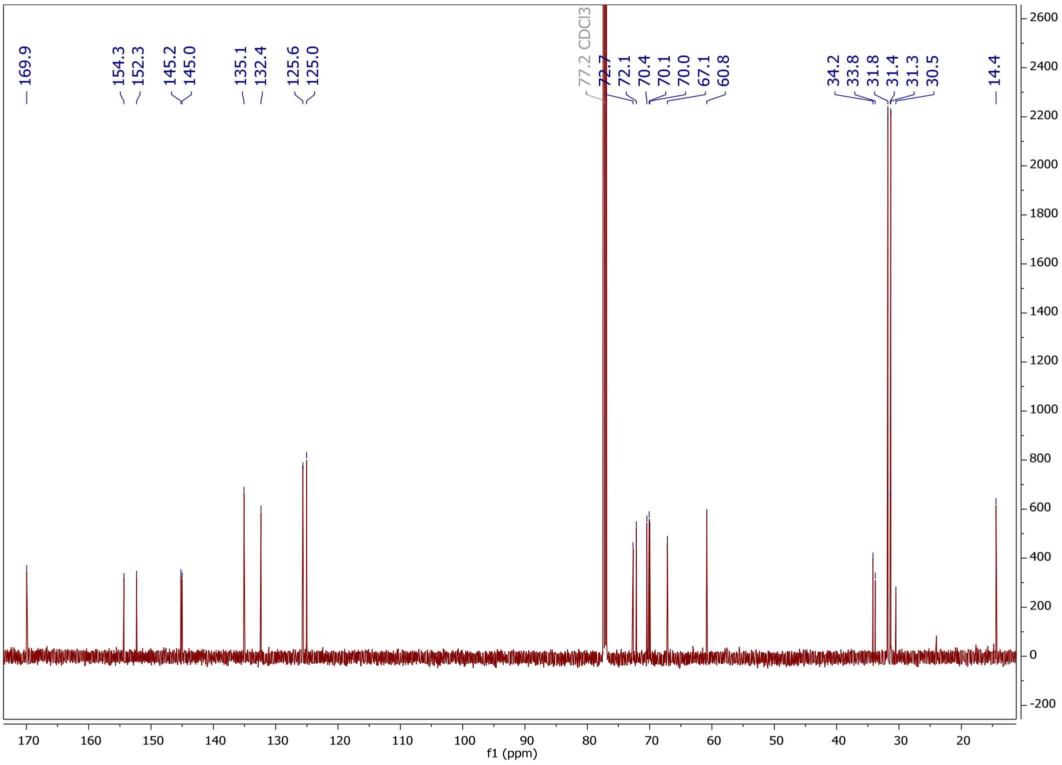

Figure S21. ^{1}H and ^{13}C NMR spectra of compound **15b**.

Compound **15c**

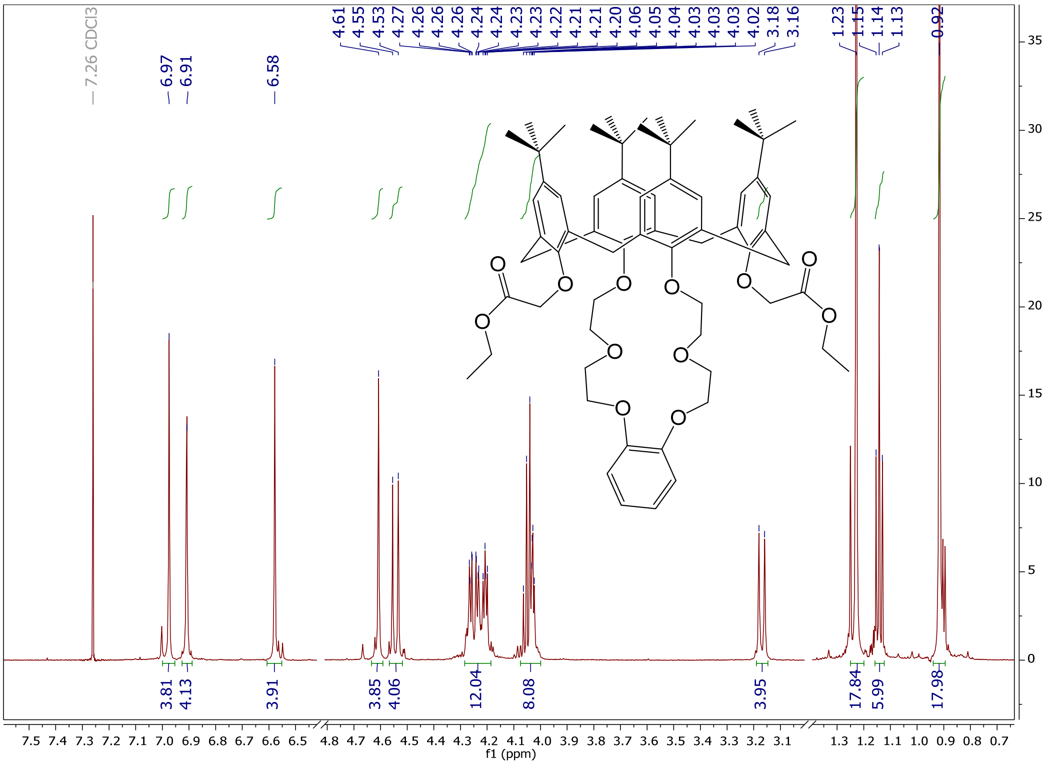

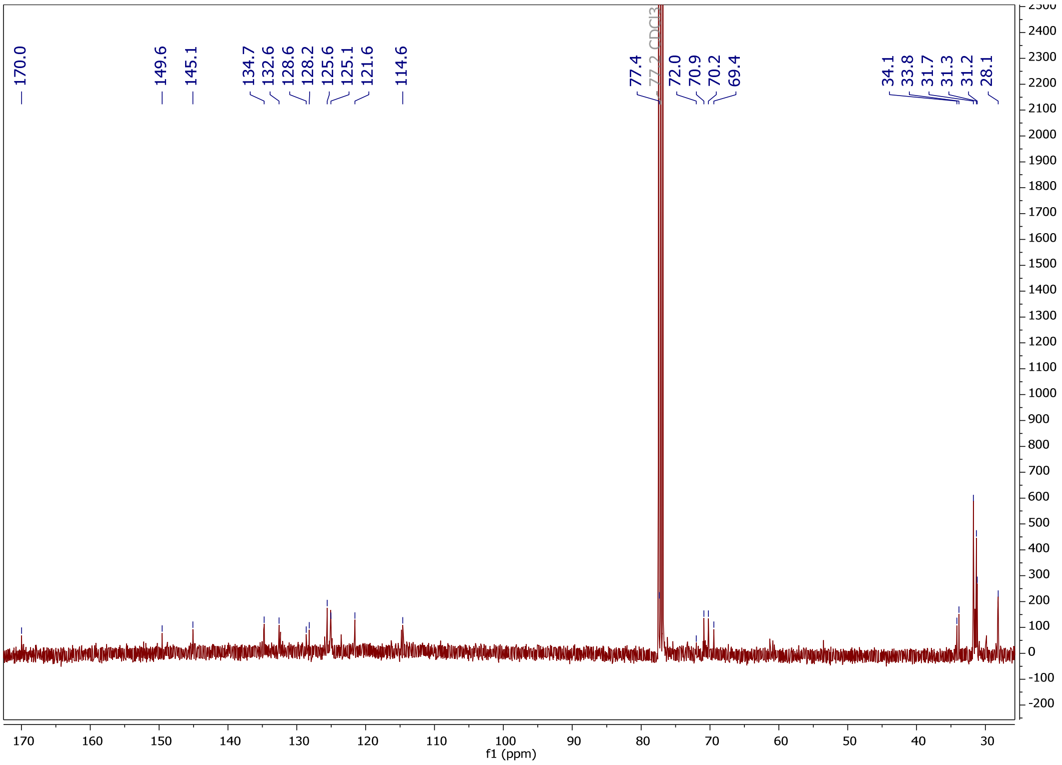

Figure S22. ^{1}H and ^{13}C NMR spectra of compound **15c**.

Compound **15d**

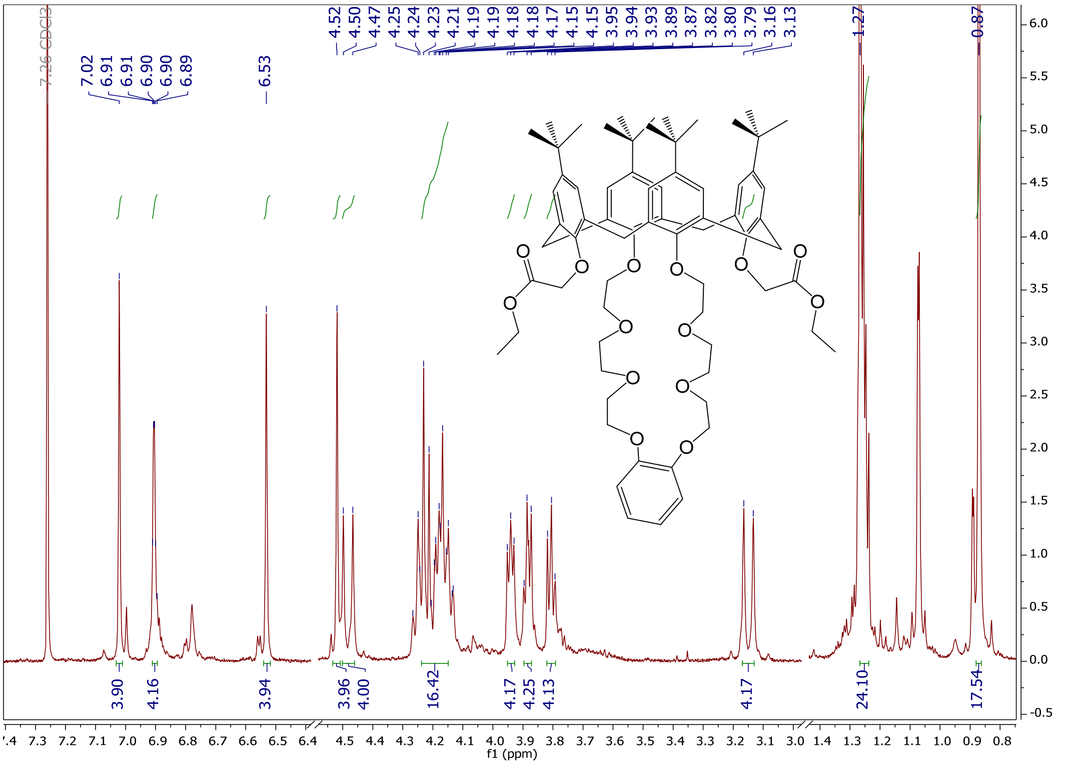

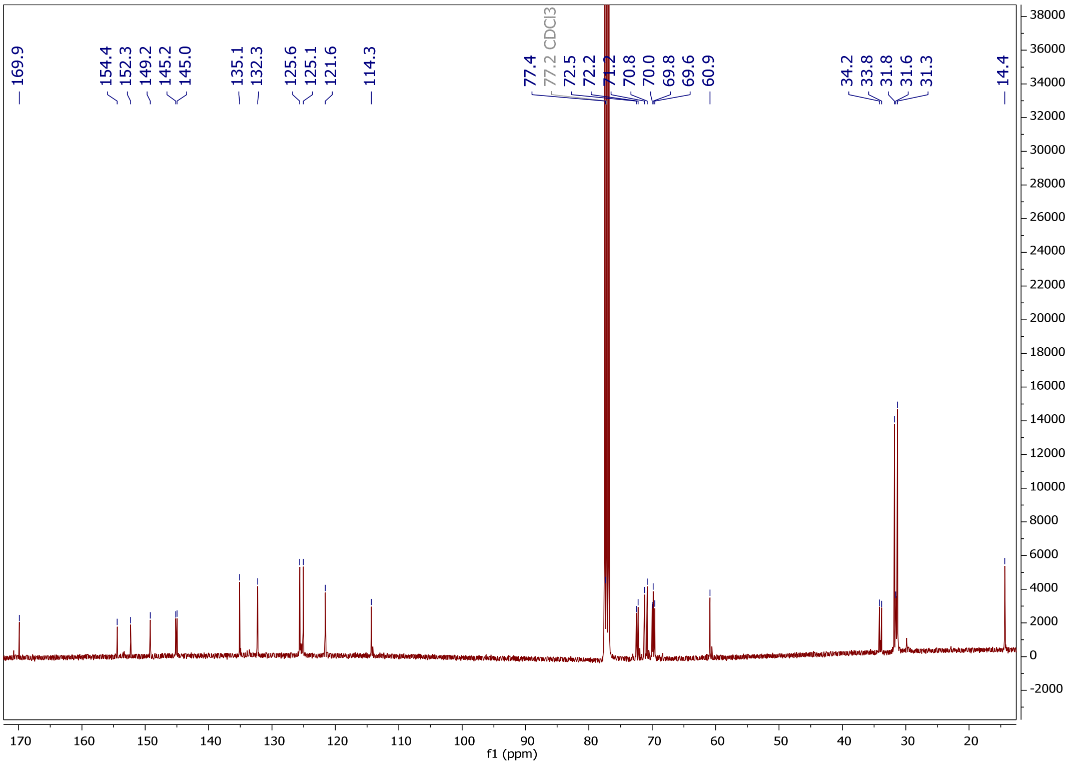

Figure S23. ^{1}H and ^{13}C NMR spectra of compound **15d**.

Compound **15e**

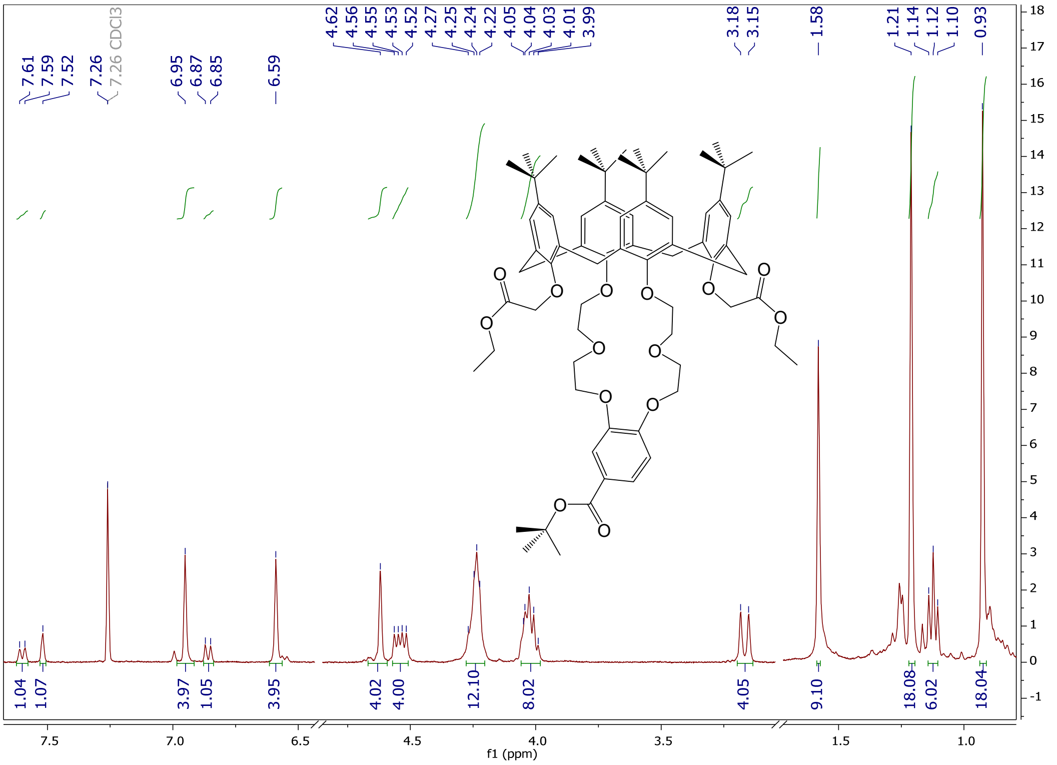

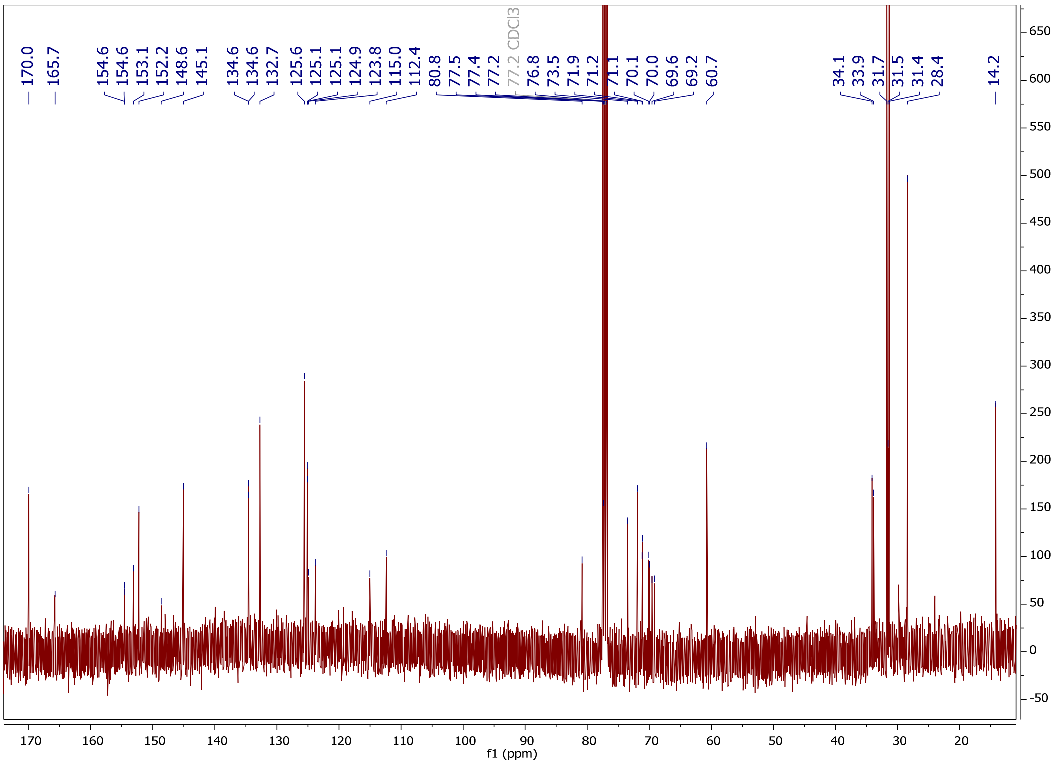

Figure S24. ^{1}H and ^{13}C NMR spectra of compound **15e**.

Compound **15f**

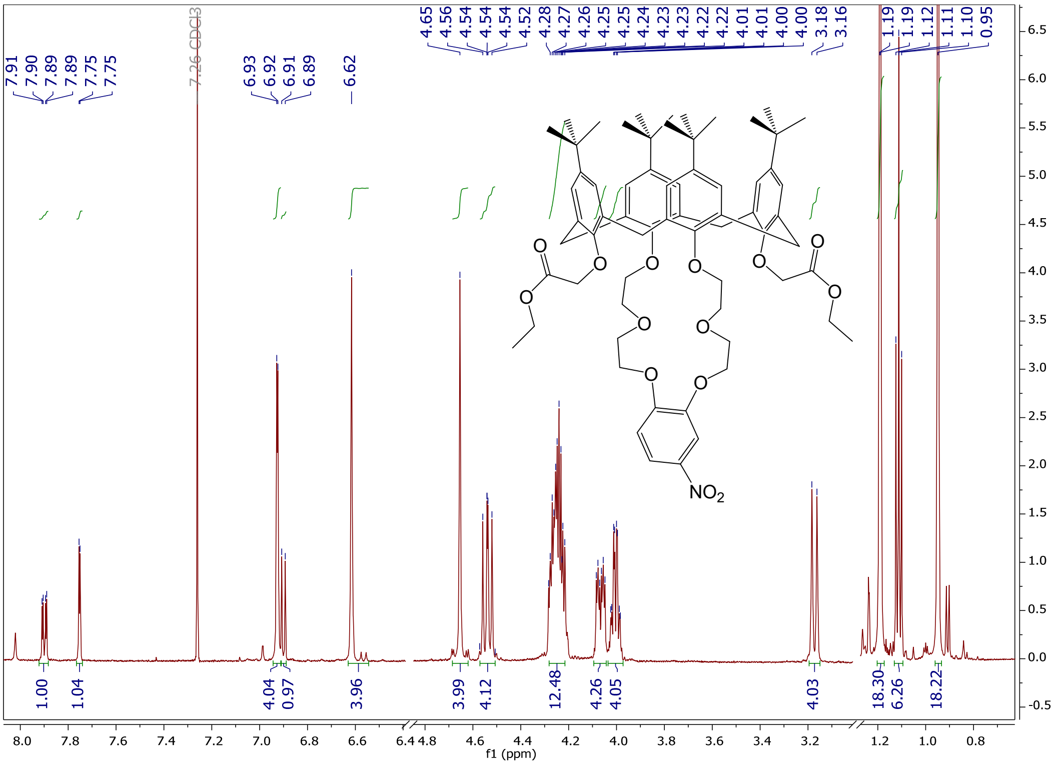

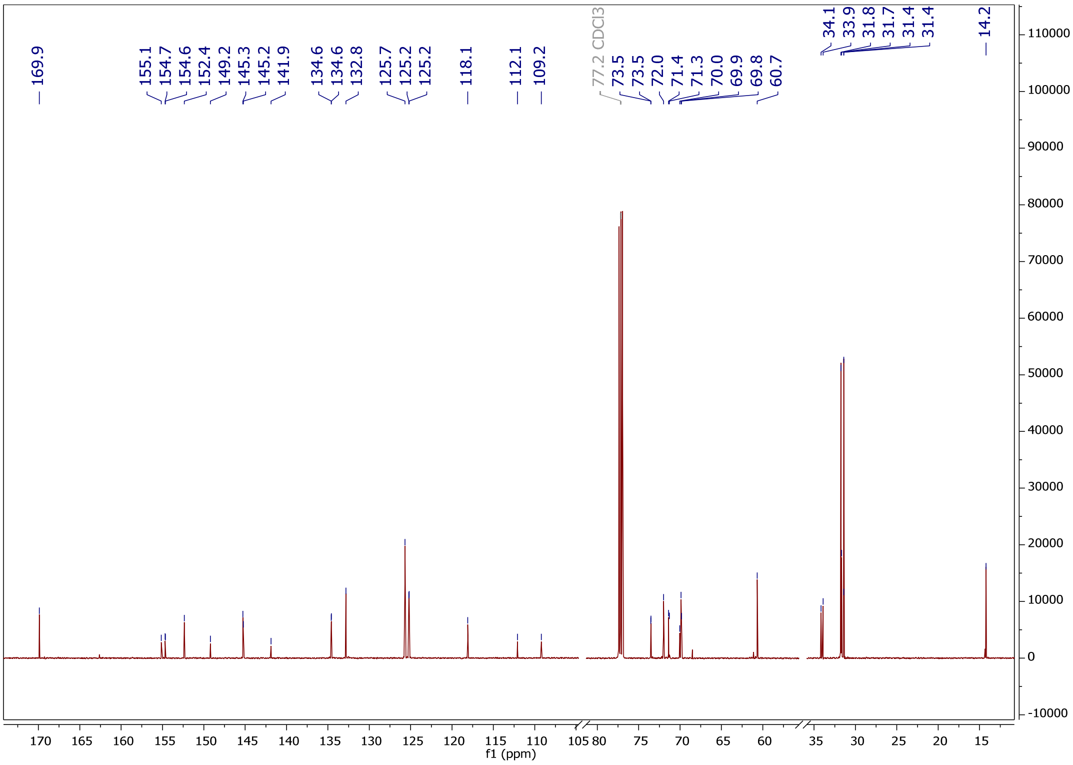

Figure S25. ^{1}H and ^{13}C NMR spectra of compound **15f**.

Compound **16b**

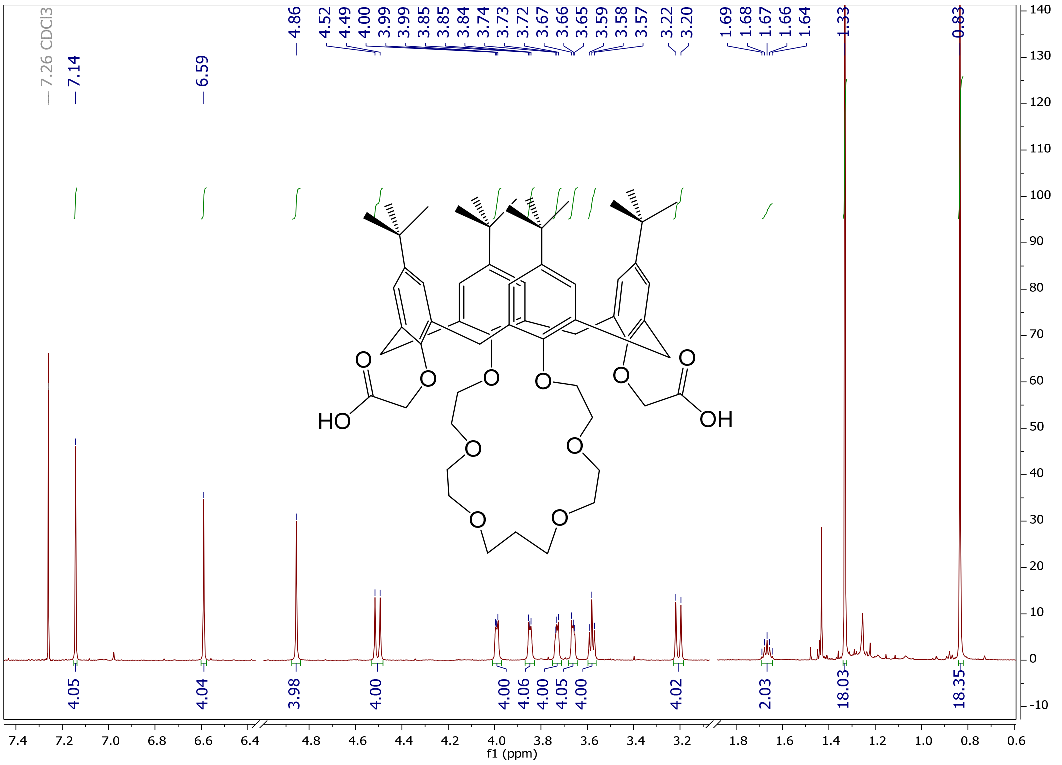

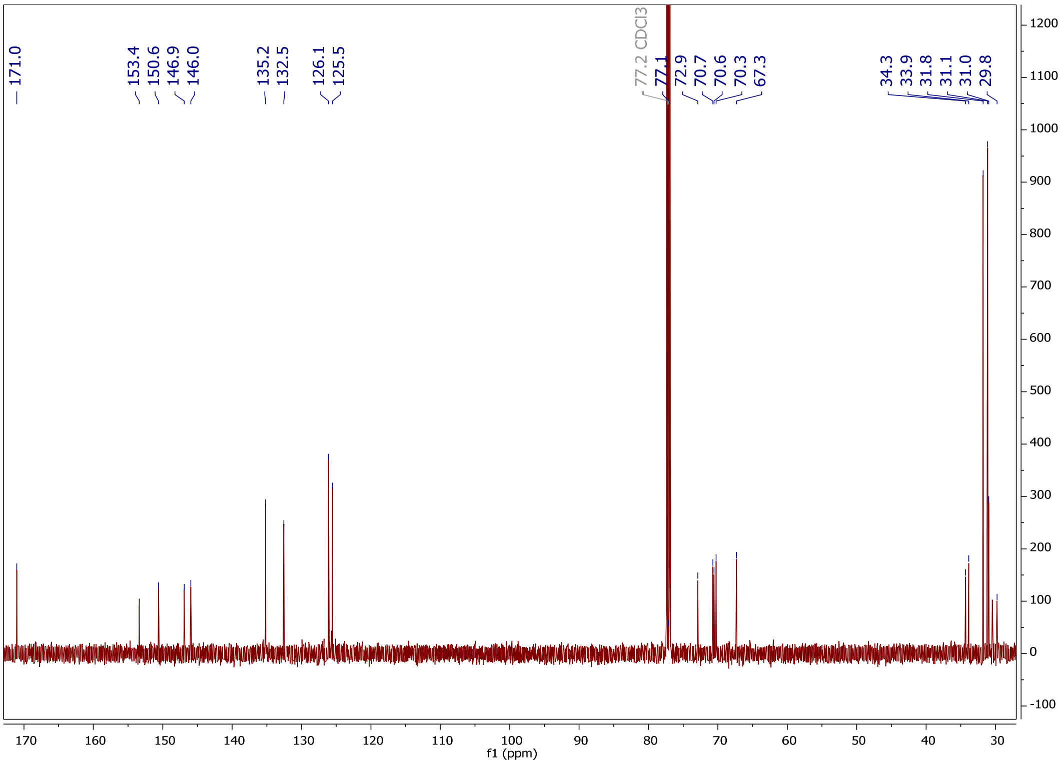

Figure S26. ^{1}H and ^{13}C NMR spectra of compound **16b**.

Compound **16c**

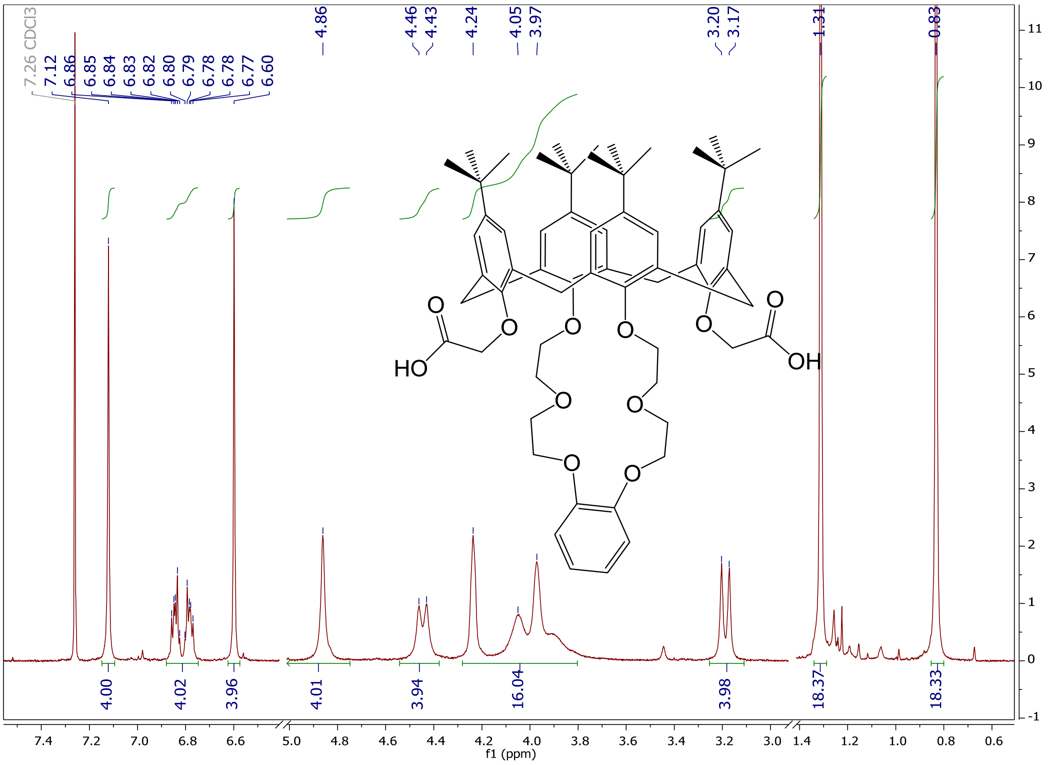

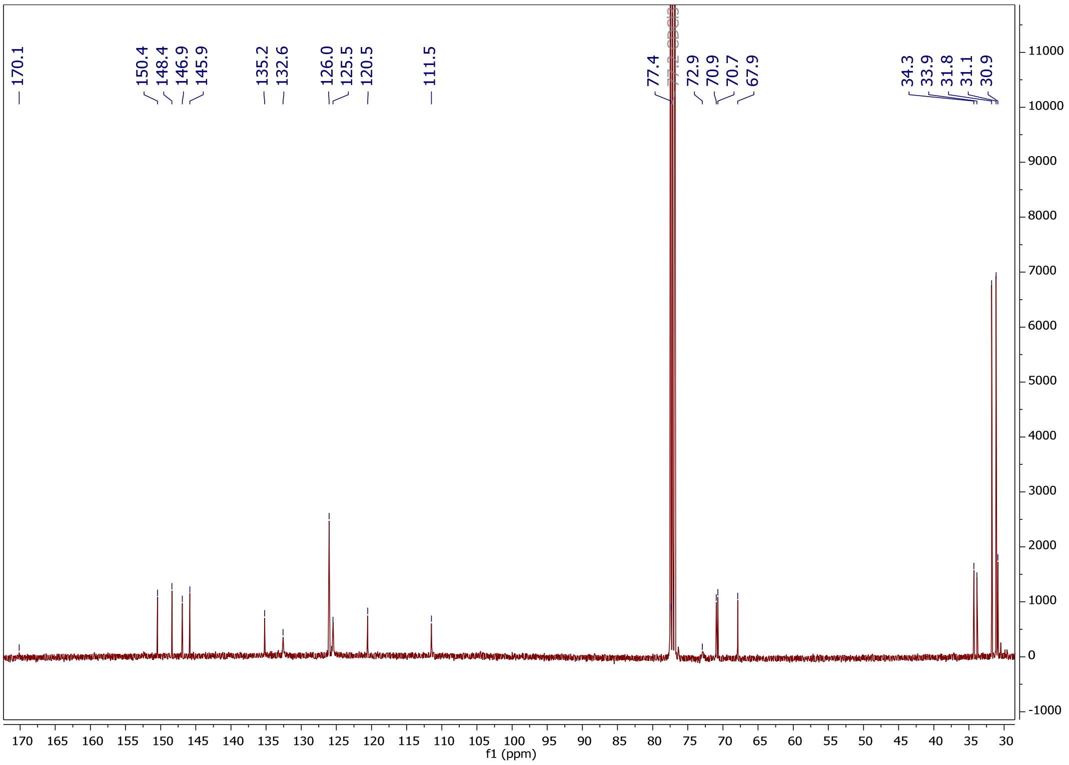

Figure S27. ^{1}H and ^{13}C NMR spectra of compound **16c**.

Compound **16d**

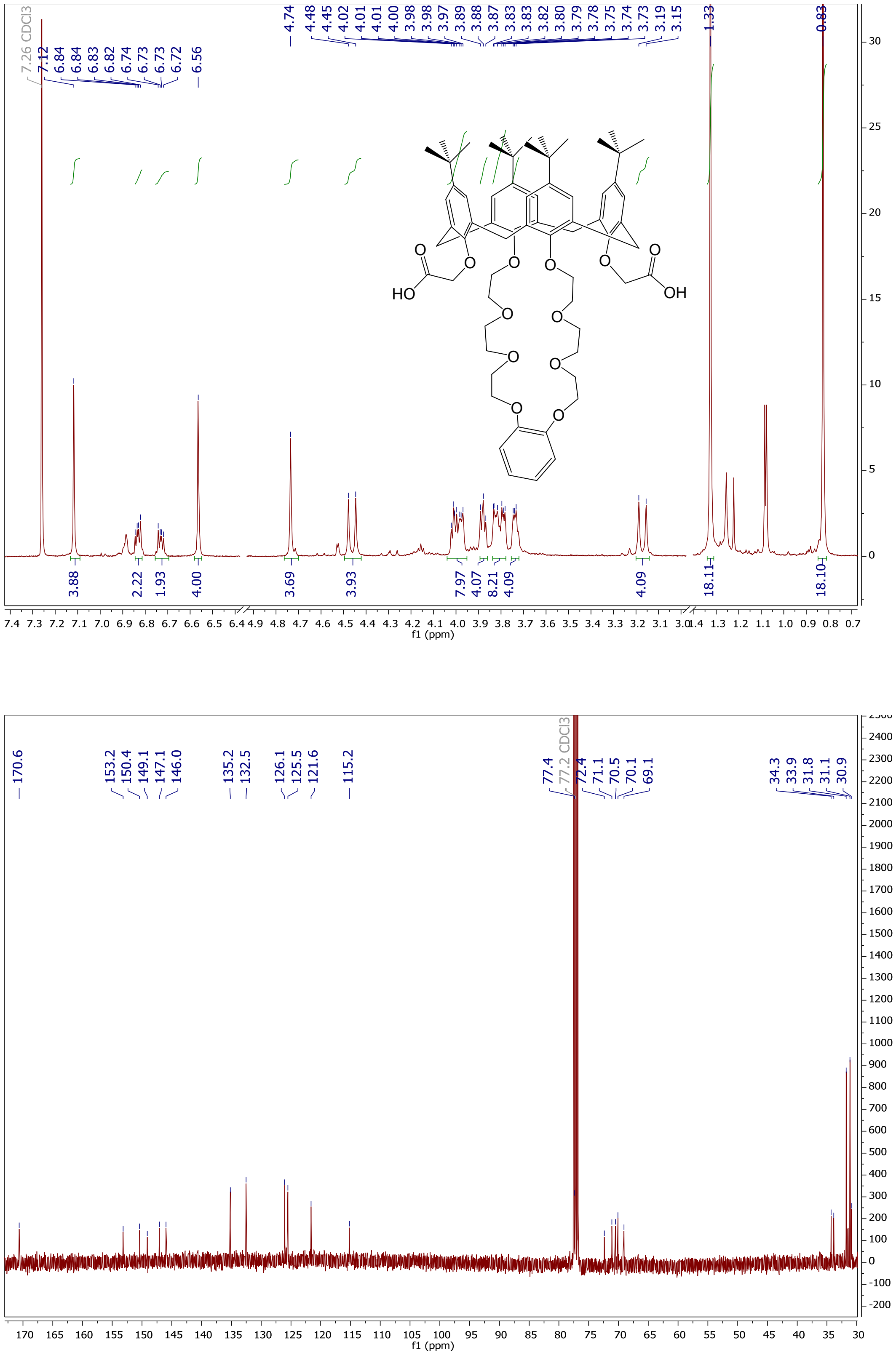

Figure S27. ^{1}H and ^{13}C NMR spectra of compound **16d**.

Compound **16e**

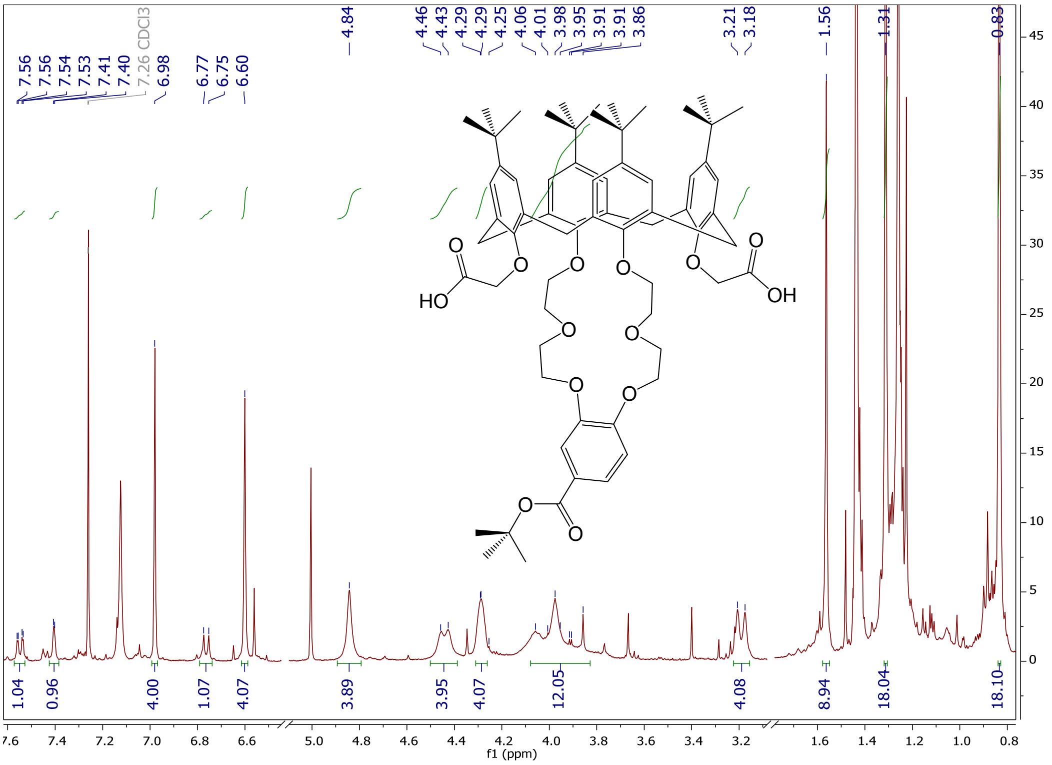

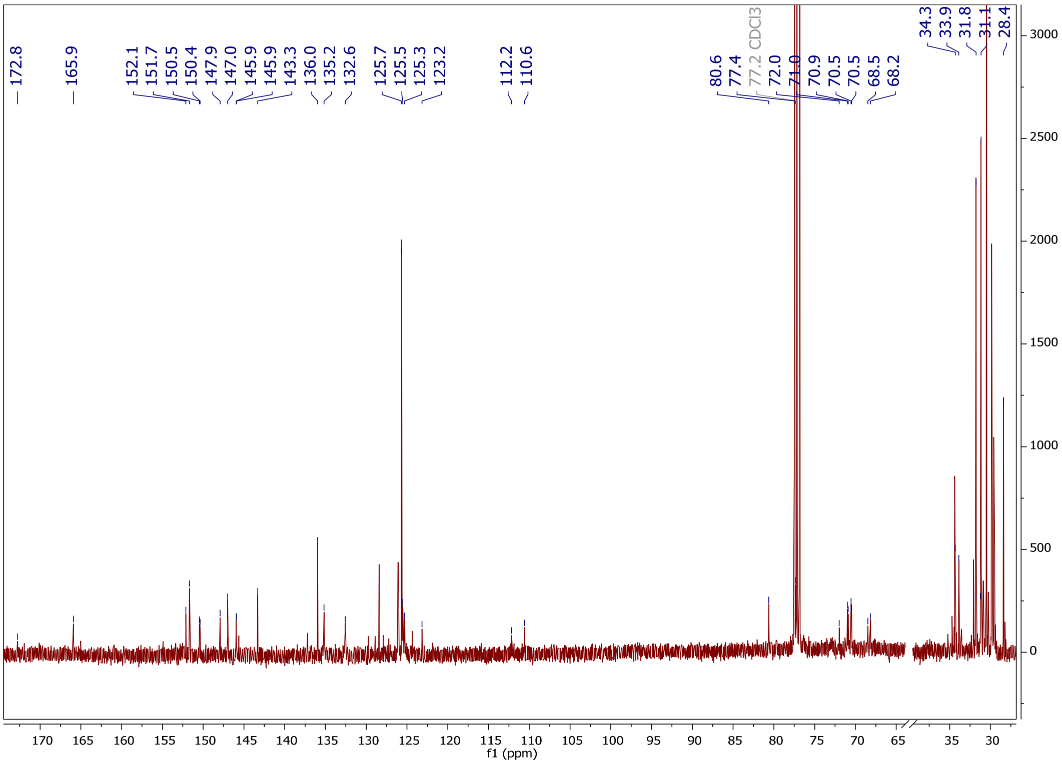

Figure S28. ^{1}H and ^{13}C NMR spectra of compound **16e**.

Compound **22**

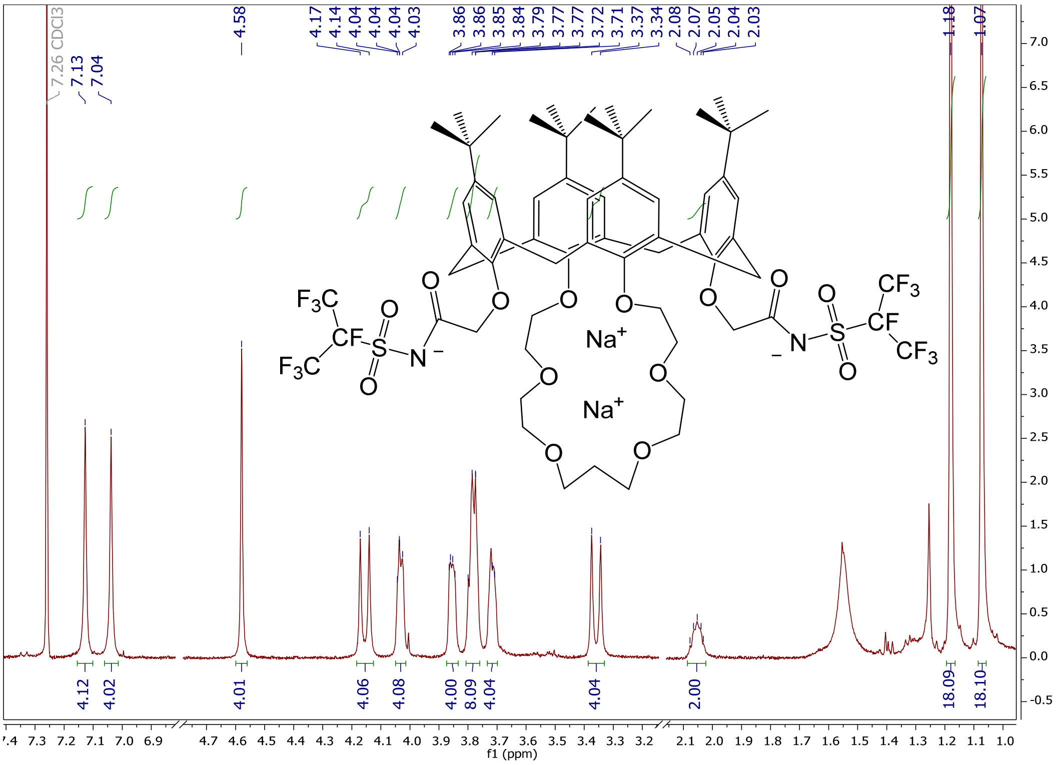

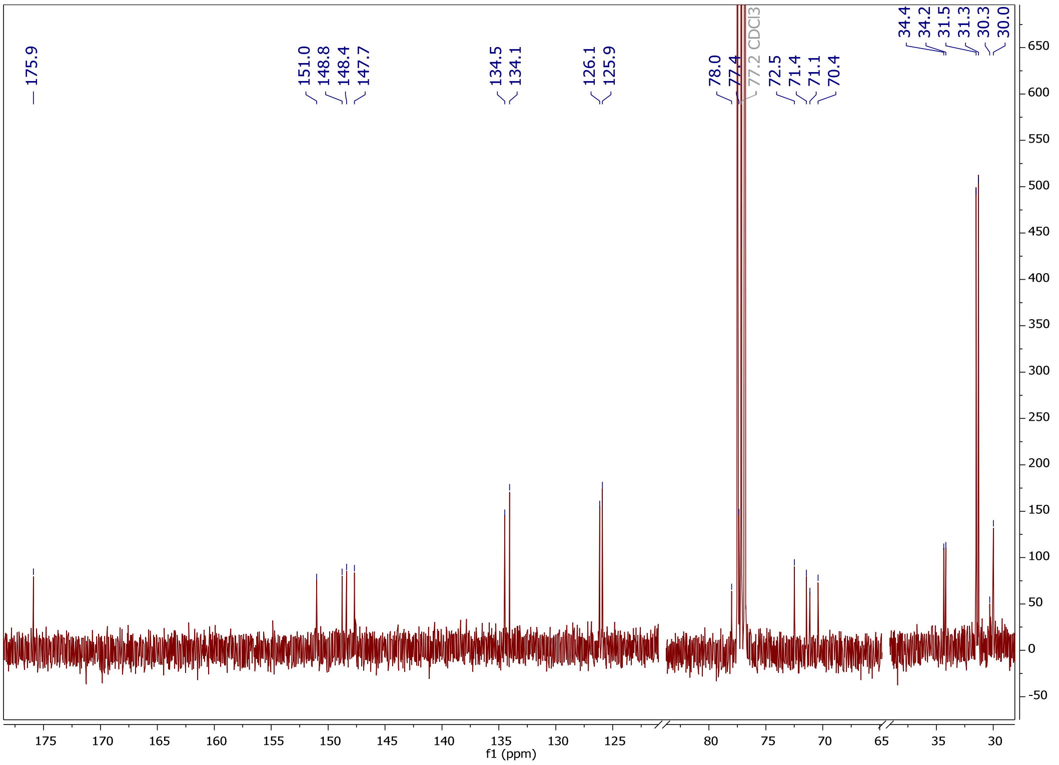

Figure S29. ^{1}H and ^{13}C NMR spectra of compound **22**.

Compound **23**

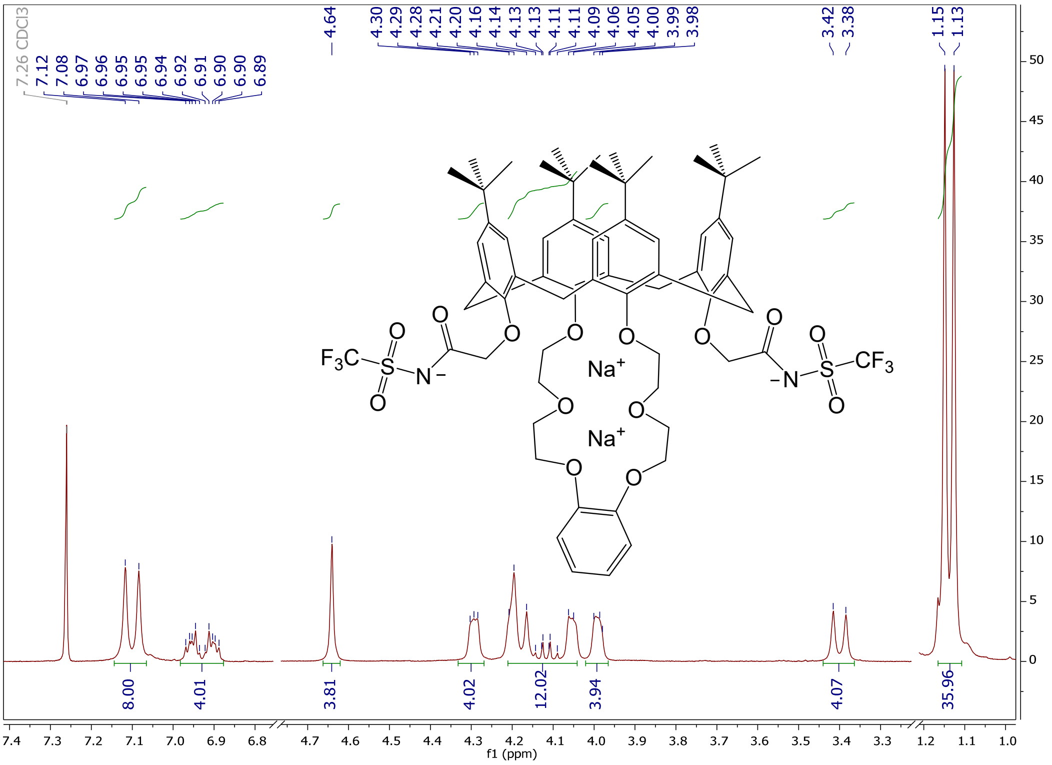

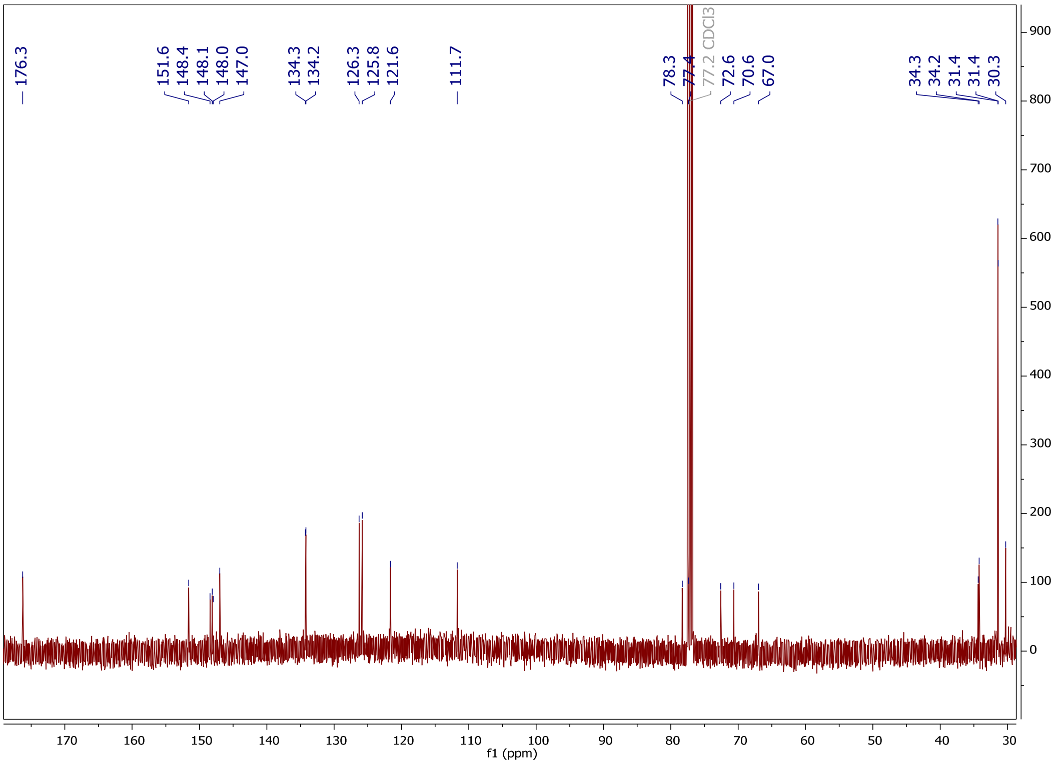

Figure S30. ^{1}H and ^{13}C NMR spectra of compound **23**.

Compound **24**

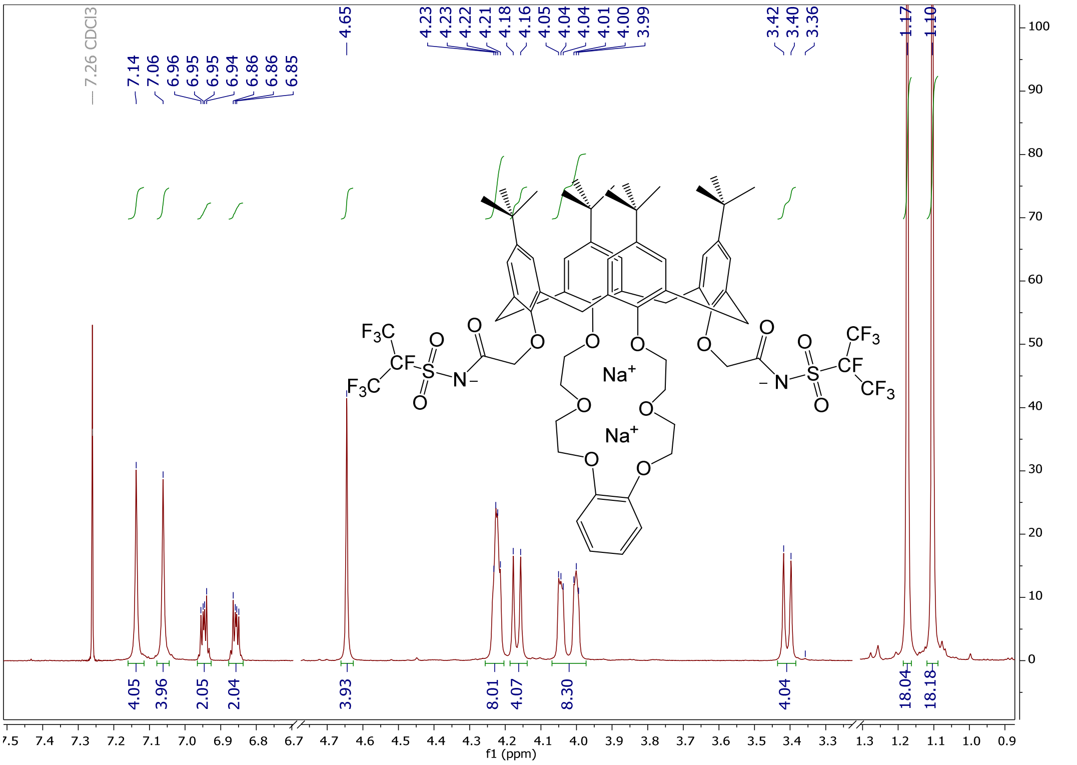

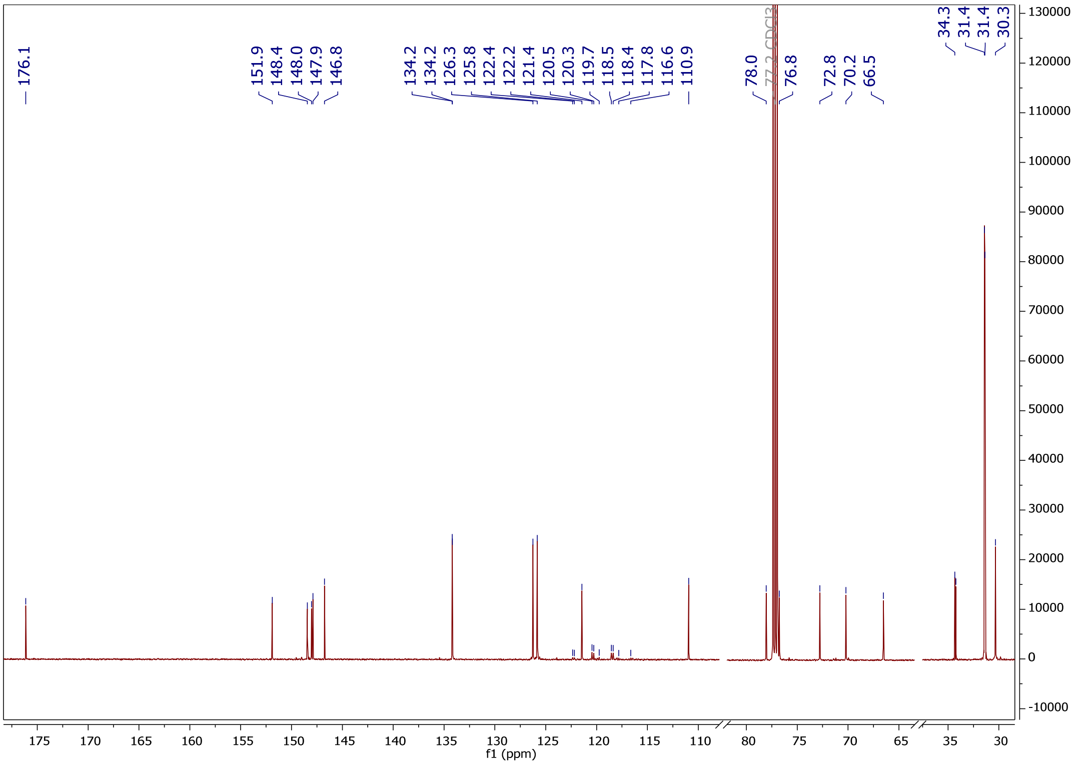

Figure S31. ^{1}H and ^{13}C NMR spectra of compound **24**.

Compound **25**

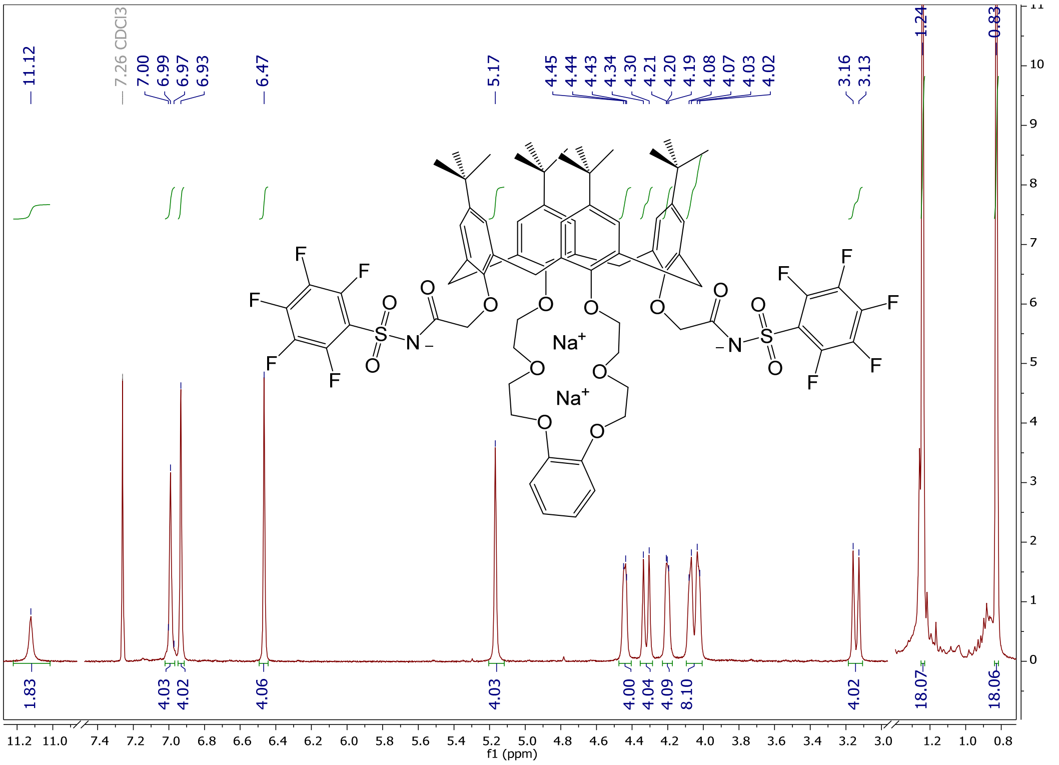

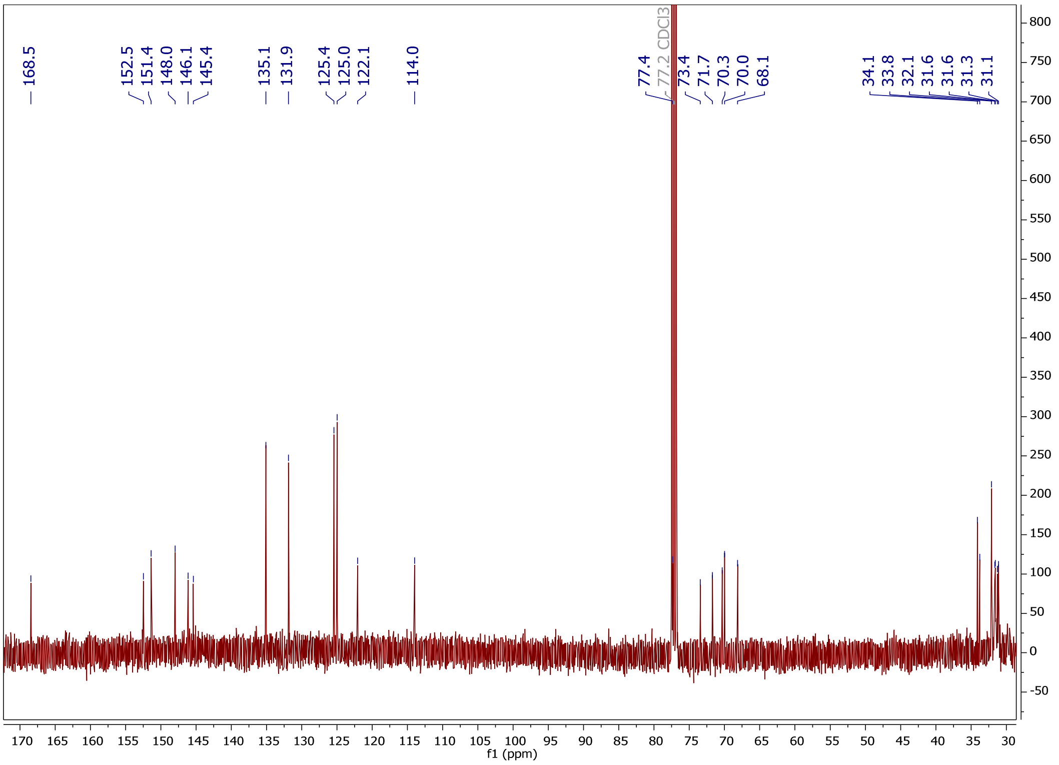

Figure S32. ^{1}H and ^{13}C NMR spectra of compound **25**.

Compound **26**

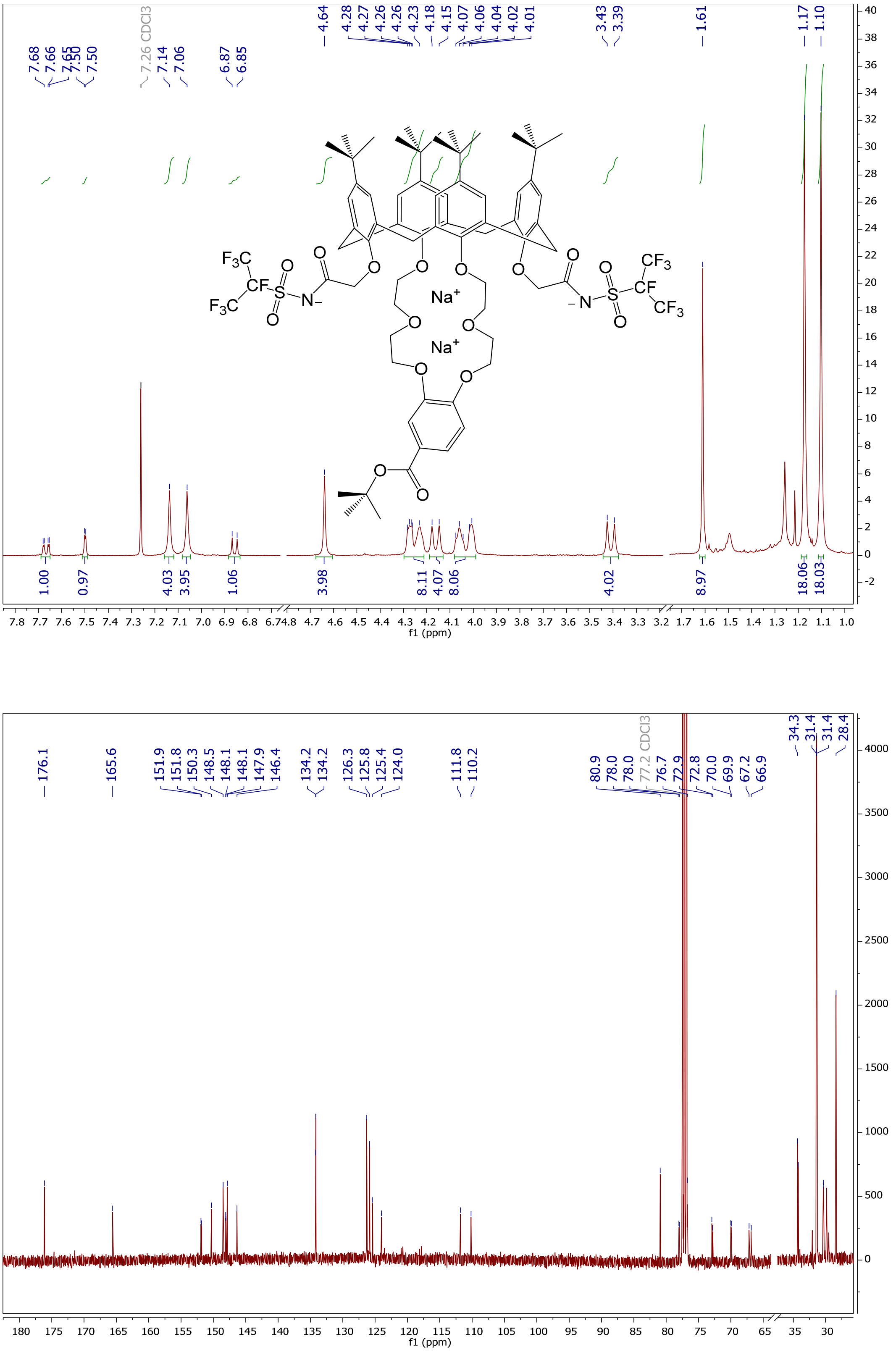

Figure S33. ^{1}H and ^{13}C NMR spectra of compound **26**.

Compound **27**

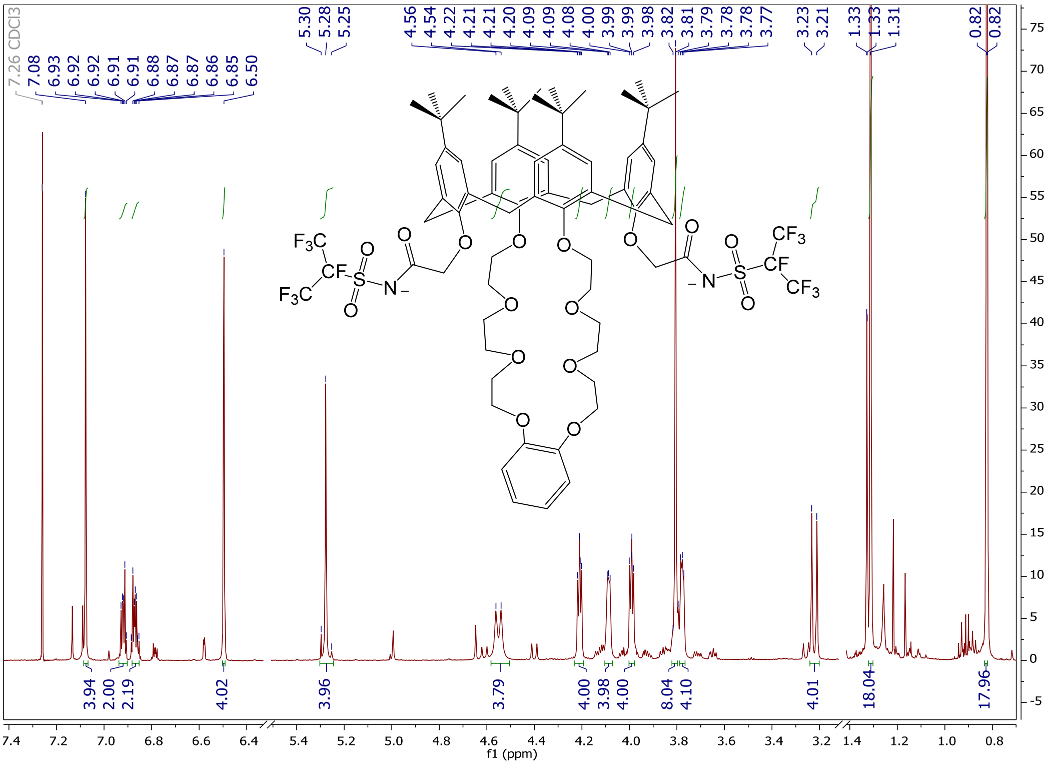

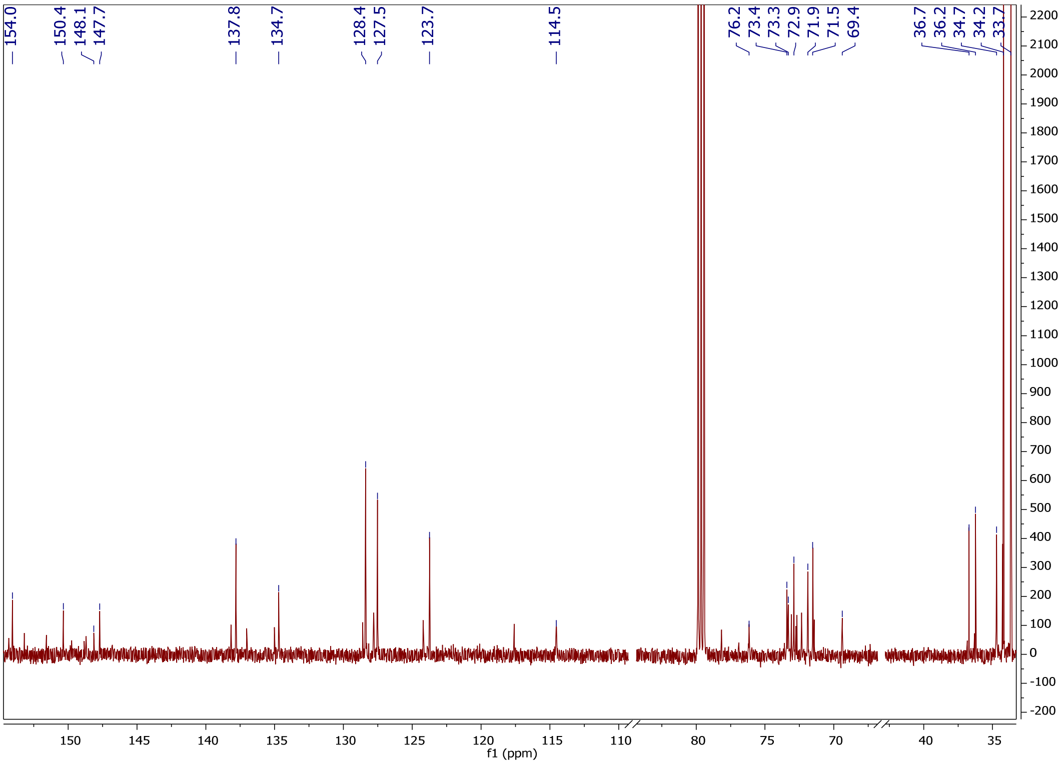

Figure S34. ^{1}H and ^{13}C NMR spectra of compound **27**.

UV spectra and titration plots of final compounds

Compound **5** with Ba^{2+}

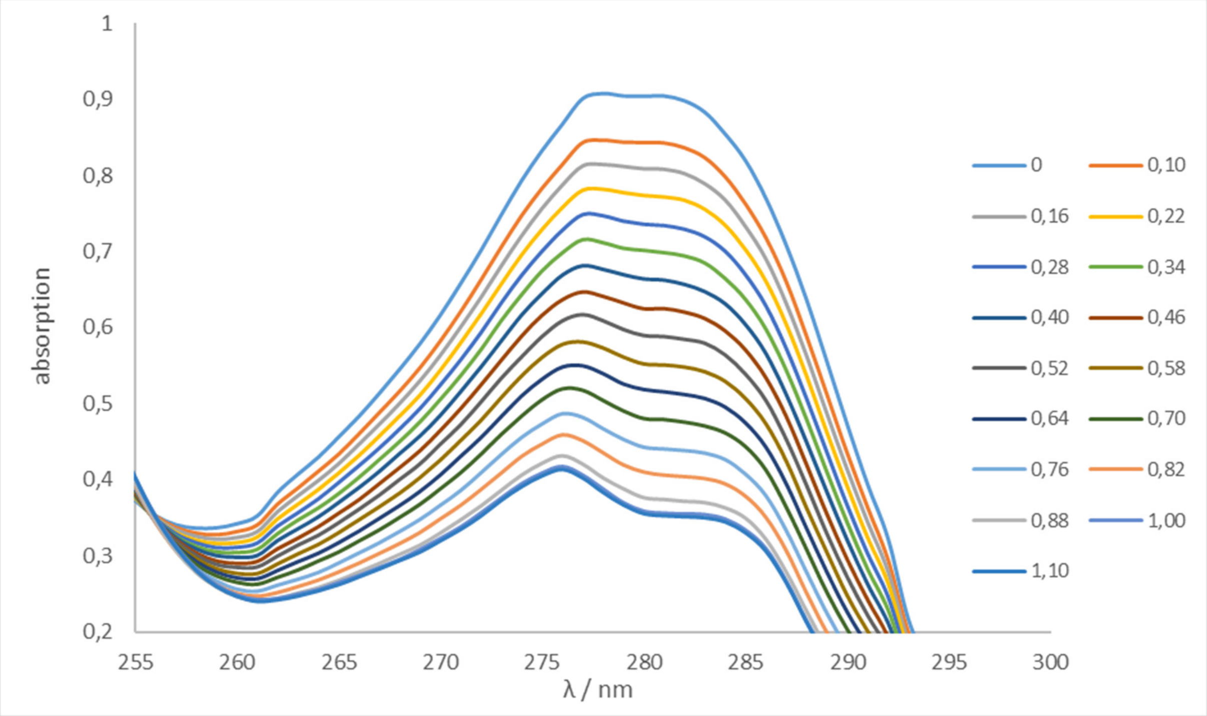

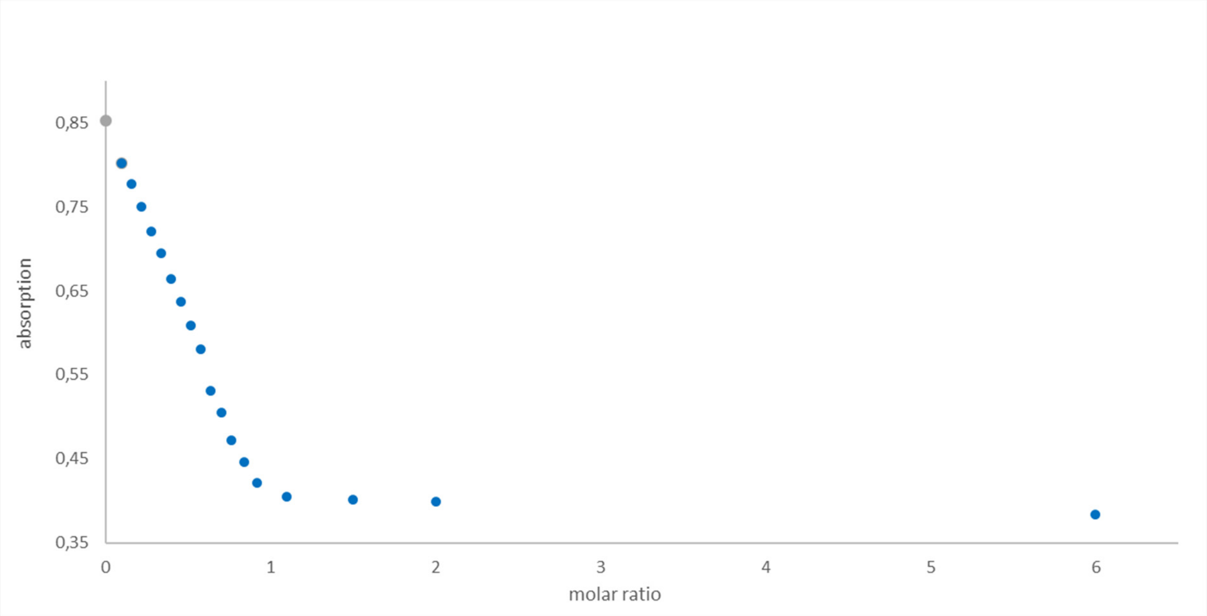

Figure S35. UV spectra and UV titration plot of compound **5** with Ba^{2+}.

Compound **5** with Sr^{2+}

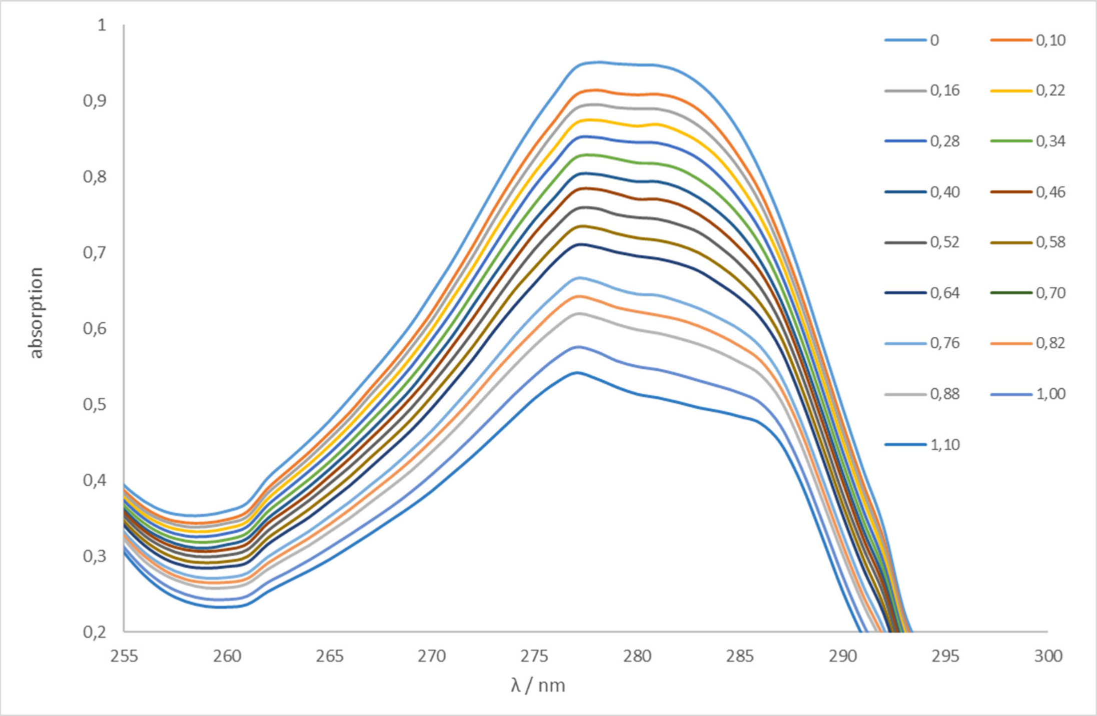

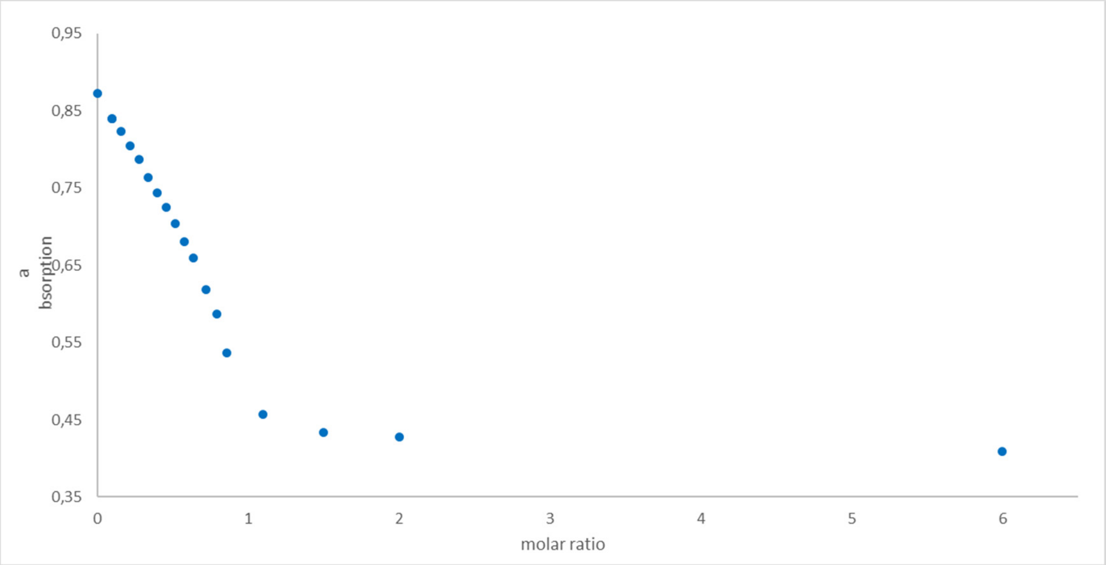

Figure S36. UV spectra and UV titration plot of compound **5** with Sr^{2+}.

Compound **5** with Pb^{2+}

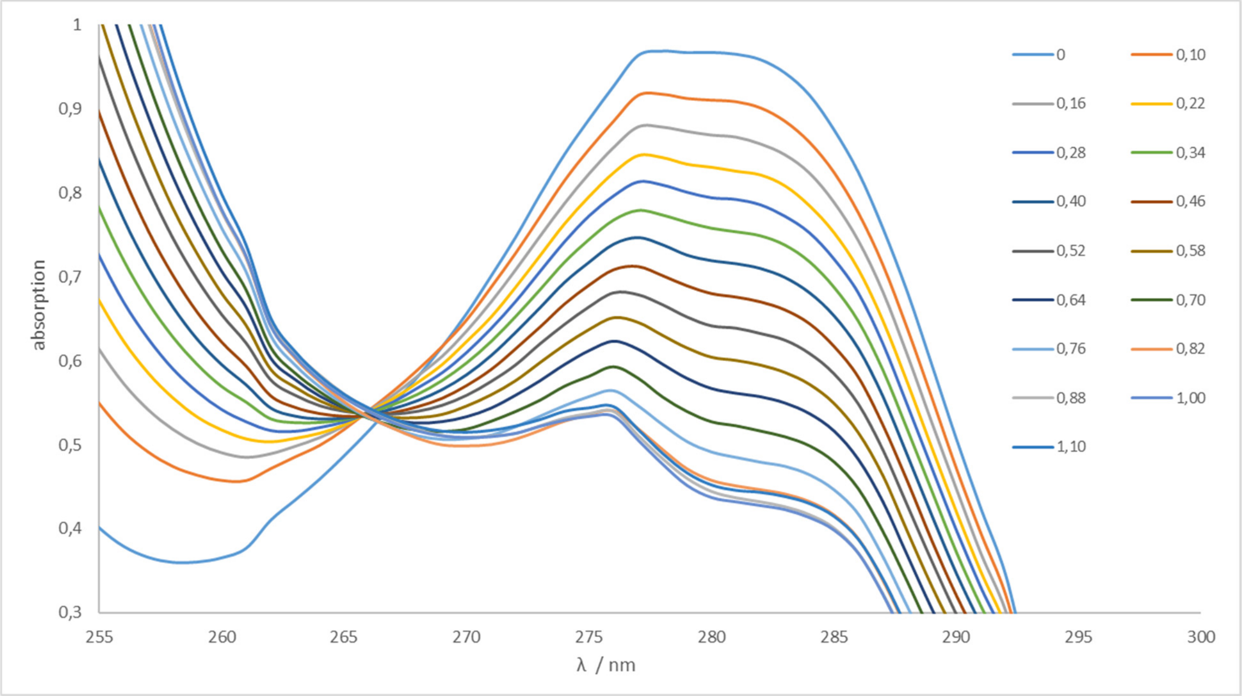

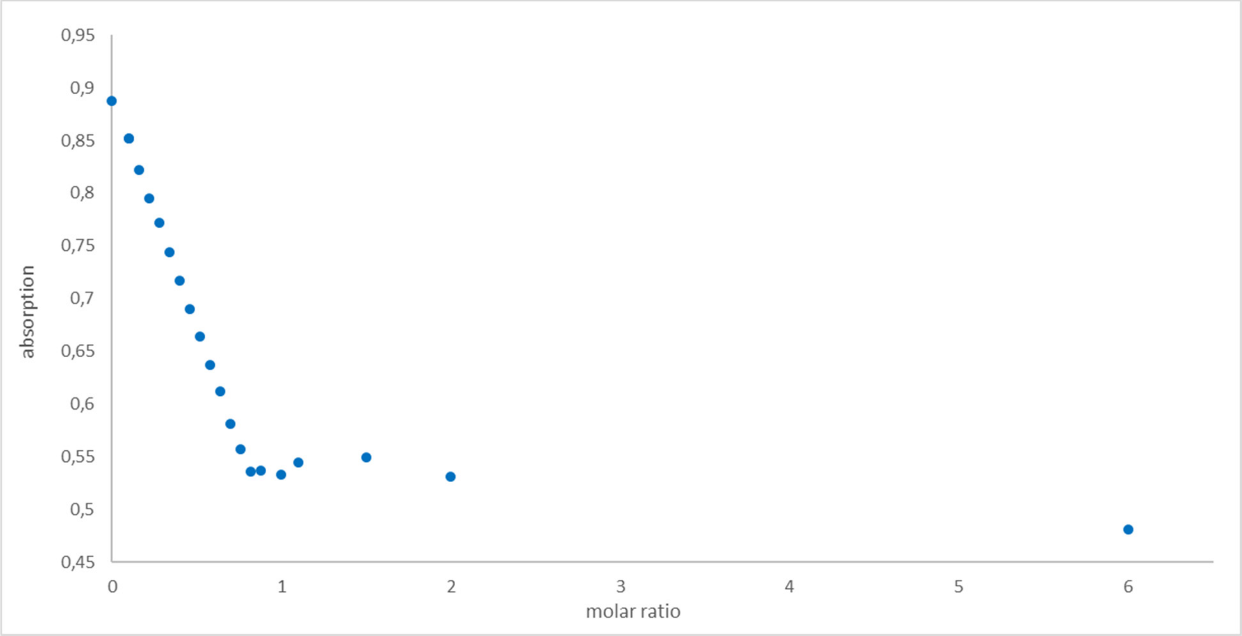

Figure S37. UV spectra and UV titration plot of compound **5** with Pb^{2+}.

Compound **6** with Ba^{2+}

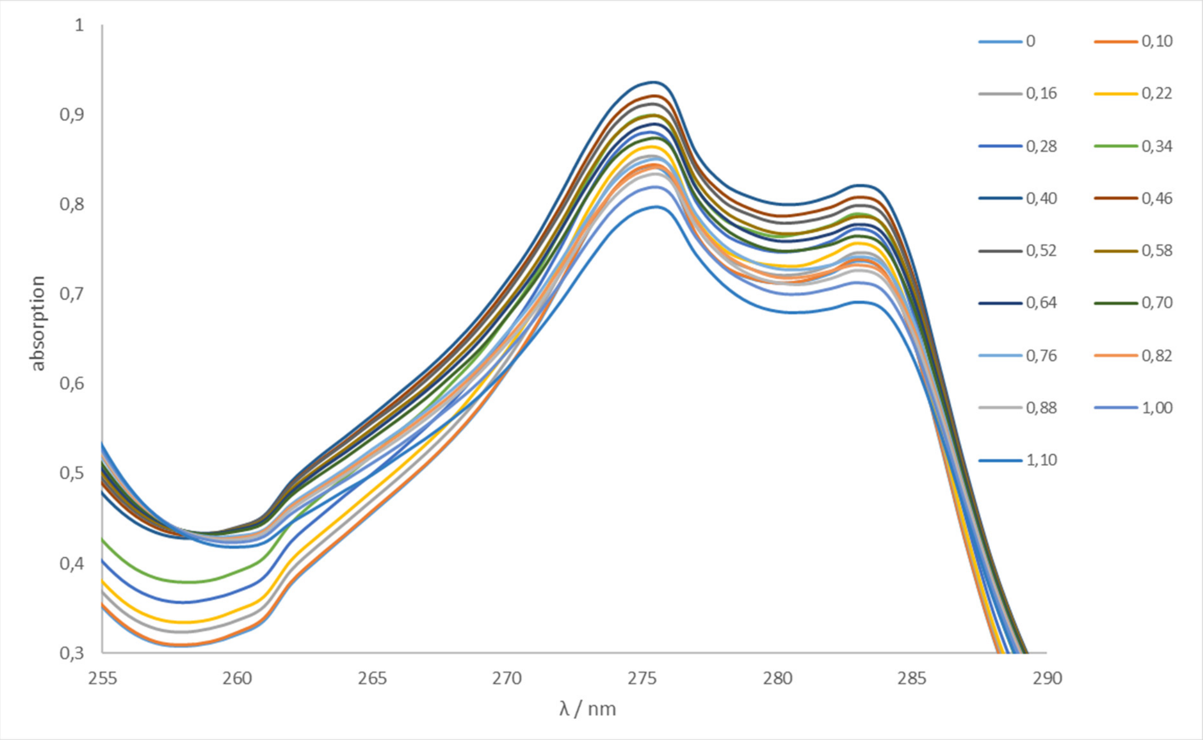

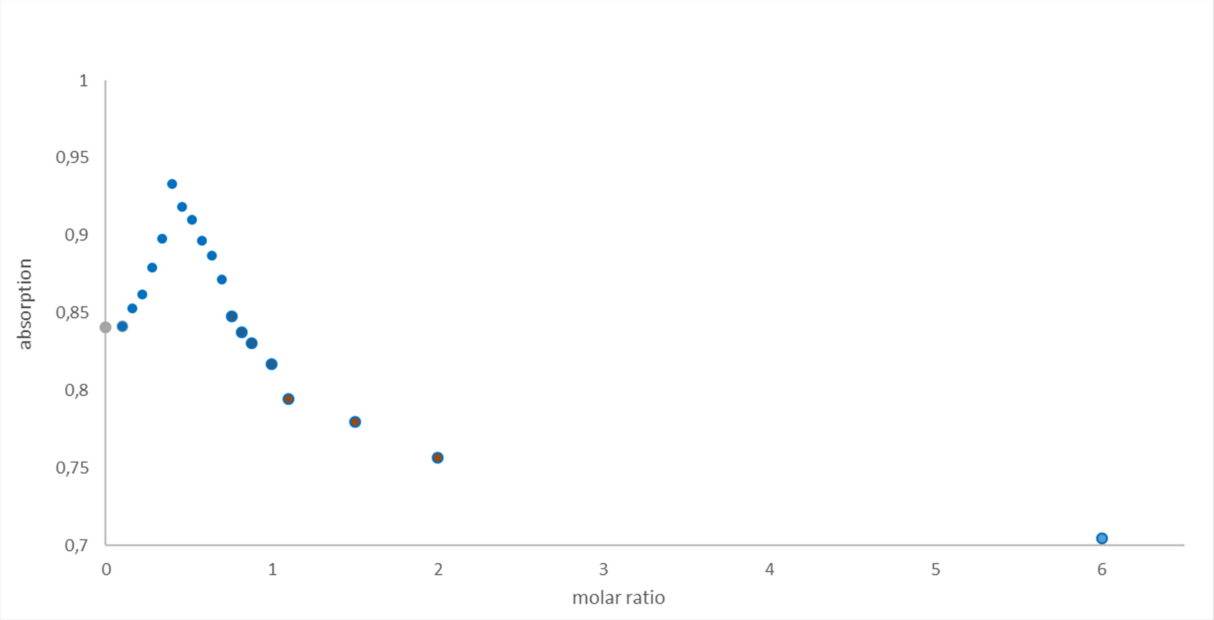

Figure S38. UV spectra and UV titration plot of compound **6** with Ba^{2+}.

Compound **6** with Sr^{2+}

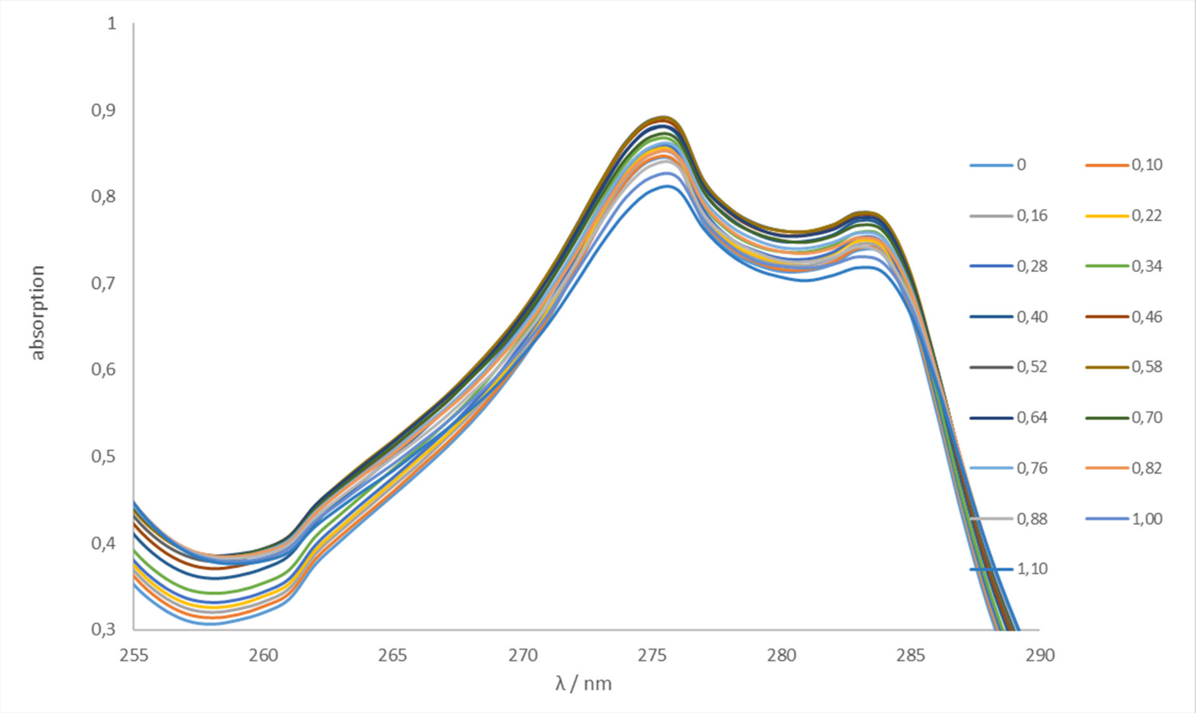

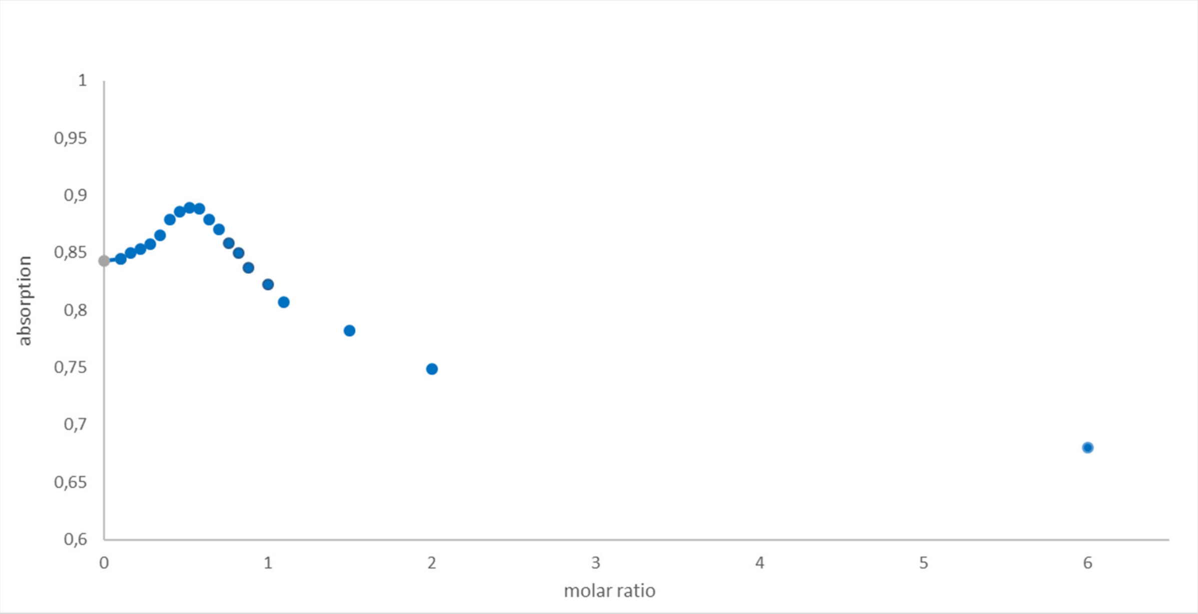

Figure S39. UV spectra and UV titration plot of compound **6** with Sr^{2+}.

Compound **6** with Pb^{2+}

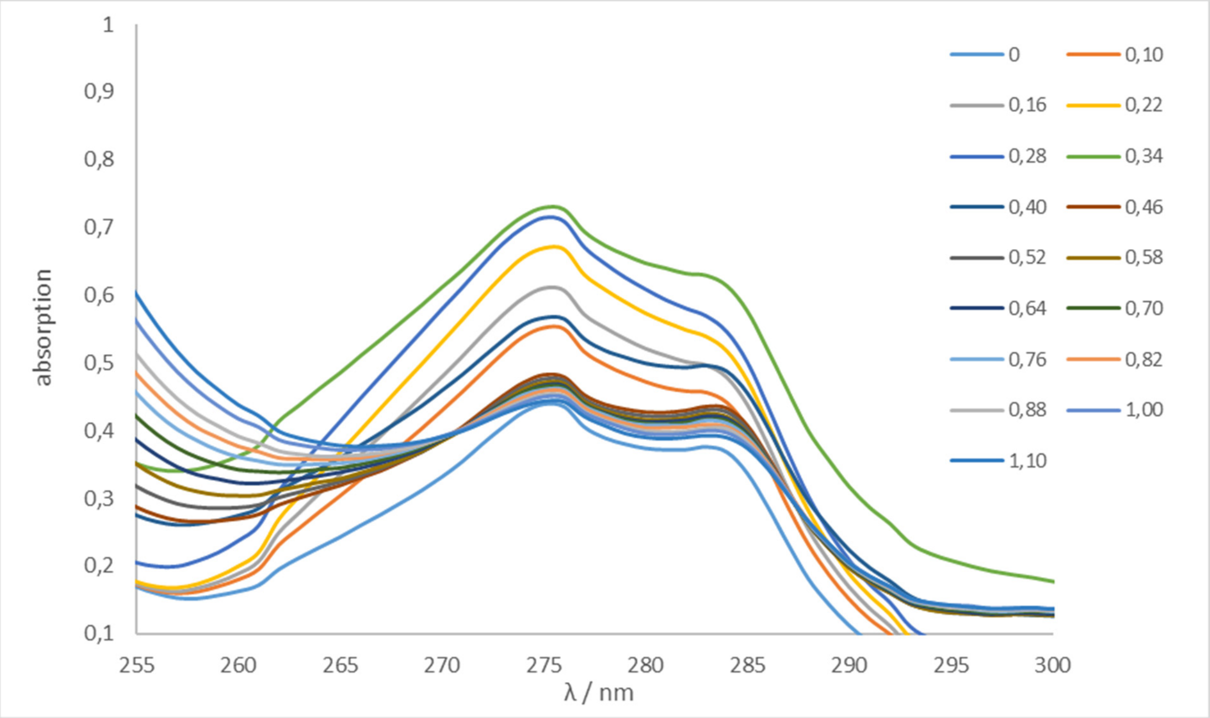

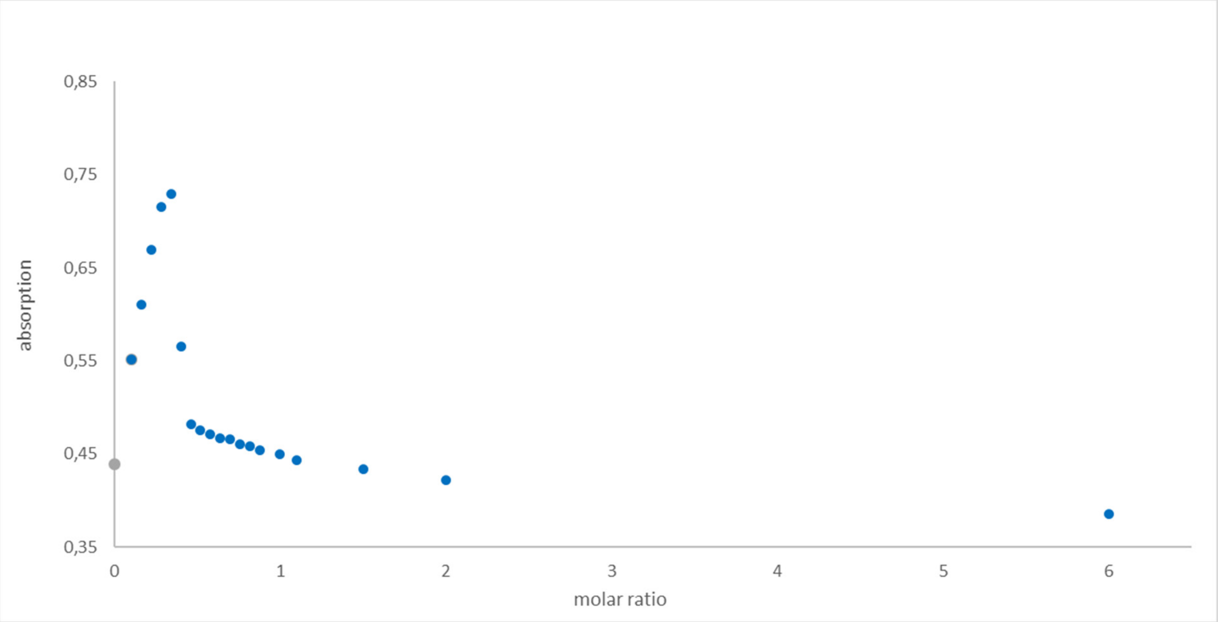

Figure S40. UV spectra and UV titration plot of compound **6** with Pb^{2+}.

Compound **7a** with Ba^{2+}

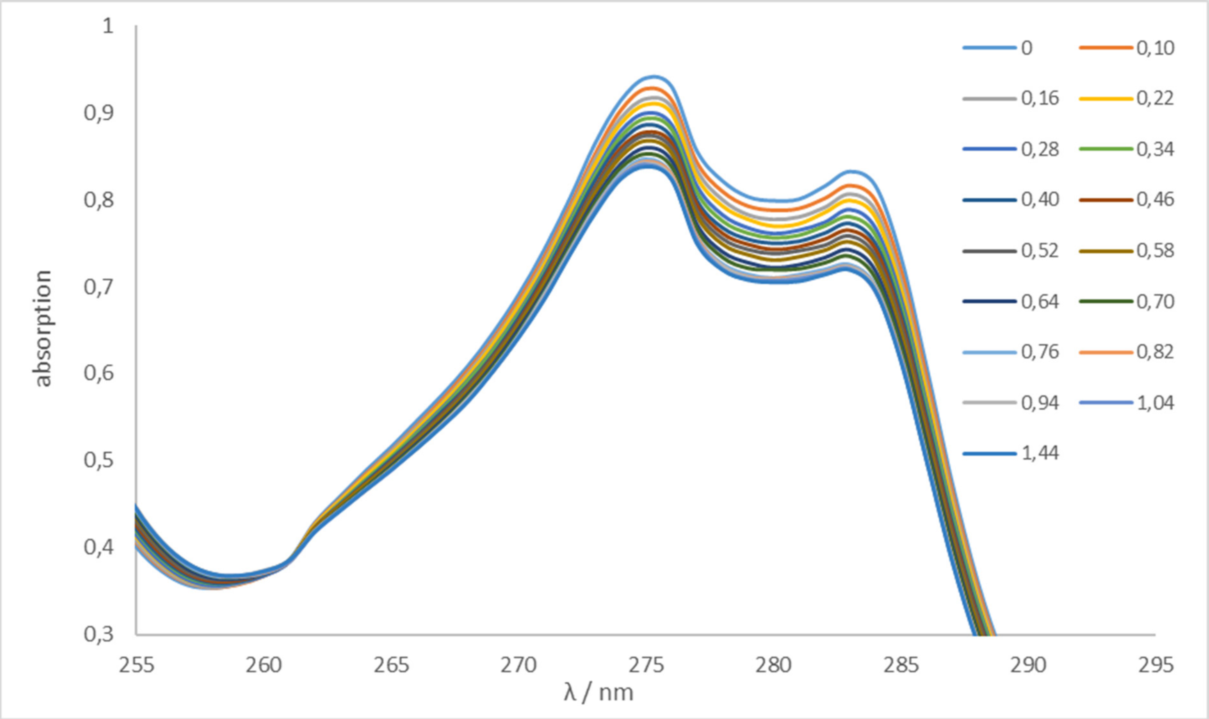

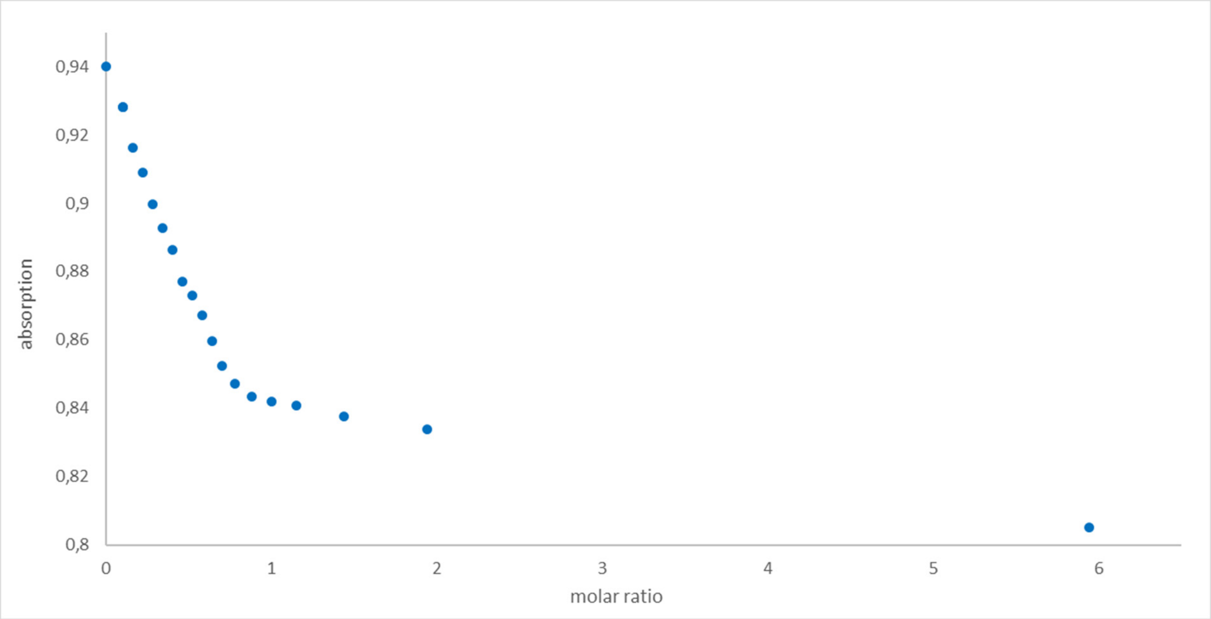

Figure S41. UV spectra and UV titration plot of compound **7a** with Ba^{2+}.

Compound **7a** with Sr^{2+}

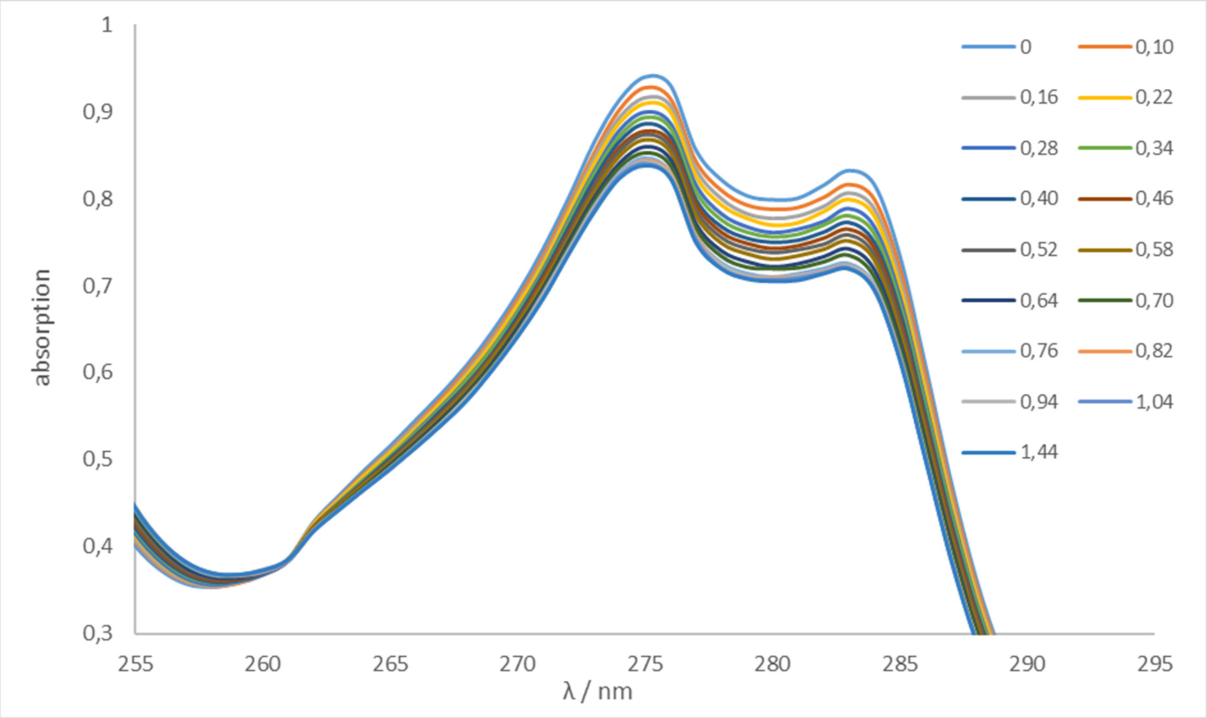

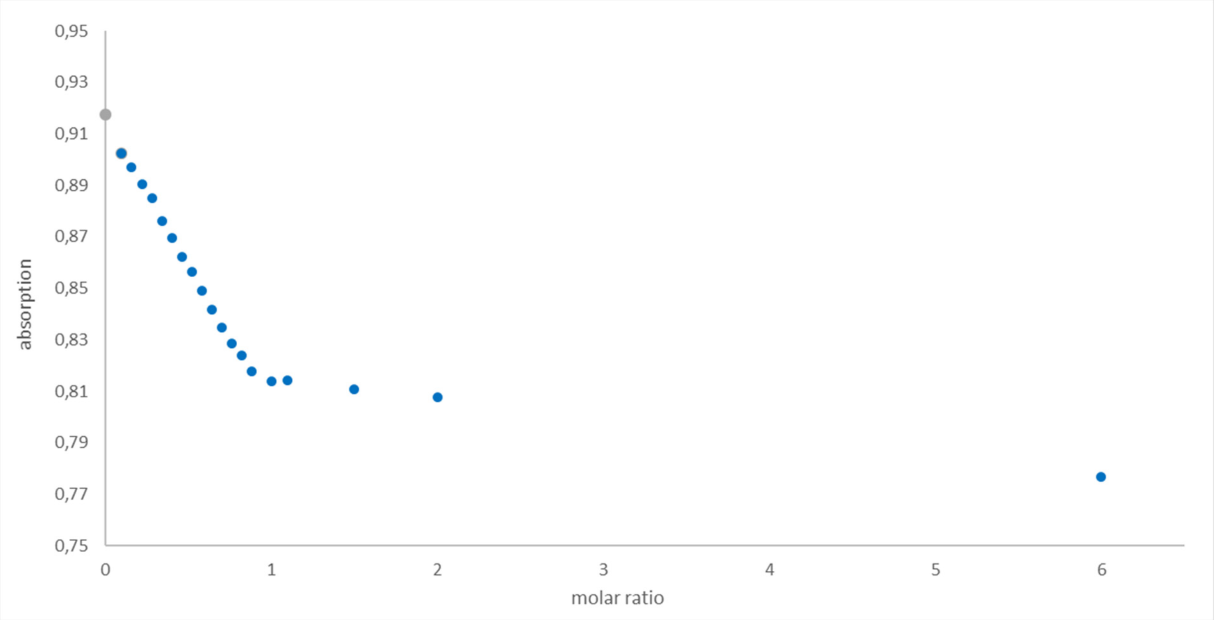

Figure S42. UV spectra and UV titration plot of compound **7a** with Sr^{2+}.

Compound **7a** with Pb^{2+}

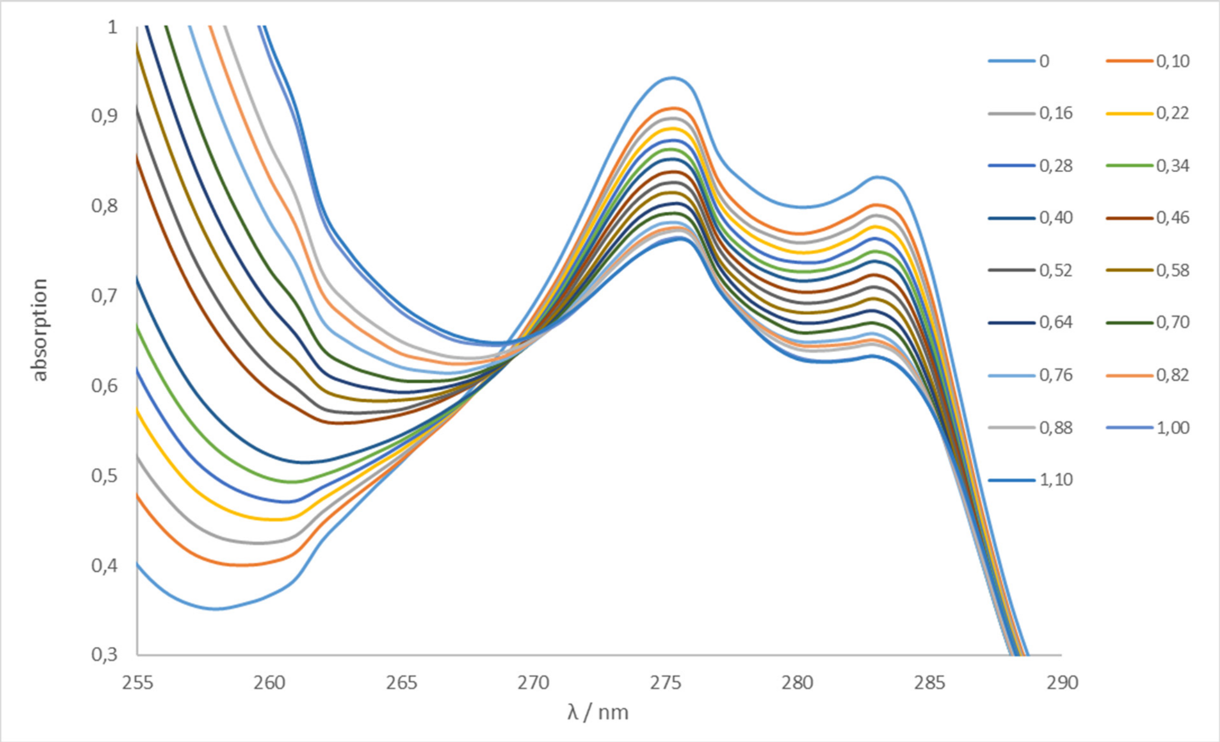

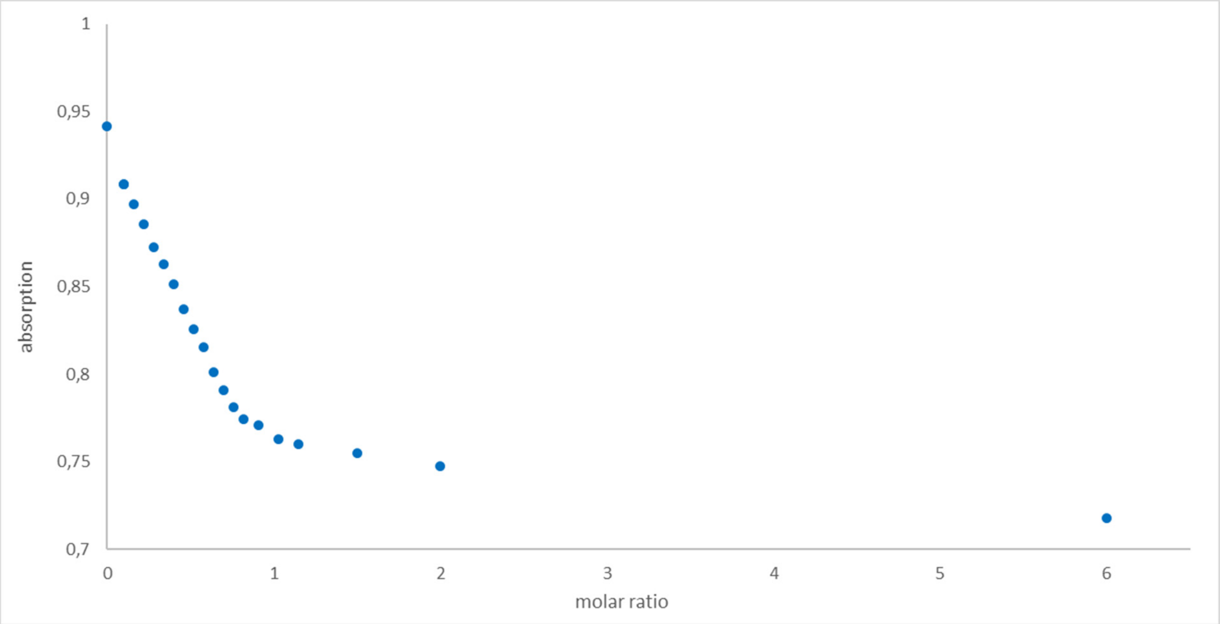

Figure S43. UV spectra and UV titration plot of compound **7a** with Pb^{2+}.

Compound **7b** with Ba^{2+}

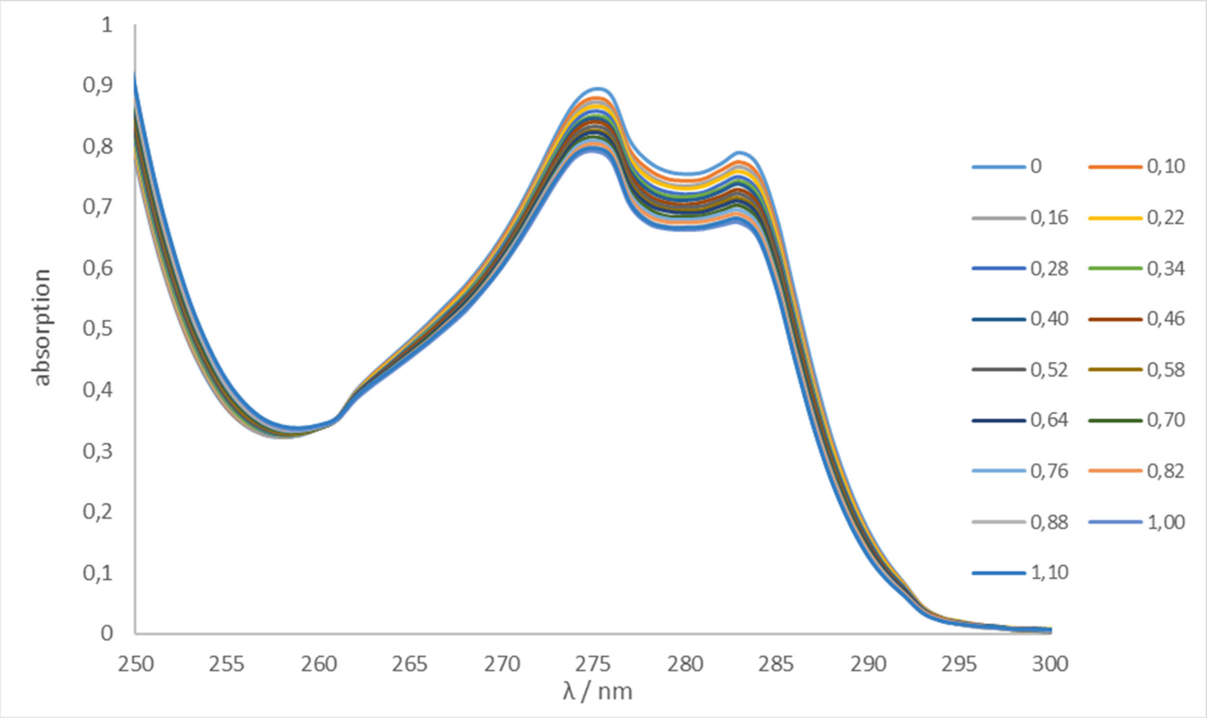

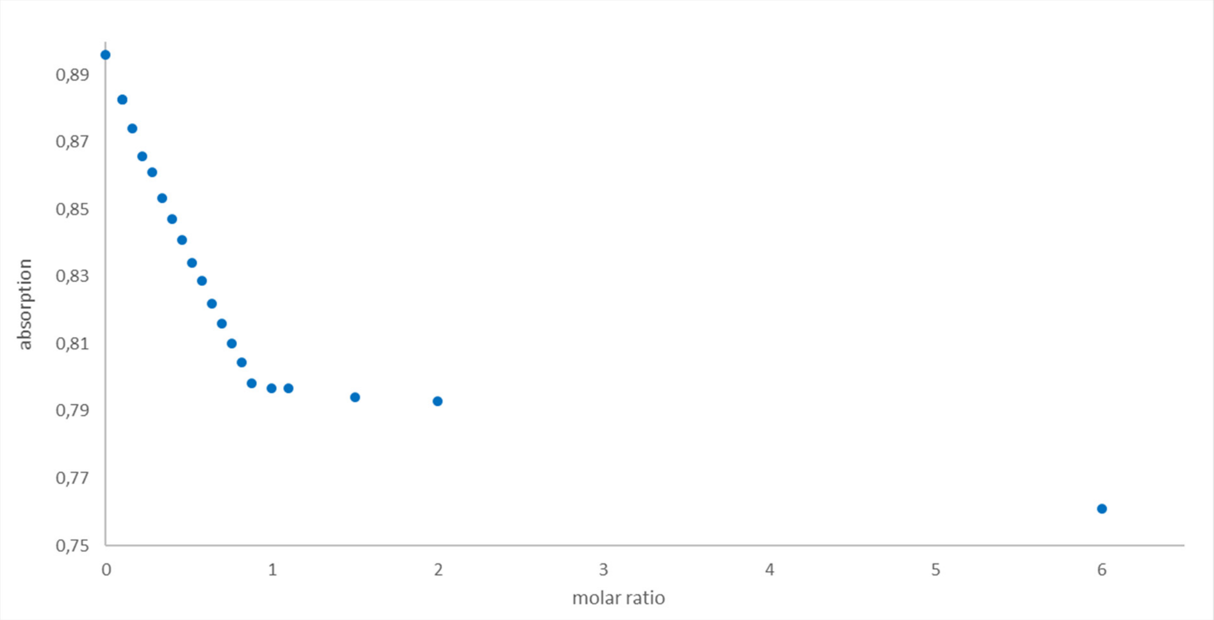

Figure S44. UV spectra and UV titration plot of compound **7b** with Ba^{2+}.

Compound **7b** with Sr^{2+}

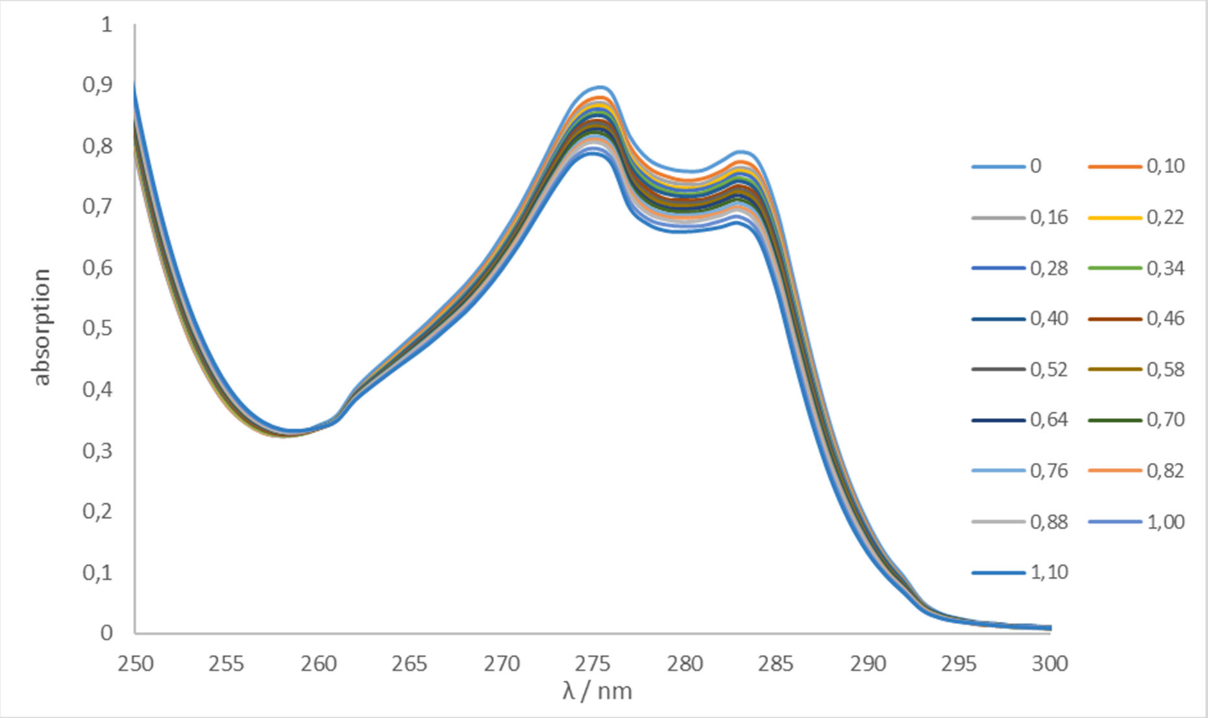

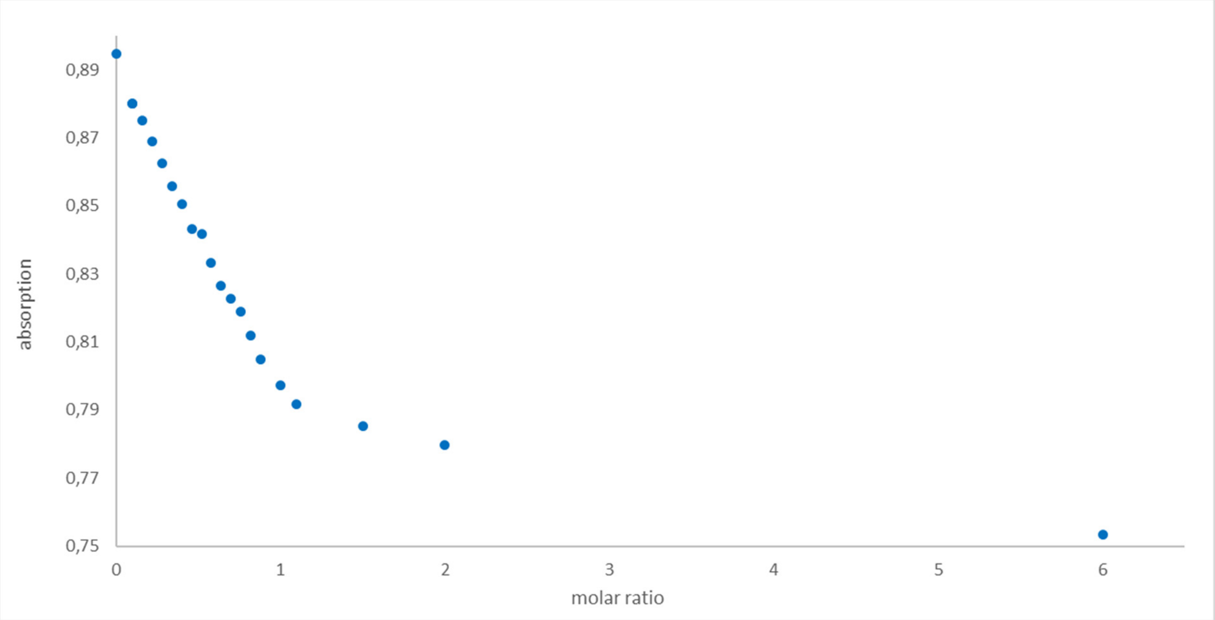

Figure S45. UV spectra and UV titration plot of compound **7b** with Sr^{2+}.

Compound **7b** with Pb^{2+}

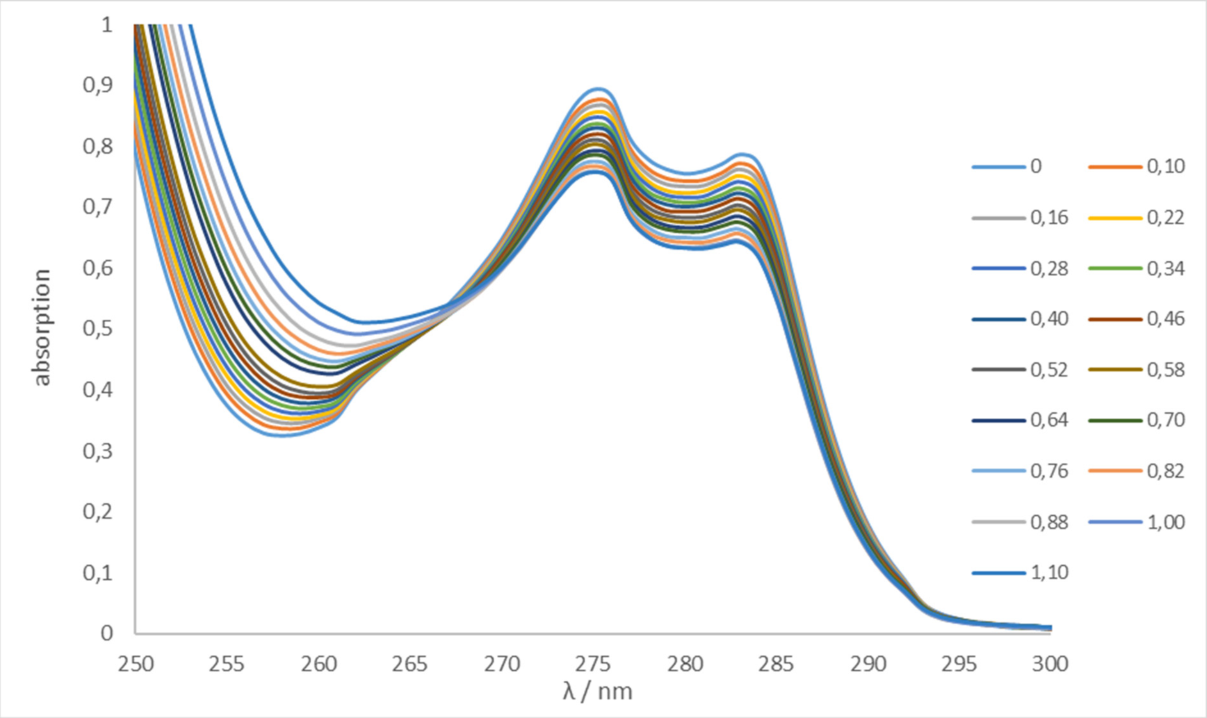

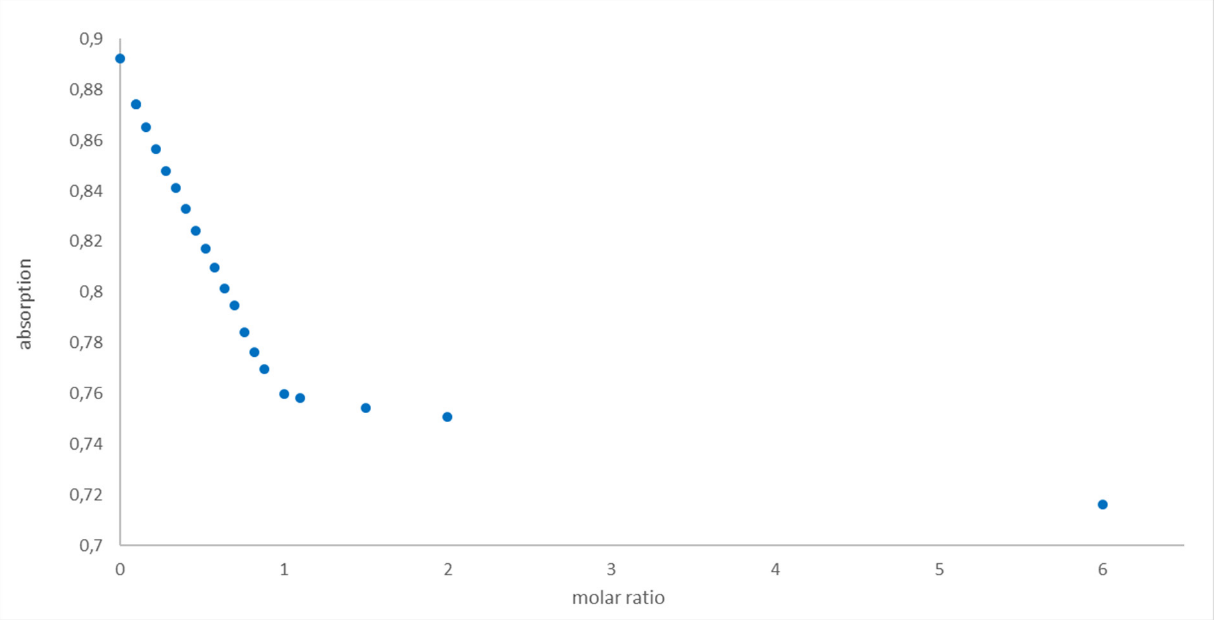

Figure S46. UV spectra and UV titration plot of compound **7b** with Pb^{2+}.

Compound **21** with Ba^{2+}

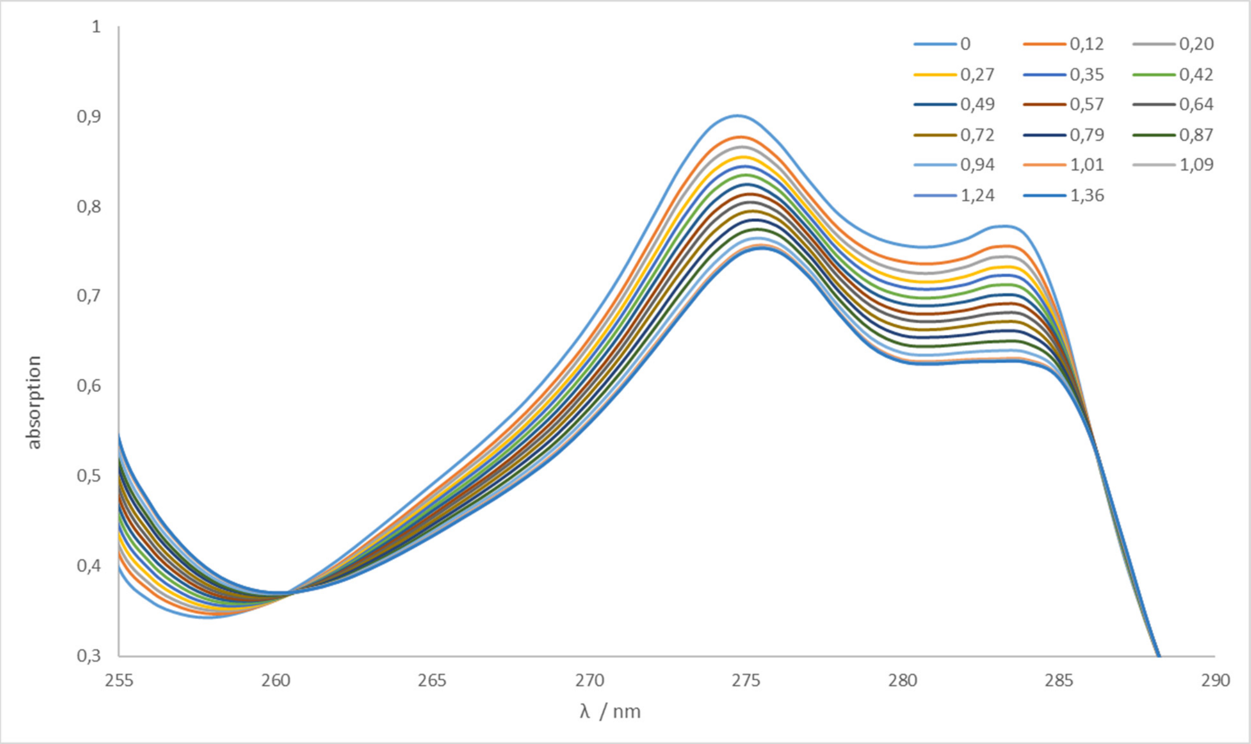

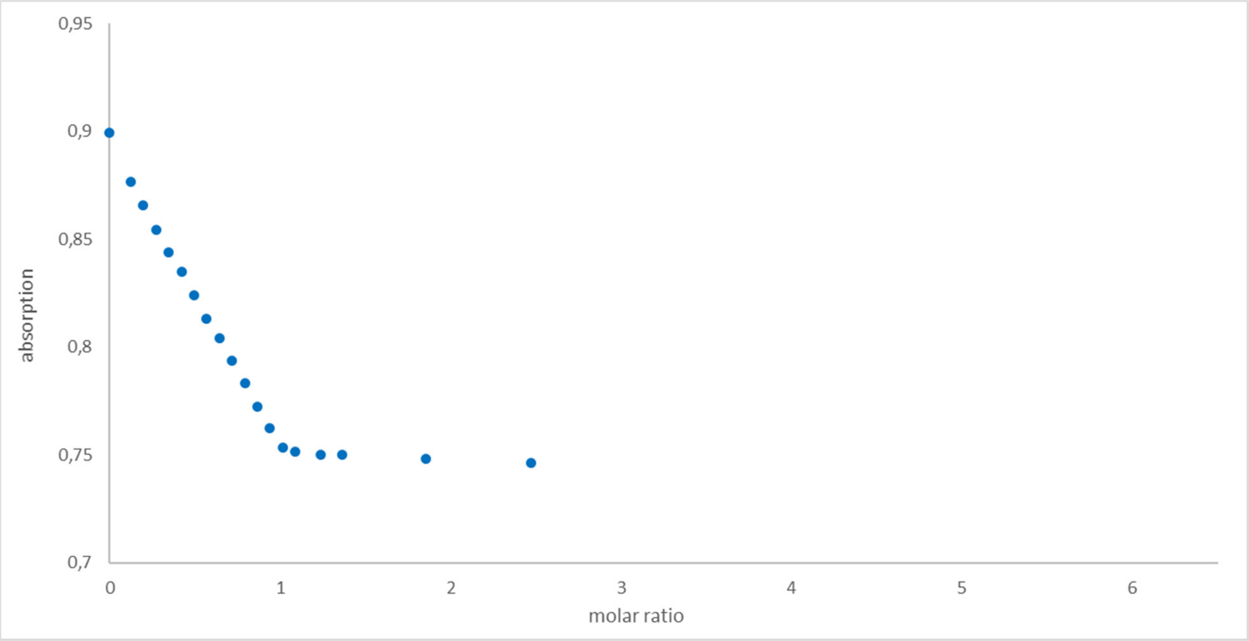

Figure S47. UV spectra and UV titration plot of compound **21** with Ba^{2+}.

Compound **21** with Sr^{2+}

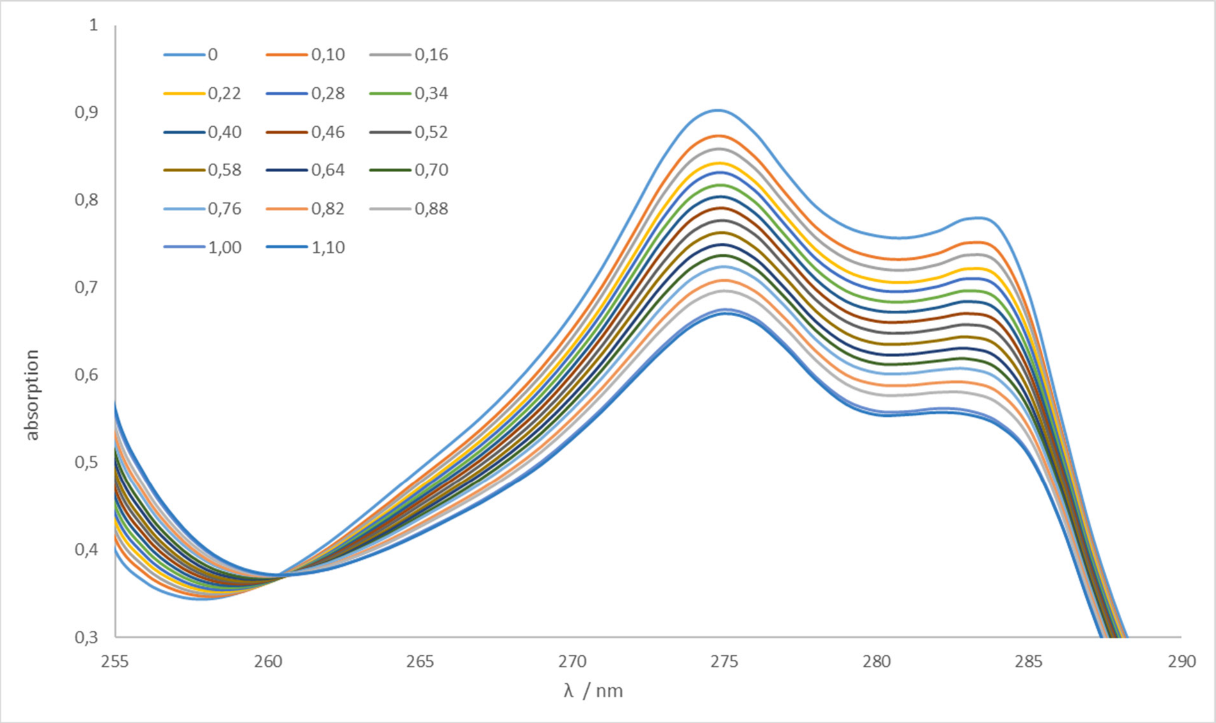

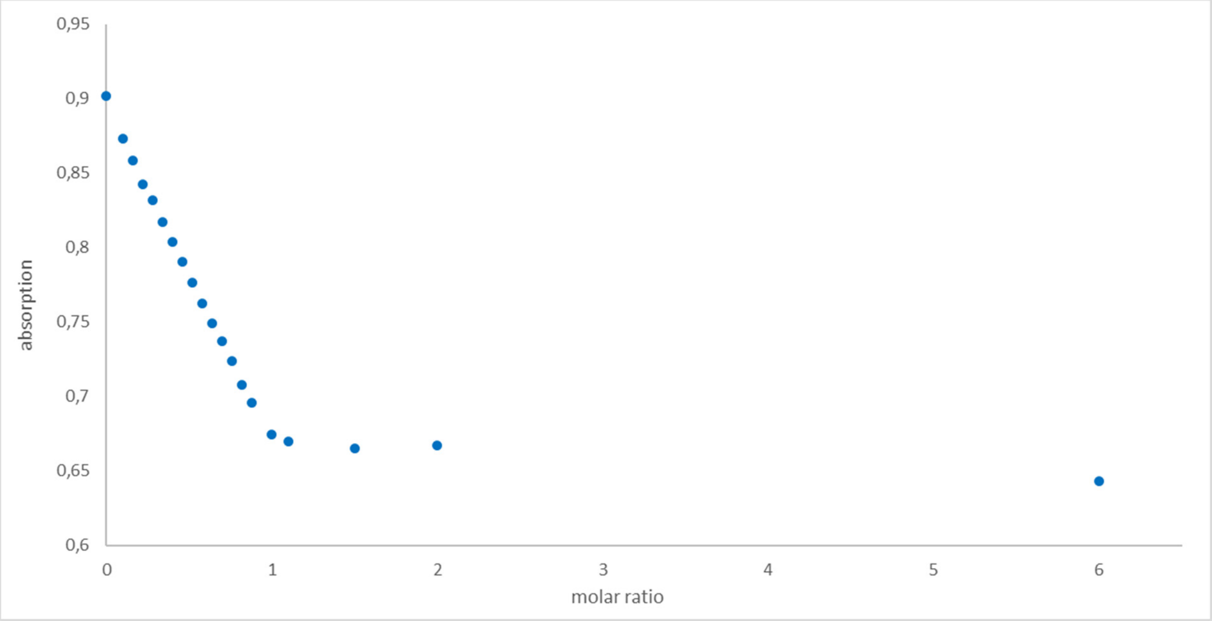

Figure S48. UV spectra and UV titration plot of compound **21** with Sr^{2+}.

Compound **21** with Pb^{2+}

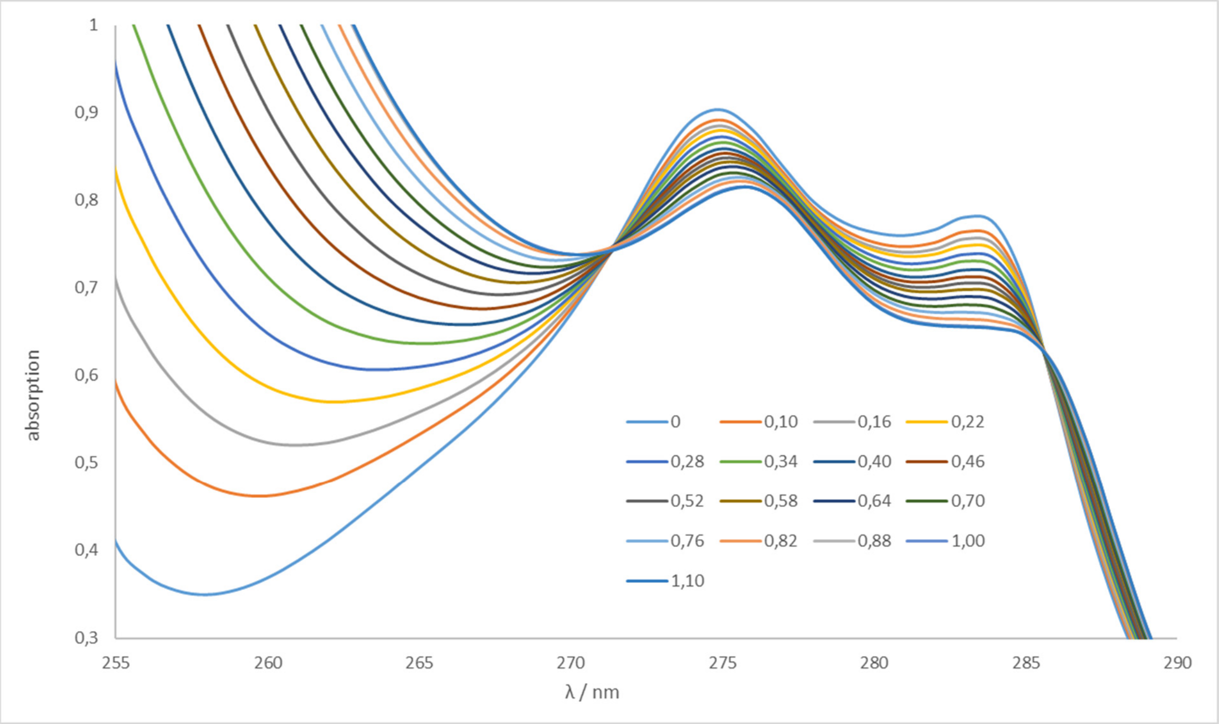

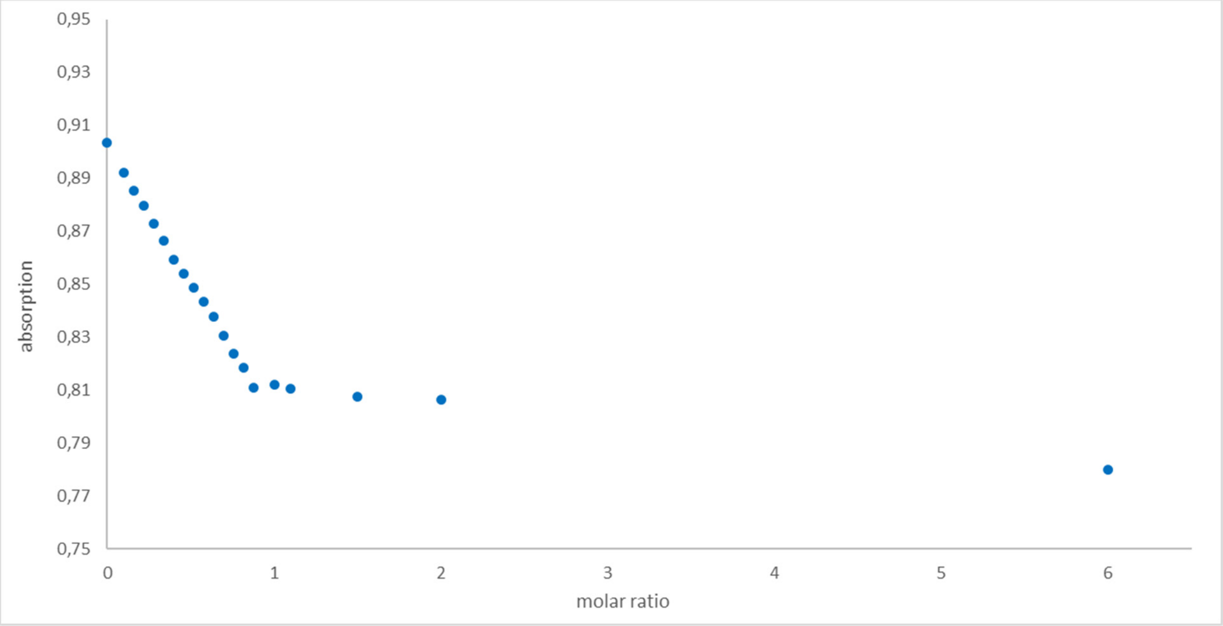

Figure S49. UV spectra and UV titration plot of compound **21** with Pb^{2+}.

Compound **22** with Ba^{2+}

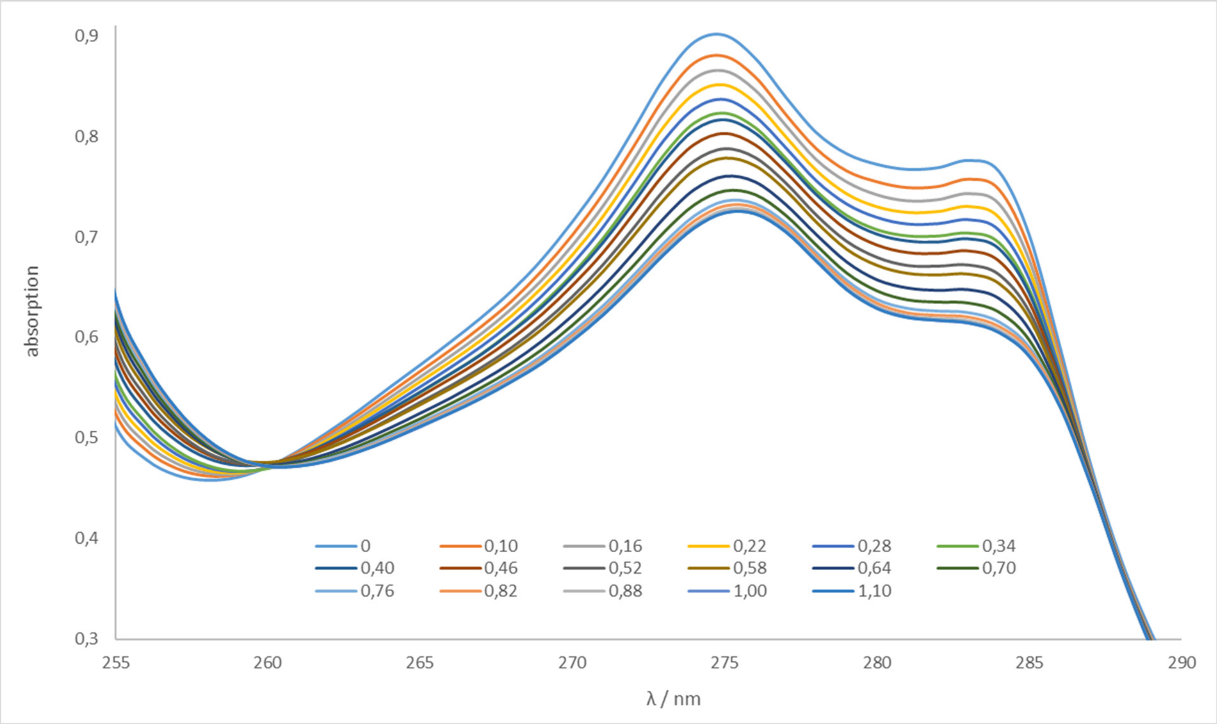

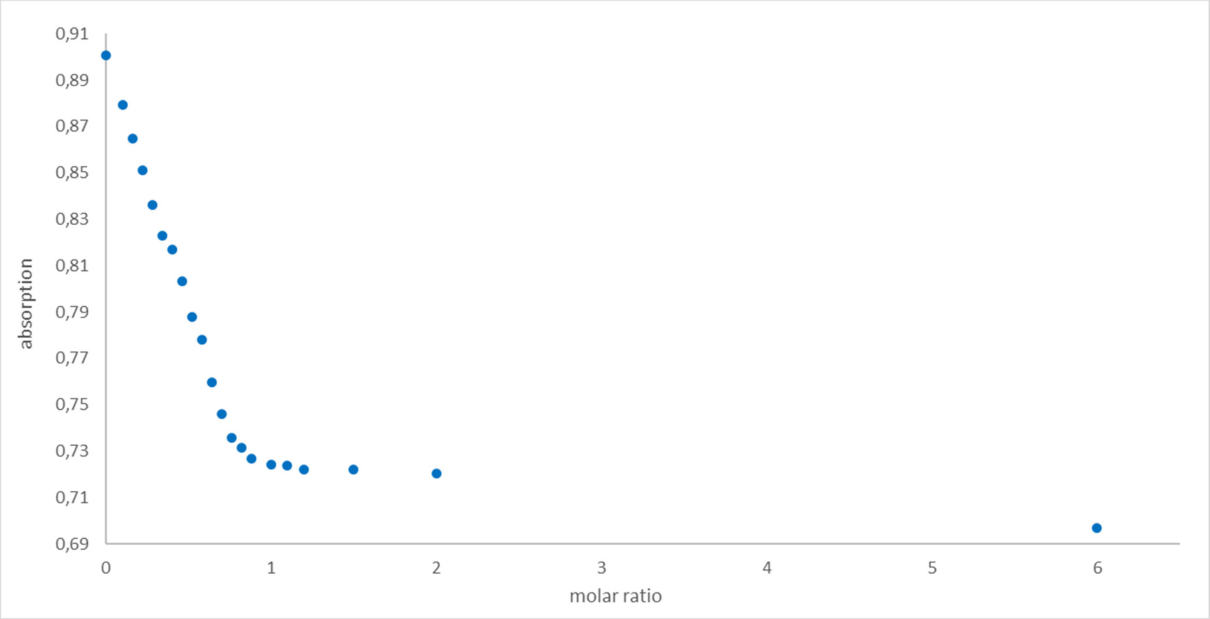

Figure S50. UV spectra and UV titration plot of compound **22** with Ba^{2+}.

Compound **22** with Sr^{2+}

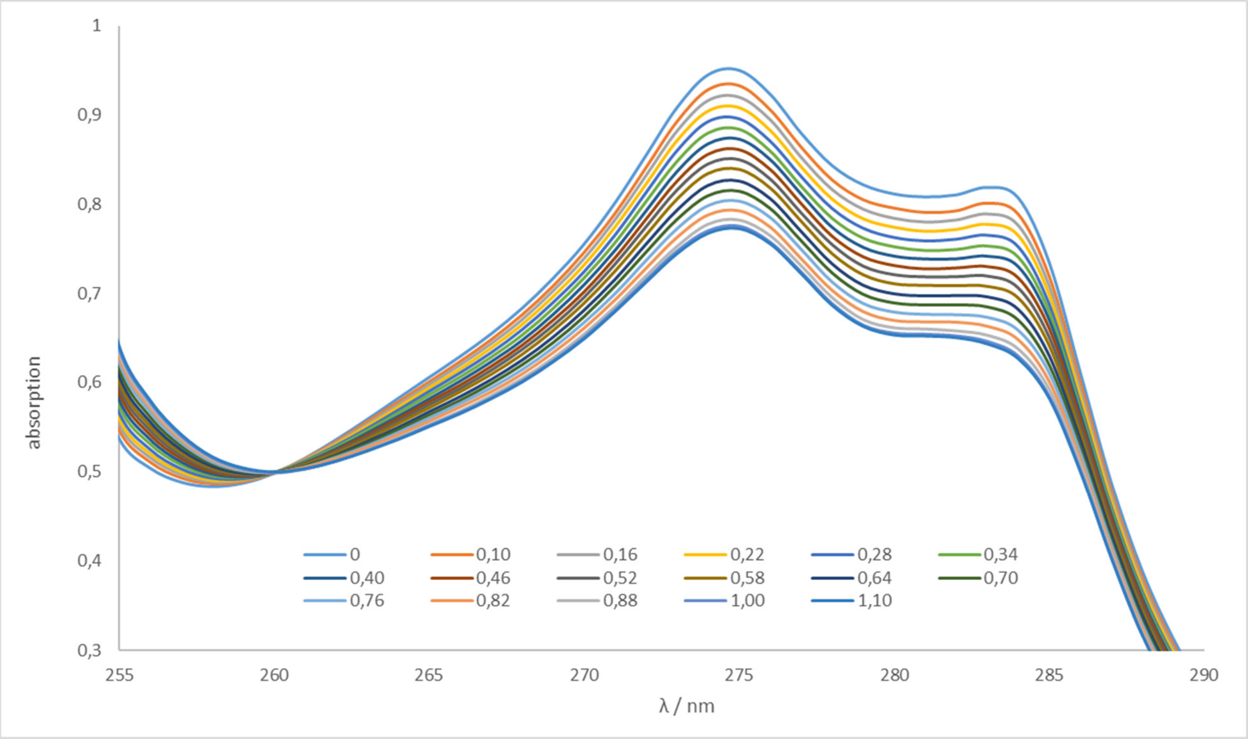

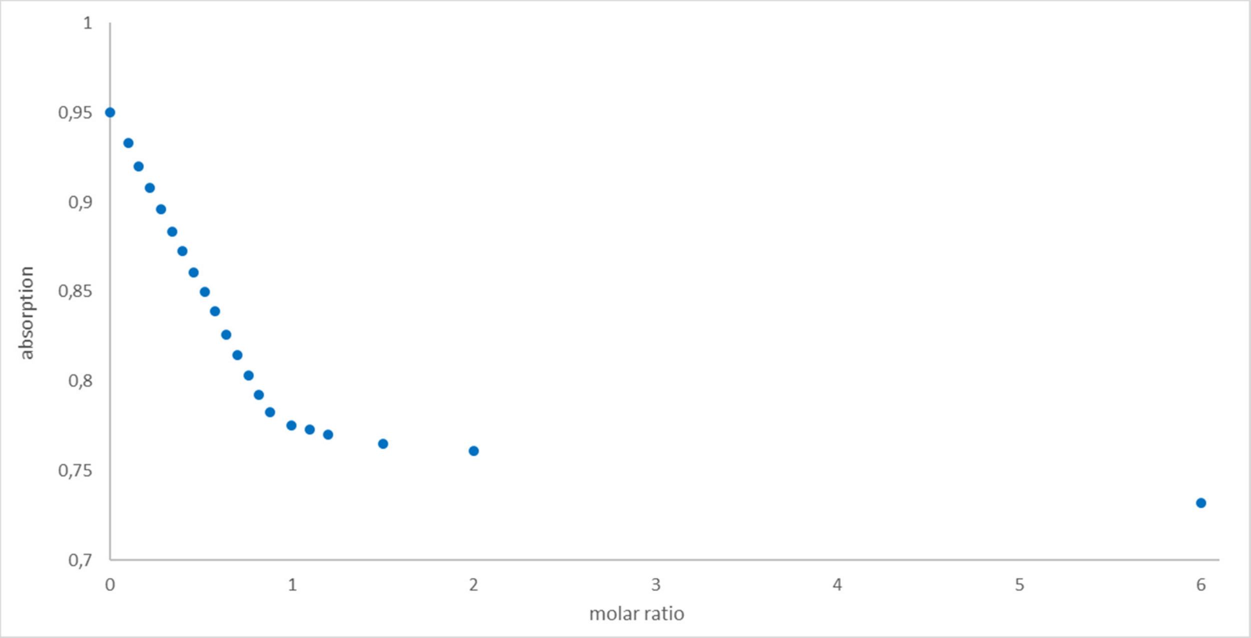

Figure S51. UV spectra and UV titration plot of compound **22** with Sr^{2+}.

Compound **22** with Pb^{2+}

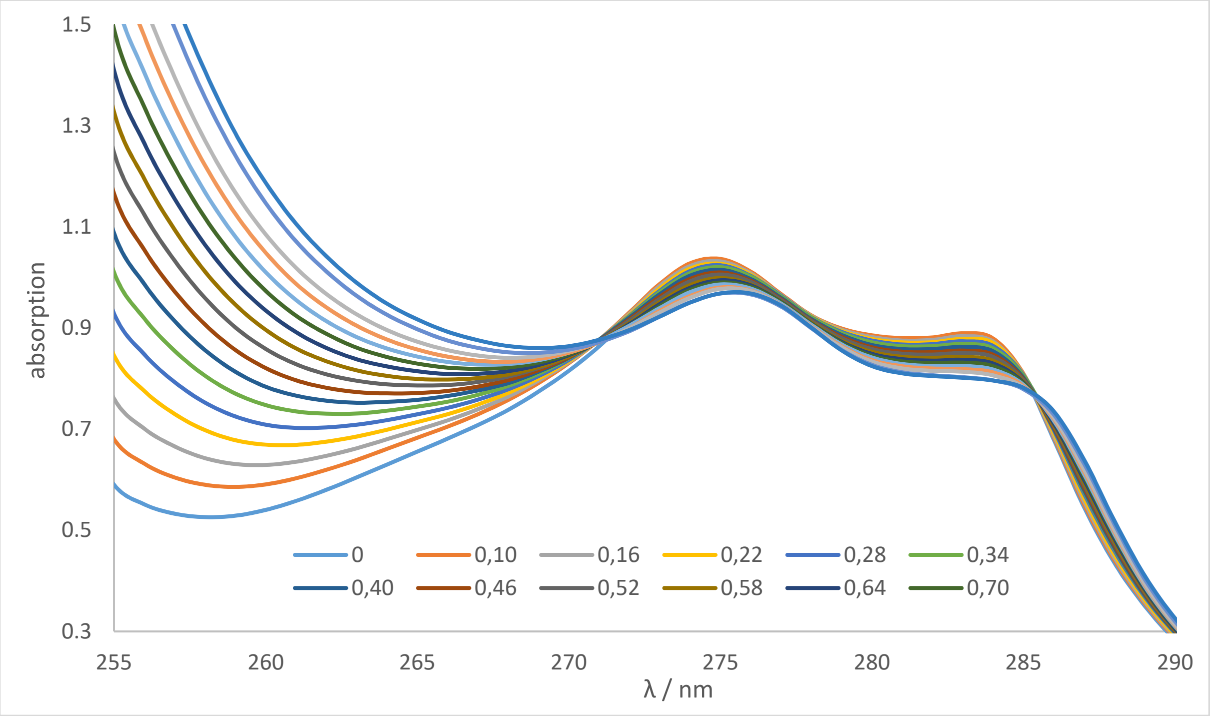

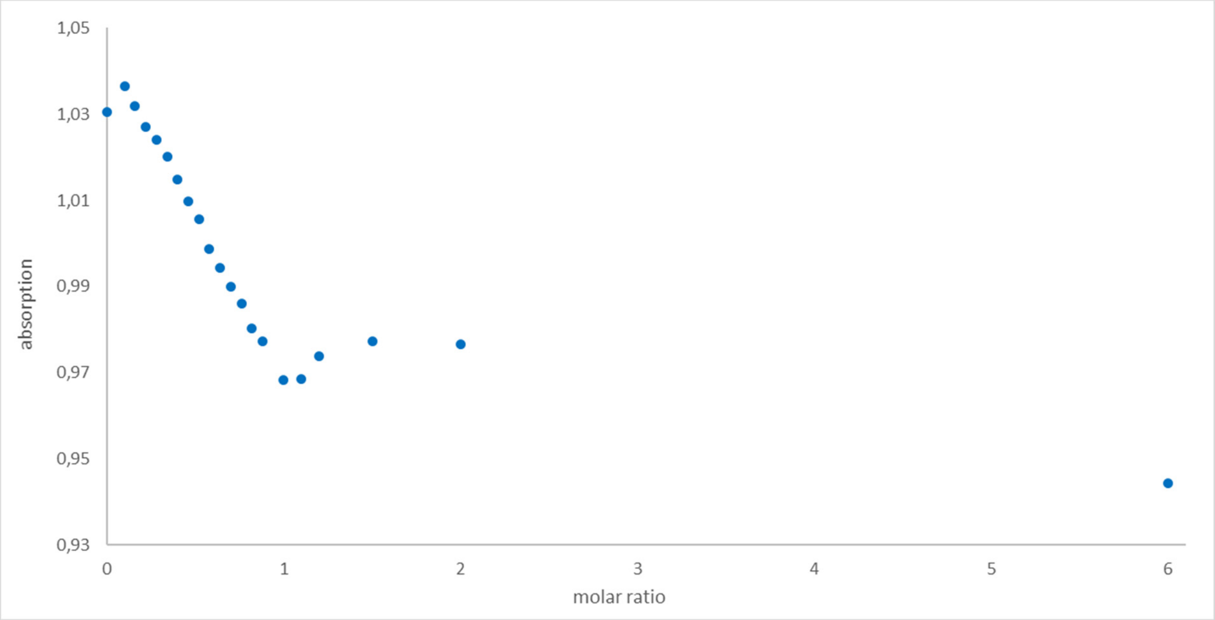

Figure S52. UV spectra and UV titration plot of compound **22** with Pb^{2+}.

Compound **23** with Ba^{2+}

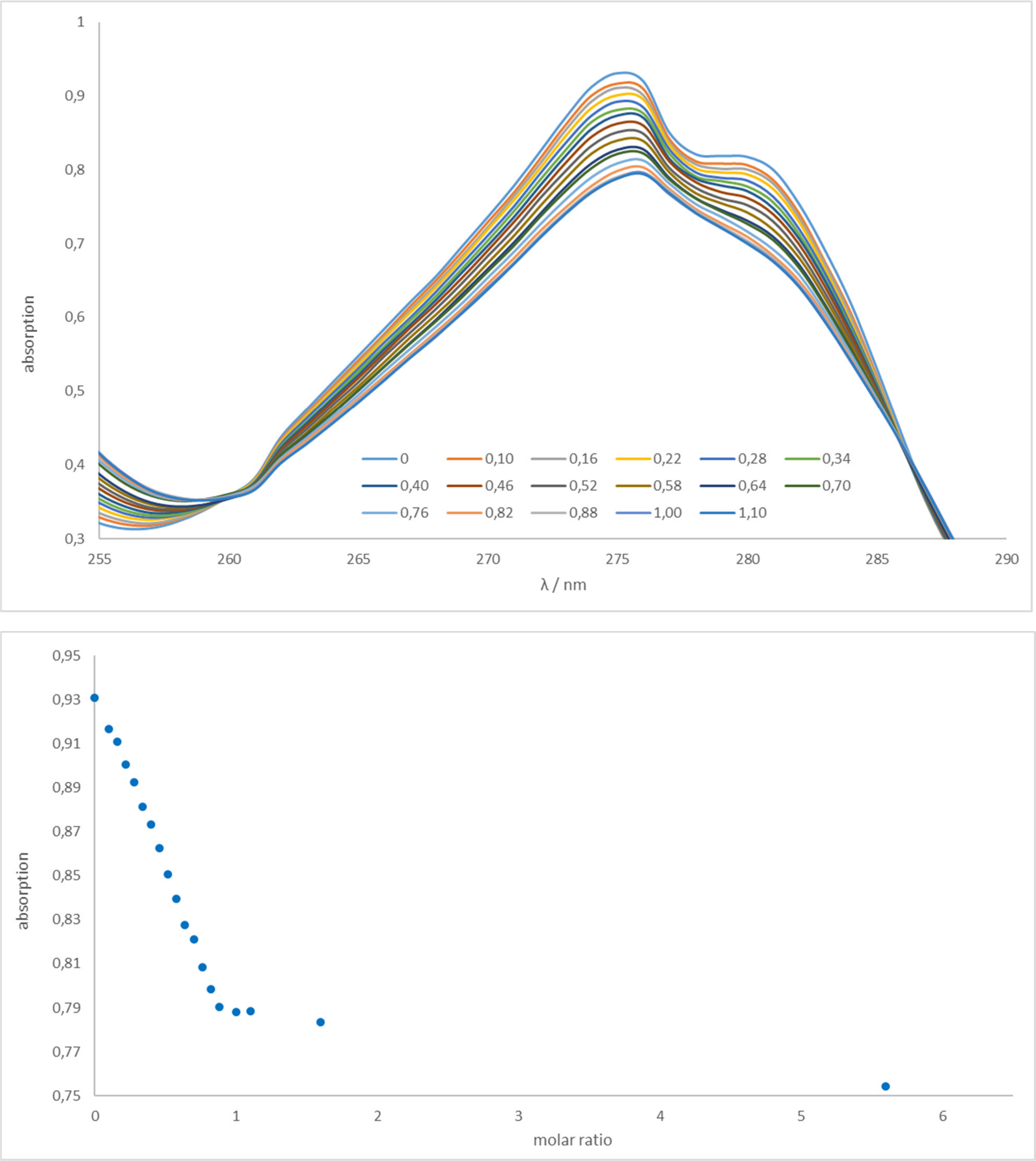

Figure S53. UV spectra and UV titration plot of compound **23** with Ba^{2+}.

Compound **23** with Sr^{2+}

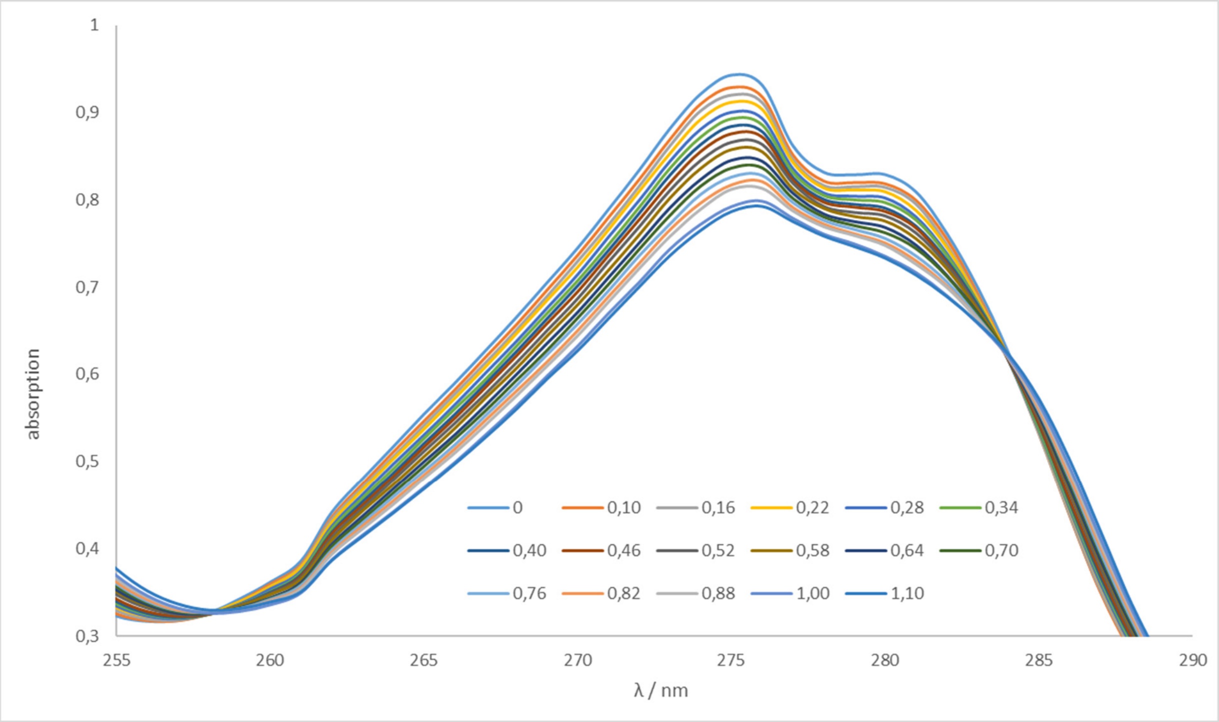

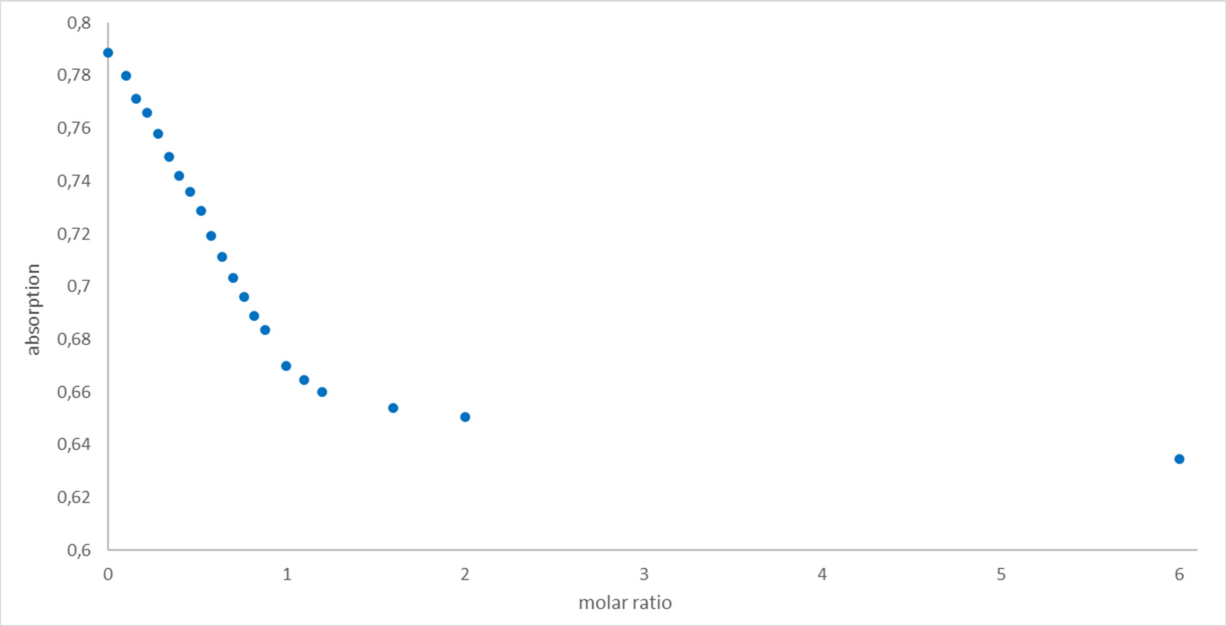

Figure S54. UV spectra and UV titration plot of compound **23** with Sr^{2+}.

Compound **23** with Pb^{2+}

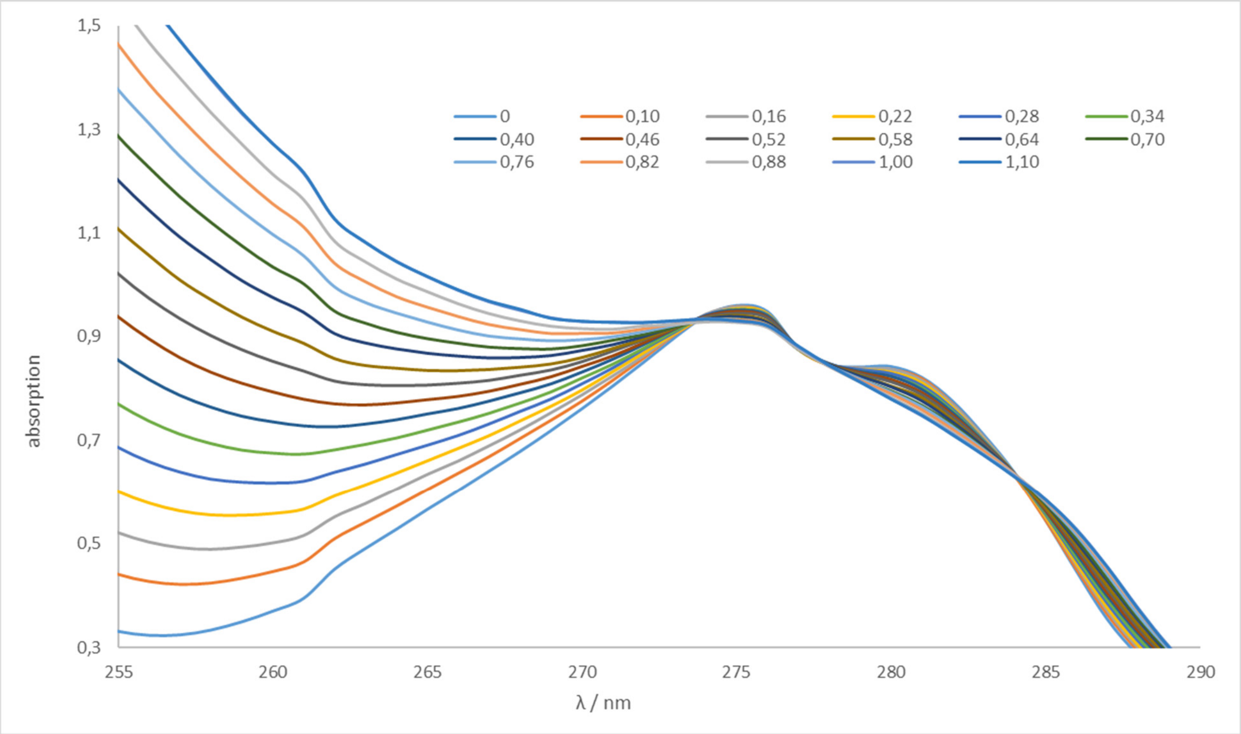

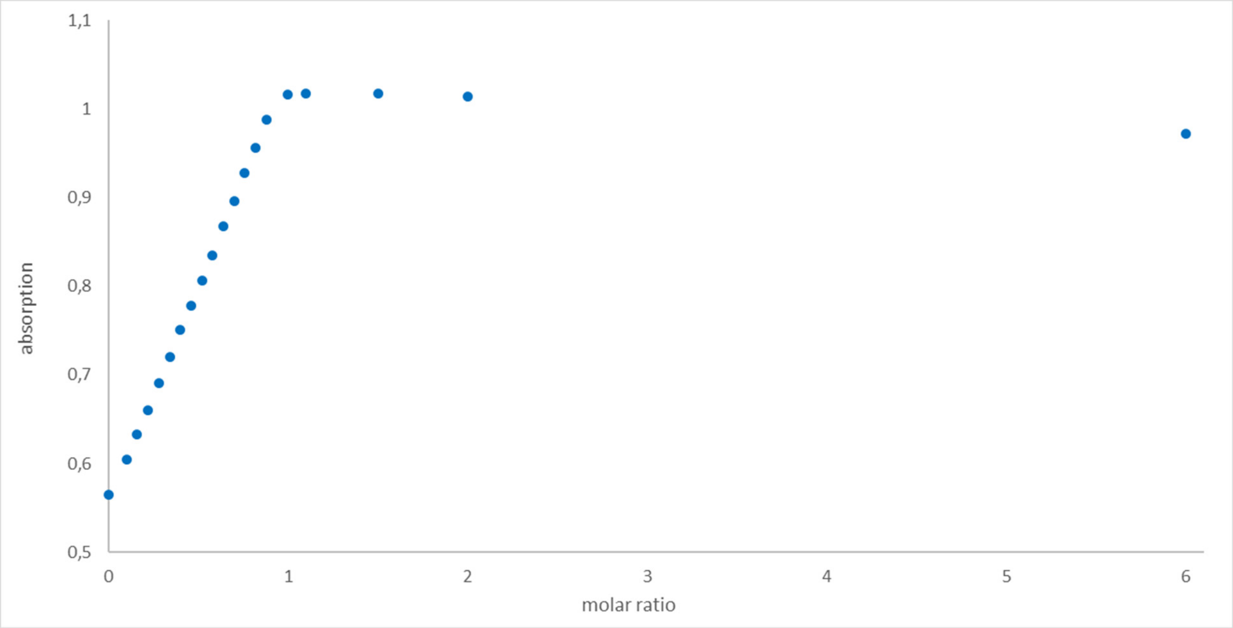

Figure S55. UV spectra and UV titration plot of compound **23** with Pb^{2+}.

Compound **24** with Ba^{2+}

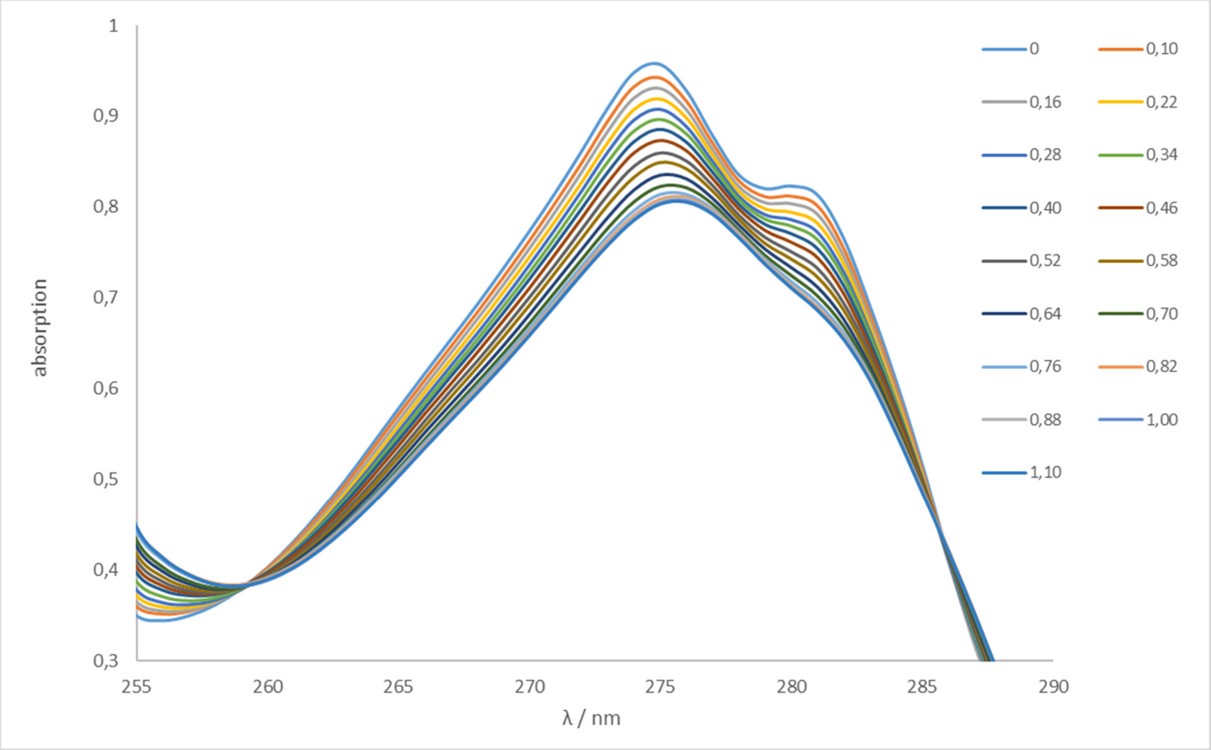

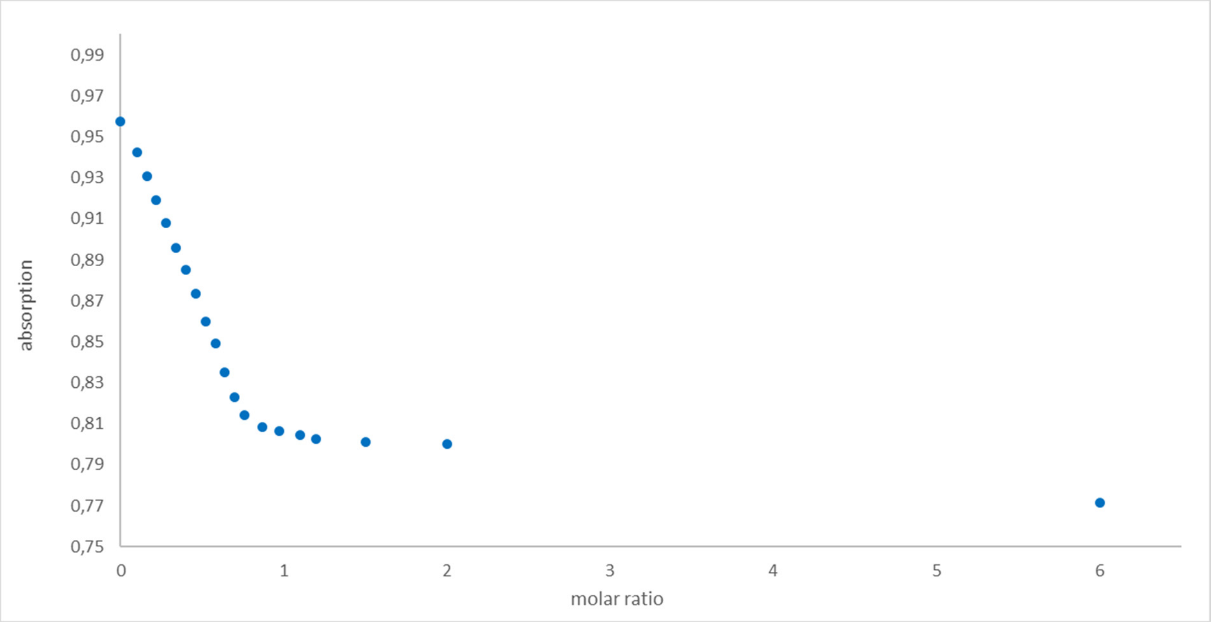

Figure S56. UV spectra and UV titration plot of compound **24** with Ba^{2+}.

Compound **24** with Sr^{2+}

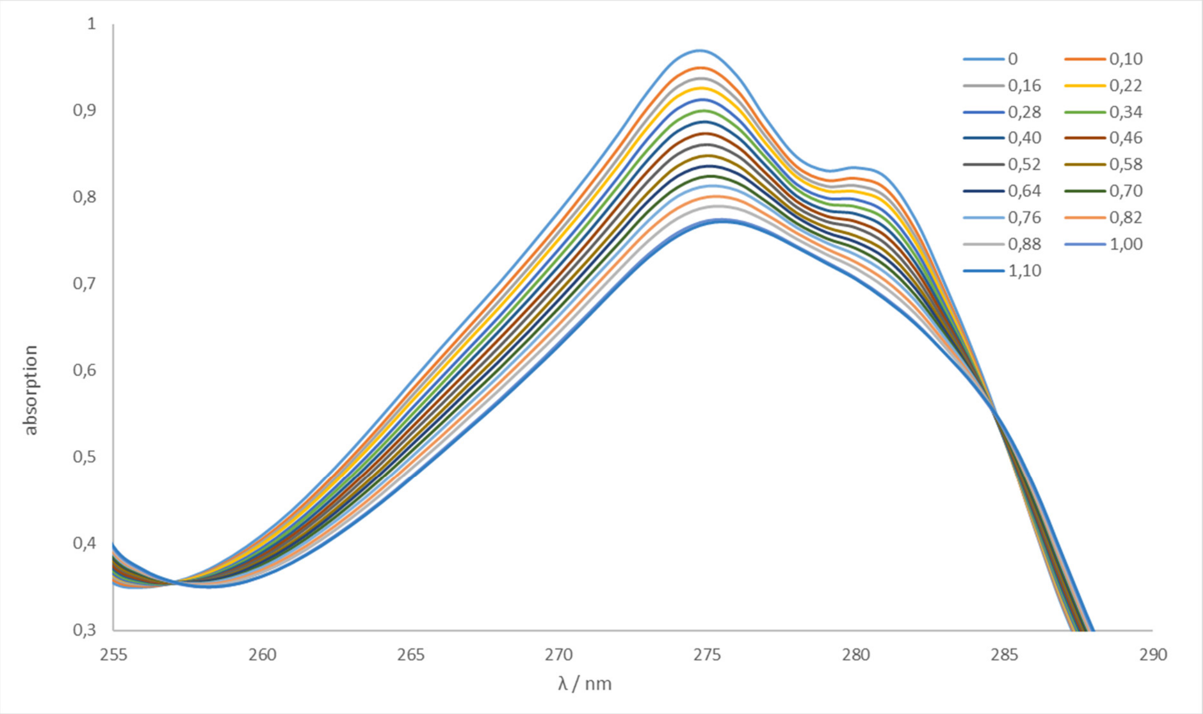

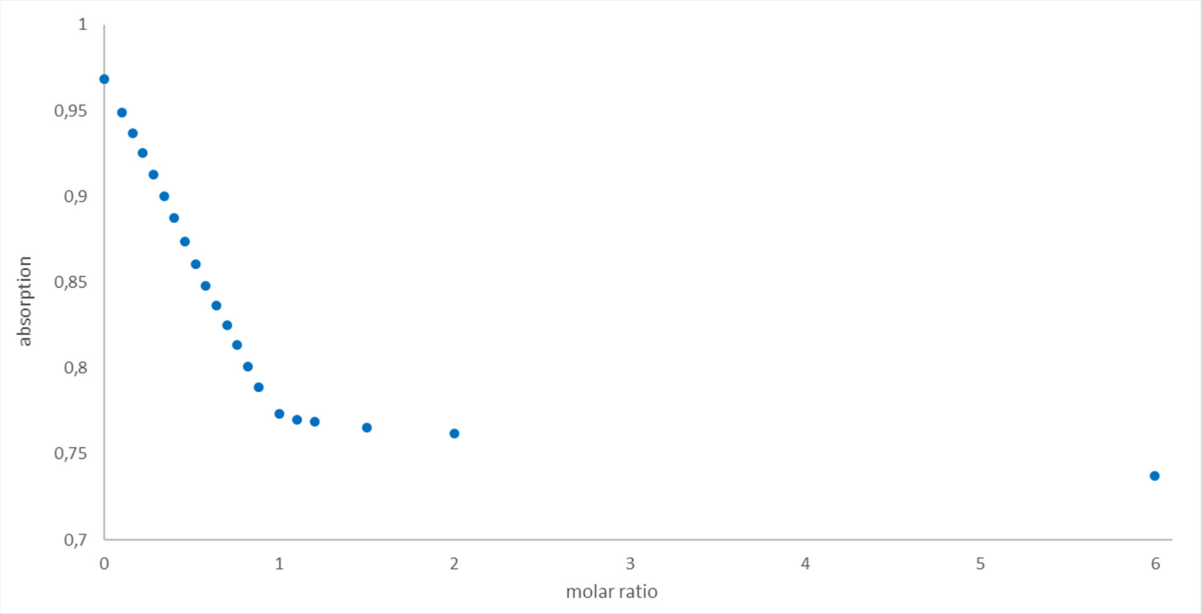

Figure S57. UV spectra and UV titration plot of compound **24** with Sr^{2+}.

Compound **24** with Pb^{2+}

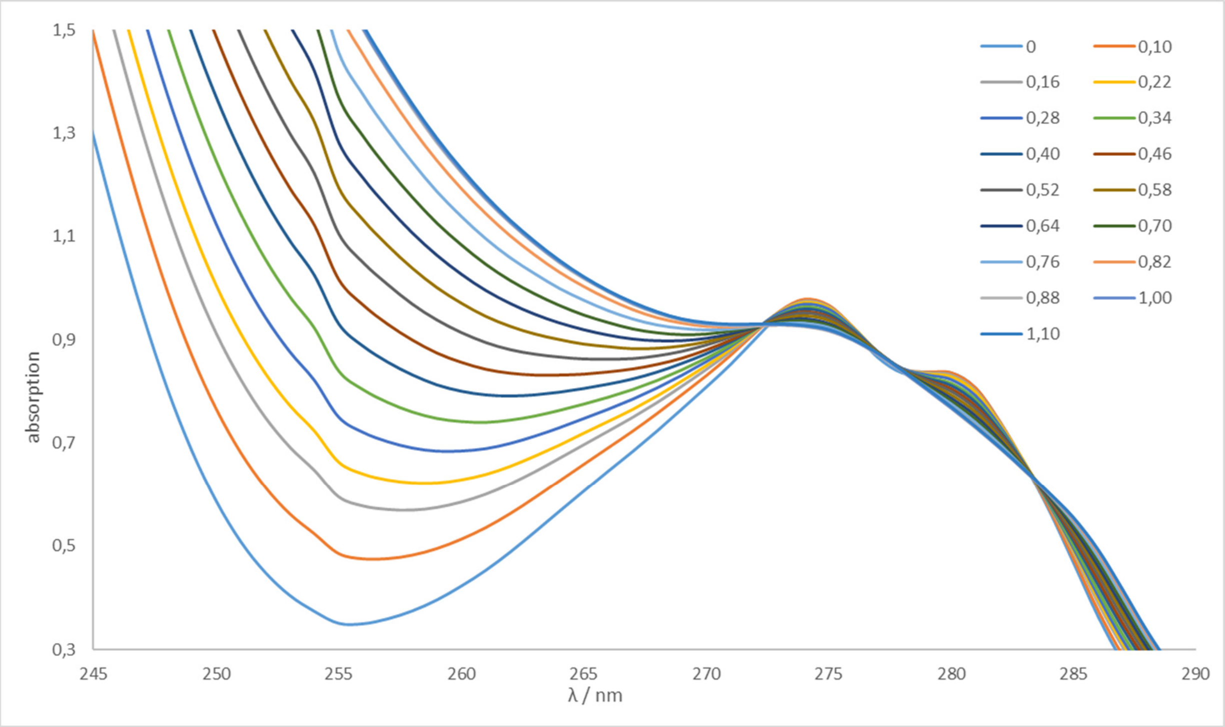

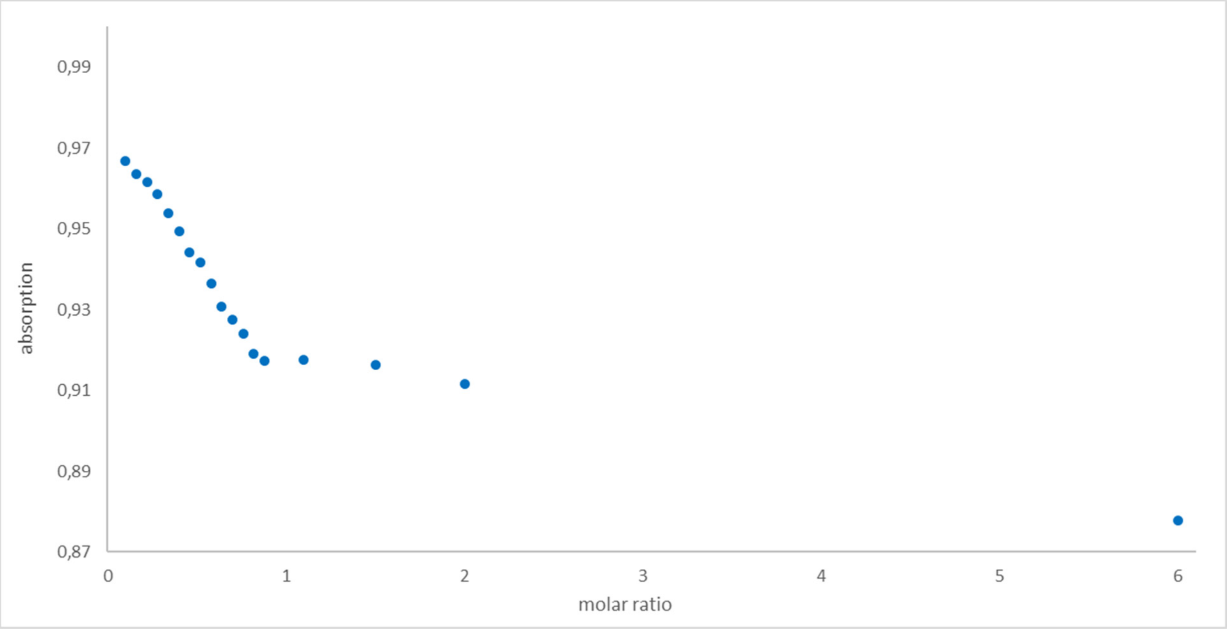

Figure S58. UV spectra and UV titration plot of compound **24** with Pb^{2+}.

Compound **25** with Ba^{2+}

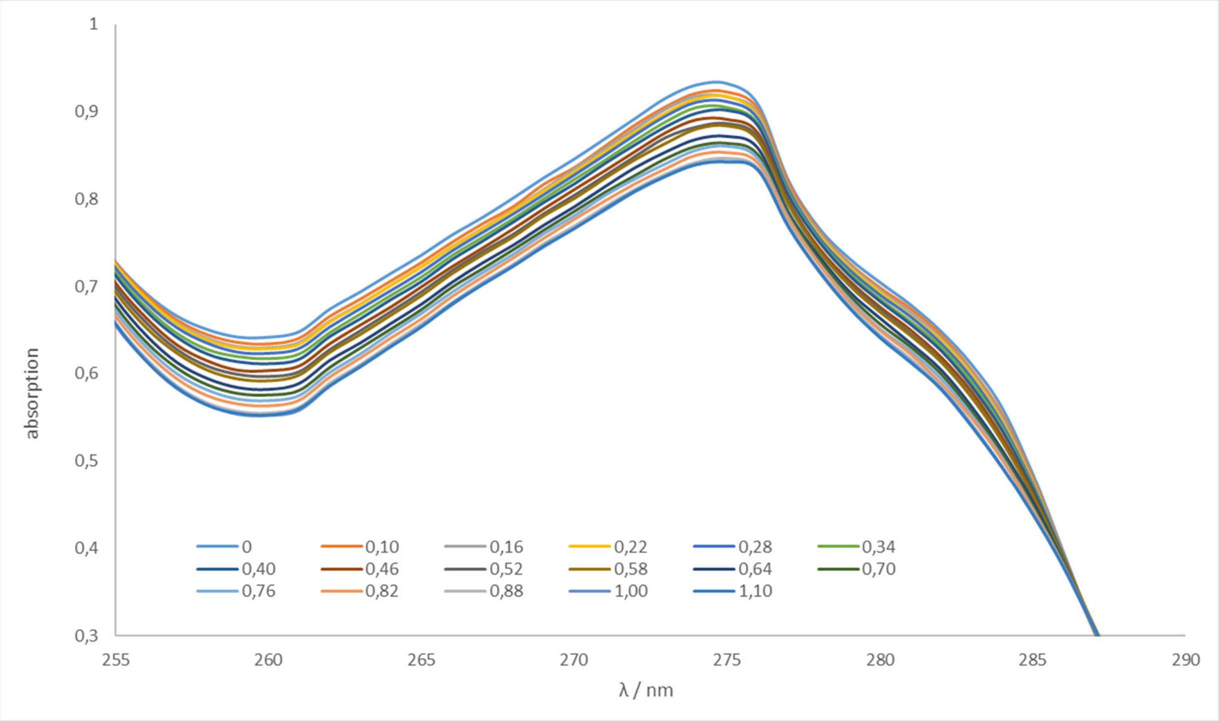

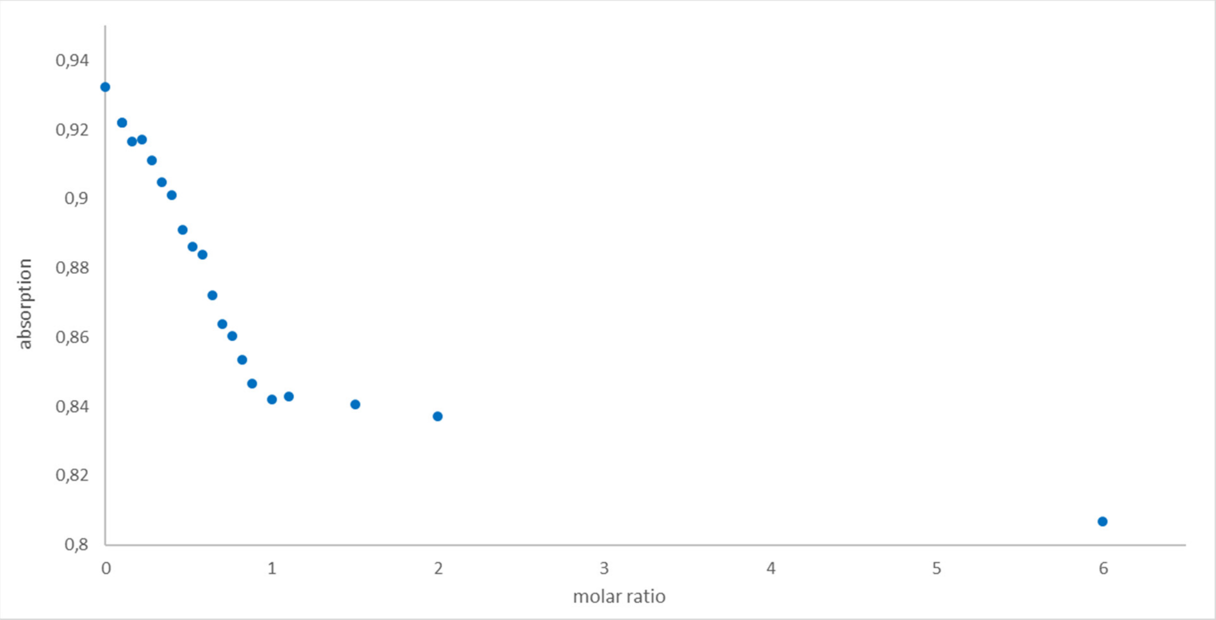

Figure S59. UV spectra and UV titration plot of compound **25** with Ba^{2+}.

Compound **25** with Sr^{2+}

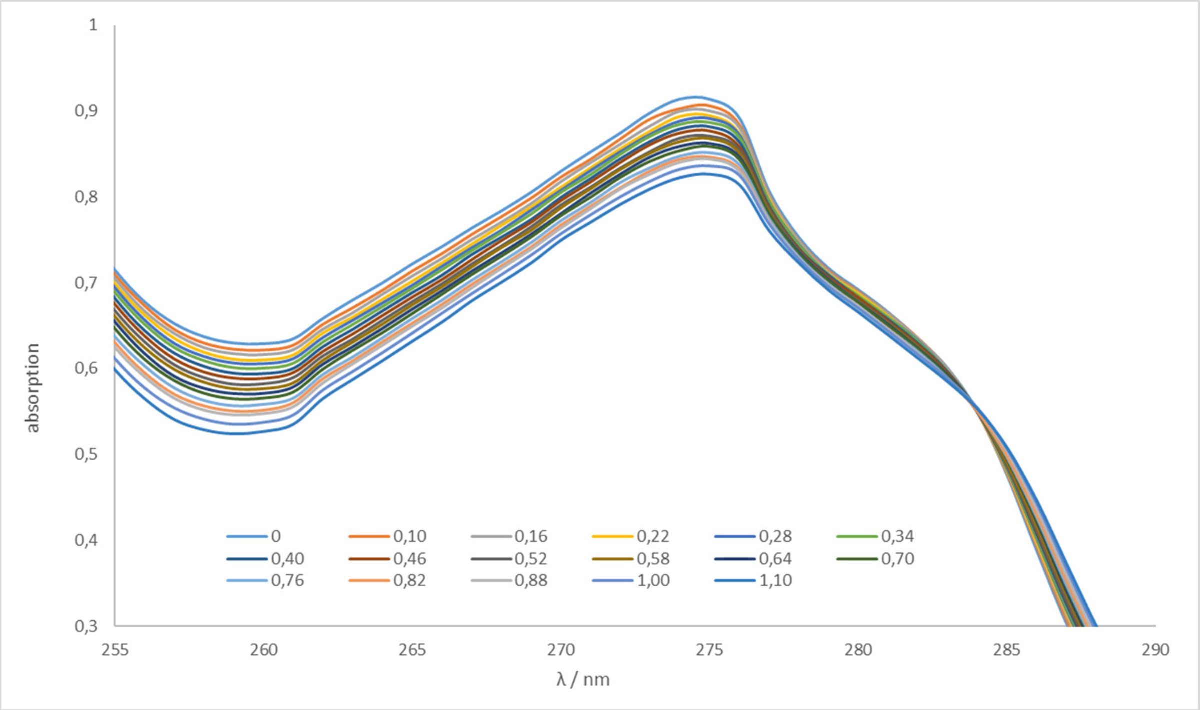

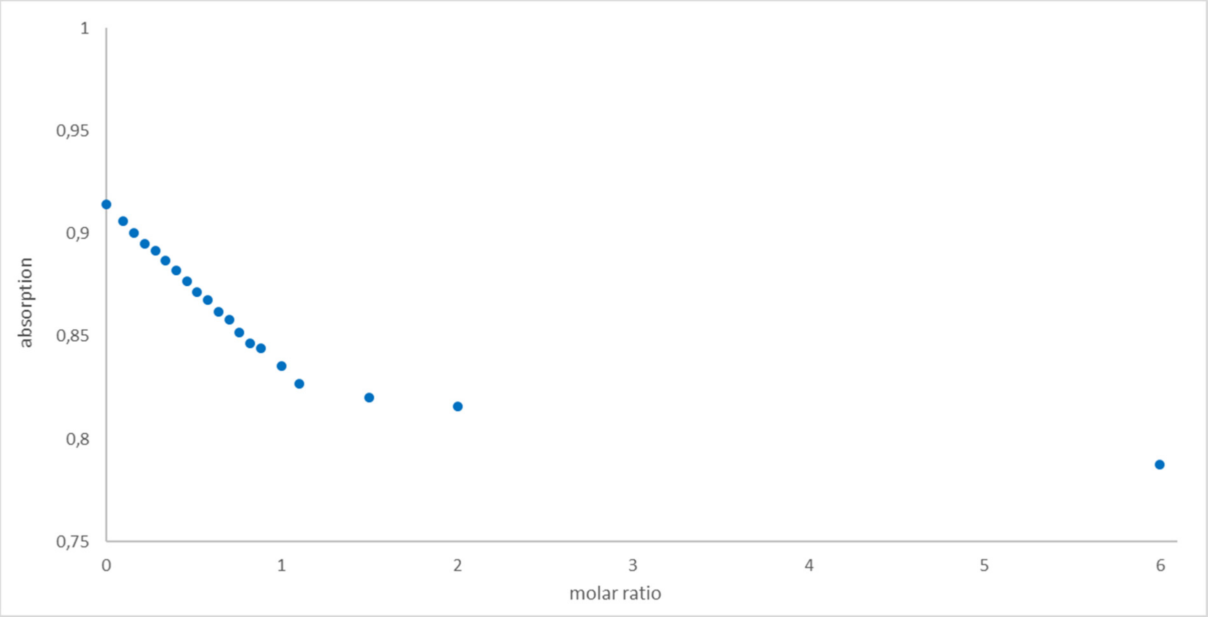

Figure S60. UV spectra and UV titration plot of compound **25** with Sr^{2+}.

Compound **25** with Pb^{2+}

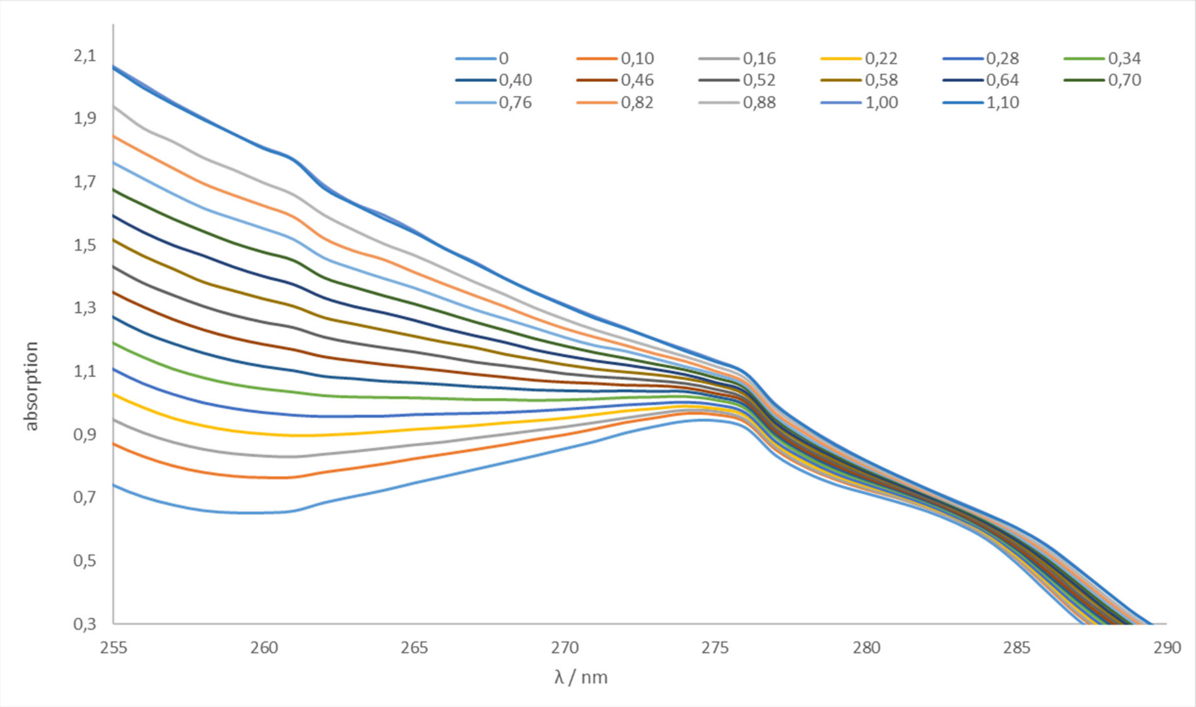

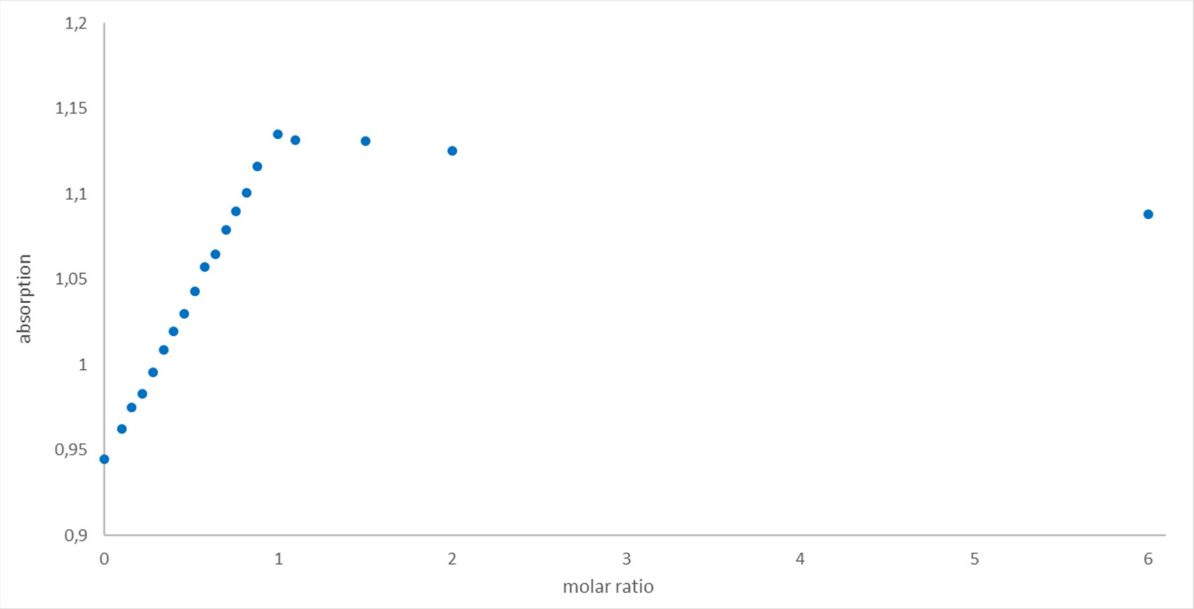

Figure S61. UV spectra and UV titration plot of compound **25** with Pb^{2+}.

Compound **27** with Ba^{2+}

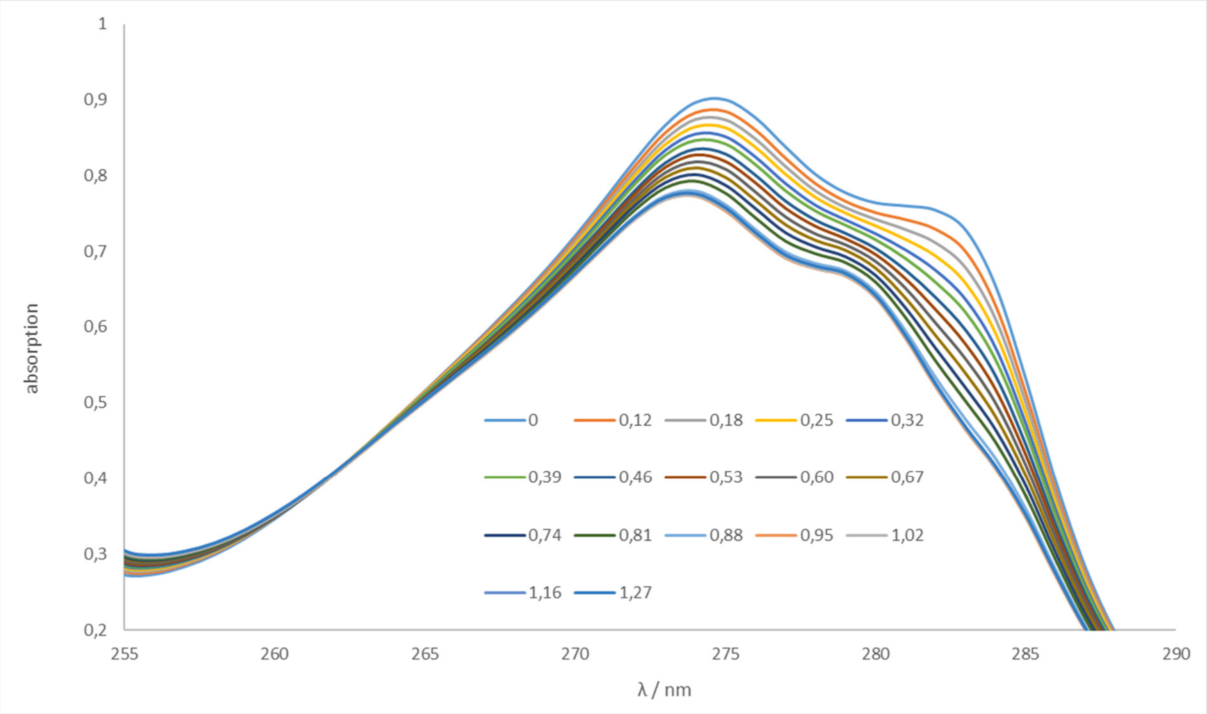

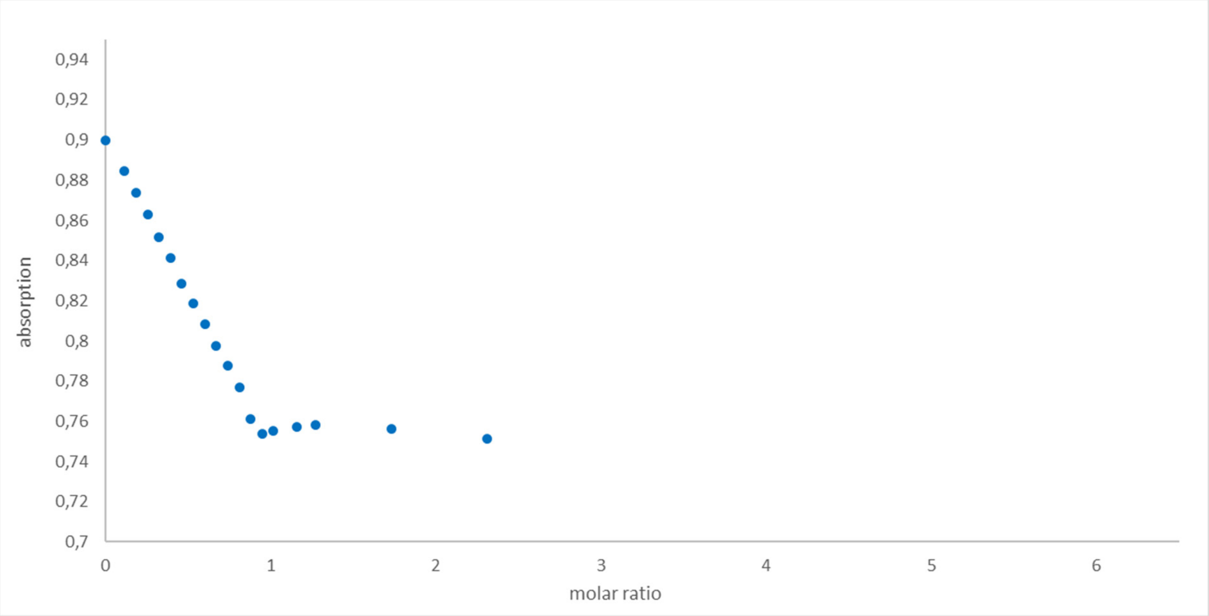

Figure S62. UV spectra and UV titration plot of compound **27** with Ba^{2+}.

Compound **27** with Sr^{2+}

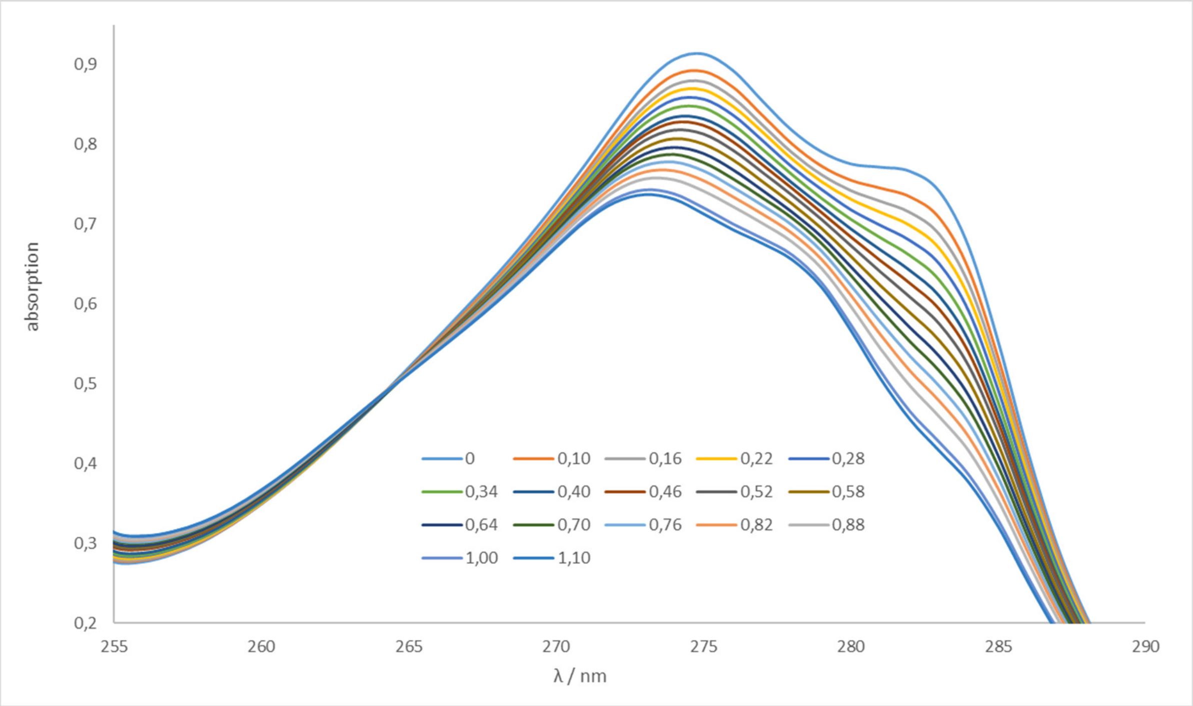

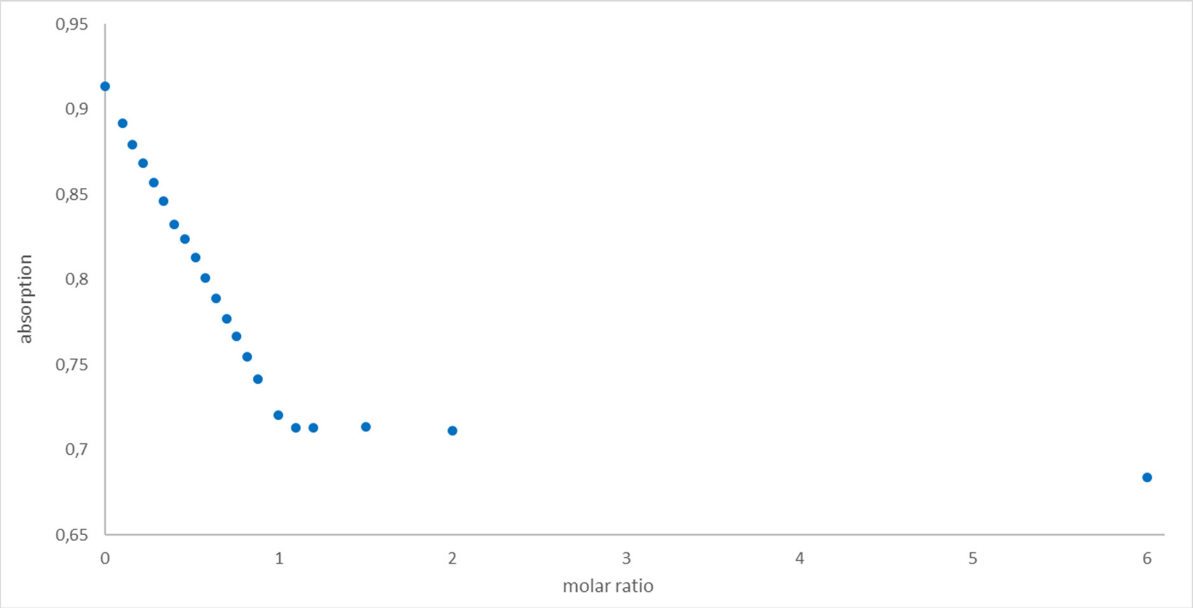

Figure S63. UV spectra and UV titration plot of compound **27** with Sr^{2+}.

Compound **27** with Pb^{2+}

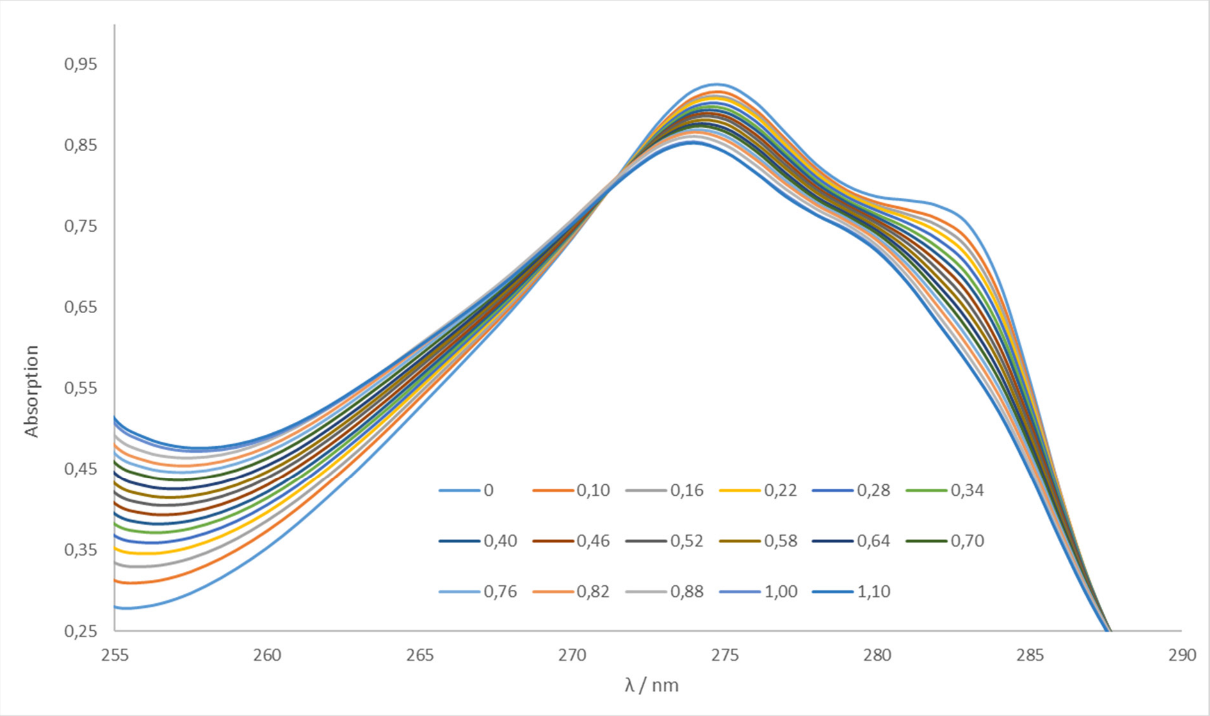

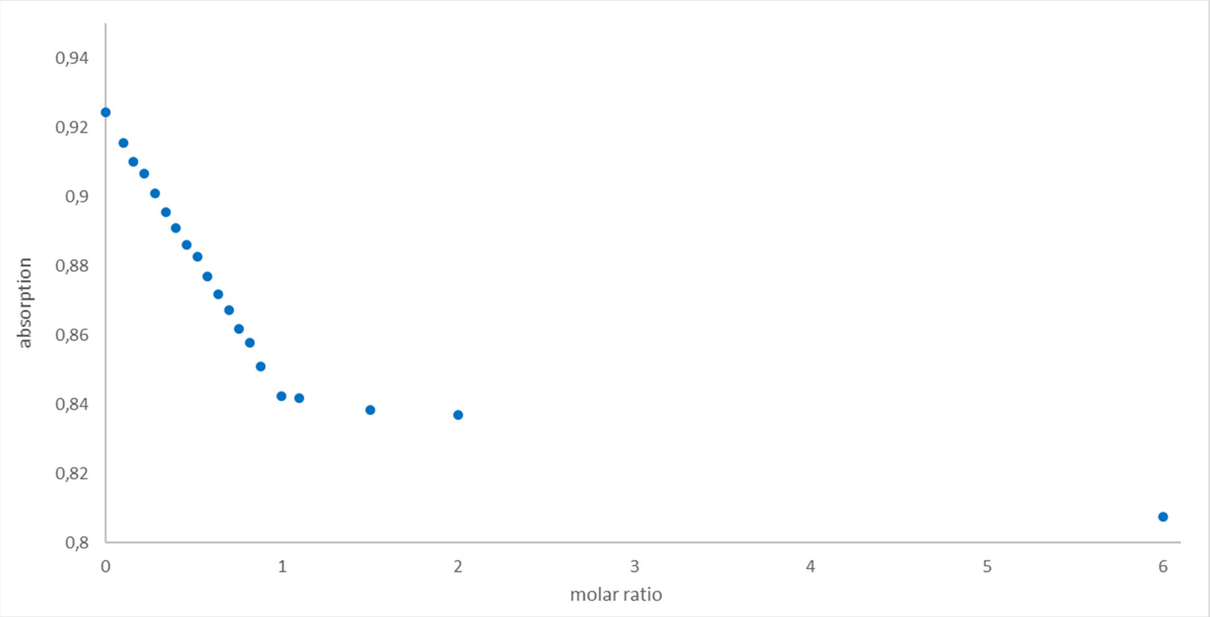

Figure S64. UV spectra and UV titration plot of compound **27** with Pb^{2+}.
